# Mechanochemical Sustainable Synthesis of Platinum(II) Complexes: Reactivity Inversion and Coordination Mode Control

**DOI:** 10.1021/acs.inorgchem.6c02094

**Published:** 2026-08-05

**Authors:** Leonardo Genesin, Talha Munir, Eleonora Aneggi, Daniele Zuccaccia

**Affiliations:** Dipartimento di Scienze Agroalimentari, Ambientali e Animali, Sezione di Chimica, 9316 Università di Udine, Via Cotonificio 108, Udine I-33100, Italy

## Abstract

Mechanochemistry is emerging as a sustainable alternative to conventional solution-based synthesis, yet its application to platinum­(II) coordination chemistry remains limited. Herein, we report a systematic investigation of the mechanochemical synthesis of Pt­(II) complexes bearing S-, N-, P-donor, and diene ligands. Under solvent-free ball-milling conditions, a broad range of structurally diverse complexes was efficiently prepared from commercially available precursors, often reaching quantitative conversion within minutes and requiring minimal workup. A key finding is the reversal of precursor reactivity relative to solution-phase chemistry, with PtCl_2_ consistently outperforming K_2_[PtCl_4_] under mechanochemical conditions. This behavior highlights the dominant influence of solid-state effects, where local mixing and metal–ligand bond activation govern reactivity rather than solubility and ligand-exchange kinetics. Mechanochemical conditions also led to atypical coordination outcomes, including the formation of κ^2^-terpyridine complexes instead of the expected κ^3^ species. Comparison with conventional synthetic methods revealed significantly improved green metrics, including lower E-factors, EMY, and reduced solvent consumption. Preliminary catalytic studies demonstrate the potential of selected Pt­(II) complexes in mechanochemically assisted oxidation reactions. Overall, this work establishes mechanochemistry as an efficient and versatile complementary approach to Pt­(II) complex synthesis and provides new insights into solid-state reactivity and coordination behavior.

## Graphical abstract

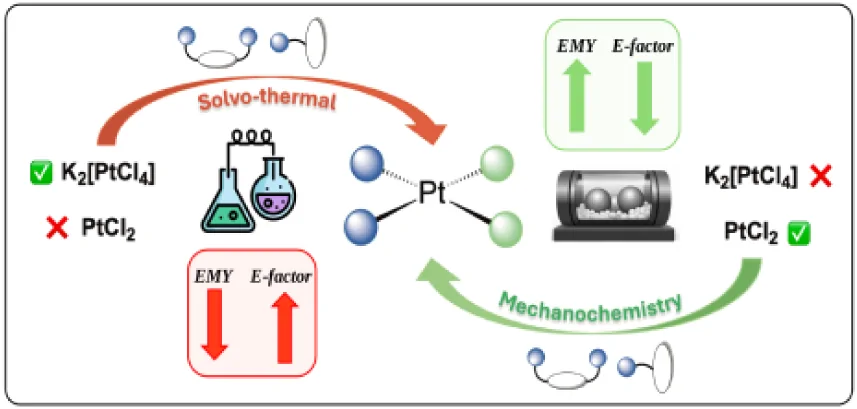

## 1 Introduction

Platinum­(II) complexes represent a cornerstone of coordination chemistry, owing to their established roles in catalysis,^1–5^ medicinal chemistry^6–9^ and luminescence.^10–14^ In particular, their relevance in anticancer therapy^15,16^ together with their rich coordination chemistry and diverse reactivity has sustained continuous interest in the development of reliable synthetic methodologies.^17^ Conventionally, Pt­(II) complexes are prepared via solution-based approaches that rely on organic solvents and in many cases the use of additives or auxiliaries to achieve satisfactory conversions.^18,19^ Although these methods are effective, they are often associated with significant solvent consumption and multistep procedures.

The increasing emphasis on sustainable practices has stimulated efforts to align platinum chemistry with the principles of Green Chemistry.^20^ These efforts have largely focused on catalytic applications, where the use of benign media, reduced solvent volumes and milder conditions has been explored. In contrast, the synthesis of Pt­(II) complexes has received comparatively less attention from a sustainability perspective. Commonly employed precursors such as K_2_[PtCl_4_],^21^ [Pt­(DMSO)_2_Cl_2_][Bibr ref19] or [Pt­(acac)_2_][Bibr ref22] are typically accessed through solution-phase protocols that involve prolonged reaction times, nonambient temperatures and multiple purification steps. As a result, these routes often remain resource-intensive and operationally demanding.

Mechanochemistry has emerged as a viable alternative to conventional solution-based synthesis, enabling chemical transformations under solvent-free or solvent-minimized conditions.^23–25^ This approach offers several advantages, including shorter reaction times, reduced waste generation and simplified workup procedures. Although mechanochemical methods have been widely applied in organic synthesis^26–28^ and materials chemistry,^29,30^ their use in coordination chemistry is comparatively recent. Nevertheless, a growing number of studies have demonstrated the successful preparation of late-transition metal complexes under mechanochemical conditions.^31–48^ However, systematic investigations addressing the scope, limitations, and mechanistic aspects of platinum­(II) complex formation in the solid state are still scarce.^47–57^

In this work, we present a comprehensive study on the mechanochemical synthesis of platinum­(II) complexes from commercially available precursors using a diverse set of ligands, including S-, N-, and P-donor systems as well as dienes, across different metal-to-ligand stoichiometries. Systematic variation of milling parameters, such as time and frequency, allowed the identification of key factors governing product formation. The resulting protocol provides a rapid and efficient route to Pt­(II) complexes under solvent-free conditions. Particular attention is devoted to the comparative reactivity of PtCl_2_ and K_2_[PtCl_4_], revealing an inversion of reactivity trends relative to solution chemistry. Furthermore, deviations from expected coordination behavior, such as κ^2^-coordination of terpyridine, are examined in the context of solid-state constraints. The mechanochemical protocols developed herein are evaluated against conventional solution-based methods in terms of reaction efficiency, operational simplicity, and environmental impact with green metrics, including the E-factor and the Effective Mass Yield (EMY). Collectively, the results provide both a practical and more sustainable framework for the synthesis of platinum­(II) complexes and insight into the factors governing reactivity and coordination under mechanochemical conditions.

## 2 Experimental Section

PtCl_2_ was purchased from Alfa Aesar Chemicals. K_2_[PtCl_4_] was purchased from Sigma-Aldrich. The reagents: 1,10-phenanthroline, 2,2′;6′,2″-terpyridine, acetonitrile, benzonitrile, acetic acid, triphenylphosphine, tris­[3,5-bis­(trifluoromethyl)­phenyl]­phosphine (P­(3,5-(CF_3_)_2_Ph)_3_), (9,9-Dimethyl-9H-xanthene-4,5-diyl)­bis­(diphenylphosphane) (Xantphos), 1,2-bis­(diphenylphosphino)­benzene (dppbz), 2,6-diisopropylphenylaniline, acenaphthoquinone, diphenylmethanol, Oxone and trisodium phosphate are Sigma-Aldrich products. Cycloocta-1,5-diene (COD) is produced by Ega-Chemie. Bis­(diphenylphosphino)­methane, -ethane, -propane, -butane, -ferrocene were purchased from Strem Chemicals, Inc. Bis­(diisopropylphosphino)­ferrocene (dippf) and (*R*)-2,2′-bis­(diphenylphosphino)-1,1′-binaphthyl ((*R*)-BINAP) were purchased from Alfa Aesar Chemicals. The ligand *N* ^1^,*N* ^2^-bis­(2,6-diisopropylphenyl)­acenaphthylene-1,2-diimine (BIAN) was synthesized from acenaphthoquinone and 2,6-diisopropylphenylaniline using a modified methodology already present in the literature.^58^ FTIR spectra were acquired in ATR mode using an Agilent Cary 630 FTIR spectrometer over the 4000–650 cm^–1^ spectral range. FTIR analysis was performed on freshly ground samples, as is, without further purification. Deuterated solvents chloroform (CDCl_3_), dimethyl sulfoxide (DMSO-*d*_6_) and methanol (CD_3_OD) used for NMR spectra were purchased from Eurisotop. The ^1^H, ^13^C­{^1^H}, ^19^F­{^1^H}, ^31^P­{^1^H}, ^195^Pt NMR spectra were acquired using a Bruker Avance III HD 400 MHz spectrometer equipped with a broadband 5 mm probe (1H/BBF iProbe) with a *z*-axis gradient (50 G/cm). Chemical shifts are reported in parts per million and calibrated to the solvent residue. Elemental analysis (C, H, N) was carried out with a Carlo Erba 1106 elemental analyzer. XRD patterns were collected on a Philips X’Pert diffractometer using Ni-filtered Cu–Kα radiation (40 kV, 40 mA). Data were acquired in the 2θ range of 5°–45° with a step size of 0.02° and a counting time of 15 s per step. Mechanochemical synthesis of all complexes was carried out using Retsch Ball Mill (MM 500 Vario) with 2 mL Eppendorf and zirconia balls (3 mm diameter, ∼0.4 g), zirconia jar of 10 mL and zirconia ball (10 mm diameter, ∼3.3 g). In an Eppendorf, appropriate amounts of the reactants were taken with 4 zirconia balls inside. To calculate the final mass of the products, the Eppendorf, or zirconia jar, and balls were appropriately calibrated. The Eppendorf is then closed (under Argon when required to avoid oxidation by air), airtight sealed using parafilm and then milled at suitable milling conditions (as highlighted below for each synthesis). Approximately 5 mg of the solid sample is extracted from Eppendorf (or jar) and dissolved in a suitable deuterated solvent for NMR analysis. A second portion is utilized for elemental analysis.

### 2.1 MC Synthesis of [Pt­(DMSO)_2_Cl_2_] (**1**)

*Method A* , PtCl_2_ (20.0 mg, 0.0752 mmol) and dimethyl sulfoxide (10.7 μL, 11.7 mg, 0.1504 mmol) were milled at 30 Hz for 30 min in 2 mL Eppendorf and 4 zirconia balls (3 mm). Conversion: >99%. Selectivity: 95% of *cis*-[Pt­(DMSO)_2_Cl_2_] (**1a**); 5% of *trans*-[Pt­(DMSO)_2_Cl_2_] (**1b**) (selectivity determined by ^1^H NMR in CDCl_3_). *Method B* , K_2_[PtCl_4_] (29.0 mg, 0.0699 mmol) and dimethyl sulfoxide (10.0 μL, 10.9 mg, 0.1397 mmol) were milled at 30 Hz for 90 min in 2 mL Eppendorf and 4 zirconia balls (3 mm). The product was then washed with 2 mL of water and dried at low pressure. Conversion: 94%. Selectivity: 87% of *cis*-[Pt­(DMSO)_2_Cl_2_] (**1a**); 7% of *trans*-[Pt­(DMSO)_2_Cl_2_] (**1b**) (selectivity determined by ^1^H NMR in CDCl_3_).

*cis*-[Pt­(DMSO)_2_Cl_2_] (**1a**): ^1^H NMR (400.1 MHz, CDCl_3_, 298 K) δ 3.54 (app. t, *J* = 11.0 Hz, 12H, −CH_3_). ^13^C­{^1^H} NMR (100.6 MHz, CDCl_3_, 298 K) δ 44.89 (Figures S5 and S6). *trans*-[Pt­(DMSO)_2_Cl_2_] (**1b**): ^1^H NMR (400.1 MHz, CDCl_3_, 298 K) δ 3.42 (app. t, *J* = 10.0 Hz, 12H, −CH_3_). FTIR (ATR, cm^–1^): 1154–1131 s (v_S=O_), 735–689 m (ν_C–S_) (Figure S100).

### 2.2 MC Synthesis of [Pt­(COD)­Cl_2_] (**2**)

PtCl_2_ (75.0 mg, 0.2820 mmol) and cycloocta-1,5-diene (42 μL, 36.7 mg, 0.3394 mmol) were milled at 30 Hz for 60 min in 10 mL zirconia jar and 1 zirconia ball (10 mm) under air atmosphere. The product was then extracted with 5 mL of dichloromethane filtered through cotton, washed with other 2 mL of dichloromethane and dried at low pressure. The powder obtained is then washed three times with 3 mL of pentane and dried at low pressure. Yield: 96% (107.2 mg).

[Pt­(COD)­Cl_2_] (**2**): ^1^H NMR (400.1 MHz, CDCl_3_, 298 K) δ 5.62 (app. t, *J* = 33.3 Hz, 4H); 2.83–2.66 (m, 4H); 2.36–2.15 (m, 4H). ^13^C­{^1^H} NMR (100.6 MHz, CDCl_3_, 298 K) δ 100.02 (app. t, *J*_PtC_ = 76.0 Hz, CH of COD); 30.92 (−CH_2_– of COD) (Figures S7 and S8). FTIR (ATR, cm^–1^): 1476 s (ν_C=C_), 1425 s (ν_C=C_), 1340 s, 1180 m, 1008 s (δ_CH_), 832 (δ_CH_) (Figure S101). Elemental Analysis: calculated (%) for (C_8_H_12_Cl_2_Pt): 25.68; H, 3.23; Cl, 18.95; Pt, 52.14. Found (%): C, 25.16; H, 3.21.

### 2.3 MC Synthesis of [Pt­(phen)­Cl_2_] (**3**)

PtCl_2_ (15.0 mg, 0.0564 mmol) and 1,10-phenanthroline (10.2 mg, 0.0564 mmol) were milled at 30 Hz for 60 min in 2 mL Eppendorf and 4 zirconia balls (3 mm) under argon atmosphere. Yield: >99% (25.0 mg).

[Pt­(phen)­Cl_2_] (**3**): ^1^H NMR (400.1 MHz, DMSO-*d*_6_, 298 K) δ 9.70 (dd, *J*_HH_ = 5.5, 1.2 Hz, 2H); 9.04 (dd, *J*_HH_ = 8.2, 1.2 Hz, 2H); 8.29 (s, 2H); 8.17 (dd, *J*_HH_ = 8.2, 5.5 Hz, 2H). ^13^C­{^1^H} NMR (100.6 MHz, DMSO-*d*_6_, 298 K) δ 149.47 (CH of phen); 140.03 (CH of phen); 131.09 (quaternary C of phen); 128.23 (CH of phen); 126.60 (CH of phen) (Figures S9 and S10). FTIR (ATR, cm^–1^): 1463 m/w, 1366 m/w, 840 m, 706 m (δ_aromCH_) (Figure S102). Elemental Analysis: calculated (%) for (C_12_H_8_Cl_2_N_2_Pt): C, 32.30; H, 1.81; Cl, 15.89; N, 6.28; Pt, 43.72. Found (%): C, 32.42; H, 1.84; N, 6.57.

### 2.4 MC Synthesis of [Pt­(κ^2^-terpy)­Cl_2_] (**4**)

*Method A* , PtCl_2_ (15.0 mg, 0.0563 mmol) and 2,2′;6′,2″-terpyridine (13.2 mg, 0.0563 mmol) were milled at 30 Hz for 90 min in 2 mL Eppendorf and 4 zirconia balls (3 mm) under air atmosphere. Yield: >99% (28.1 mg). *Method B* , K_2_[PtCl_4_] (20.0 mg, 0.0482 mmol) and 2,2′;6′,2″-terpyridine (11.2 mg, 0.0482 mmol) were milled at 30 Hz for 120 min in 2 mL Eppendorf and 4 zirconia balls (3 mm) under air atmosphere. The product was then washed with 2 mL of water and dried at low pressure. Yield: >99% (24.1 mg).

[Pt­(κ^2^-terpy)­Cl_2_] (**4**): ^1^H NMR (400.1 MHz, DMSO-*d*_6_, 298 K) δ 8.96 (d, *J*_HH_ = 5.6 Hz, 1H); 8.73 (d, *J*_HH_ = 4.5 Hz, 1H); 8.68–8.60 (m, 3H); 8.51 (td, *J*_HH_ = 7.9, 1.5 Hz, 1H); 8.45 (d, *J*_HH_ = 7.8 Hz, 1H); 8.15–8.08 (m, 1H); 8.03 (td, *J*_HH_ = 7.8, 1.8 Hz, 1H); 7.97 (ddd, *J*_HH_ = 7.4, 5.6, 1.4 Hz, 1H); 7.56–7.47 (m, 1H). ^13^C­{^1^H} NMR (100.6 MHz, DMSO-*d*_6_, 298 K) δ 158.84 (quaternary C of terpy); 154.90 (quaternary C of terpy); 151.80 (CH of terpy); 149.66 (CH of terpy); 143.16 (CH of terpy); 142.50 (CH of terpy); 139.10 (CH of terpy); 138.16 (CH of terpy); 129.64 (CH of terpy); 126.29 (CH of terpy); 124.97 (CH of terpy); 124.87 (CH of terpy); 121.30 (CH of terpy) ^195^Pt NMR (85.9 MHz, DMSO-*d*_6_, 298 K) δ −2953.68 (Figures S11–S18). FTIR (ATR, cm^–1^): 1602 m/w, 1450 m/w, 771 m (δ_aromCH_) (Figure S103). Elemental Analysis: calculated (%) for (C_15_H_11_Cl_2_N_3_Pt): C, 36.09; H, 2.22; Cl, 14.20; N, 8.42; Pt, 39.07. Found (%): C, 36.14; H, 2.32; N, 8.67.

### 2.5 MC Synthesis of [Pt­(BIAN)­Cl_2_] (**5**)

PtCl_2_ (10.0 mg, 0.0376 mmol) and *N* ^1^,*N* ^2^-bis­(2,6-diisopropylphenyl)­acenaphthylene-1,2-diimine (BIAN) (18.8 mg, 0.0376 mmol) were milled at 30 Hz for 90 min in 2 mL Eppendorf and 4 zirconia balls (3 mm) under argon atmosphere. The product was then washed with 3 mL of diethyl ether and dried at low pressure. Yield: 88% (25.3 mg).

[Pt­(BIAN)­Cl_2_] (**5**): ^1^H NMR (400.1 MHz, CDCl_3_, 298 K) δ 8.28 (d, *J*_HH_ = 8.3 Hz, 2H, arom.); 7.59–7.51 (m, 2H, arom.); 7.47–7.40 (m, 6H, arom.); 6.69 (d, *J*_HH_ = 7.3 Hz, 2H, arom.); 3.52 (hept, *J*_HH_ = 6.8 Hz, 4H, −CH−); 1.49 (d, *J*_HH_ = 6.7 Hz, 12H, −CH_3_); 0.96 (d, *J* = 6.9 Hz, 12H, −CH_3_). ^13^C­{^1^H} NMR (100.6 MHz, CDCl_3_, 298 K) δ 164.90 (quaternary C); 140.97 (quaternary C, *o*-C of phenyl-*i*Pr); 140.69 (quaternary C); 139.99 (quaternary C, *o*-C of phenyl-*i*Pr); 132.43 (quaternary C, arom.); 131.28 (CH, arom.); 129.85 (CH, arom.); 129.54 (CH, arom.); 124.35 (*m*-CH of phenyl); 124.13 (CH, arom.); 28.86 (−CH– of *i*Pr); 23.98 (−CH_3_ of *i*Pr); 23.69 (−CH_3_ of *i*Pr) (Figures S19 and S20). FTIR (ATR, cm^–1^): 1655 w (ν_C=N_), 1601 w (ν_C=N_), 1458 m/w, 832 m/w, 778 m/w (δ_aromCH_), 702 m/w (δ_aromCH_) (Figure S104). Elemental Analysis: calculated (%) for (C_36_H_40_Cl_2_N_2_Pt): C, 56.42; H, 5.26; Cl, 9.25; N, 3.66; Pt, 25.46. Found (%): C, 55.97; H, 5.13; N, 3.04.

### 2.6 MC Synthesis of [Pt­(PPh_3_)_2_Cl_2_] (**6**)

*Method A* , PtCl_2_ (10.0 mg, 0.0376 mmol) and triphenylphosphine (19.7 mg, 0.0752 mmol) were milled at 30 Hz for 15 min in 2 mL Eppendorf and 4 zirconia balls (3 mm) under argon atmosphere. Conversion: >99%. Selectivity: 80% of *cis*-[Pt­(PPh_3_)_2_Cl_2_] (**6a**); 20% of *trans*-[Pt­(PPh_3_)_2_Cl_2_] (**6b**) (selectivity determined by ^31^P­{^1^H} NMR in CDCl_3_). *Method B* , K_2_[PtCl_4_] (10.0 mg, 0.0241 mmol) and triphenylphosphine (12.64 mg, 0.0241 mmol) were milled at 30 Hz for 60 min in 2 mL Eppendorf and 4 zirconia balls (3 mm) under argon atmosphere. The product was then washed with 2 mL of water and dried at low pressure. Conversion: 88%. Selectivity: 87% of *cis*-[Pt­(PPh_3_)_2_Cl_2_] (**6a**) (selectivity determined by ^1^H NMR in CDCl_3_).

[Pt­(PPh_3_)_2_Cl_2_] (**6a**): ^1^H NMR (400.1 MHz, CDCl_3_, 298 K) δ 7.54–7.46 (m, 12H, *o*-H of phenyl); 7.36–7.30 (m, 6H, *p*-H of phenyl); 7.18 (td, *J* = 7.8, 2.2 Hz, 12H, *m*-H of phenyl). ^13^C­{^1^H} NMR (100.6 MHz, CDCl_3_, 298 K) δ 134.83 (app. t, *J*_PC_ = 5.0 Hz, CH of phenyl); 130.75 (s, *p*-CH of phenyl); 129.51 (d, *J*_PC_ = 66.7 Hz, quaternary C of phenyl); 127.88 (app. t, *J*_PC_ = 5.5 Hz, CH of phenyl). ^31^P­{^1^H} NMR (162.0 MHz, CDCl_3_, 298 K) δ 14.30 (app. t, ^1^ *J*_PPt_ = 1834.9 Hz) (Figures S21–S23). FTIR (ATR, cm^–1^): 1434 m (ν_C=C_), 1092 m (ν_P–C_), 832 m/w, 742 m (δ_aromCH_), 686 m/s (δ_aromCH_) (Figure S105). Elemental Analysis: calculated (%) for (C_36_H_30_Cl_2_P_2_Pt): C, 54.69; H, 3.83; Cl, 8.97; P, 7.84; Pt, 24.68. Found (%): C, 54.81; H, 3.78.

### 2.7 MC Synthesis of *trans*-[Pt­(P­(3,5-(CF_3_)_2_Ph)_3_)_2_Cl_2_] (**7**)

PtCl_2_ (10.0 mg, 0.0376 mmol) and tris­[3,5-bis­(trifluoromethyl)­phenyl]­phosphine (P­(3,5-(CF_3_)_2_Ph)_3_) (50.4 mg, 0.0752 mmol) were milled at 30 Hz for 90 min in 2 mL Eppendorf and 4 zirconia balls (3 mm) under argon atmosphere. Yield: 91% (55.0 mg).

*trans*-[Pt­(P­(3,5-(CF_3_)_2_Ph)_3_)_2_Cl_2_] (**7**): ^1^H NMR (400.1 MHz, CDCl_3_, 298 K) δ 8.13 (s, 6H, *p*-H of phenyl); 8.11–8.04 (m, 8H, *o*-H of phenyl). ^13^C­{^1^H} NMR (100.6 MHz, CDCl_3_, 298 K) δ 134.07 (s, *o*-CH of phenyl); 132.80 (quaternary C, *m*-C of phenyl); 128.26 (t, *J*_PC_ = 29.2 Hz, quaternary C, C–P); 126.56 (s, *p*-CH of phenyl); 122.31 (q, *J*_FC_ = 273.8 Hz, −CF_3_). ^19^F­{^1^H} NMR (376.9 MHz, CDCl_3_, 298 K) δ −63.32 (s). ^31^P­{^1^H} NMR (162.0 MHz, CDCl_3_, 298 K) δ 22.51 (app. t, ^1^ *J*_PPt_ = 1391.8 Hz) (Figures S24–S27). FTIR (ATR, cm^–1^): 1355 s (ν_C–F_), 1277 s (ν_C–F_), 1125 s (ν_C–F_), 1098 s (ν_P–C_), 899 m/w, 699 m/s (δ_aromCH_), 683 s (δ_aromCH_) (Figure S106). Elemental Analysis: calculated (%) for (C_48_H_18_Cl_2_F_36_P_2_Pt): Elemental Analysis: C, 35.89; H, 1.13; Cl, 4.41; F, 42.57; P, 3.86; Pt, 12.14. Found (%): C, 35.12; H, 1.10.

### 2.8 MC Synthesis of [Pt­(Xantphos)­Cl_2_] (**8**)

PtCl_2_ (10.0 mg, 0.0376 mmol) and (9,9-Dimethyl-9H-xanthene-4,5-diyl)­bis­(diphenylphosphane) (Xantphos) (21.8 mg, 0.0376 mmol) were milled at 30 Hz for 20 min in 2 mL Eppendorf and 4 zirconia balls (3 mm) under argon atmosphere. Yield: >99% (31.6 mg).

[Pt­(Xantphos)­Cl_2_] (**8**): ^1^H NMR (400.1 MHz, CDCl_3_, 298 K) δ 7.64 (d, *J* = 7.7 Hz, 2H, arom.); 7.50–7.42 (m, 10H, arom.); 7.27–7.22 (m, 2H, arom.); 7.18–7.09 (m, 4H, arom.); 7.08–6.99 (m, 8H, arom.); 1.87 (s, 6H, −CH_3_). ^13^C­{^1^H} NMR (100.6 MHz CDCl_3_, 298 K) δ 150.60 (quaternary C, arom.); 135.79 (quaternary C, arom.); 134.60 (CH of phenyl); 130.16 (CH of phenyl); 129.14 (quaternary C of phenyl); 128.35 (CH, arom.); 127.95 (CH of phenyl); 127.66 (CH, arom.); 126.29 (quaternary C, arom.); 125.19 (CH arom.); 31.26 (quaternary C, alk.); 27.69 (−CH_3_). ^31^P­{^1^H} NMR (162.0 MHz, CDCl_3_, 298 K) δ 6.00 (app. t, ^1^ *J*_PPt_ = 1846.0 Hz) (Figures S28–S30). FTIR (ATR, cm^–1^): 1436 m/s (v_C=C_), 1404 s (v_C=C_), 1223 m (v_C–O–C_), 1095 m (v_P–C_), 1095 m (v_P–C_), 738 m/s (δ_aromCH_), 686 s (δ_aromCH_) (Figure S107). Elemental Analysis: calculated (%) for (C_39_H_32_Cl_2_OP_2_Pt): C, 55.46; H, 3.82; Cl, 8.39; O, 1.89; P, 7.33; Pt, 23.10. Found (%): C, 55.25; H, 3.77.

### 2.9 MC Synthesis of [Pt­(dppm)­Cl_2_] (**9**)

*Method A* , PtCl_2_ (15.0 mg, 0.0564 mmol) and bis­(diphenylphosphino)­methane (dppm) (21.7 mg, 0.0564 mmol) were milled at 30 Hz for 10 min in 2 mL Eppendorf and 4 zirconia balls (3 mm) under argon atmosphere. Yield: >99% (36.5 mg). *Method B* , K_2_[PtCl_4_] (15.0 mg, 0.0361 mmol) and bis­(diphenylphosphino)­methane (13.9 mg, 0.0361 mmol) were milled at 30 Hz for 90 min in 2 mL Eppendorf and 4 zirconia balls (3 mm) under argon atmosphere. The product was then washed with 2 mL of water and dried at low pressure. Yield: >99% (23.4 mg).

[Pt­(dppm)­Cl_2_] (**9**): ^1^H NMR (400.1 MHz, CDCl_3_, 298 K) δ 8.01–7.89 (m, 8H, phenyl); 7.58–7.42 (m, 12H, phenyl); 4.60–4.29 (2H, −CH_2_−). ^13^C­{^1^H} NMR (100.6 MHz, CDCl_3_, 298 K) δ 133.00 (app. t, *J*_PC_ = 5.6 Hz, CH of phenyl); 132.45 (s, *p*-CH of phenyl); 129.35 (app. t, *J*_PC_ = 6.2 Hz, CH of phenyl); 43.82 (−CH_2_−). ^31^P­{^1^H} NMR (162.0 MHz, CDCl_3_, 298 K) δ −64.17 (t,^1^ *J*_PPt_ = 1542.4 Hz) (Figures S31–S33). FTIR (ATR, cm^–1^): 1437 s (v_C=C_), 1103 s (v_P–C_), 999 m (v_P–C_), 803 s (δ_aromCH_), 738 s (δ_aromCH_), 617 s (δ_aromCH_), 689 vs (δ_aromCH_) (Figure S108). Elemental Analysis: calculated (%) for (C_25_H_22_Cl_2_P_2_Pt): C, 46.17; H, 3.41; Cl, 10.90; P, 9.52; Pt, 30.00. Found (%): C, 46.37; H, 3.49.

### 2.10 MC Synthesis of [Pt­(dppe)­Cl_2_] (**10**)

*Method A* , PtCl_2_ (15.0 mg, 0.0563 mmol) and bis­(diphenylphosphino)­ethane (dppe) (22.5 mg, 0.0563 mmol) were milled at 30 Hz for 15 min in 2 mL Eppendorf and 4 zirconia balls (3 mm) under argon atmosphere. Yield: >99% (37.3 mg). *Method B* , K_2_[PtCl_4_] (25.0 mg, 0.0602 mmol) and bis­(diphenylphosphino)­ethane (24.0 mg, 0.0602 mmol) were milled at 30 Hz for 120 min in 2 mL Eppendorf and 4 zirconia balls (3 mm) under argon atmosphere. The product was then washed with 2 mL of water and dried at low pressure. Yield: >99% (39.8 mg).

[Pt­(dppe)­Cl_2_] (**10**): ^1^H NMR (400.1 MHz, CDCl_3_, 298 K) δ 7.92–7.82 (m, 8H, phenyl); 7.58–7.43 (m, 12H, phenyl); 2.49–2.20 (m, 4H, −CH_2_−). ^13^C­{^1^H} NMR (100.6 MHz, CDCl_3_, 298 K) δ 133.99–133.13 (m, CH of phenyl); 132.05 (s, *p*-CH of phenyl); 129.26–128.75 (m, CH of phenyl); 126.90 (quaternary C of phenyl); 29.09–28.22 (−CH_2_−). ^31^P­{^1^H} NMR (162.0 MHz, CDCl_3_, 298 K) δ 41.16 (app. t, ^1^ *J*_PPt_ = 1810.5 Hz) (Figures S34–S36). FTIR (ATR, cm^–1^): 1436 s (v_C=C_), 1102 s (v_P–C_), 820 m/w, 705 s (δ_aromCH_), 688 s (δ_aromCH_), 658 vs (δ_aromCH_) (Figure S109). Elemental Analysis: calculated (%) for (C_26_H_24_Cl_2_P_2_Pt): C, 47.00; H, 3.64; Cl, 10.67; P, 9.32; Pt, 29.36. Found (%): C, 46.95; H, 3.59.

### 2.11 MC Synthesis of [Pt­(dppp)­Cl_2_] (**11**)

*Method A* , PtCl_2_ (15.0 mg, 0.0563 mmol) and bis­(diphenylphosphino)­propane (dppp) (23.3 mg, 0.0563 mmol) were milled at 30 Hz for 30 min in 2 mL Eppendorf and 4 zirconia balls (3 mm) under argon atmosphere. Yield: >99% (38.1 mg). *Method B* , K_2_[PtCl_4_] (15.0 mg, 0.0361 mmol) and bis­(diphenylphosphino)­propane (14.9 mg, 0.0361 mmol) were milled at 30 Hz for 120 min in 2 mL Eppendorf and 4 zirconia balls (3 mm) under argon atmosphere. The product was then washed with 2 mL of water and dried at low pressure. Yield: >99% (24.4 mg).

[Pt­(dppp)­Cl_2_] (**11**): ^1^H NMR (400.1 MHz, CDCl_3_, 298 K) δ 7.83–7.73 (m, 8H, phenyl); 7.54–7.35 (m, 12H, phenyl); 2.67–2.39 (m, 4H, P-CH_2_−); 2.16–1.96 (m, 2H, −CH_2_−). ^13^C­{^1^H} NMR (100.6 MHz, CDCl_3_, 298 K) δ 133.58 (app. t, *J*_PC_ = 4.8 Hz, CH of phenyl); 131.37 (s, *p*-CH of phenyl); 128.54 (app. t, *J*_PC_ = 5.5 Hz, CH of phenyl); 25.87–25.13 (P-CH_2_−); 19.00 (−CH_2_−). ^31^P­{^1^H} NMR (162.0 MHz, CDCl_3_, 298 K) δ −5.59 (app. t, ^1^ *J*_PPt_ = 1703.6 Hz) (Figures S37–S39). FTIR (ATR, cm^–1^): 1436 s (v_C=C_), 1098 s (v_P–C_), 743 s (δ_aromCH_), 690 vs (δ_aromCH_) (Figure S110). Elemental Analysis: calculated (%) for (C_27_H_26_Cl_2_P_2_Pt): C, 47.80; H, 3.86; Cl, 10.45; P, 9.13; Pt, 28.75. Found (%): C, 47.54; H, 3.71.

### 2.12 MC Synthesis of [Pt­(dppb)­Cl_2_] (**12**)

*Method A* , PtCl_2_ (15.0 mg, 0.0563 mmol) and bis­(diphenylphosphino)­butane (dppb) (24.0 mg, 0.0563 mmol) were milled at 30 Hz for 10 min in 2 mL Eppendorf and 4 zirconia balls (3 mm) under argon atmosphere. Yield: >99% (38.9 mg). *Method B* , K_2_[PtCl_4_] (15.0 mg, 0.0361 mmol) and bis­(diphenylphosphino)­butane (15.4 mg, 0.0361 mmol) were milled at 30 Hz for 90 min in 2 mL Eppendorf and 4 zirconia balls (3 mm) under argon atmosphere. The product was then washed with 2 mL of water and dried at low pressure. Yield: >99% (24.9 mg).

[Pt­(dppb)­Cl_2_] (**12**): ^1^H NMR (400.1 MHz, CDCl_3_, 298 K) δ 7.83–7.60 (m, 8H, phenyl); 7.60–7.34 (m, 12H, phenyl); 2.68–2.43 (m, 4H, P-CH_2_−); 1.95–1.77 (m, 4H, −CH_2_−). ^13^C­{^1^H} NMR (100.6 MHz, CDCl_3_, 298 K) δ 133.67 (CH of phenyl); 131.15 (*p*-CH of phenyl); 128.37 (CH of phenyl); 27.31 (d, *J*_PC_ = 40.5 Hz, P-CH_2_−); 23.01 (s, −CH_2_−). ^31^P­{^1^H} NMR (162.0 MHz, CDCl_3_, 298 K) δ 9.31 (app. t, ^1^ *J*_PPt_ = 1768.7 Hz) (Figures S40–S42). FTIR (ATR, cm^–1^): 1436 s (v_C=C_), 1100 s (v_P–C_), 742 s (δ_aromCH_), 691 vs (δ_aromCH_) (Figure S111). Elemental Analysis: calculated (%) for (C_28_H_28_Cl_2_P_2_Pt): C, 48.57; H, 4.08; Cl, 10.24; P, 8.95; Pt, 28.17. Found (%): C, 49.05; H, 4.23.

### 2.13 MC Synthesis of [Pt­(dppbz)­Cl_2_] (**13**)

PtCl_2_ (10.0 mg, 0.0376 mmol) and 1,2-bis­(diphenylphosphino)­benzene (dppbz) (16.8 mg, 0.0376 mmol) were milled at 30 Hz for 10 min in 2 mL Eppendorf and 4 zirconia balls (3 mm) under argon atmosphere. Yield: >99% (26.7 mg).

[Pt­(dppbz)­Cl_2_] (**13**): ^1^H NMR (400.1 MHz, CDCl_3_, 298 K) δ 7.77 (ddd, *J* = 12.6, 8.3, 1.4 Hz, 8H, arom.); 7.70–7.62 (m, 2H, arom.); 7.61–7.55 (m, 2H, arom.); 7.54–7.48 (m, 4H; arom.); 7.48–7.40 (m, 8H, arom.). ^13^C­{^1^H} NMR (100.6 MHz, CDCl_3_, 298 K) δ 133.93 (d, *J*_PC_ = 11.6 Hz, CH of phenyl); 133.23 (d, *J*_PC_ = 18.0 Hz, CH of benzene); 132.75 (s, CH of benzene); 131.92 (s, *p*-CH of phenyl); 128.87 (d, *J*_PC_ = 12.2 Hz, CH of phenyl). ^31^P­{^1^H} NMR (162.0 MHz, CDCl_3_, 298 K) δ 40.85 (app. t, ^1^ *J*_PPt_ = 1804.4 Hz) (Figures S43–S45). FTIR (ATR, cm^–1^): 1436 s (v_C=C_), 1098 s (v_P–C_), 745 s (δ_aromCH_), 689 vs (δ_aromCH_), 675 vs (δ_aromCH_) (Figure S112). Elemental Analysis: calculated (%) for (C_30_H_24_Cl_2_P_2_Pt): C, 50.56; H, 3.39; Cl, 9.95; P, 8.69; Pt, 27.41. Found (%): C, 49.98; H, 3.34.

### 2.14 MC Synthesis of [Pt­(dppf)­Cl_2_] (**14**)

*Method A* , PtCl_2_ (10.0 mg, 0.0376 mmol) and bis­(diphenylphosphino)­ferrocene (dppf) (20.8 mg, 0.0376 mmol) were milled at 30 Hz for 15 min in 2 mL Eppendorf and 4 zirconia balls (3 mm) under argon atmosphere. Yield: >99% (30.7 mg). *Method B* , K_2_[PtCl_4_] (15.0 mg, 0.0361 mmol) and bis­(diphenylphosphino)­ferrocene (20.0 mg, 0.0361 mmol) were milled at 30 Hz for 120 min in 2 mL Eppendorf and 4 zirconia balls (3 mm) under argon atmosphere. The product was then washed with 2 mL of water and dried at low pressure. Yield: >99% (29.5 mg).

[Pt­(dppf)­Cl_2_] (**14**): ^1^H NMR (400.1 MHz, CD_3_OD, 298 K) δ 7.85 (dd, *J*_PH_ = 11.9, 7.7 Hz, 8H, phenyl); 7.58–7.30 (m, 12H, phenyl); 4.51 (s, br, 4H, Cp^–^); 4.27 (s, br, 4H, Cp^–^). ^31^P­{^1^H} NMR (162.0 MHz, CD_3_OD, 298 K) δ 13.08 (app. t, ^1^ *J*_PPt_ = 1900.0 Hz) (Figures S46–S48). FTIR (ATR, cm^–1^): 1436 s (v_C=C_), 1095 s (v_P–C_), 743 s (δ_aromCH_), 689 vs (δ_aromCH_) (Figure S113). Elemental Analysis: calculated (%) for (C_34_H_28_Cl_2_FeP_2_Pt): C, 49.78; H, 3.44; Cl, 8.64; Fe, 6.81; P, 7.55; Pt, 23.78. Found (%): C, 48.02; H, 3.47.

### 2.15 MC Synthesis of [Pt­(dippf)­Cl_2_] (**15**)

*Method A* , PtCl_2_ (10.0 mg, 0.0376 mmol) and bis­(diisopropylphosphino)­ferrocene (dippf) (15.7 mg, 0.0376 mmol) were milled at 30 Hz for 20 min in 2 mL Eppendorf and 4 zirconia balls (3 mm) under argon atmosphere. Yield: >99% (25.6 mg). *Method B* , K_2_[PtCl_4_] (15.0 mg, 0.0361 mmol) and bis­(diisopropylphosphino)­ferrocene (15.1 mg, 0.0361 mmol) were milled at 30 Hz for 120 min in 2 mL Eppendorf and 4 zirconia balls (3 mm) under argon atmosphere. The product was then washed with 2 mL of water and dried at low pressure. Yield: >99% (24.6 mg).

[Pt­(dippf)­Cl_2_] (**15**): ^1^H NMR (400.1 MHz, CD_3_OD, 298 K) δ 5.80 (ddt, *J* = 17.0, 10.2, 6.7 Hz, 4H, −CH−); 4.65 (s, 4H, Cp^–^); 4.56 (s, 4H, Cp^–^); 1.33–1.27 (m, 12H, −CH_3_), 0.90 (t, *J* = 6.9 Hz, 12H, −CH_3_). ^31^P­{^1^H} NMR (162.0 MHz, CD_3_OD, 298 K) δ 30.71 (app. t, ^1^ *J*_PPt_ = 1909.5 Hz) (Figures S49 and S50). FTIR (ATR, cm^–1^): 2950 s (v_C–H_), 2924 s (v_C–H_), 2892 (v_C–H_), 2866 s (v_C–H_), 1458 s (v_C=C_), 1381 m, 1364 m, 1244 m, 1157 s (v_P–C_), 1030 vs (v_P–C_), 881 s (δ_CH_), 826 s (δ_CH_) (Figure S114). Elemental Analysis: calculated (%) for (C_22_H_36_Cl_2_FeP_2_Pt): C, 38.61; H, 5.30; Cl, 10.36; Fe, 8.16; P, 9.05; Pt, 28.51. Found (%): C, 38.22; H, 5.22.

### 2.16 MC Synthesis of [Pt­((*R*)-BINAP)­Cl_2_] (**16**)

PtCl_2_ (10.0 mg, 0.0376 mmol) and (*R*)-2,2′-bis­(diphenylphosphino)-1,1′-binaphthyl ((*R*)-BINAP) (23.4 mg, 0.0376 mmol) were milled at 30 Hz for 15 min in 2 mL Eppendorf and 4 zirconia balls (3 mm) under argon atmosphere. Yield: >99% (33.2 mg).

[Pt­((*R*)-BINAP)­Cl_2_] (**16**): ^1^H NMR (400.1 MHz, CDCl_3_, 298 K) δ 7.89–7.78 (m, 8H); 7.75–7.32 (m, 14H); 7.17–7.10 (m, 2H); 6.89–6.58 (m, 8H). ^13^C­{^1^H} NMR (100.6 MHz, CDCl_3_, 298 K) δ 135.52; 133.80 (quaternary C); 132.99 (quaternary C); 131.00; 130.34; 129.56 (quaternary C); 128.98; 127.71; 127.53; 127.25; 126.57; 126.24 (quaternary C). ^31^P­{^1^H} NMR (162.0 MHz, CDCl_3_, 298 K) δ 9.87 (app. t, ^1^ *J*_PPt_ = 1832.9 Hz) (Figures S51–S53). FTIR (ATR, cm^–1^): 1437 s (v_C=C_), 1095 s (v_P–C_), 813 m, 742 s (δ_aromCH_), 693 vs (δ_aromCH_) (Figure S115). Elemental Analysis: calculated (%) for (C_44_H_32_Cl_2_P_2_Pt): C, 59.47; H, 3.63; Cl, 7.98; P, 6.97; Pt, 21.95. Found (%): C, 58.73; H, 3.55.

The acquired NMR spectra, detailed experimental procedures, and additional experimental data are included in the Supporting Information (SI).

## 3 Results and Discussion

### 3.1 Mechanochemical Synthesis of Platinum­(II) Complexes with DMSO

Following our previous work on the mechanochemical synthesis of Pd­(II) complexes,^43^ the same synthetic strategy was employed to obtain square-planar platinum­(II) complexes. At first, our study was focused on the coordination ability of dimethyl sulfoxide (DMSO). The first attempt to synthesize the complex [Pt­(DMSO)_2_Cl_2_] (**1**) was carried out in Eppendorf for 30 min of milling a 1:2 mixture of PtCl_2_ and DMSO in air. The frequency chosen for milling was 30 Hz, which very often resulted as the best parameter (Scheme 1).

**Scheme 1:**
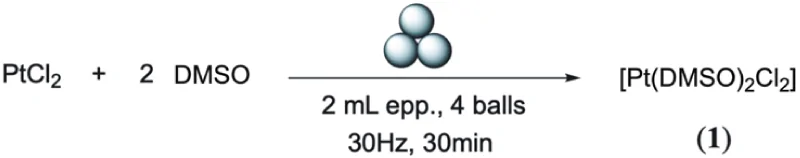
MC Synthesis of [Pt­(DMSO)_2_Cl_2_] (**1**)

After 30 min, complete conversion was observed with a selectivity of 95% for the *cis*-isomer (**1a**) and 5% for the *trans*-isomer (**1b**). The NMR characterization data agree with those reported in the literature.^59^ This result is consistent with the known greater stability of **1a** isomer,^59^ given the very stringent mechanochemical reaction conditions.

The same reaction was then performed with K_2_[PtCl_4_], a widely used precursor in Pt­(II) chemistry and preferred over PtCl_2_ in solution. Under the same mechanochemical reaction conditions, surprisingly, the reaction showed poorer performance. After 45 min, the reaction stopped at 81% conversion (**1a**: 84%, **1b**: 16%). Given this difference in terms of reactivity of the two different Pt­(II) precursors, a reactivity study was performed to monitor the reaction over time, analyzing the conversion of both precursors and selectivity. The results of these tests are shown in Figures 1 and 2.

**Figure 1:**
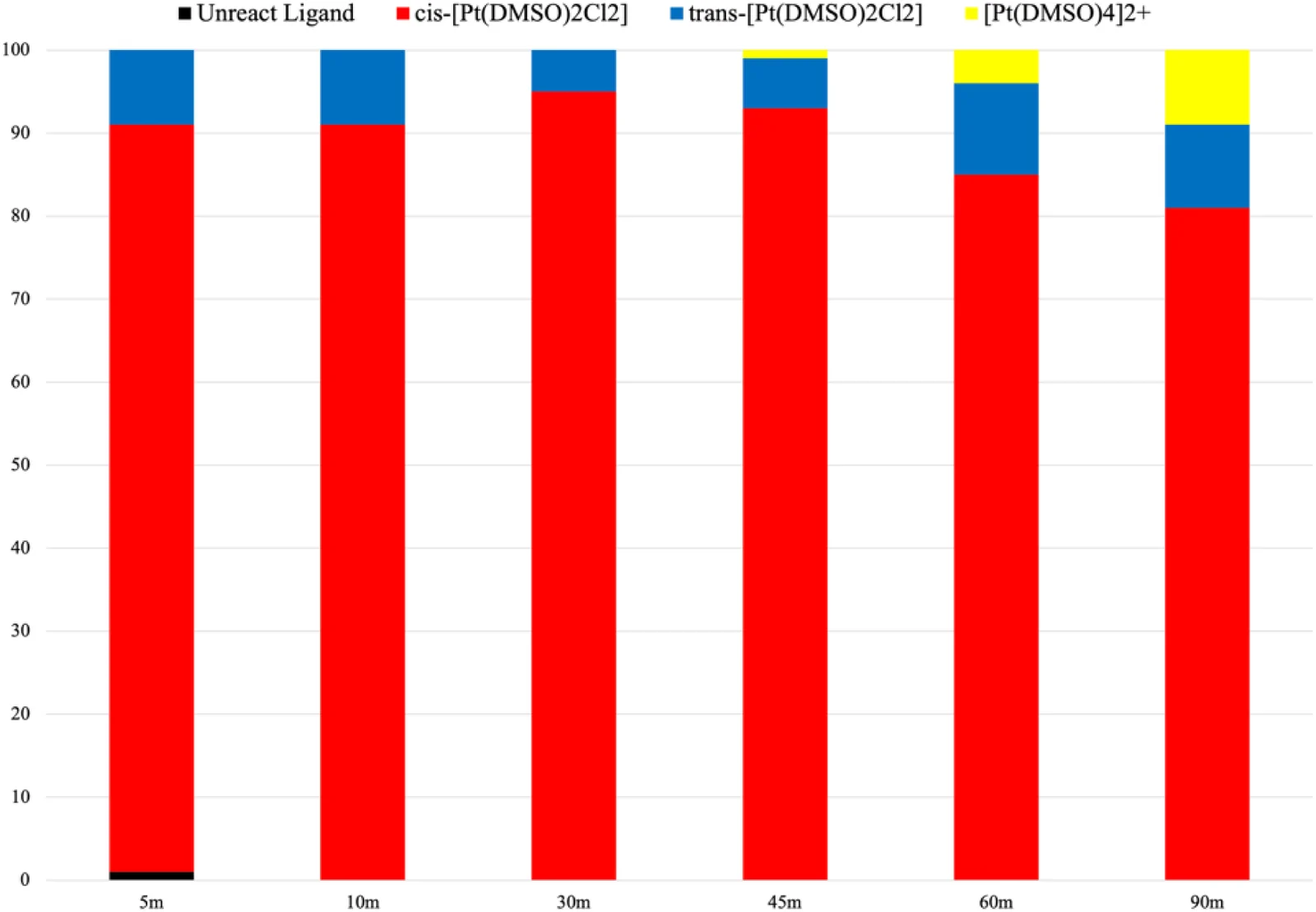
Conversion of PtCl_2_ into [Pt­(DMSO)_2_Cl_2_] (**1**).

**Figure 2:**
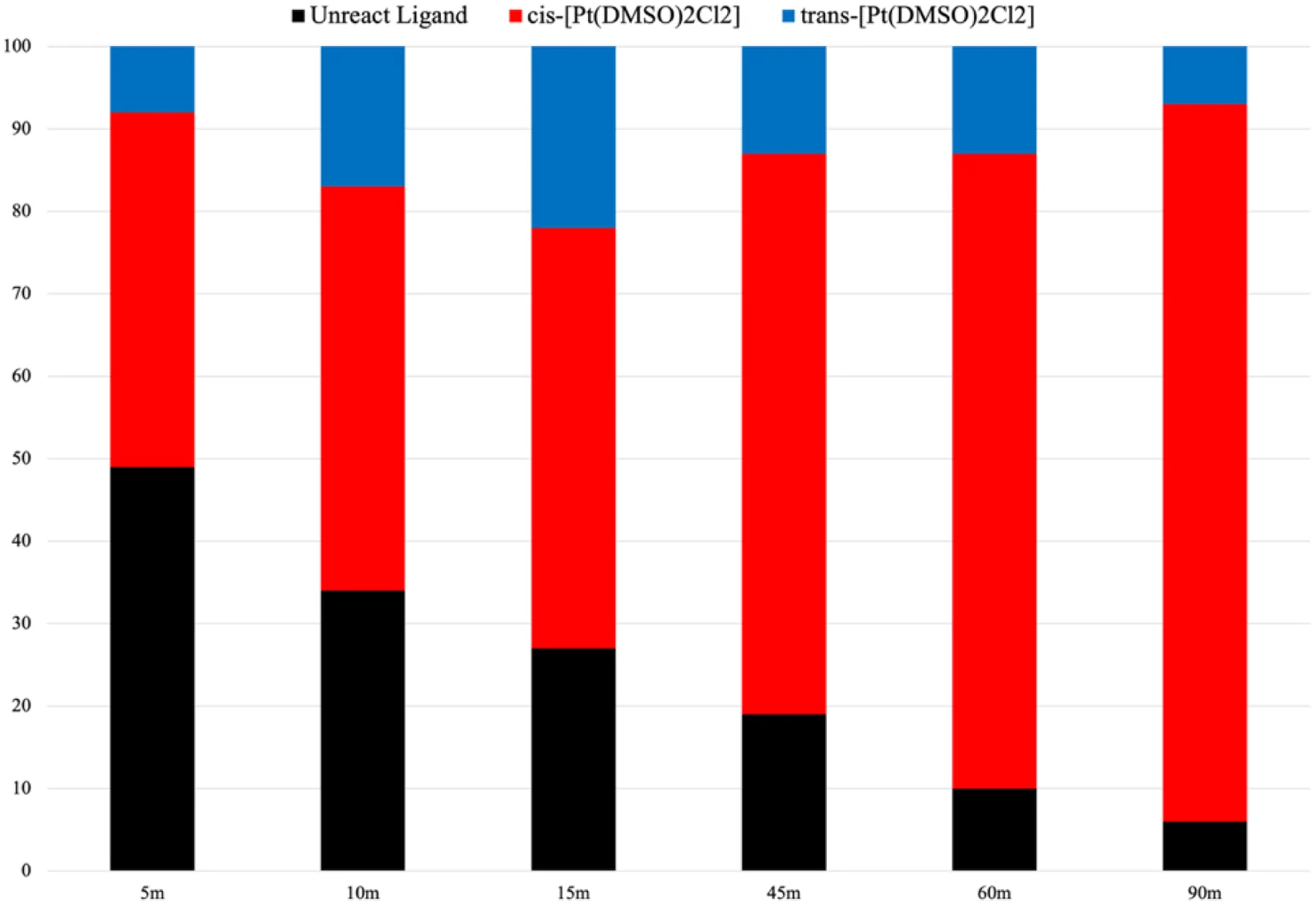
Conversion of K_2_[PtCl_4_] into [Pt­(DMSO)_2_Cl_2_] (**1**).

When PtCl_2_ is used and DMSO is added in a 2:1 molar ratio, the reaction is complete within 10 min. The selectivity toward **1a** reaches its maximum of 95% after 30 min. Longer reaction times result in the increasing formation of the unstable species [Pt­(DMSO)_4_]^2+^.^59^ Thus, stopping the reaction after 30 min is the best choice starting from PtCl_2_.

Starting from K_2_[PtCl_4_] instead of PtCl_2_, the higher conversion of 94% of the precursor into **1** is reached after 90 min. At this time, the **1a** reaches the maximum amount over **1b** (93:7), while no [Pt­(DMSO)_4_]^2+^ is detected, even for reaction times longer than 90 min.

The markedly different reactivity observed for PtCl_2_ and K_2_[PtCl_4_] under mechanochemical conditions can be rationalized by considering the distinct solid-state properties of the two precursors. In contrast to solution-phase chemistry, where reactivity is largely governed by solubility and ligand exchange kinetics, mechanochemical transformations are controlled by solid-state factors such as metal–ligand bond activation and interparticle contact. PtCl_2_ adopts a polymeric structure in the solid state, in which platinum centers are bridged by chloride ligands. Under ball-milling conditions, mechanical impacts induce metal–ligand bond activation, generating coordinatively accessible Pt­(II) sites. This process effectively activates the precursor by exposing reactive metal centers that can readily undergo ligand coordination. In addition, the repeated fracture and reagglomeration of particles during milling promote intimate local mixing between PtCl_2_ and the ligand, facilitating rapid solid–solid reactions.

In contrast, K_2_[PtCl_4_] consists of a more stable ionic lattice, in which discrete [PtCl_4_]^2**–**^ anions are embedded within a rigid crystal framework stabilized by electrostatic interactions. The higher lattice stability and the absence of extended metal–ligand connectivity reduce the efficiency of mechanical activation, limiting the formation of reactive sites. These observations highlight a fundamental difference between mechanochemical and solution-phase reactivity: whereas the latter is governed by solvation and molecular diffusion, the former is dictated by the ability of mechanical forces to activate metal bond and promote local contact between reactants. More broadly, this behavior underscores that mechanochemical reactivity is governed by bond activation processes driven by solid-state mixing rather than by solubility considerations, providing a framework to rationalize the distinct reactivity trends observed in this work.

### 3.2 Mechanochemical Synthesis of [Pt­(COD)­Cl_2_]

Considering the potential of mechanochemistry in the synthesis of widely used precursor complexes in a faster, more efficient and sustainable way, we engaged with the synthesis of the complex [Pt­(COD)­Cl_2_] (**2**) which is widely used in the coordination chemistry of Pt­(II) complexes. The synthetic attempts were performed with both PtCl_2_ and K_2_[PtCl_4_] precursors by milling them with 1 equiv of cycloocta-1,5-diene (COD) for 30 min at 30 Hz in a zirconia jar (10 mL) in air. The zirconia jar was preferred over the Eppendorf for sticky problems, which were solved by changing the milling container. Surprisingly, K_2_[PtCl_4_] was found to be completely unresponsive to coordination, while PtCl_2_ showed to be quite reactive. Starting from PtCl_2_, a second trial was carried out doubling the reaction time and a conversion of 79% was observed. Increasing the reaction time to 90 min showed visible degradation of the complex, changing from a whitish color to a gray-black color. Decreasing the milling frequency to 20 Hz for 60 or 90 min of milling did not lead to an improvement of the yield. The best methodology to synthesize **2** was achieved using PtCl_2_ and 1.2 equiv of COD for 60 min of milling at 30 Hz in a zirconia jar (Scheme 2). However, under these conditions purification was required by extraction with a small volume of CH_2_Cl_2_, filtration through cotton, removal of the solvent, and three final washes with pentane. The characterization data obtained agree with those reported in the literature.^60^

**Scheme 2:**
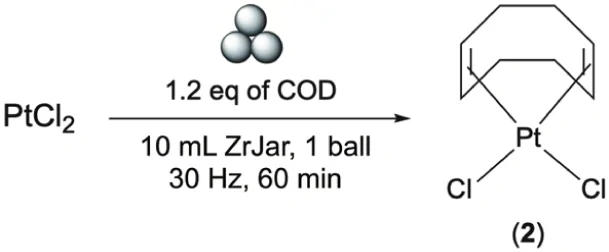
MC Synthesis of [Pt­(COD)­Cl_2_] (**2**)

### 3.3 Mechanochemical Synthesis of Platinum­(II) Complexes with N-Donor Ligands

Considering our previous work where PtCl_2_ was used to obtain the [Pt­(bipy)­Cl_2_] complex,^51^ we then extended the investigation by using other N-donor ligands. In this context, the first ligand employed was 1,10-Phenanthroline (phen). The mechanochemical synthesis reaction of the compound [Pt­(phen)­Cl_2_] (**3**) is shown in Scheme 3. As above-reported, only PtCl_2_ was found to be reactive, leading to the quantitative formation of **3** after 60 min of reaction in Eppendorf at 30 Hz. The milling was carried out under argon atmosphere to prevent possible side reactions. The NMR characterization data of **3** agree with those present in the literature.^61^

**Scheme 3:**
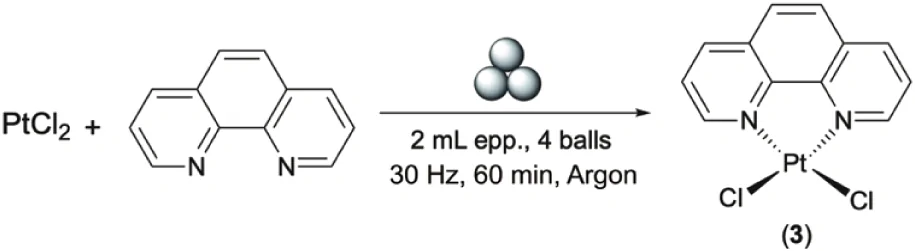
MC Synthesis of [Pt­(phen)­Cl_2_] (**3**)

As regards the complex of Pt­(II) with 2,2′;6′,2“-terpyridine (terpy), the mechanochemical approach leads to the formation of a species different from that typically obtained in solution. Based on our spectroscopic characterization, the product can be assigned as the [Pt­(κ^2^-terpy)­Cl_2_] (**4**) complex, in which the ligand coordinates in a bidentate and asymmetric fashion rather than adopting the expected κ^3^-coordination mode well-known and documented in literature.^62–65^

This assignment is supported by extensive NMR analysis. ^1^H NMR spectrum shows a significantly higher number of signals than expected for a symmetric [Pt­(κ^3^-terpy)­X]­X species,^66^ indicating loss of molecular symmetry. Consistently, the ^13^C­{^1^H} NMR spectrum displays 11 distinct signals for CH-type carbons (Figure 3), which is incompatible with a κ^3^-coordinated terpy ligand and instead suggests an asymmetric coordination environment. Other confirmation arises from two-dimensional NMR experiments. In particular, the ^1^H–^1^H COSY spectrum (see SI, Figure S13) reveals three distinct aromatic spin systems: two sets of three protons and one set of four protons, all inequivalent. This pattern is consistent with an asymmetrical κ^2^-coordination mode of the terpyridine ligand, leading to differentiation of all aromatic environments. Further confirmation of the asymmetrical κ^2^-coordination is given by ^195^Pt NMR spectrum, which shows a signal at −2953.68 ppm in DMSO-*d*_6_ at 298 K (see SI, Figures S17 and S18). In contrast to literature reports on solvothermal synthesis, where the [Pt­(κ^3^-terpy)­Cl]Cl complex is typically obtained and widely studied for luminescent applications, the mechanochemical product clearly deviates from this behavior. The **4** complex is obtained in quantitative yields from both precursors after 90 (Scheme 4), or 120 min of milling in an Eppendorf mill at 30 Hz.

**Figure 3:**
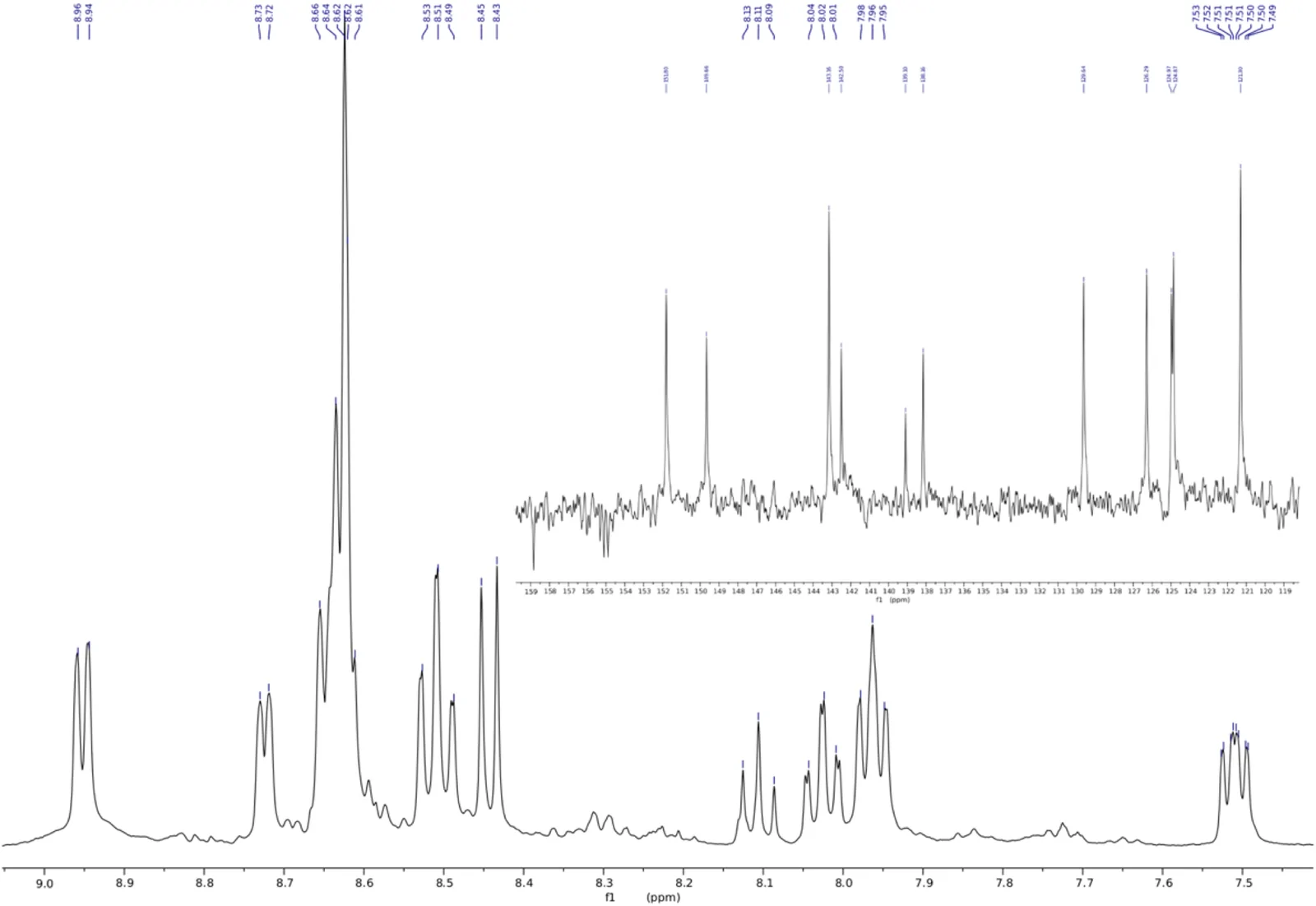
^1^H NMR (400.1 MHz, DMSO-*d*_6_, 298 K) and ^13^C­{^1^H} NMR spectra (100.6 MHz, DMSO-*d*_6_, 298 K) of [Pt­(κ^2^-terpy)­Cl_2_] (**4**).

**Scheme 4:**
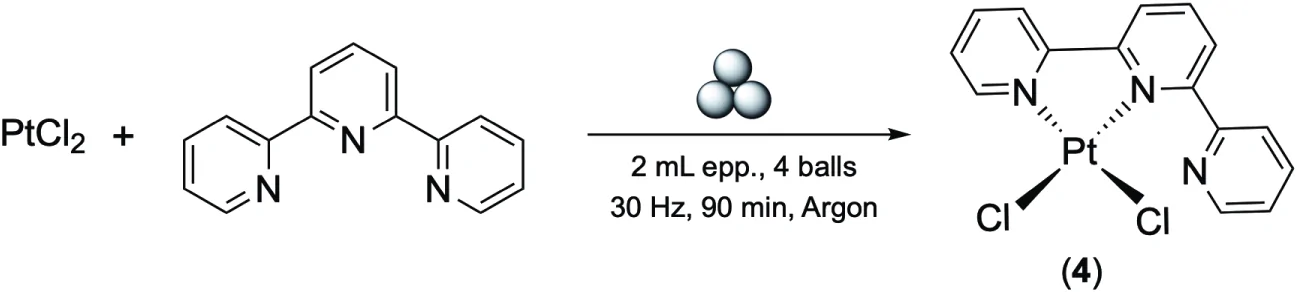
MC Synthesis of [Pt­(κ^2^-terpy)­Cl_2_] (**4**), Method A

The XRD pattern of sample **4**, obtained by ball milling, differs markedly from that of crystalline sample [Pt­(κ^3^-terpy)­Cl]Cl prepared in solution. When combined with NMR evidence for a different local coordination environment, these data demonstrate that ball milling does not simply reduce crystallinity but generates a new species.

Attempts to obtain the known [Pt­(κ^3^-terpy)­Cl]­Cl complex through mechanosynthesis in LAG (30 μL of solvent) with water, methanol and DMSO did not yield the desired product (Figure S68 in SI). Further testing was conducted on compound **4** by reacting it for 60, 90, and 120 min in LAG (50 μL of solvent) with water, methanol and DMSO, attempting to make it react by switching from κ^2^ to κ^3^-coordination, but without the expected results (Figure S69 in SI).

The formation of a κ^2^-coordinated species instead of the expected κ^3^-complex highlights the influence of solid-state conditions on coordination chemistry. In solution, κ^3^-coordination is favored by conformational flexibility, solvent-mediated rearrangements, and chloride ion dissociation. In the solid state, however, restricted molecular mobility and limited structural reorganization promote kinetically accessible coordination modes. Under these conditions, partial ligand coordination at locally activated metal centers is favored, while formation of the thermodynamically preferred κ^3^-geometry is hindered by steric constraints and the stability of Pt–Cl bonds. These findings demonstrate that mechanochemistry can significantly influence not only reactivity but also coordination modes, enabling access to species that are not readily obtainable under conventional solution-phase conditions.

As regards the mechanosynthesis of [Pt­(BIAN)­Cl_2_] (**5**), the reaction proceeds smoothly when PtCl_2_ is used as the precursor, with yields reaching almost 90% in 90 min of reaction. By further extending the reaction times, no increase in yield was observed. On the contrary, K_2_[PtCl_4_], after 3 h of milling, was not found to be active in the synthesis of **5**.^67^ In addition to the NMR spectra, further confirmation of the formation of the complex **5** is provided by the FTIR spectrum of the postmilling powder without any workup. The shift to lower wavenumbers of the IR signal corresponding to the v_C=N_ of the ligand between the coordinated and free ligand is visible, going from 1670 cm^–1^ in the free ligand (Figure S116 in SI) to 1601 cm^–1^ in the complex (Figure S104 in SI).

Similar attempts to obtain platinum derivatives bearing other potential N-donor ligands such as acetonitrile and benzonitrile were unsuccessful, despite numerous attempts and variations of the reaction conditions.

### 3.4 Mechanochemical Synthesis of Platinum­(II) Complexes with P-Donor Ligands

Our interest was then focused on the synthesis of Pt­(II) complexes with P-donor ligands. The first ligand investigated was triphenylphosphine (PPh_3_). The reaction between PtCl_2_ and PPh_3_ (1:2 molar ratio) was carried out in a ball mill for 15 min at 30 Hz, in Eppendorf previously charged with argon (Scheme 5).

**Scheme 5:**
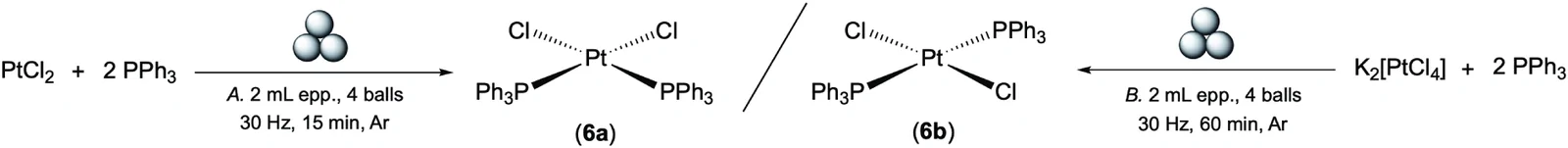
MC Synthesis of [Pt­(PPh_3_)_2_Cl_2_] (**6**)

As expected, the reaction leads to the formation of both complexes *cis*-[Pt­(PPh_3_)­Cl_2_] (**6a**) and *trans*-[Pt­(PPh_3_)­Cl_2_] (**6b**) with a greater selectivity for **6a** (Figure 4). The NMR characterization data agree with those present in the literature.^68^

**Figure 4:**
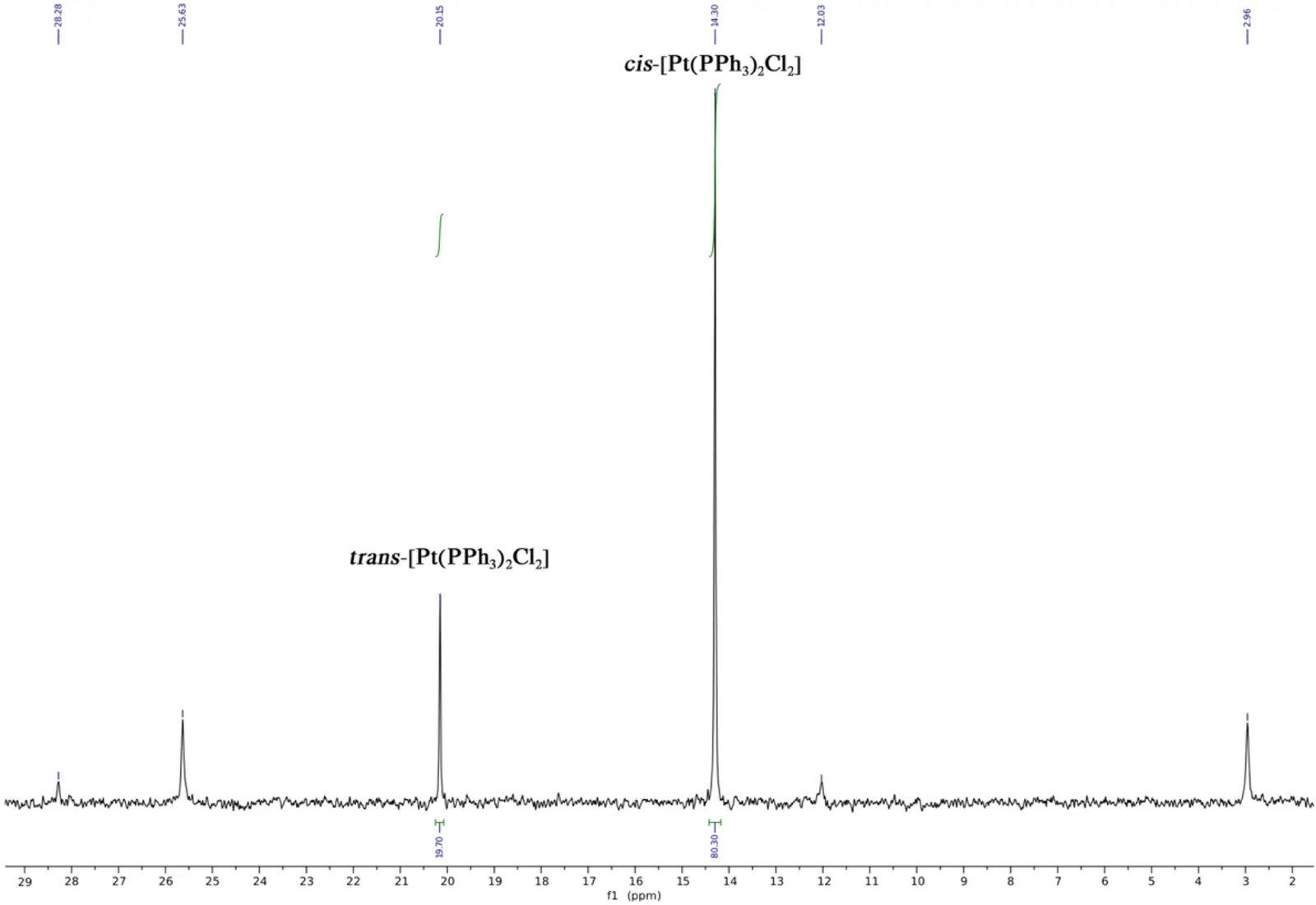
^31^P­{^1^H} NMR spectra (162.0 MHz, CDCl_3_, 298 K) of [Pt­(PPh_3_)_2_Cl_2_] (**6**).

The literature data confirm that the **6a** is the thermodynamically favored.^68^ This trend would therefore be expected to persist in the solid state despite the absence of solvent, further confirming the findings of Balema and coworkers,^53^ who, under more drastic conditions, obtained exclusively the *cis*-isomer in the solid state. In solution, stabilization is provided by an electrostatic interaction between the complex and the solvent, which is maximized in the *cis*-isomer, resulting in the latter being more stable. It is therefore reasonable to assume that in the mechanochemical approach, the crystal lattice of the ground solid stabilizes the *cis*-product, again by maximizing electrostatic interactions, behaving as a sort of solvent during the mill reaction.

As done for [Pt­(DMSO)_2_Cl_2_] (**1**), we carried out a test aimed at studying the conversion of both PtCl_2_ and K_2_[PtCl_4_] precursors into **6**. Using PtCl_2_, the reaction proceeds very quickly, showing an 82% conversion after just 5 min of milling and 81% selectivity for the **6a**. After 10 min, the conversion is complete, with 76% selectivity for the **6a**, that increases slightly to 78% after further 5 min. Then, the reaction proceeds with some decomposition of the compounds, as highlighted in Figure 5.

**Figure 5:**
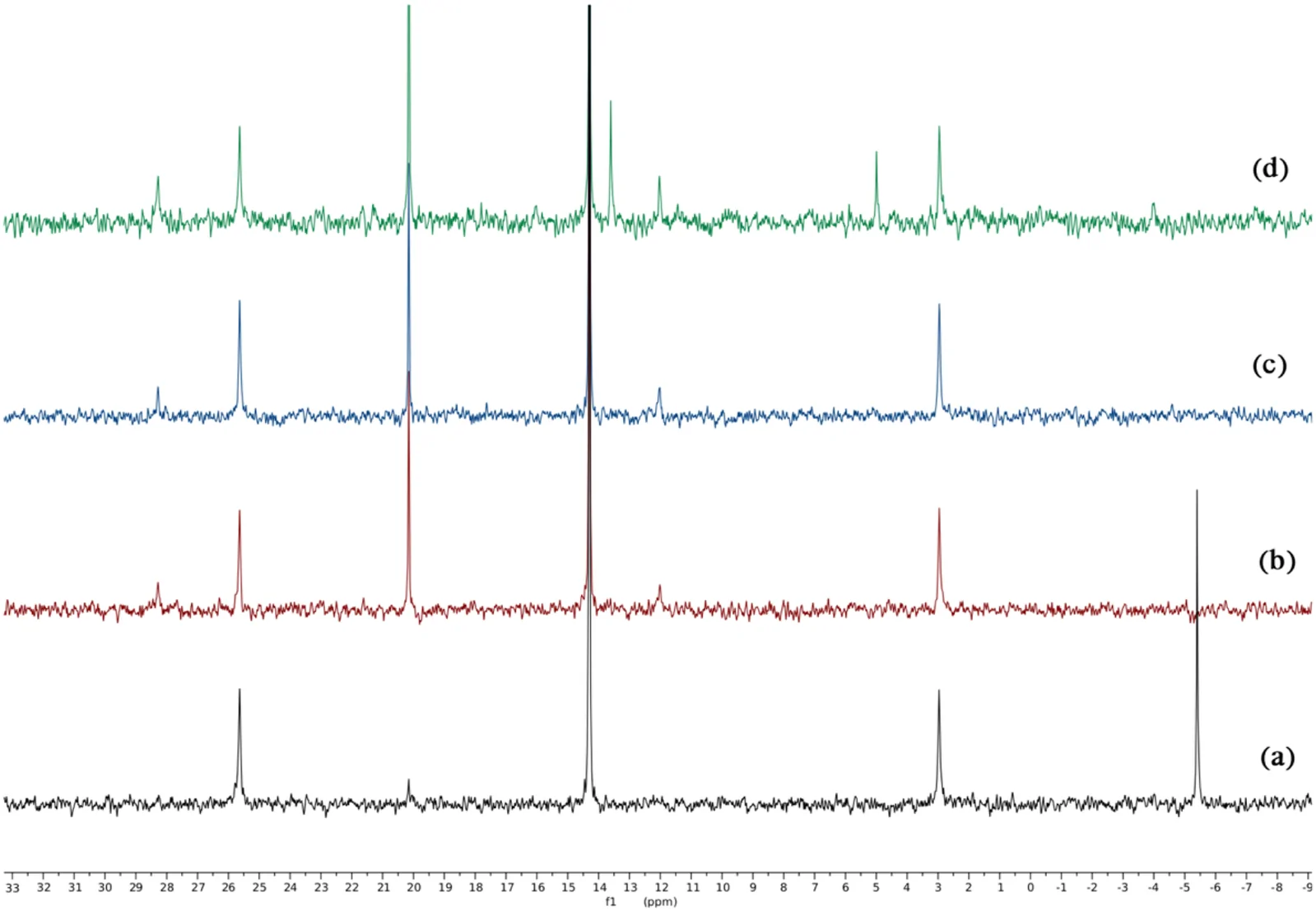
^31^P­{^1^H} NMR spectra (162.0 MHz, CDCl_3_, 298 K) of the mixture PtCl_2_/PPh_3_ (**6**) in 1:2 molar ratio milled for: (a) 5 min, (b) 10 min, (c) 15 min, and (d) for 30 min.

Given the abrupt reactivity between PtCl_2_ and PPh_3_ in the mill, to verify that the reaction does occur indeed mechanochemically, the precursor and 2 equiv of ligand were placed into an NMR tube containing CDCl_3_. After the insertion of the reagents, the content of the tube was monitored after 5 min, revealing a conversion of only 30% of the reagents into **6**. Despite the initial high reactivity in the NMR tube, the reaction than almost stops, reaching only 40% conversion after 1 day (see SI, Figure S86). In this test, the only Pt-containing species observed was the *cis*-isomer.

As observed for **1**, the precursor K_2_[PtCl_4_] was found to be less reactive. In fact, at 5 min of milling the conversion was 4%, then increasing steadily up to a maximum of 90% at 90 min of reaction (Scheme 5). The selectivity of the *cis*- and *trans*-products is in line with the previous test, even if at longer reaction times it is slightly higher. It is important to note that with this precursor, having to use longer reaction times, the formation of PPh_3_O was observed. The data obtained are reported in Figure 6.

**Figure 6:**
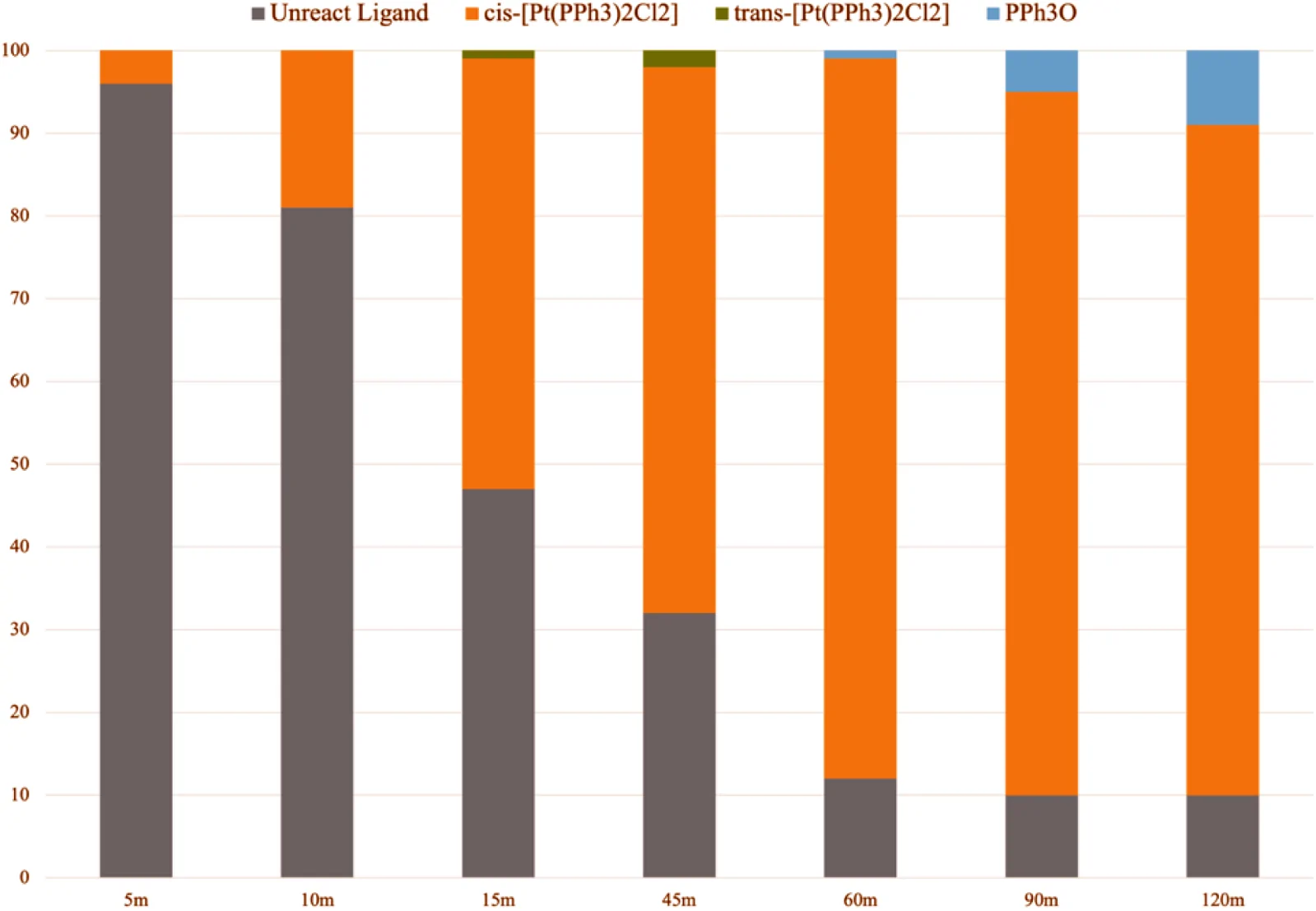
Conversion test of K_2_[PtCl_4_]/PPh_3_ 1:2 mixture into [Pt­(PPh_3_)_2_Cl_2_] (**6**).

Another monodentate P-donor ligand, tris­[3,5-bis­(trifluoromethyl)­phenyl]­phosphine (P­(3,5-(CF_3_)_2_Ph)_3_), was investigated in the reaction with PtCl_2_. This ligand proved to be much less reactive than PPh_3_, leading to complex *trans*-[Pt­(P­(3,5-(CF_3_)_2_Ph)_3_)_2_Cl_2_] (**7**). A single signal at 22.51 ppm, accompanied by satellite peaks of Pt, is present in the ^31^P­{^1^H} NMR spectrum, thus confirming the exclusive formation of the *trans*-isomer.^69^ The best yield of 91% was obtained after 90 min in Eppendorf at 30 Hz under an argon atmosphere. Low yield was observed when K_2_[PtCl_4_] was used as starting material.

Several bidentate P-donor ligands were then investigated, the first of them being (9,9-Dimethyl-9H-xanthene-4,5-diyl)­bis­(diphenylphosphane) (Xantphos). The mechanochemical synthesis of complex [Pt­(Xantphos)­Cl_2_] (**8**) starting from PtCl_2_ in Eppendorf was successful, as the conversion was quantitative after 20 min. The characterization data are consistent with those reported in the literature.^70^ Again, K_2_[PtCl_4_] showed much lower reactivity as, after 2 h of milling in Eppendorf the conversion was only 5%. When the reaction was performed in a 25 mL steel jar, a better result was achieved, however the conversion of the reagents into **8** was only 57% after 2 h.

Both PtCl_2_ and K_2_[PtCl_4_] were used to investigate their reactivity with the diphosphine ligands dppm, dppe, dppp, dppb, dppbz, dppf, dippf, and (*R*)-BINAP, the synthetic route is shown in Scheme 6. A Pt/ligand 1:1 molar ratio was used, and the Eppendorf was charged with argon to prevent ligand oxidation. The complete conversion of PtCl_2_ into the corresponding [Pt­(P∩P)­Cl_2_] complex (**9**–**16**) was observed for all ligands. The same was found starting from K_2_[PtCl_4_], except for complexes [Pt­(dppbz)­Cl_2_] (**13**) and [Pt­((*R*)-BINAP)­Cl_2_] (**16**). In fact, the formation of complex **13** stopped at 30% conversion after 2 h (see Figure S90 in SI), while in the case of the ligand (*R*)-BINAP a quantitative conversion was observed, but complex **16** was formed in only 7% yield. A product identified as [1,1′-binaphthalene]-2,2′-diylbis­(diphenylphosphine oxide) was observed at 28.42 ppm in ^31^P­{^1^H} NMR spectrum, lacking the platinum satellite peaks (see Figure S91 in SI).^71^ The whole results are collected in Table 1. Stability tests of the phosphine complexes to prolonged milling were performed, showing that the compounds, once formed, are stable even after milling for 2 h.

**Scheme 6:**
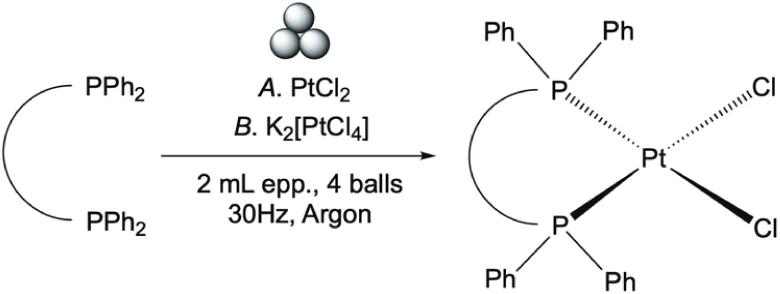
MC Formation of [Pt­(P∩P)­Cl_2_] (**9**–**16**) Complexes

**Table 1:** Complexes Obtained by MC Route with Diphosphine Ligands

Ligand	Yield (%)	
-	PtCl_2_	K_2_[PtCl_4_]
dppm (**9**)	>99%	>99%
dppe (**10**)	>99%	>99%
dppp (**11**)	>99%	>99%
dppb (**12**)	>99%	>99%
dppbz (**13**)	>99%	30%
dppf (**14**)	>99%	>99%
dippf (**15**)	>99%	>99%
(*R*)-BINAP (**16**)	>99%	7%

To confirm that the reactions under examination are quantitative and that there is no longer the presence of the metal precursor PtCl_2_, a test was performed with a ligand excess of 1.2 equiv. The first ligand studied was dppm, but the reaction did not show the excess as expected. The ^31^P­{^1^H} NMR spectra showed the signal relating to species **9**, but at −37.15 ppm the signal of a new Pt­(II) species correlated with the characteristic satellite peaks was noted (Figure S92 in SI). From a comparison in the literature, this species was identified as [Pt­(dppm)_2_]­Cl_2_.^72^ Given this result, to verify the quantitative yield of the reaction, dppb was used as the ligand, given its greater steric hindrance. The test was then conducted with an excess of 1.2 equiv of dppb with respect to PtCl_2_ precursor, in an Eppendorf at 30 Hz for 30 min, to ensure a complete reaction. The ^31^P­{^1^H} NMR spectra showed the signal of **12** as predominant, but also two additional signals at −16.09 ppm, characteristic of the free ligand, and at 31.99 ppm, the position of dppbO_2_.^73^ Despite that, these last two signals represent exactly the excess equiv. introduced at the beginning of the reaction, thus demonstrating that the reaction is overall quantitative (Figure S93 in SI).

### 3.5 X-ray Powder Diffraction Analysis (XRPD)

The XRPD patterns of the platinum complexes obtained in solution and by mechanochemical ball milling reveal significant differences in terms of crystallinity, long-range order and, in some cases, the formation of distinct solid-state phases. In general, the samples prepared in solution display sharper, more intense and better-resolved reflections, consistent with more crystalline materials. In contrast, the samples obtained by ball milling, except for [Pt­(dppb)­Cl_2_], show pronounced peak broadening, a decrease in the signal-to-background ratio and, in some cases, the appearance of diffuse scattering contributions. These features are consistent with a reduction in crystalline domain size, an increase in microstrain and, or partial amorphization.

For [Pt­(PPh_3_)_2_Cl_2_] (**6**) obtained from solution, the XRD pattern displays several intense reflections, particularly in the low-angle region, suggesting the formation of a well-defined crystalline phase. The corresponding ball-milled sample retains some features of the pattern observed for the solution one, but the peaks are significantly broader and less resolved. This indicates that the mechanical treatment does not necessarily lead to a complete loss of structural order but induces a substantial decrease in crystallinity and an increase in solid-state disorder. The pattern of MC product can therefore be interpreted as arising from a nanocrystalline or partially amorphized material in which a structural component related to the solution-derived phase is still retained (Figure S117 in SI).

Samples corresponding to [Pt­(dppm)­Cl_2_] (**9**) prepared in solution, exhibit very similar XRPD profiles (Figure S118 in SI). The good overlap between the two patterns confirms the reproducibility of the solution synthesis and suggests the formation of the same crystalline phase. The intense and relatively sharp reflections, mainly observed in the 9–22° 2θ range, indicate a high degree of long-range order. Nevertheless, a careful inspection of the low-angle region (5–15° 2θ) reveals a distinct broad background hump underneath the sharp Bragg peaks. This feature clearly indicates a semicrystalline nature, confirming the coexistence of a residual amorphous or disordered phase alongside the predominant crystalline framework. This is a common outcome of rapid solution crystallization, where a fraction of the molecules fails to organize into the long-range lattice before precipitating. In contrast, the sample obtained by ball milling, shows a drastically different profile, with strong attenuation of the main reflections and a more pronounced diffuse background. The profile becomes fully dominated by the broad amorphous halo, which matches the position of the residual amorphous hump observed in the solution samples. This result indicates that, for **9**, the mechanochemical route promotes a marked loss of crystallinity. Although some residual reflections may suggest the presence of a minor crystalline fraction, the pattern is dominated by a much less ordered component compared with the solution-derived samples.

A different behavior is observed for [Pt­(dppb)­Cl_2_] (**12**). The PXRD profiles of **12** prepared via different synthetic routes are compared in Figure S119. All three samples exhibit the same set of characteristic diffraction peaks, confirming that they share the same crystalline phase and polymorphic form. However, a significant difference in the degree of crystallinity and background features can be observed depending on the synthetic pathway employed. The samples prepared via solution-based methods (red and blue curves) display a prominent amorphous halo in the 10–25 2θ region. In stark contrast, the sample obtained through mechanochemical synthesis (green curve) demonstrates a markedly superior crystallinity. The diffraction peaks are significantly sharper and more intense compared to the solution-derived counterparts. Mechanochemistry can promotes quantitative conversion and high crystal density through continuous mechanical shearing and recrystallization processes. The reduced amorphous background and enhanced peak definition clearly indicate that the mechanochemical route yields a more highly ordered and crystalline **12** framework compared to traditional solution-based methodologies. This striking divergence between the two systems suggests that the shorter methylene spacer of the dppm ligand (−CH_2_−), compared to the longer and more adaptable butyl chain of dppb (−CH_2_−)_4_, drastically alters the mechanical stability and packing energy of the crystalline network.

The most pronounced difference is observed for the terpyridine-containing system. [Pt­(κ^3^-terpy)­Cl]Cl obtained from solution, shows the most crystalline profile of the whole series, with numerous sharp and very intense reflections. The presence of particularly dominant peaks may be associated with a high degree of crystallinity. In sharp contrast, the milling product, formulated as [Pt­(κ^2^-terpy)­Cl_2_] (**4**), displays a completely different pattern, characterized by the disappearance of most of the intense reflections observed for [Pt­(κ^3^-terpy)­Cl]Cl and by the appearance of a much weaker and more diffuse profile. This behavior cannot be described simply as a reduction in peak intensity, but rather indicates a substantial structural transformation induced by milling. The combination of XRPD data with NMR analysis further supports this interpretation. While XRPD highlights the loss of long-range order and the formation of a poorly crystalline or amorphous solid, NMR confirms the presence of a different local environment compared with the solution-derived sample. Therefore, for the comparison between the two terpyridine system (Figure S120 in SI), it can be concluded that ball milling does not simply produce a less crystalline form of the same phase but induces the formation of a distinct solid-state phase. This phase appears to be characterized by a different short-range structural organization, but by a strongly reduced long-range order, as evidenced by the absence of a well-resolved XRPD pattern.

Overall, these results indicate that the synthetic route plays a decisive role in determining the solid-state structure of the Pt­(II) complexes investigated. Crystallization from solution generally favors the formation of ordered and reproducible phases, that nevertheless retain a partial amorphous fraction. In contrast, the impact of mechanochemical ball milling is highly divergent and depends strictly on the nature of the ligand. In the phosphine-based systems, ball milling can either significantly enhance long-range order, as seen in the highly crystalline **12**, or induce total amorphization, as observed for the more rigid **9** due to its lower capacity to absorb mechanical stress. Meanwhile, in the terpyridine complex the combined XRPD and NMR evidence indicates the formation of a clearly distinct solid-state phase. These findings confirm that ball milling is not merely an alternative synthetic methodology but can also act as a powerful tool to access metastable or structurally distinct solid forms that are not easily accessible by crystallization from solution.

To further assess the crystalline phases obtained by solution and mechanochemical synthesis, the experimental PXRD patterns were compared with powder diffraction patterns calculated from the crystallographic information files (CIFs) of the corresponding crystal structures retrieved from the Cambridge Structural Database (CSD). The simulated PXRD patterns were generated using the Mercury software (CCDC) and are reported in the Supporting Information together with the experimental diffractograms.

For complex **6**, calculated PXRD patterns for both *cis*- and *trans*-[Pt­(PPh_3_)_2_Cl_2_] were considered (Figure S121 in SI). Although the experimental diffractograms exhibit broad reflections owing to the limited crystallinity of the samples, the principal diffraction maxima show a satisfactory qualitative agreement with the calculated patterns. In particular, the position of the most intense experimental reflection closely matches that of the strongest reflection calculated for the *cis*-isomer, consistent with the 80% *cis*-selectivity independently determined by NMR spectroscopy. Nevertheless, the significant peak broadening prevents an unambiguous distinction between the *cis*- and *trans*-isomers based solely on PXRD data. A similar comparison was carried out for complexes **9** (Figure S122 in SI). The experimental patterns are qualitatively consistent with the calculated PXRD profile of *cis*-[Pt­(dppm)­Cl_2_], particularly in the low-angle region. Additional experimental reflections and differences in relative intensities are observed, which can reasonably be attributed to the limited crystallinity of the samples and/or preferred orientation effects. Therefore, the calculated PXRD patterns were used as qualitative references for phase identification rather than as evidence of complete structural identity.

### 3.6 Green Metrics: Mechano- vs Solution-Synthesis

A comparison of green metrics between the present mechanochemical syntheses and standard solvothermal methods is depicted in Table 2.

**Table 2:** Green Metrics Comparison between Mechano- and Solution-Synthesis

	Mechanochemical Synthesis^a^			Solution Synthesis				
Complex	E-factor	EMY	Time [min]	E-factor	EMY	Time [min]	Temp [°C]	Ref
[Pt(DMSO)_2_Cl_2_] (**1**)	≃0	≃100	30	54.9	1.74	90	<100, r.t.	^19^
[Pt(COD)Cl_2_] (**2**)	115	0.86	60	161	0.62	120	60, 0, r.t.	^18^
[Pt(phen)Cl_2_] (**3**)	≃0	≃100	60	402	0.25	120	100	^74^
[Pt(κ^2^-terpy)Cl_2_] (**4**)	≃0	≃100	90	-	-	-	-	-
[Pt(BIAN)Cl_2_] (**5**)	106	0.94	90	214	0.47	1200	<80	^67^
[Pt(PPh_3_)_2_Cl_2_] (**6**)	≃0	≃100	15	16.19	5.68	360	<140	^68^
[Pt(P(3,5-(CF_3_)_2_Ph)_3_)_2_Cl_2_] (**7**)	0.1	91	90	342	0.29	1500	r.t.	^69^
[Pt(Xantphos)Cl_2_] (**8**)	≃0	≃100	20	68.64	1.43	540	r.t.	^75^
[Pt(dppm)Cl_2_] (**9**)	≃0	≃100	10	14.12	6.56	60	r.t.	^76^
[Pt(dppe)Cl_2_] (**10**)	≃0	≃100	15	14.04	6.60	60	r.t.	^76^
[Pt(dppp)Cl_2_] (**11**)	≃0	≃100	30	13.72	6.73	60	r.t.	^76^
[Pt(dppb)Cl_2_] (**12**)	≃0	≃100	10	13.42	6.89	60	r.t.	^76^
[Pt(dppbz)Cl_2_] (**13**)	≃0	≃100	10	409	0.24	540	r.t.	^77^
[Pt(dppf)Cl_2_] (**14**)	≃ 0	≃100	15	44.89	2.18	1200	<80	^78^
[Pt(dippf)Cl_2_] (**15**)	≃0	≃100	20	1220	0.08	90	r.t.	^79^
[Pt((*R*)-BINAP)Cl_2_] (**16**)	≃0	≃100	15	83.88	1.17	120	r.t.	^80^

a E-factor and EMY parameters have been calculated for the mechanochemical reactions given by PtCl_2_.

The formation of Pt­(II) complexes **1**–**16** was efficiently achieved, with reaction times spanning from 10 up to 90 min but, in most cases, 30 min were enough to achieve a quantitative yield. When compared to traditional solution-phase methodologies, the mechanochemical strategy proves considerably more straightforward, as it obviates the necessity for intermediate operations such as precipitation, solvent-based washing, and subsequent drying.

The exclusion of bulk solvents from the synthetic protocol constitutes a fundamental advancement toward more sustainable chemistry, primarily due to the significant reduction in chemical waste generation. In contrast to conventional solution-based reactions, which often rely on stoichiometric excesses to drive reactions to completion, the mechanochemical approach typically enables the use of equimolar quantities of reactants. This behavior can be rationalized by the increased reactivity and intimate mixing induced by mechanical grinding, which generally facilitates near-quantitative consumption of starting materials. As a result, reagent excess is minimized, and postsynthetic purification procedures are markedly simplified.

A comparative evaluation between mechanochemical routes and analogous solution-based procedures was performed employing two widely recognized green metrics: the E-factor and the Effective Mass Yield (EMY).^81^ These parameters provide valuable insight into the environmental footprint of the synthetic process. It should be emphasized, however, that numerous literature-reported solution-phase protocols lack the comprehensive experimental details required for a rigorous and fully reliable determination of such metrics. Consequently, the calculated values for these procedures may not always accurately represent their true environmental impact. The plot shown in Figure 7 presents the EMYs and Reaction Times values for a representative series of platinum­(II) complexes discussed herein, highlighting the superior performance of mechanochemical methods, where applicable, in terms of both sustainability and synthetic efficiency. The selected compounds correspond to systems frequently encountered in the literature, often employed as precursors or catalytic species.

**Figure 7:**
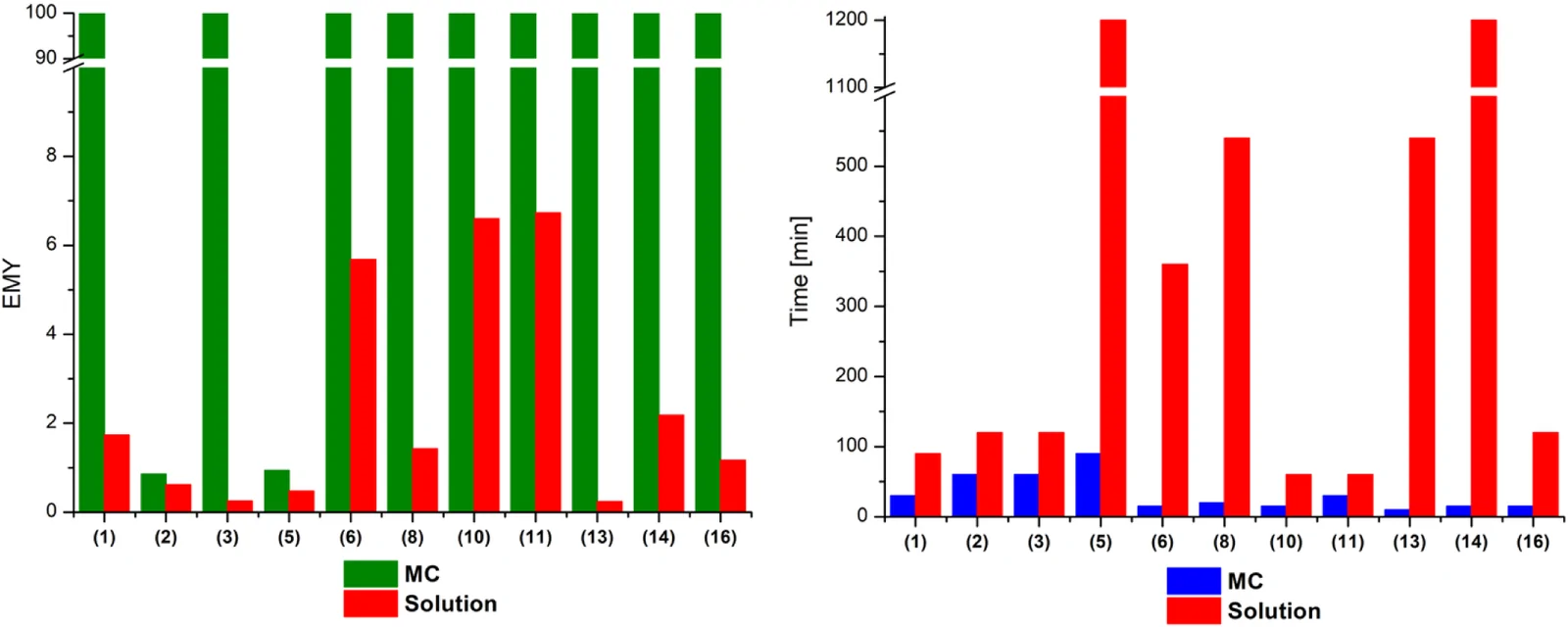
Comparison of EMYs and reaction times in mechanochemistry and solution.

Overall, the results obtained in this study indicate that, for most of the investigated platinum­(II) complexes, mechanochemical synthesis affords more favorable green metrics values. In contrast, the corresponding solution-based methods reported in the literature tend to exhibit higher green metrics values. These observations underscore the improved sustainability profile of mechanochemical approaches relative to conventional solution-phase techniques. Nevertheless, it must be emphasized that mechanochemistry does not universally supplant solvothermal or solution-based methodologies for all systems; rather, it should be regarded as a complementary synthetic strategy that integrates efficiency with reduced environmental impact. Beyond ecological considerations, mechanochemistry also offers tangible practical benefits, including streamlined synthetic procedures. Indeed, whereas solution-based protocols frequently suffer from incomplete yields due to solvent handling and purification losses, the mechanochemical conditions described herein often enable quantitative conversions within relatively short milling times. Although not universally applicable, this methodology has proven to be highly effective across a broad spectrum of platinum­(II) complexes.

## 4 Conclusions

This study demonstrates that mechanochemistry is an effective and versatile strategy for synthesizing a wide range of platinum­(II) complexes. Solvent-free ball milling enables rapid, high-yielding, and operationally simple syntheses, often achieving quantitative conversions in substantially shorter times than conventional solution-based methods. Under mechanochemical conditions, PtCl_2_ consistently outperforms K_2_[PtCl_4_], providing faster, simpler, more cost-effective, and more sustainable protocols. A key finding is the inversion of precursor reactivity compared to solution chemistry, highlighting the importance of solid-state effects in mechanochemical activation. Mechanical force generates reactive sites and promotes coordination independently of solubility, leading to distinct reactivity patterns. Mechanochemistry also enabled access to unusual coordination modes, exemplified by the formation of a κ^2^-terpyridine complex instead of the typical κ^3^-species, underscoring the influence of solid-state constraints on coordination geometry.

XRPD results demonstrate that while solution synthesis yields reproducible phases with a residual amorphous fraction, the impact of mechanochemistry is highly divergent. Mechanochemical processing acts as a tunable structural tool that, depending on ligand flexibility, can drive the Pt­(II) complexes toward enhanced crystallinity, total amorphization, or novel solid-state phases.

From a sustainability perspective, mechanochemistry outperforms traditional approaches through improved green metrics, reduced solvent use, simplified workup, and near-stoichiometric operating conditions. Preliminary investigations were carried out to evaluate the application of platinum­(II) complexes in mechanocatalysis. Although the results remain exploratory and the reaction conditions have not yet been optimized, oxidation of diphenylmethanol to benzophenone was achieved using Oxone as the oxidant and Na_3_PO_4_ as the base, with conversions reaching up to 59%. The highest catalytic activity was observed for complexes bearing the most sterically demanding ligands (e.g., complexes **5** and **8**). This behavior may be attributed to the steric bulk of the ligands, which likely contributes to enhanced stability of the complexes by protecting them from decomposition under mechanochemical conditions.

## Supplementary Material



## Data Availability

The data supporting this article are included as part of the Supporting Information (SI). Supporting Information is available. Any further data are available from the corresponding author upon request.

## Notes

Experimental details, green metrics calculations and characterization data for all compounds (^1^H NMR, ^13^C­{^1^H} NMR, ^19^F­{^1^H} NMR, ^31^P­{^1^H} NMR, ^195^Pt NMR, FTIR-ATR, XRPD) (PDF)
