# Supplementary material for: Mechanochemical Sustainable Synthesis of Platinum(II) Complexes: Reactivity Inversion and Coordination Mode Control
Source: Inorg Chem. 2026 Aug 5;65(32):18648–63. doi: 10.1021/acs.inorgchem.6c02094 (PMC13488397; doi:10.1021/acs.inorgchem.6c02094)
Supplement: Supplementary file 1 [file ic6c02094_si_001.pdf]

SUPPORTING INFORMATION

Mechanochemical Sustainable Synthesis of Platinum(II) Complexes: Reactivity Inversion and Coordination Mode Control

Leonardo Genesin[a], Talha Munir[a], Eleonora Aneggi[a], and Daniele Zuccaccia*[a]

[a] Dipartimento di Scienze Agroalimentari, Ambientali e Animali, Sezione di Chimica, Università di Udine, Via Cotonificio 108, I-33100 Udine, Italy

*Corresponding Author

E-mail: daniele.zuccaccia@uniud.it (D.Zuccaccia)

Via Cotonificio 108, I-33100 Udine, Italy

Contents

Part 1: General Procedure used for Mechanochemical synthesis.

Part 2: Details of Trials.

Part 3: Calculations for E-factor and Effective Mass Yield (EMY).

Part 4: NMR's characterizations spectra's.

Part 5: NMR's tests spectra's.

Part 6: FTIR spectra's.

Part 7: XRPD analysis.

Part 8: Other pictures.

Part 1. General procedure for Mechanochemical Synthesis

Mechanochemical synthesis of all the products has been carried out with the same approach by means of a vibrational mill (Figure S1). For all, relevant ratios of the metallic precursors are used and milled at required frequencies, depending upon their interaction. The exact milling frequency and time of milling with the resultant output has been highlighted in the table for each product. For most of the synthesis, 2 mL Eppendorf with 4 zirconia balls inside were used (3 mm diameter, ~0.4 g) (Figure S2). To avoid oxidation to occur during milling, Eppendorf was closed in presence of argon and sealed with parafilm. However, in some cases, 10 mL zirconia jar with one ball (10 mm diameter, ~3.3 g) (Figure S3) and 25 mL stainless steel jars with one ball (15 mm diameter, ~13.4 g) (Figure S4) were used.

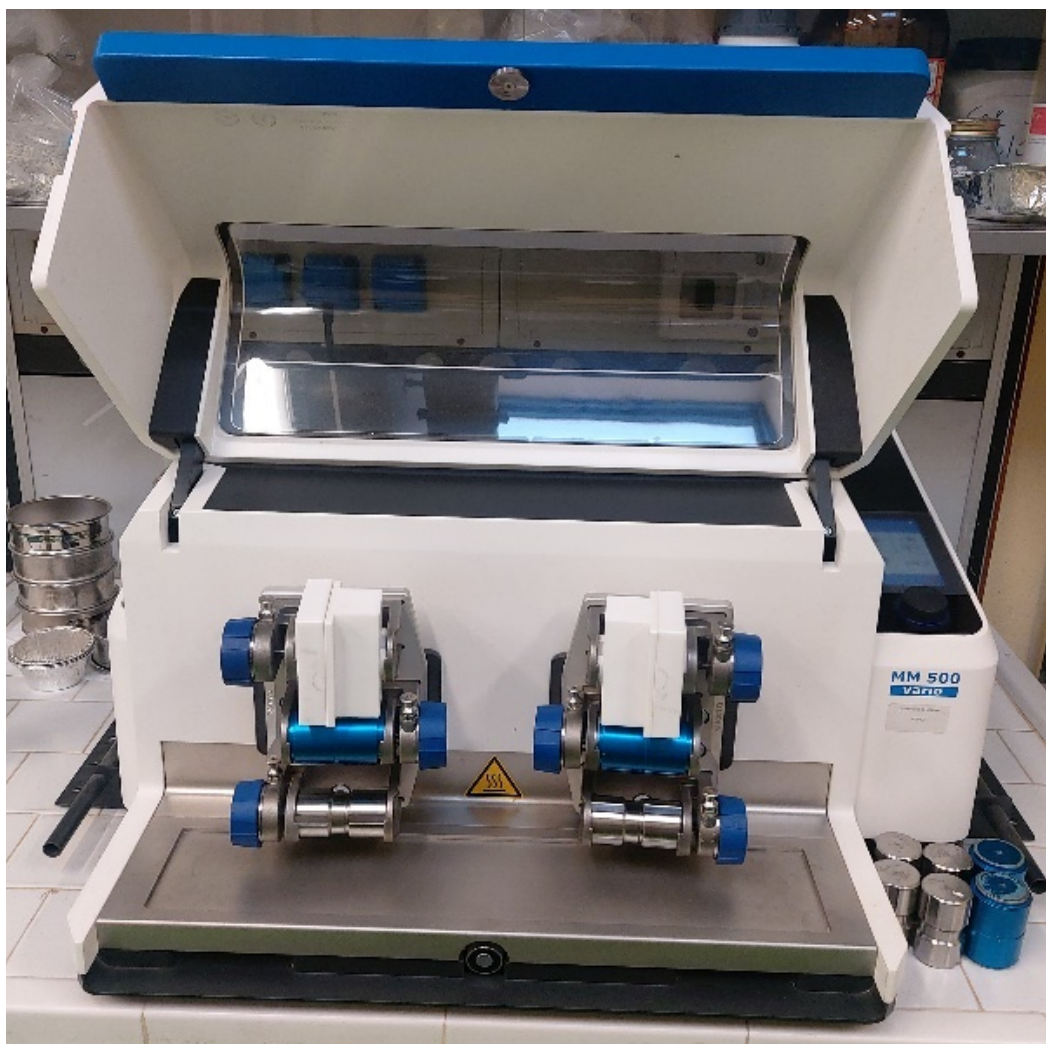

Figure S1. Retsch Ball Mill (MM 500 Vario).

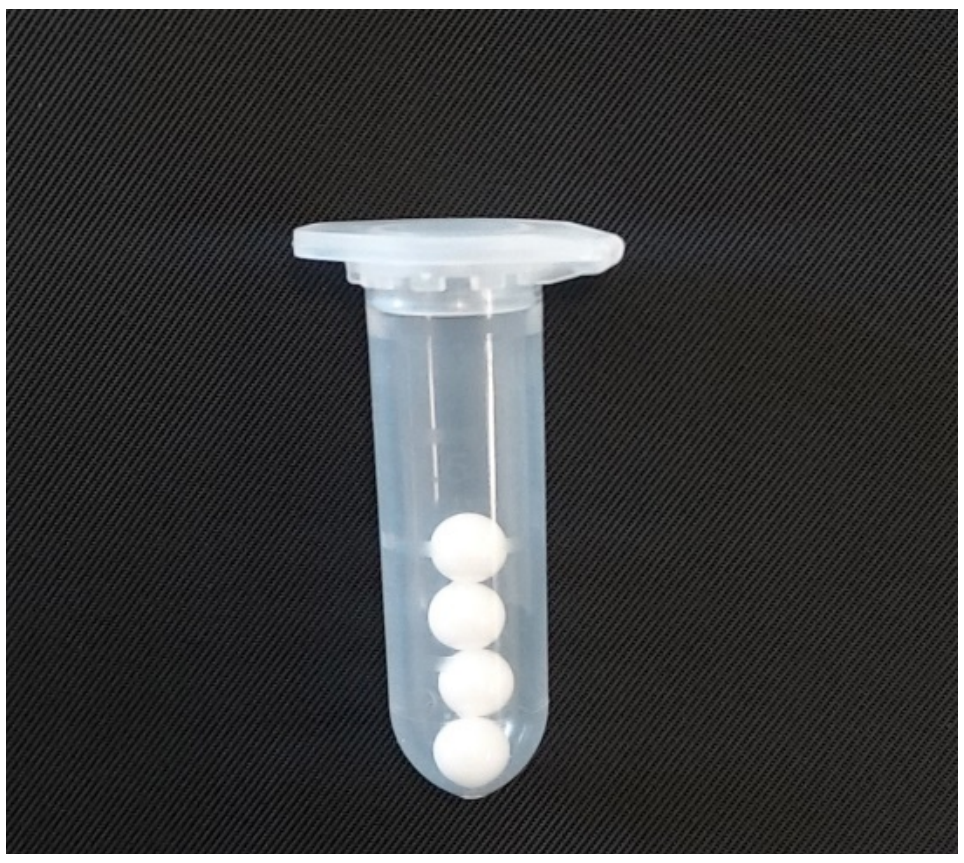

Figure S2. Eppendorf with 4 balls.

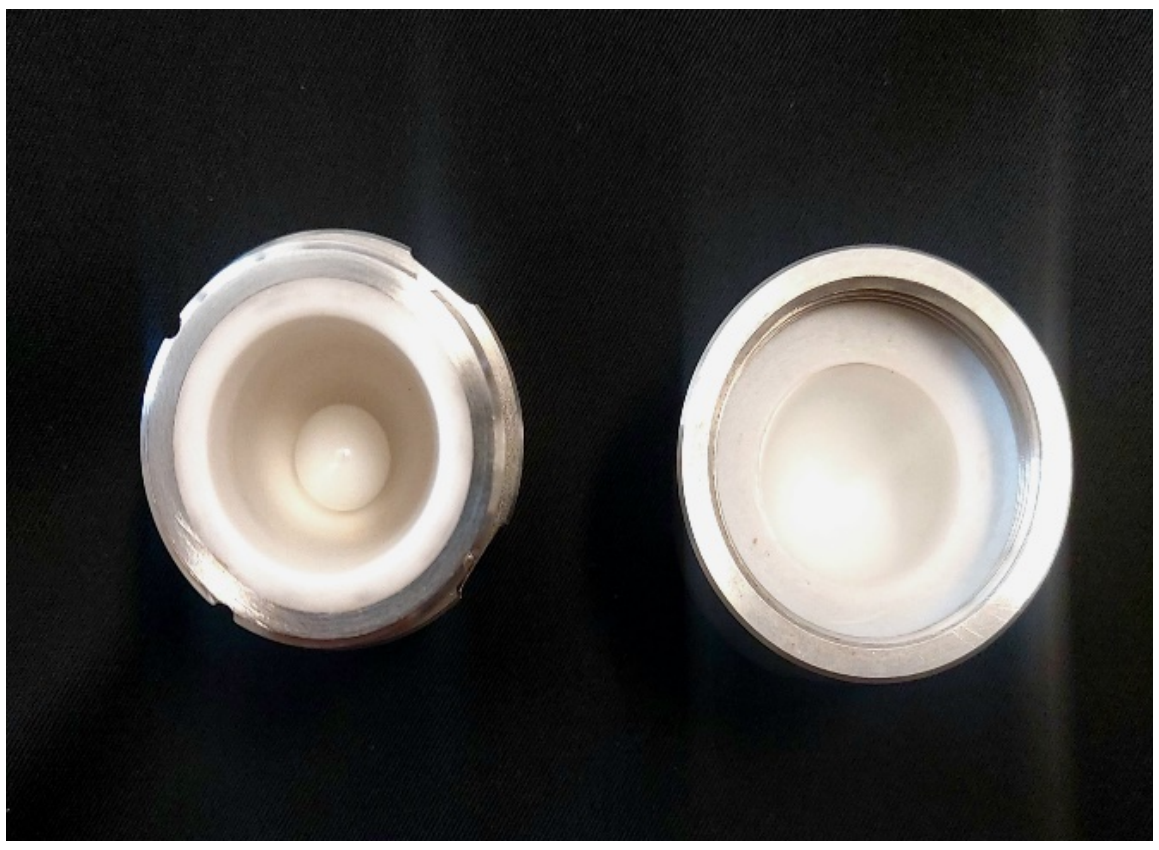

Figure S3. 10 mL zirconia jar with one ball.

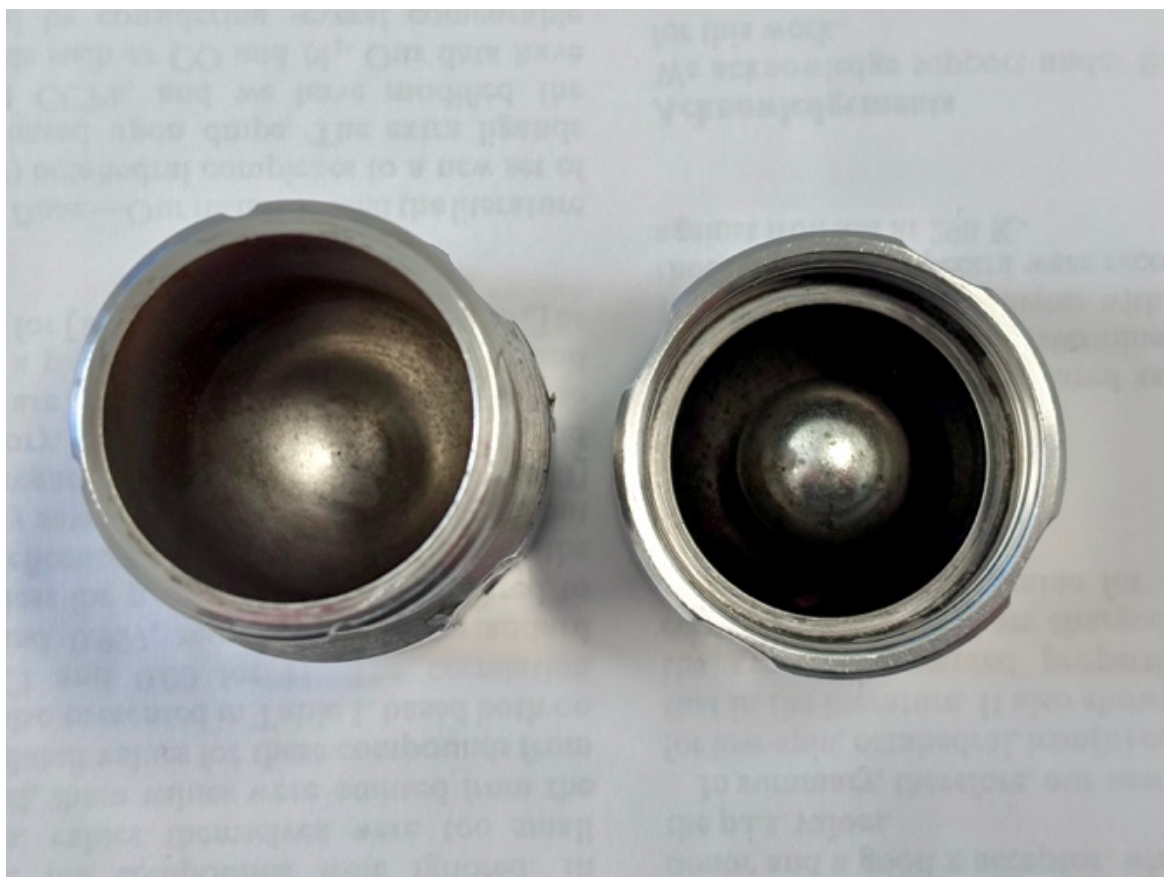

Figure S4. 25 mL stainless steel jar with one ball.

MC oxidation of diphenylmethanol

Method A, diphenylmethanol (25.0 mg, 0.14 mmol), Pt(II) complex (0.014 mmol, 10% in mol), Na_3PO_4 (150 mg) and Oxone® (250 mg) were milled at 30 Hz for 90 min in 25 mL stainless steel jar with one stainless steel ball (15 mm). Yield: [Pt(κ^2-terpy)Cl_2] (**4**) 35% (Fig. S94); [Pt(BIAN)Cl_2] (**5**) 33% (Fig. S95); [Pt(Xantphos)Cl_2] (**8**) 32% (Fig. S96).

Method B, Diphenylmethanol (25.0 mg, 0.14 mmol), $PtCl_2$ (3.7 mg, 0.014 mmol), Complex Ligand (0.014 mmol), Na_3PO_4 (150 mg) and Oxone® (250 mg) were milled at 30 Hz for 90 min in 25 mL stainless steel jar with one stainless steel ball (15 mm). Yield: [Pt(κ^2-terpy)Cl_2] (**4**) 34% (Fig. S97); [Pt(BIAN)Cl_2] (**5**) 59% (Fig. S98); [Pt(Xantphos)Cl_2] (**8**) 31% (Fig. S99).

Part 2. Details of Trails

[Pt(DMSO)$_2$Cl$_2$] (1)					
**Number of trails**	**Time (min)**	**Frequency (Hz)**	**Ratio (M:L)**	**Washing**	**Comments**
**T1** (Epp.)	5	30	(PtCl$_2$) 1:2	No	χ=99%, S_{cis}=90%, S_{trans}=9%
**T2** (Epp.)	10	30	(PtCl$_2$) 1:2	No	χ>99%, S_{cis}=91%, S_{trans}=9%
**T3** (Epp.)	30	30	(PtCl$_2$) 1:2	No	χ>99%, S_{cis}=95%, S_{trans}=5%
**T4** (Epp.)	45	30	(PtCl$_2$) 1:2	No	χ>99%, S_{cis}=93%, S_{trans}=6%, O-donor species=1%
**T5** (Epp.)	60	30	(PtCl$_2$) 1:2	No	χ>99%, S_{cis}=85%, S_{trans}=11%, O-donor species=4%
**T6** (Epp.)	90	30	(PtCl$_2$) 1:2	No	χ>99%, S_{cis}=81%, S_{trans}=10%, O-donor species=9%
**T7** (Epp.)	5	30	(K$_2$[PtCl$_4$]) 1:2	Yes, 2 times water.	χ=51%, S_{cis}=43%, S_{trans}=8%
**T8** (Epp.)	10	30	(K$_2$[PtCl$_4$]) 1:2	Yes, 2 times water.	χ=66%, S_{cis}=49%, S_{trans}=17%
**T9** (Epp.)	15	30	(K$_2$[PtCl$_4$]) 1:2	Yes, 2 times water.	χ=73%, S_{cis}=51%, S_{trans}=22%
**T10** (Epp.)	45	30	(K$_2$[PtCl$_4$]) 1:2	Yes, 2 times water.	χ=81%, S_{cis}=68%, S_{trans}=13%
**T11** (Epp.)	60	30	(K$_2$[PtCl$_4$]) 1:2	Yes, 2 times water.	χ=90%, S_{cis}=77%, S_{trans}=13%
**T12** (Epp.)	90	30	(K$_2$[PtCl$_4$]) 1:2	Yes, 2 times water.	χ=94%, S_{cis}=87%, S_{trans}=7%

[Pt(COD)Cl$_2$] (2)					
**Number of trails**	**Time (min)**	**Frequency (Hz)**	**Ratio (M:L)**	**Washing**	**Comments**
**T13** (ZrJar)	60	20	(PtCl$_2$) 1:1	Yes, extract with CH$_2$Cl$_2$ and 3 times with pentane	χ=37%, S=37%.
**T14** (ZrJar)	60	30	(PtCl$_2$) 1:1	Yes, extract with CH$_2$Cl$_2$ and 3 times with pentane	χ=79%, S=79%
**T15** (ZrJar)	60	30	(PtCl$_2$) 1:1.2	Yes, extract with CH$_2$Cl$_2$ and 3 times with pentane	Selectivity after washing, S = 98%
**T16** (ZrJar)	60	30	(PtCl$_2$) 1:1.2; LAG of H$_2$O	Yes, extract with CH$_2$Cl$_2$ and 3 times with pentane	χ=90%, S=90%. Very dark gray color, not good.
**T17** (ZrJar)	90	30	(PtCl$_2$) 1:1	Yes, extract with CH$_2$Cl$_2$ and 3 times with pentane	χ=64%, S=64%. Dark gray color, not good.
**T18** (ZrJar)	30	30	(K$_2$[PtCl$_4$]) 1:1	Yes, 2 times water.	No reaction.

[Pt(phen)Cl$_2$] (3)					
**Number of trails**	**Time (min)**	**Frequency (Hz)**	**Ratio (M:L)**	**Washing**	**Comments**
**T19** (Epp.)*	120	30	(PtCl$_2$) 1:1	No	χ>99%, S>99%
**T20** (Epp.)*	90	30	(PtCl$_2$) 1:1	No	χ>99%, S>99%
**T21** (Epp.)*	60	30	(PtCl$_2$) 1:1	No	χ>99%, S>99%

**T22** (Epp.)*	45	30	($PtCl_2$) 1:1	No	χ>99%, S=83%
**T23** (Epp.)*	120	30	($K_2[PtCl_4]$) 1:1	Yes, 2 times water.	χ=27%, S=27%
**T24** (Epp.)*	45	30	($K_2[PtCl_4]$) 1:1	Yes, 2 times water.	χ=13%, S=13%

*Argon atmosphere

**[Pt(κ²-terpy)Cl₂] (4)**					
**Number of trails**	**Time (min)**	**Frequency (Hz)**	**Ratio (M:L)**	**Washing**	**Comments**
**T25** (Epp.)	120	30	($PtCl_2$) 1:1	No	χ>99%, S>99%
**T26** (Epp.)	120	30	($PtCl_2$) 1:1	No	χ>99%, S>99%
**T27** (Epp.)	120	30	($PtCl_2$) 1:1	No	χ>99%, S>99%
**T28** (Epp.)	90	30	($PtCl_2$) 1:1	No	χ>99%, S>99%
**T29** (Epp.)	90	30	($PtCl_2$) 1:1	No	χ>99%, S>99%
**T30** (Epp.)	120	30	($K_2[PtCl_4]$) 1:1	Yes, 2 times water.	χ>99%, S>99%

**[Pt(BIAN)Cl₂] (5)**					
**Number of trails**	**Time (min)**	**Frequency (Hz)**	**Ratio (M:L)**	**Washing**	**Comments**
**T31** (Epp.)*	180	30	($PtCl_2$) 1:1	Yes, 2 times ether.	χ=89%, S=89%
**T32** (Epp.)*	120	30	($PtCl_2$) 1:1	Yes, 2 times ether.	χ=89%, S=89%
**T33** (Epp.)*	90	30	($PtCl_2$) 1:1	Yes, 2 times ether.	χ=88%, S=88%
**T34** (Epp.)*	60	30	($PtCl_2$) 1:1	Yes, 2 times ether.	χ=67%, S=67%
**T35** (Epp.)*	180	30	($K_2[PtCl_4]$) 1:1	Yes, 2 times water.	χ=0%, S=0%

*Argon atmosphere

**[Pt(PPh₃)₂Cl₂] (6)**					
**Number of trails**	**Time (min)**	**Frequency (Hz)**	**Ratio (M:L)**	**Washing**	**Comments**
**T36** (Epp.)*	5	30	($PtCl_2$) 1:2	No	χ=82%, S_{cis}=81%, S_{trans}=1%
**T37** (Epp.)*	10	30	($PtCl_2$) 1:2	No	χ>99%, S_{cis}=76%, S_{trans}=24%
**T38** (Epp.)*	15	30	($PtCl_2$) 1:2	No	χ>99%, S_{cis}=78%, S_{trans}=22%
**T39** (Epp.)*	5	30	($K_2[PtCl_4]$) 1:2	Yes, 2 times water.	χ=4%, S_{cis}=4%
**T40** (Epp.)*	10	30	($K_2[PtCl_4]$) 1:2	Yes, 2 times water.	χ=19%, S_{cis}=19%
**T41** (Epp.)*	15	30	($K_2[PtCl_4]$) 1:2	Yes, 2 times water.	χ=53%, S_{cis}=52% S_{trans}=1%
**T42** (Epp.)*	45	30	($K_2[PtCl_4]$) 1:2	Yes, 2 times water.	χ=68%, S_{cis}=66%, S_{trans}=2%
**T43** (Epp.)*	60	30	($K_2[PtCl_4]$) 1:2	Yes, 2 times water.	χ=88%, S_{cis}=87%, PPh_3O=1%
**T44** (Epp.)*	90	30	($K_2[PtCl_4]$) 1:2	Yes, 2 times water.	χ=90%, S_{cis}=85%, PPh_3O=5%
**T45** (Epp.)*	120	30	($K_2[PtCl_4]$) 1:2	Yes, 2 times water.	χ=90%, S_{cis}=81%, PPh_3O=9%

*Argon atmosphere

**[Pt(P(3,5-(CF₃)₂Ph)₃)₂Cl₂] (7)**					
**Number of trails**	**Time (min)**	**Frequency (Hz)**	**Ratio (M:L)**	**Washing**	**Comments**
**T46** (Epp.)*	120	30	($PtCl_2$) 1:2	No	χ=90%, S=90%
**T47** (Epp.)*	120	30	($PtCl_2$) 1:2	No	χ=91%, S=91%
**T48** (Epp.)*	90	30	($PtCl_2$) 1:2	No	χ=91%, S=91%
**T49** (Epp.)*	120	30	($K_2[PtCl_4]$) 1:2	Yes, 2 times water.	χ=32%, S=32%

*Argon atmosphere

[Pt(Xantphos)Cl$_2$] (8)					
**Number of trails**	**Time (min)**	**Frequency (Hz)**	**Ratio (M:L)**	**Washing**	**Comments**
**T50** (Epp.)*	120	30	($PtCl_2$) 1:1	No	χ>99%, S>99%
**T51** (Epp.)*	90	30	($PtCl_2$) 1:1	No	χ>99%, S>99%
**T52** (Epp.)*	60	30	($PtCl_2$) 1:1	No	χ>99%, S>99%
**T53** (Epp.)*	45	30	($PtCl_2$) 1:1	No	χ>99%, S>99%
**T54** (Epp.)*	30	30	($PtCl_2$) 1:1	No	χ>99%, S>99%
**T55** (Epp.)*	20	30	($PtCl_2$) 1:1	No	χ>99%, S>99%
**T56** (Epp.)*	15	30	($PtCl_2$) 1:1	No	χ=94%, S=94%
**T57** (Epp.)*	120	30	($K_2[PtCl_4]$) 1:1	Yes, 2 times water.	χ=5%, S=5%
**T58** (SSjar)	120	30	($K_2[PtCl_4]$) 1:1	Yes, 2 times water.	χ=57%, S=57%

*Argon atmosphere

[Pt(dppm)Cl$_2$] (9)					
**Number of trails**	**Time (min)**	**Frequency (Hz)**	**Ratio (M:L)**	**Washing**	**Comments**
**T59** (Epp.)*	120	30	($PtCl_2$) 1:1	No	χ>99%, S>99%
**T60** (Epp.)*	90	30	($PtCl_2$) 1:1	No	χ>99%, S>99%
**T61** (Epp.)*	60	30	($PtCl_2$) 1:1	No	χ>99%, S>99%
**T62** (Epp.)*	45	30	($PtCl_2$) 1:1	No	χ>99%, S>99%
**T63** (Epp.)*	30	30	($PtCl_2$) 1:1	No	χ>99%, S>99%
**T64** (Epp.)*	15	30	($PtCl_2$) 1:1	No	χ>99%, S>99%
**T65** (Epp.)*	10	30	($PtCl_2$) 1:1	No	χ>99%, S>99%
**T66** (Epp.)*	120	30	($K_2[PtCl_4]$) 1:1	Yes, 2 times water.	χ>99%, S>99%
**T67** (Epp.)*	90	30	($K_2[PtCl_4]$) 1:1	Yes, 2 times water.	χ>99%, S>99%
**T68** (Epp.)*	60	30	($K_2[PtCl_4]$) 1:1	Yes, 2 times water.	χ=50%, S=50%
**T69** (Epp.)*	45	30	($K_2[PtCl_4]$) 1:1	Yes, 2 times water.	χ=41%, S=41%

*Argon atmosphere

[Pt(dppe)Cl$_2$] (10)					
**Number of trails**	**Time (min)**	**Frequency (Hz)**	**Ratio (M:L)**	**Washing**	**Comments**
**T70** (Epp.)*	120	30	($PtCl_2$) 1:1	No	χ>99%, S>99%
**T71** (Epp.)*	90	30	($PtCl_2$) 1:1	No	χ>99%, S>99%
**T72** (Epp.)*	60	30	($PtCl_2$) 1:1	No	χ>99%, S>99%
**T73** (Epp.)*	45	30	($PtCl_2$) 1:1	No	χ>99%, S>99%
**T74** (Epp.)*	30	30	($PtCl_2$) 1:1	No	χ>99%, S>99%
**T75** (Epp.)*	15	30	($PtCl_2$) 1:1	No	χ>99%, S>99%
**T76** (Epp.)*	10	30	($PtCl_2$) 1:1	No	χ>99%, S=70%
**T77** (Epp.)*	120	30	($K_2[PtCl_4]$) 1:1	Yes, 2 times water.	χ>99%, S>99%
**T78** (Epp.)*	90	30	($K_2[PtCl_4]$) 1:1	Yes, 2 times water.	χ>99%, S=84%
**T79** (Epp.)*	60	30	($K_2[PtCl_4]$) 1:1	Yes, 2 times water.	χ>99%, S=47%

*Argon atmosphere

[Pt(dppp)Cl$_2$] (11)					
**Number of trails**	**Time (min)**	**Frequency (Hz)**	**Ratio (M:L)**	**Washing**	**Comments**
**T80** (Epp.)*	120	30	($PtCl_2$) 1:1	No	χ>99%, S>99%
**T81** (Epp.)*	90	30	($PtCl_2$) 1:1	No	χ>99%, S>99%
**T82** (Epp.)*	60	30	($PtCl_2$) 1:1	No	χ>99%, S>99%
**T83** (Epp.)*	45	30	($PtCl_2$) 1:1	No	χ>99%, S>99%
**T84** (Epp.)*	30	30	($PtCl_2$) 1:1	No	χ>99%, S>99%
**T85** (Epp.)*	15	30	($PtCl_2$) 1:1	No	χ=94%, S=94%
**T86** (Epp.)*	10	30	($PtCl_2$) 1:1	No	Mixture of products.
**T87** (Epp.)*	120	30	($K_2[PtCl_4]$) 1:1	Yes, 2 times water.	χ>99%, S>99%
**T88** (Epp.)*	90	30	($K_2[PtCl_4]$) 1:1	Yes, 2 times water.	χ=87%, S=87%
**T89** (Epp.)*	60	30	($K_2[PtCl_4]$) 1:1	Yes, 2 times water.	χ=45%, S=45%

*Argon atmosphere

**$[Pt(dppb)Cl_2]$ (12)**					
**Number of trails**	**Time (min)**	**Frequency (Hz)**	**Ratio (M:L)**	**Washing**	**Comments**
**T90** (Epp.)*	120	30	($PtCl_2$) 1:1	No	χ>99%, S>99%
**T91** (Epp.)*	90	30	($PtCl_2$) 1:1	No	χ>99%, S>99%
**T92** (Epp.)*	60	30	($PtCl_2$) 1:1	No	χ>99%, S>99%
**T93** (Epp.)*	45	30	($PtCl_2$) 1:1	No	χ>99%, S>99%
**T94** (Epp.)*	30	30	($PtCl_2$) 1:1	No	χ>99%, S>99%
**T95** (Epp.)*	15	30	($PtCl_2$) 1:1	No	χ>99%, S>99%
**T96** (Epp.)*	10	30	($PtCl_2$) 1:1	No	χ>99%, S>99%
**T97** (Epp.)*	30	30	($PtCl_2$) 1:1.2	No	χ>99%, S>99%; 12% free dppb, 13% $dppbO_2$.
**T98** (Epp.)*	120	30	($K_2[PtCl_4]$) 1:1	Yes, 2 times water.	χ>99%, S>99%
**T99** (Epp.)*	90	30	($K_2[PtCl_4]$) 1:1	Yes, 2 times water.	χ>99%, S>99%
**T100** (Epp.)*	60	30	($K_2[PtCl_4]$) 1:1	Yes, 2 times water.	χ=97%, S=97%

*Argon atmosphere

**$[Pt(dppbz)Cl_2]$ (13)**					
**Number of trails**	**Time (min)**	**Frequency (Hz)**	**Ratio (M:L)**	**Washing**	**Comments**
**T101** (Epp.)*	120	30	($PtCl_2$) 1:1	No	χ>99%, S>99%
**T102** (Epp.)*	120	30	($PtCl_2$) 1:1	No	χ>99%, S>99%
**T103** (Epp.)*	60	30	($PtCl_2$) 1:1	No	χ>99%, S>99%
**T104** (Epp.)*	45	30	($PtCl_2$) 1:1	No	χ>99%, S>99%
**T105** (Epp.)*	30	30	($PtCl_2$) 1:1	No	χ>99%, S>99%
**T106** (Epp.)*	15	30	($PtCl_2$) 1:1	No	χ>99%, S>99%
**T107** (Epp.)*	10	30	($PtCl_2$) 1:1	No	χ>99%, S>99%
**T108** (Epp.)*	120	30	($K_2[PtCl_4]$) 1:1	Yes, 2 times water.	χ=30%, S=30%

*Argon atmosphere

**$[Pt(dppf)Cl_2]$ (14)**					
**Number of trails**	**Time (min)**	**Frequency (Hz)**	**Ratio (M:L)**	**Washing**	**Comments**
**T109** (Epp.)*	120	30	($PtCl_2$) 1:1	No	χ>99%, S>99%
**T110** (Epp.)*	90	30	($PtCl_2$) 1:1	No	χ>99%, S>99%
**T111** (Epp.)*	60	30	($PtCl_2$) 1:1	No	χ>99%, S>99%
**T112** (Epp.)*	45	30	($PtCl_2$) 1:1	No	χ>99%, S>99%
**T113** (Epp.)*	30	30	($PtCl_2$) 1:1	No	χ>99%, S>99%
**T114** (Epp.)*	15	30	($PtCl_2$) 1:1	No	χ>99%, S>99%
**T115** (Epp.)*	10	30	($PtCl_2$) 1:1	No	χ=94%, S=94%
**T116** (Epp.)*	120	30	($K_2[PtCl_4]$) 1:1	Yes, 2 times water.	χ>99%, S>99%
**T117** (Epp.)*	60	30	($K_2[PtCl_4]$) 1:1	Yes, 2 times water.	χ=58%, S=58%
**T118** (Epp.)*	60	30	($K_2[PtCl_4]$) 1:1	Yes, 2 times water.	χ=22%, S=22%

*Argon atmosphere

**$[Pt(dippf)Cl_2]$ (15)**					
**Number of trails**	**Time (min)**	**Frequency (Hz)**	**Ratio (M:L)**	**Washing**	**Comments**
**T119** (Epp.)*	120	30	($PtCl_2$) 1:1	No	χ>99%, S>99%
**T120** (Epp.)*	90	30	($PtCl_2$) 1:1	No	χ>99%, S>99%
**T121** (Epp.)*	60	30	($PtCl_2$) 1:1	No	χ>99%, S>99%
**T122** (Epp.)*	45	30	($PtCl_2$) 1:1	No	χ>99%, S>99%
**T123** (Epp.)*	30	30	($PtCl_2$) 1:1	No	χ>99%, S>99%
**T124** (Epp.)*	20	30	($PtCl_2$) 1:1	No	χ>99%, S>99%
**T125** (Epp.)*	15	30	($PtCl_2$) 1:1	No	χ=95%, S=25%
**T126** (Epp.)*	10	30	($PtCl_2$) 1:1	No	Mixture of products.
**T127** (Epp.)*	120	30	($K_2[PtCl_4]$) 1:1	Yes, 2 times water.	χ>99%, S>99%
**T128** (Epp.)*	60	30	($K_2[PtCl_4]$) 1:1	Yes, 2 times water.	χ=68%, S=31%

**T129** (Epp.)*	60	30	($K_2[PtCl_4]$) 1:1	Yes, 2 times water.	χ=22%, S=12%

*Argon atmosphere

**[Pt((*R*)-BINAP)Cl₂] (16)**					
**Number of trails**	**Time (min)**	**Frequency (Hz)**	**Ratio (M:L)**	**Washing**	**Comments**
**T130** (Epp.)*	120	30	($PtCl_2$) 1:1	No	χ>99%, S>99%
**T131** (Epp.)*	90	30	($PtCl_2$) 1:1	No	χ>99%, S>99%
**T132** (Epp.)*	60	30	($PtCl_2$) 1:1	No	χ>99%, S>99%
**T133** (Epp.)*	45	30	($PtCl_2$) 1:1	No	χ>99%, S>99%
**T134** (Epp.)*	30	30	($PtCl_2$) 1:1	No	χ>99%, S>99%
**T135** (Epp.)*	15	30	($PtCl_2$) 1:1	No	χ>99%, S>99%
**T136** (Epp.)*	10	30	($PtCl_2$) 1:1	No	χ=86%, S=86%
**T137** (Epp.)*	120	30	($K_2[PtCl_4]$) 1:1	Yes, 2 times water.	χ>99%, S=7%

*Argon atmosphere

Part 3. Calculations for *E-factor* and Effective Mass Yield (EMY)

E-factor and EMY are calculated according to the following expressions:

$$E - factor = \frac{Mass\ of\ waste}{Mass\ of\ desidered\ product}$$

$$\mathrm{EMY} = \frac{Mass\ of\ desidered\ product}{Total\ mass\ of\ material\ used}\ x\ 100$$

Table S1: Green metrics calculation for $[Pt(DMSO)_2Cl_2]$ (**1**).

**$[Pt(DMSO)_2Cl_2]$ (1)**	
**Mechanochemical synthesis**	
**Reactant**	**Amount (mg)**
$PtCl_2$	20.0
Dimethyl sulfoxide (DMSO)	11.7
Amount of product	31.7
Waste	≃0
Yield percentage	>99
E-factor	≃0
EMY	≃100
**Solution synthesis**	
**Reactant**	**Amount (g)**
$K_2[PtCl_4]$	7.00
Dimethyl sulfoxide (DMSO)	9.90
Amount of product	5.50
Waste: $KCl+H_2O+CH_2Cl_2+MgSO_4$	1.94+100+195+5=301.9
Yield percentage	92
E-factor	54.9
EMY	1.74

Table S2: Green metrics calculation for $[Pt(COD)Cl_2]$ (**2**).

**$[Pt(COD)Cl_2]$ (2)**	
**Mechanochemical synthesis**	
**Reactant**	**Amount (mg)**
$PtCl_2$	75.0
1,5-Cyclooctadiene (COD)	36.7
Amount of product	107.2
Waste: CH_2Cl_2+pentane	6650+5670=12320
Yield percentage	96 (respect of $PtCl_2$)
E-factor	115
EMY	0.86
**Solution synthesis**	
**Reactant**	**Amount (g)**
$K_2[PtCl_4]$	0.500
1,5-Cyclooctadiene (COD)	0.195
Amount of product	0.315
Waste: $KCl+H_2O+CH_3COOH$	0.17+40+10.5=50.67
Yield percentage	70
E-factor	160.9
EMY	0.62

Table S3: Green metrics calculation for [Pt(phen)Cl_2] (**3**).

**[Pt(phen)Cl_2] (3)**	
**Mechanochemical synthesis**	
**Reactant**	**Amount (mg)**
$PtCl_2$	15.0
1,10-phenanthroline (phen)	10.2
Amount of product	25.0
Waste	≃0
Yield percentage	>99
E-factor	≃0
EMY	≃100
**Solution synthesis**	
**Reactant**	**Amount (g)**
$K_2[PtCl_4]$	0.208
1,10-phenanthroline (phen)	0.09
Amount of product	0.214
Waste: KCl+H_2O+Et_2O+MeOH+HCl	0.075+55+14.2+15.84+1=86.115
Yield percentage	96
E-factor	402.4
EMY	0.25

Table S4: Green metrics calculation for [Pt(κ^2-terpy)Cl_2] (**4**).

**[Pt(κ^2-terpy)Cl_2] (4)**	
**Mechanochemical synthesis**	
**Reactant**	**Amount (mg)**
$PtCl_2$	15.0
2,2';6',2"-terpyridine (terpy)	13.2
Amount of product	28.1
Waste	≃0
Yield percentage	>99
E-factor	≃0
EMY	≃100

Table S5: Green metrics calculation for [Pt(BIAN)Cl_2] (**5**).

**[Pt(BIAN)Cl_2] (5)**	
**Mechanochemical synthesis**	
**Reactant**	**Amount (mg)**
$PtCl_2$	10.0
N[1],*N*[2]-bis(2,6-diisopropylphenyl)acenaphthylene-1,2-diimine (BIAN)	18.8
Amount of product	25.3
Waste: Et_2O	3.5+2670=2673.5
Yield percentage	88
E-factor	106
EMY	0.94
**Solution synthesis**	
**Reactant**	**Amount (g)**
$K_2[PtCl_4]$	0.200
N[1],*N*[2]-bis(2,6-diisopropylphenyl)acenaphthylene-1,2-diimine (BIAN)	0.241
Amount of product	0.185
Waste: KCl+EtOH+CH_2Cl_2+*n*-exane+Et_2O	0.23+24+9.3+6=39.53
Yield percentage	50
E-factor	214
EMY	0.47

Table S6: Green metrics calculation for $[Pt(PPh_3)_2Cl_2]$ (**6**).

**$[Pt(PPh_3)_2Cl_2]$ (6)**	
**Mechanochemical synthesis**	
**Reactant**	**Amount (mg)**
$PtCl_2$	10.0
Triphenylphosphine (PPh_3)	19.7
Amount of product	29.7
Waste	≃0
Yield percentage	>99
E-factor	≃0
EMY	≃100
**Solution synthesis**	
**Reactant**	**Amount (g)**
$K_2[PtCl_4]$	4.15
Triphenylphosphine (PPh_3)	7.90
Amount of product	7.46
Waste: KCl+Xylene+EtOH+H_2O+Et_2O	1.49+43.5+31.56+30+14.2=120.75
Yield percentage	94
E-factor	16.19
EMY	5.68

Table S7: Green metrics calculation for $[Pt(P(3,5\text{-}(CF_3)_2Ph)_3)_2Cl_2]$ (**7**)

**$[Pt(P(3,5\text{-}(CF_3)_2Ph)_3)_2Cl_2]$ (7)**	
**Mechanochemical synthesis**	
**Reactant**	**Amount (mg)**
$PtCl_2$	10.0
tris[3,5-bis(trifluoromethyl)phenyl]phosphine	50.4
Amount of product	55.0
Waste	5.4
Yield percentage	91
E-factor	0.1
EMY	91
**Solution synthesis**	
**Reactant**	**Amount (mg)**
$[Pt(DMSO)_2Cl_2]$	20.0
tris[3,5-bis(trifluoromethyl)phenyl]phosphine	69.0
Amount of product	59.0
Waste: DMSO+C_6HF_5+Toluene	25+16+8000+12180=20221
Yield percentage	72
E-factor	342
EMY	0.29

Table S8: Green metrics calculation for $[Pt(Xantphos)Cl_2]$ (**8**)

**$[Pt(Xantphos)Cl_2]$ (8)**	
**Mechanochemical synthesis**	
**Reactant**	**Amount (mg)**
$PtCl_2$	10.0
Xantphos	21.8
Amount of product	31.6
Waste	≃0
Yield percentage	>99
E-factor	≃0
EMY	≃100
**Solution synthesis**	

Reactant	Amount (g)
[Pt(COD)Cl_2]	0.187
Xantphos	0.289
Amount of product	0.298
Waste: CH_2Cl_2+Et_2O	0.054+13.3+7.1=20.45
Yield percentage	90
E-factor	68.64
EMY	1.43

Table S9: Green metrics calculation for [Pt(dppm)Cl_2] (**9**)

**[Pt(dppm)Cl_2] (9)**	
**Mechanochemical synthesis**	
**Reactant**	**Amount (mg)**
$PtCl_2$	15.0
bis(diphenylphosphino)methane (dppm)	21.7
Amount of product	36.5
Waste	≃0
Yield percentage	>99
E-factor	≃0
EMY	≃100
**Solution synthesis**	
**Reactant**	**Amount (g)**
[Pt(PhCN)$_2$$Cl_2$]	1.18
bis(diphenylphosphino)methane (dppm)	0.961
Amount of product	1.48
Waste: CH_2Cl_2+Et_2O	0.515+13.3+7.1=20.92
Yield percentage	90
E-factor	14.12
EMY	6.56

Table S10: Green metrics calculation for [Pt(dppe)Cl_2] (**10**)

**[Pt(dppe)Cl_2] (10)**	
**Mechanochemical synthesis**	
**Reactant**	**Amount (mg)**
$PtCl_2$	15.0
bis(diphenylphosphino)ethane (dppe)	21.7
Amount of product	36.5
Waste	≃0
Yield percentage	>99
E-factor	≃0
EMY	≃100
**Solution synthesis**	
**Reactant**	**Amount (g)**
[Pt(PhCN)$_2$$Cl_2$]	1.18
bis(diphenylphosphino)ethane (dppe)	0.996
Amount of product	1.49
Waste: CH_2Cl_2+Et_2O	0.515+13.3+7.1=20.92
Yield percentage	90
E-factor	14.04
EMY	6.60

Table S11: Green metrics calculation for [Pt(dppp)Cl_2] (**11**)

**[Pt(dppp)Cl_2] (11)**	
**Mechanochemical synthesis**	
**Reactant**	**Amount (mg)**
$PtCl_2$	15.0
bis(diphenylphosphino)propane (dppp)	23.3
Amount of product	38.1
Waste	≃0
Yield percentage	>99
E-factor	≃0
EMY	≃100
**Solution synthesis**	
**Reactant**	**Amount (g)**
[Pt(PhCN)$_2$$Cl_2$]	1.18
bis(diphenylphosphino)propane (dppp)	1.03
Amount of product	1.52
Waste: CH_2Cl_2+Et_2O	0.515+13.3+7.1=20.92
Yield percentage	90
E-factor	13.72
EMY	6.73

Table S12: Green metrics calculation for [Pt(dppb)Cl_2] (**12**)

**[Pt(dppb)Cl_2] (12)**	
**Mechanochemical synthesis**	
**Reactant**	**Amount (mg)**
$PtCl_2$	15.0
bis(diphenylphosphino)butane (dppb)	24.0
Amount of product	38.9
Waste	≃0
Yield percentage	>99
E-factor	≃0
EMY	≃100
**Solution synthesis**	
**Reactant**	**Amount (g)**
[Pt(PhCN)$_2$$Cl_2$]	1.18
bis(diphenylphosphino)butane (dppb)	1.06
Amount of product	1.56
Waste: CH_2Cl_2+Et_2O	0.515+13.3+7.1=20.92
Yield percentage	90
E-factor	13.42
EMY	6.89

Table S13: Green metrics calculation for [Pt(dppbz)Cl_2] (**13**)

**[Pt(dppbz)Cl_2] (13)**	
**Mechanochemical synthesis**	
**Reactant**	**Amount (mg)**
$PtCl_2$	10.0
bis(diphenylphosphino)benzene (dppbz)	16.8
Amount of product	26.7
Waste	≃0
Yield percentage	>99
E-factor	≃0
EMY	≃100
**Solution synthesis**	
**Reactant**	**Amount (mg)**
[Pt(COD)Cl_2]	36.0

bis(diphenylphosphino)benzene (dppbz)	43.0
Amount of product	65.1
Waste: CH_2Cl_2+Et_2O	14+19500+7100=26614
Yield percentage	95
E-factor	409
EMY	0.24

Table S14: Green metrics calculation for [Pt(dppf)Cl_2] (**14**)

**[Pt(dppf)Cl_2] (14)**	
**Mechanochemical synthesis**	
**Reactant**	**Amount (mg)**
$PtCl_2$	10.0
bis(diphenylphosphino)ferrocene (dppf)	20.8
Amount of product	30.7
Waste	≃0
Yield percentage	>99
E-factor	≃0
EMY	≃100
**Solution synthesis**	
**Reactant**	**Amount (g)**
$PtCl_2$	0.266
bis(diphenylphosphino)ferrocene (dppf)	0.554
Amount of product	0.763
Waste: Benzene+EtOH	26.36+7.89=34.25
Yield percentage	93
E-factor	43.85
EMY	2.23

Table S15: Green metrics calculation for [Pt(dippf)Cl_2] (**15**)

**[Pt(dippf)Cl_2] (15)**	
**Mechanochemical synthesis**	
**Reactant**	**Amount (mg)**
$PtCl_2$	10.0
bis(diisopropylphosphino)ferrocene (dippf)	15.7
Amount of product	25.6
Waste	≃0
Yield percentage	>99
E-factor	≃0
EMY	≃100
**Solution synthesis**	
**Reactant**	**Amount (g)**
[Pt(CH_3CN)$_2$$Cl_2$]	0.0703
bis(diisopropylphosphino)ferrocene (dippf)	0.0841
Amount of product	0.0440
Waste: CH_2Cl_2+*n*-exane+Et_2O	0.11+33.25+13.20+7.10=53.66
Yield percentage	32
E-factor	1220
EMY	0.08

Table S16: Green metrics calculation for [Pt((*R*)-BINAP)Cl_2] (**16**)

**[Pt((R)-BINAP)Cl_2] (16)**	
**Mechanochemical synthesis**	
**Reactant**	**Amount (mg)**
$PtCl_2$	10.0
(*R*)-2,2′-bis(diphenylphosphino)-1,1′-binaphthyl ((*R*)-BINAP)	23.4

Amount of product	33.2
Waste	≃0
Yield percentage	>99
E-factor	≃0
EMY	≃100
**Solution synthesis**	
**Reactant**	**Amount (g)**
[Pt(COD)Cl_2]	0.598
(*R*)-2,2′-bis(diphenylphosphino)-1,1′-binaphthyl ((*R*)-BINAP)	0.801
Amount of product	0.957
Waste: $CHCl_3$+Et_2O	0.173+58.8+21.3=80.27
Yield percentage	78
E-factor	83.88
EMY	1.17

Part 4. NMR's characterizations spectra's

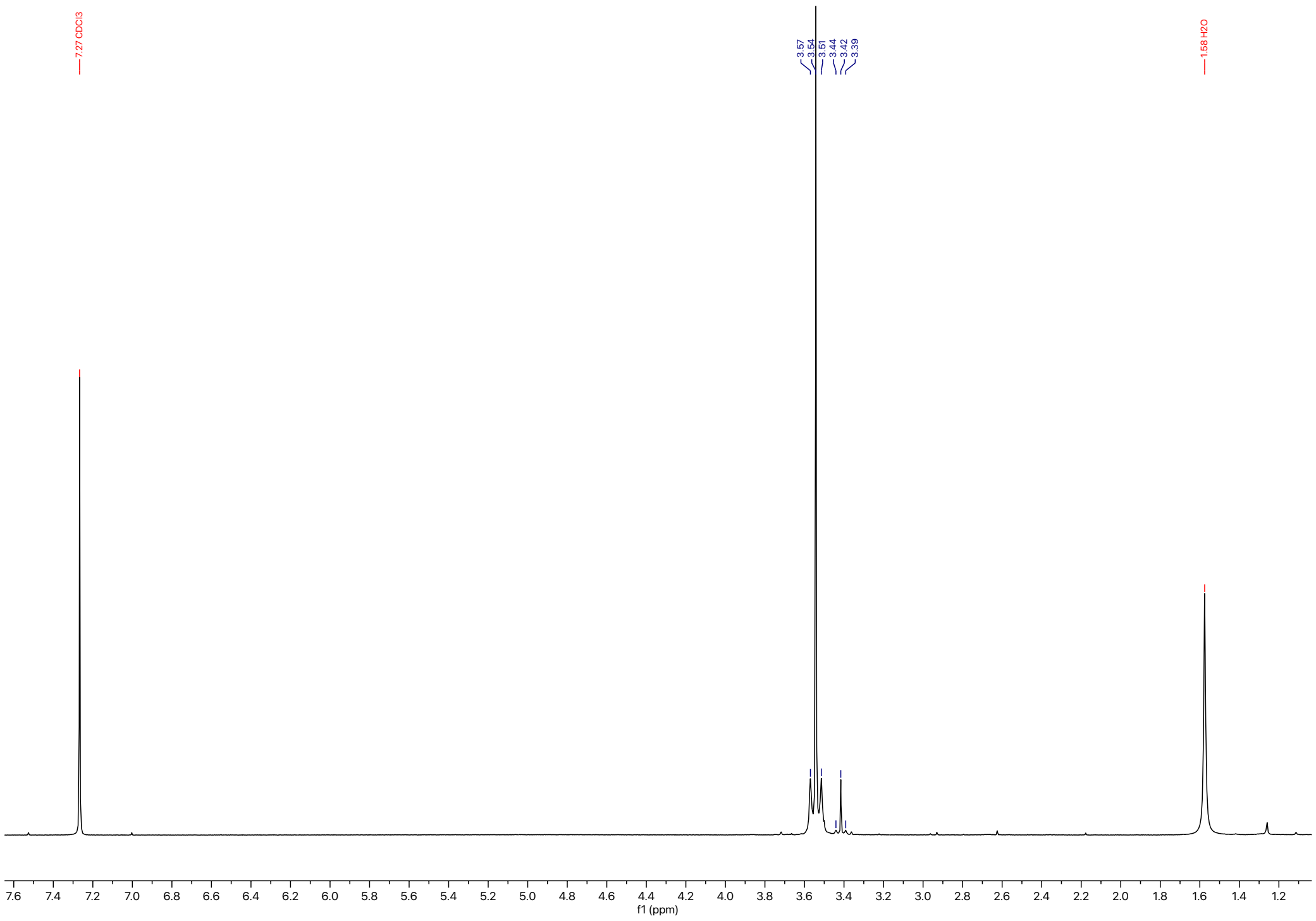

Figure S5. ^{1}H NMR spectra of $[Pt(DMSO)_2Cl_2]$ (**1**) (400.1 MHz, $CDCl_3$, 298K).

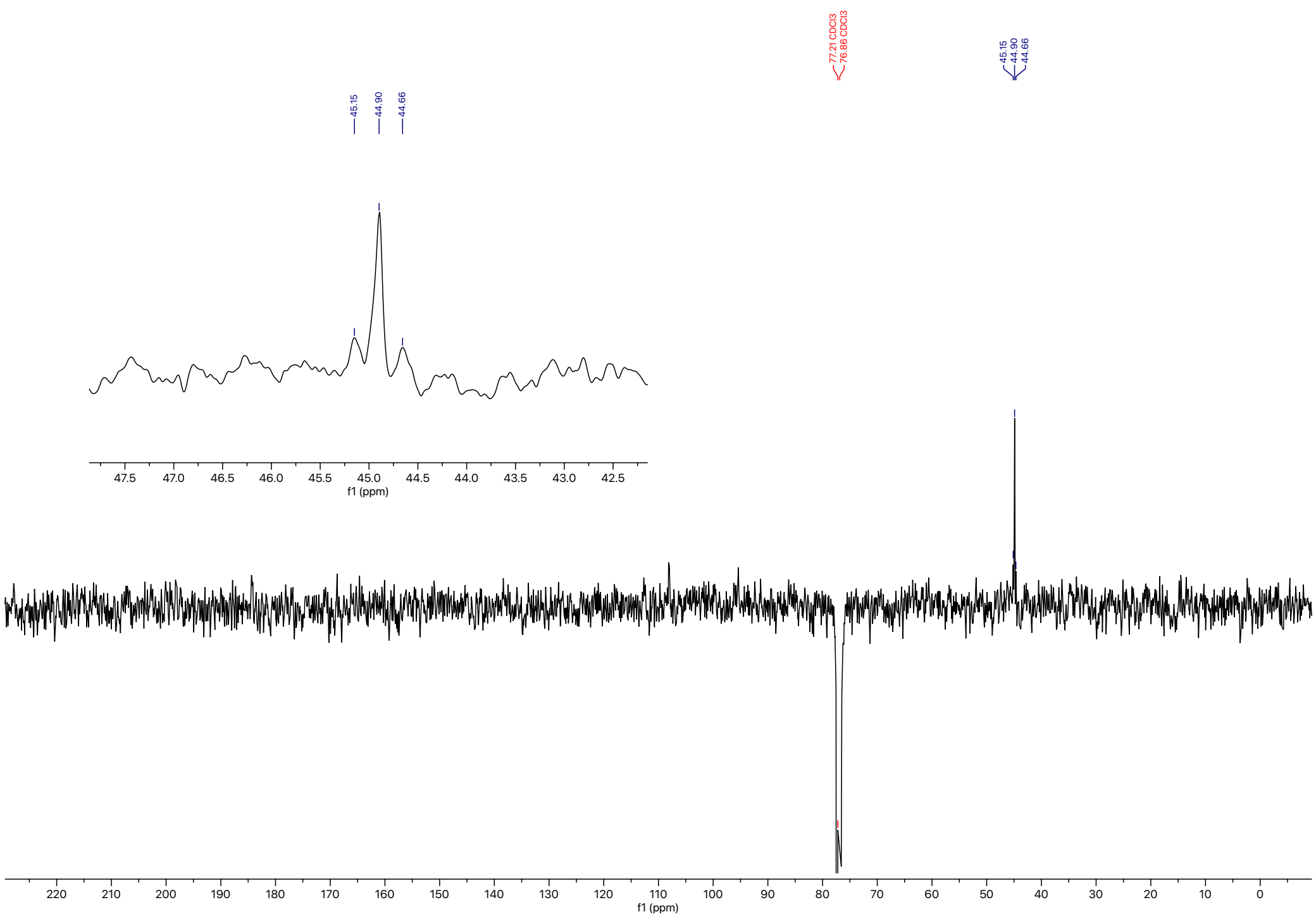

Figure S6. $^{13}C\{^{1}H\}$ NMR spectra of $[Pt(DMSO)_2Cl_2]$ (**1**) (100.6 MHz, $CDCl_3$, 298K).

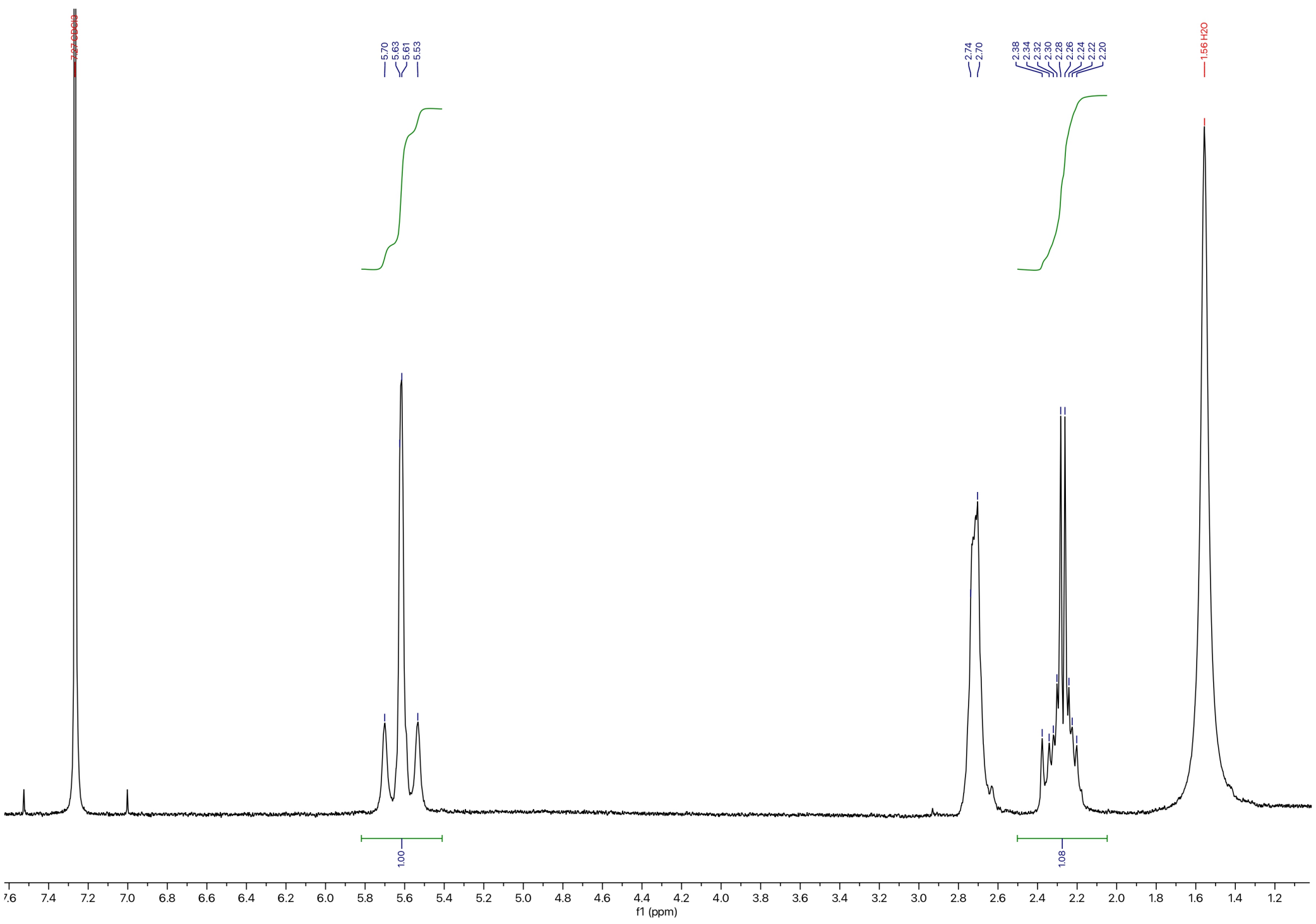

Figure S7. ^{1}H NMR spectra of [Pt(COD)Cl$_2$] (**2**) (400.1 MHz, $CDCl_3$, 298K).

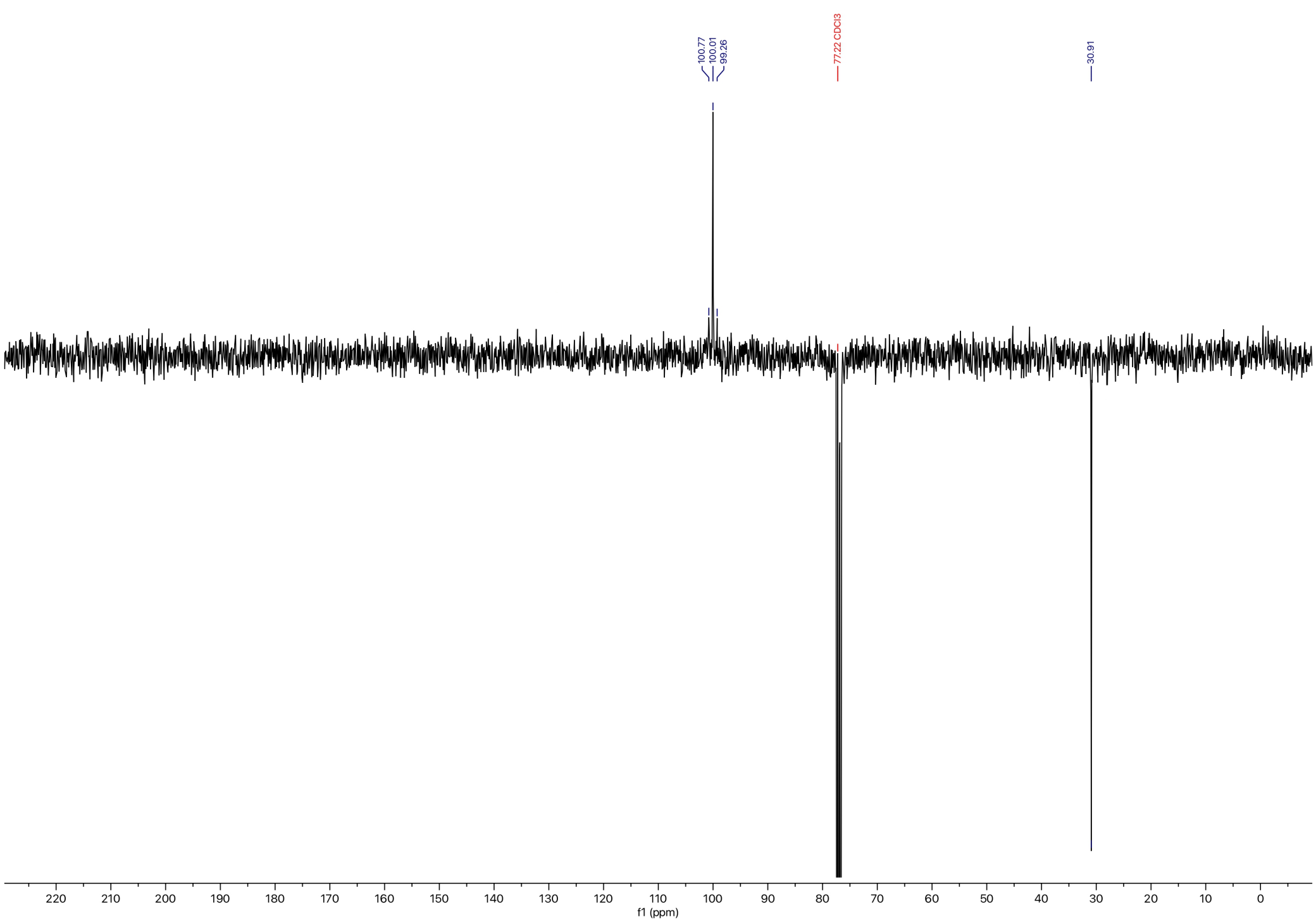

Figure S8. $^{13}C\{^{1}H\}$ NMR spectra of [Pt(COD)Cl$_2$] (**2**) (100.6 MHz, $CDCl_3$, 298K).

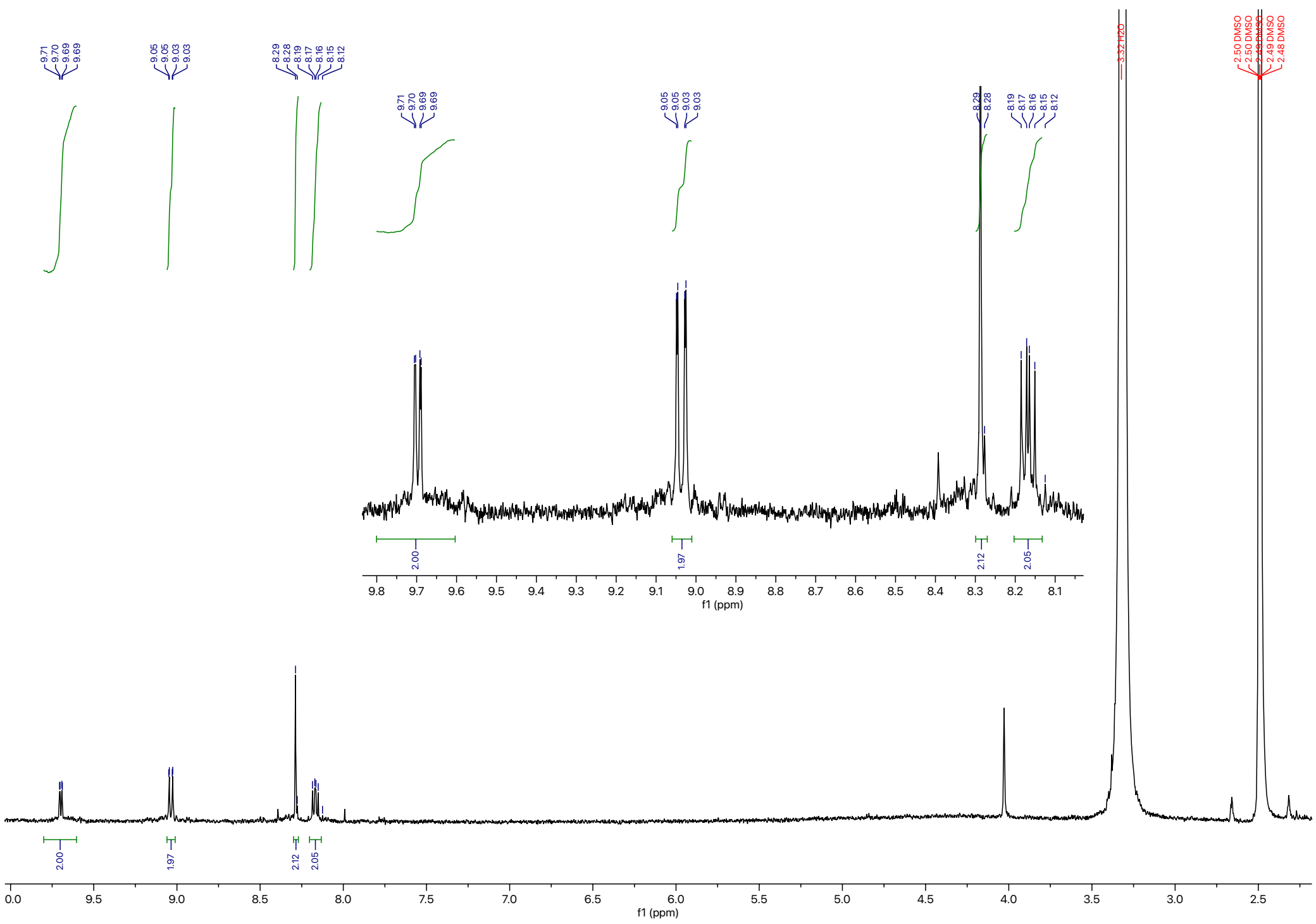

Figure S9. ^{1}H NMR spectra of $[Pt(phen)Cl_2]$ (**3**) (400.1 MHz, DMSO-d_6, 298K).

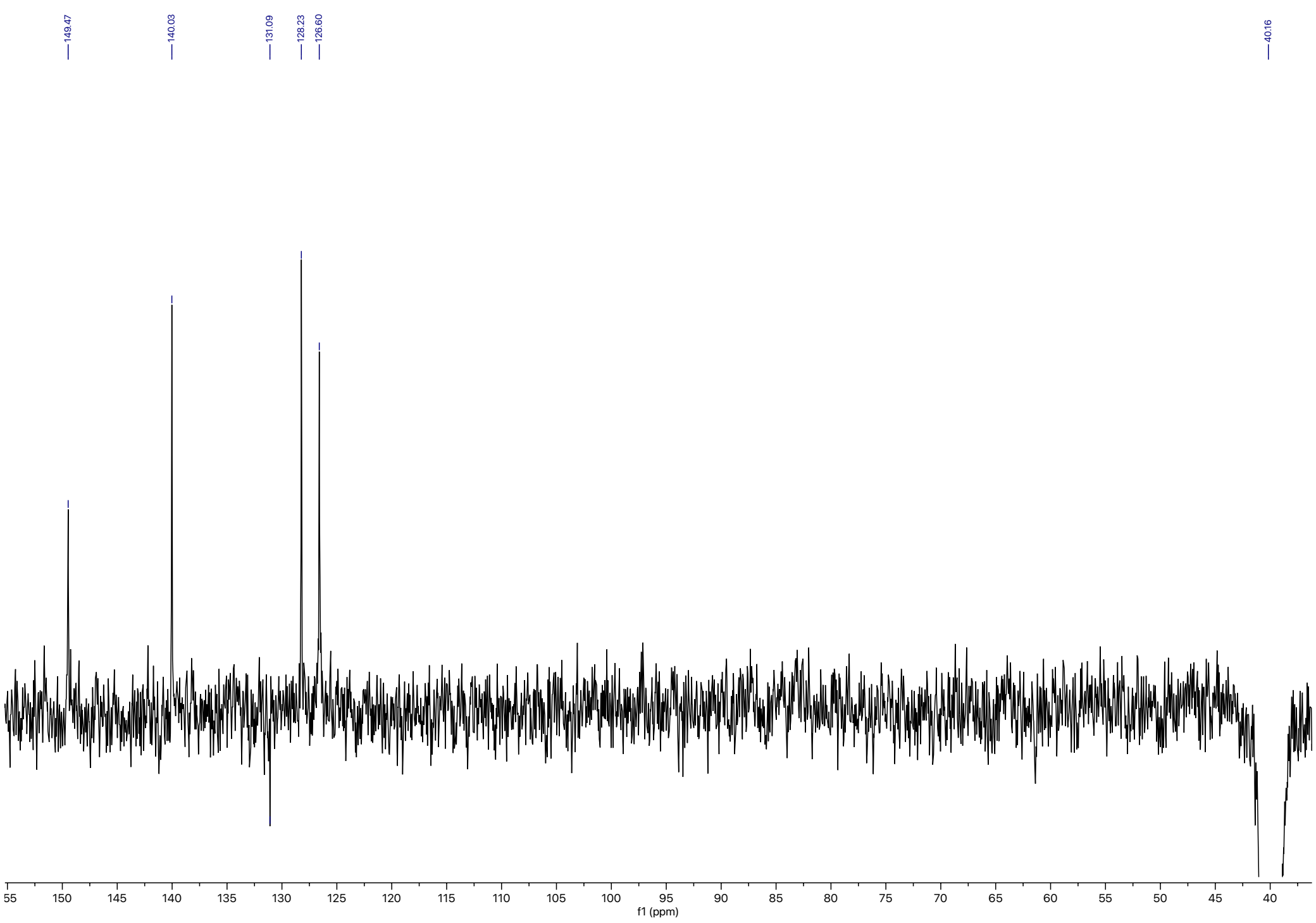

Figure S10. $^{13}C\{^{1}H\}$ NMR spectra of $[Pt(phen)Cl_2]$ (**3**) (100.6 MHz, DMSO-d_6, 298K).

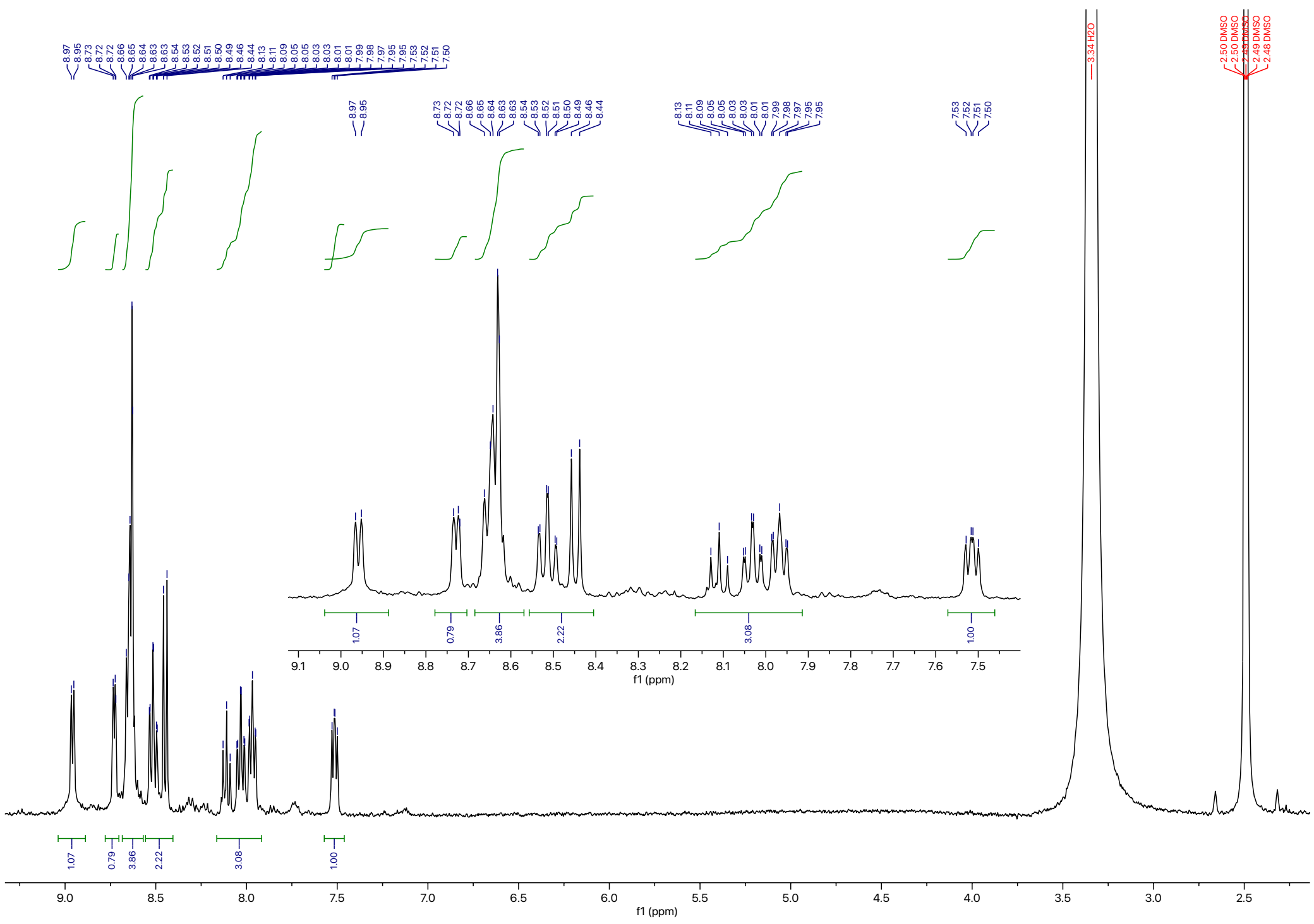

Figure S11. ^{1}H NMR spectra of [Pt(κ^2-terpy)Cl_2] (**4**) (400.1 MHz, DMSO-d_6, 298K).

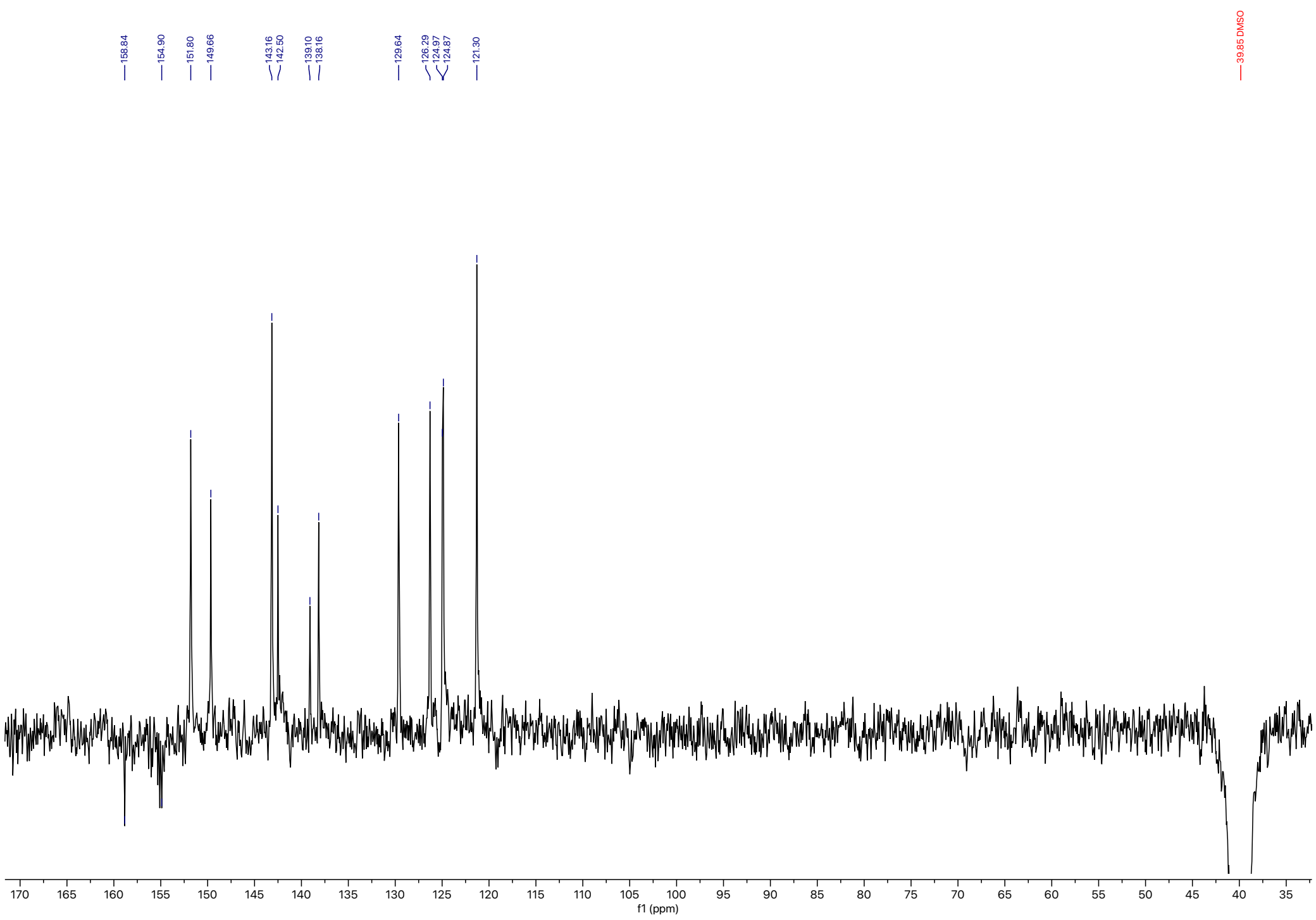

Figure S12. $^{13}C\{^{1}H\}$ NMR spectra of [Pt(κ^2-terpy)Cl_2] (**4**) (100.6 MHz, DMSO-d_6, 298K).

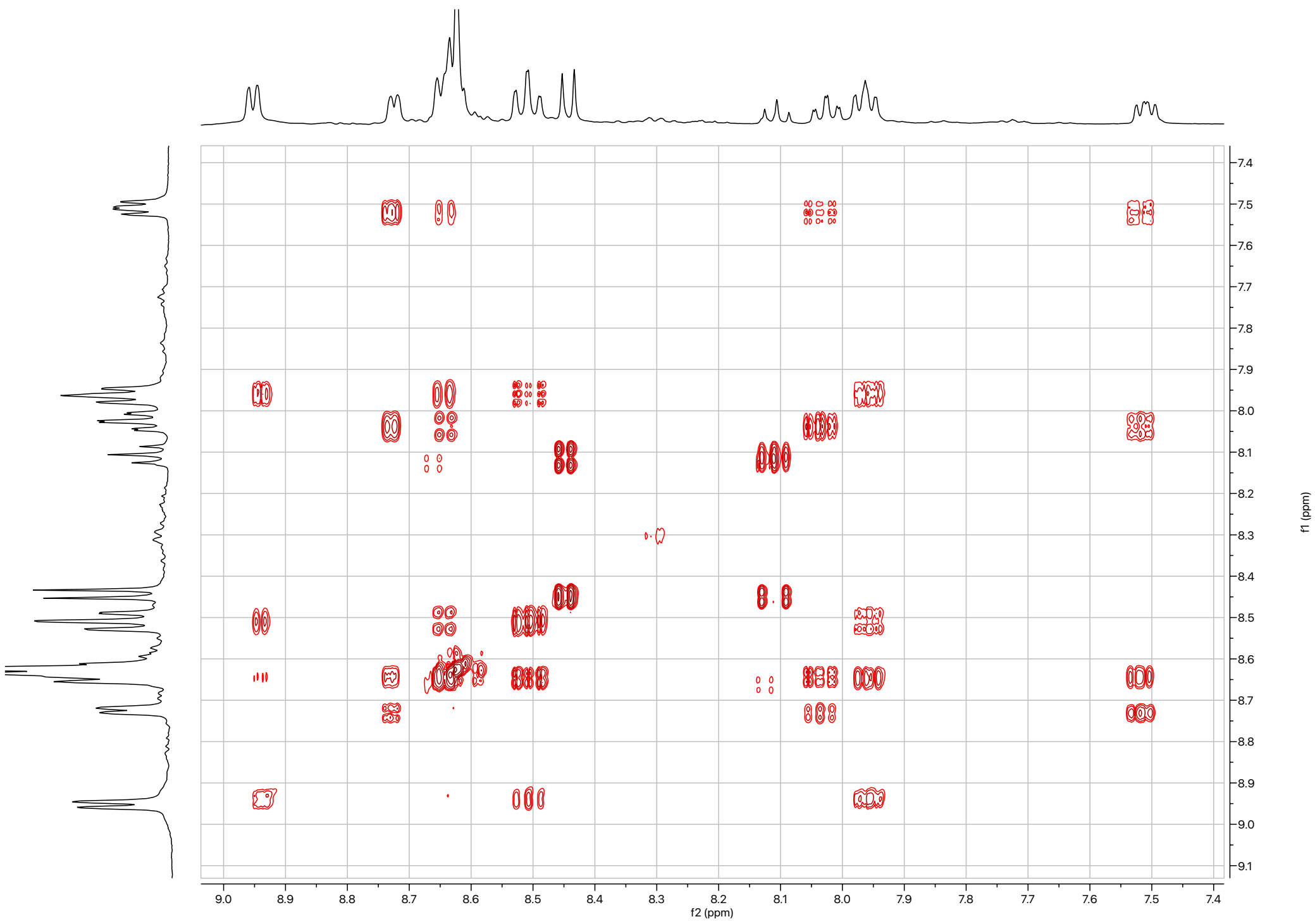

Figure S13. ^{1}H-^{1}H COSY spectra of [Pt(κ^2-terpy)Cl_2] (**4**) (400.1 MHz, DMSO-d_6, 298K).

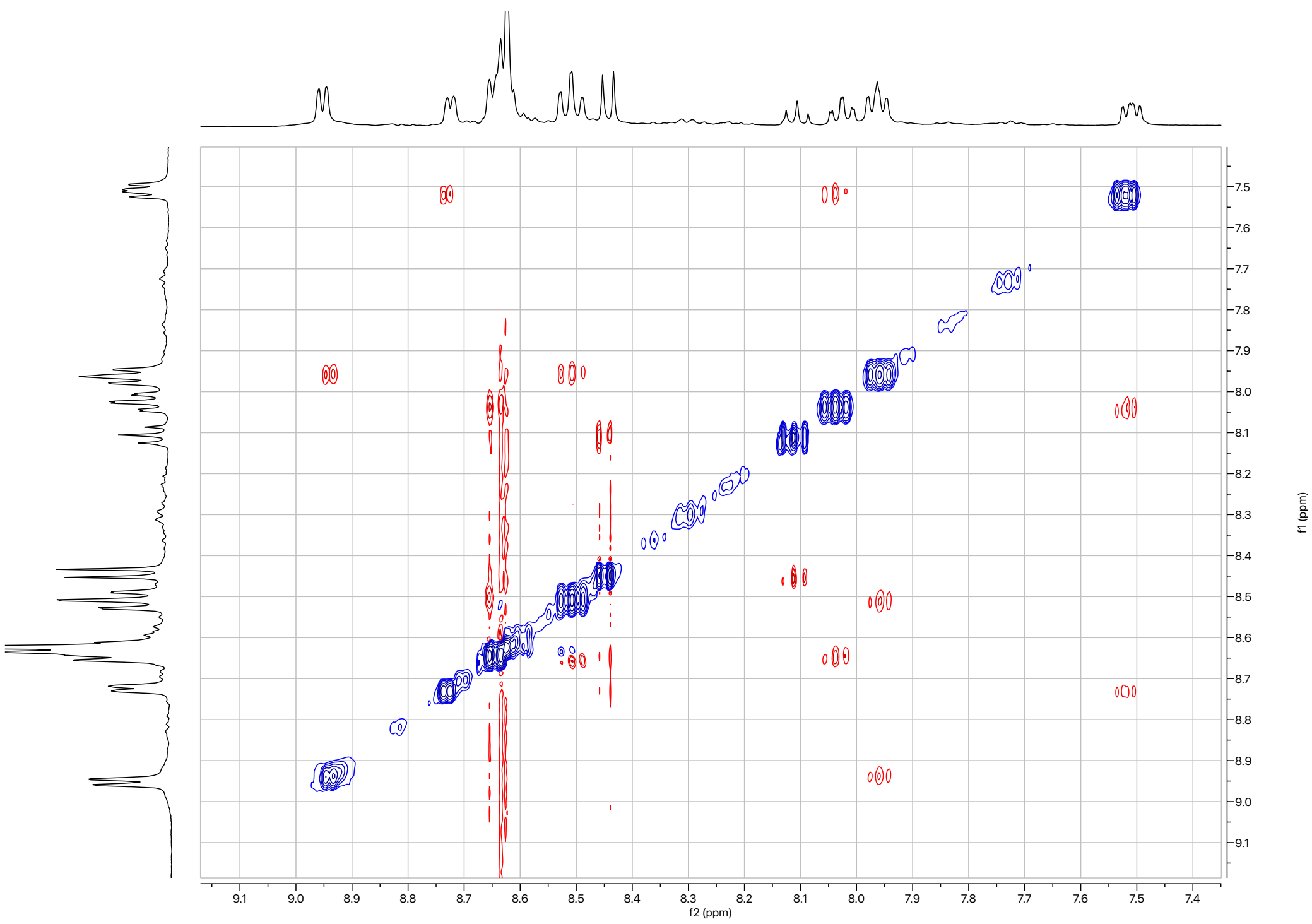

Figure S14. ^{1}H-^{1}H NOESY spectra of [Pt(κ^2-terpy)Cl_2] (**4**) (400.1 MHz, DMSO-d_6, 298K).

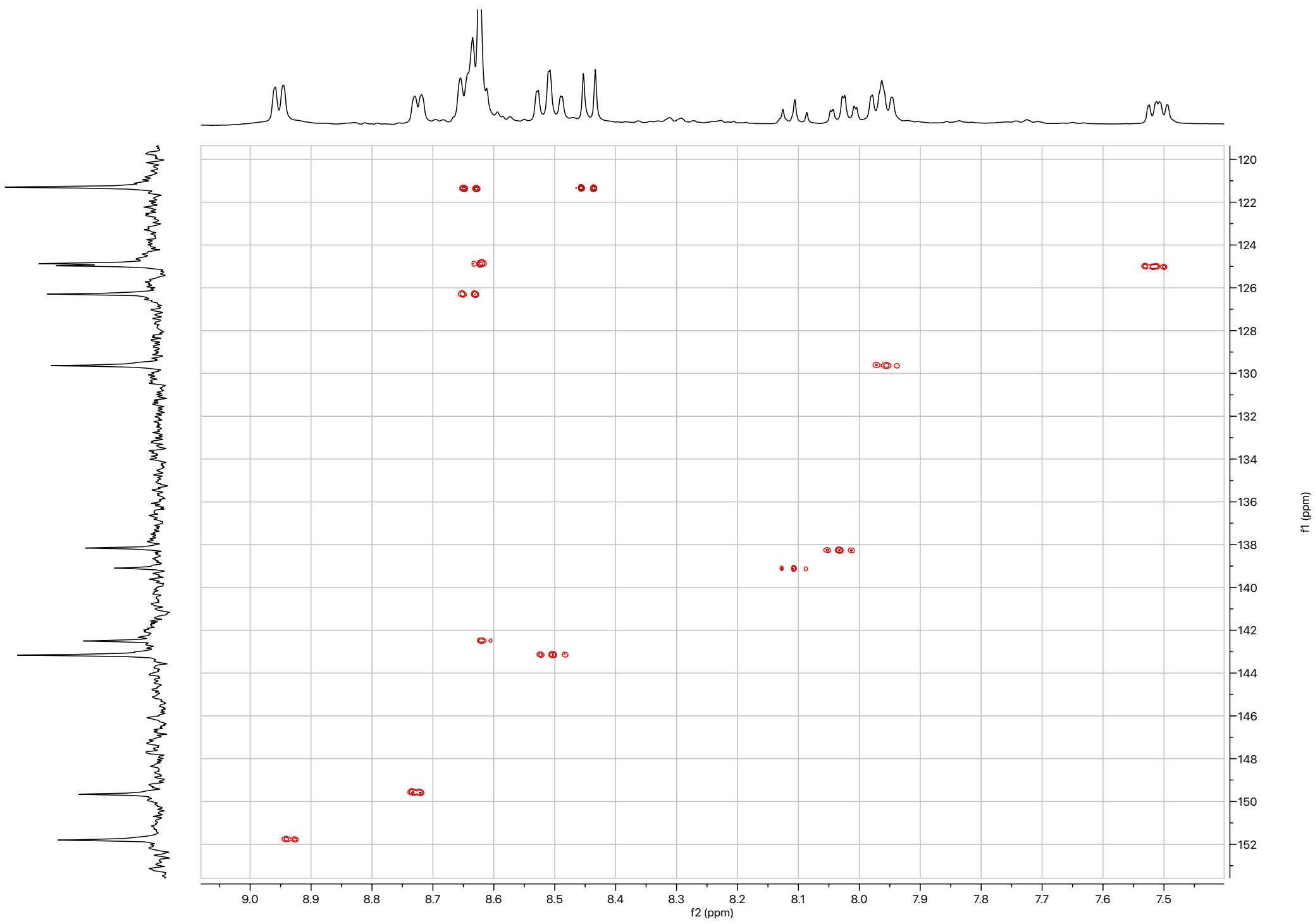

Figure S15. ^{1}H-^{13}C HSQC spectra of [Pt(κ^2-terpy)Cl_2] (**4**) (400.1 MHz, DMSO-d_6, 298K).

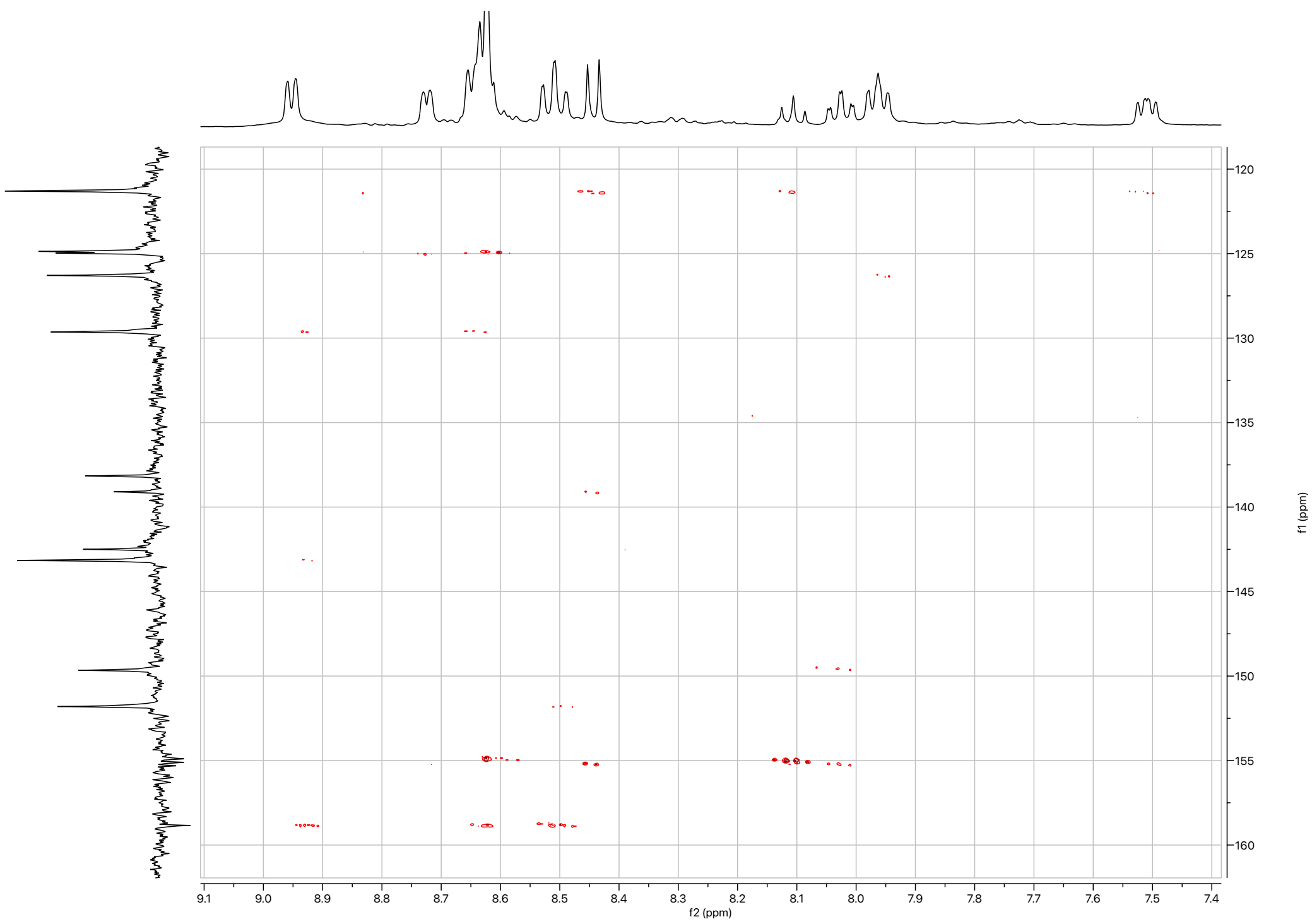

Figure S16. ^{1}H-^{13}C HMBC spectra of [Pt(κ^2-terpy)Cl_2] (**4**) (400.1 MHz, DMSO-d_6, 298K).

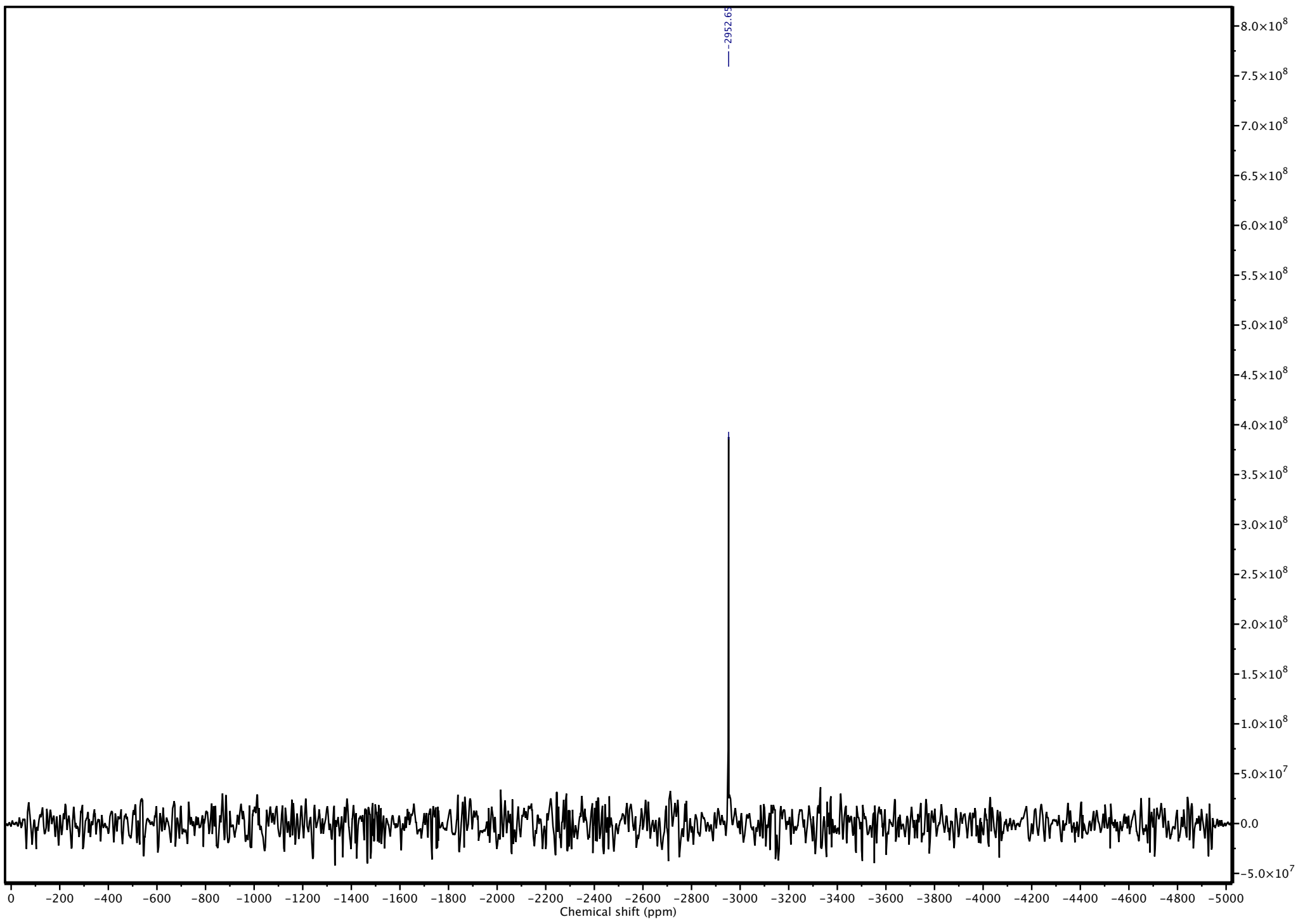

Figure S17. ^{195}Pt NMR spectra of [Pt(κ^2-terpy)Cl_2] (**4**) (85.9 MHz, DMSO-d_6, 298K).

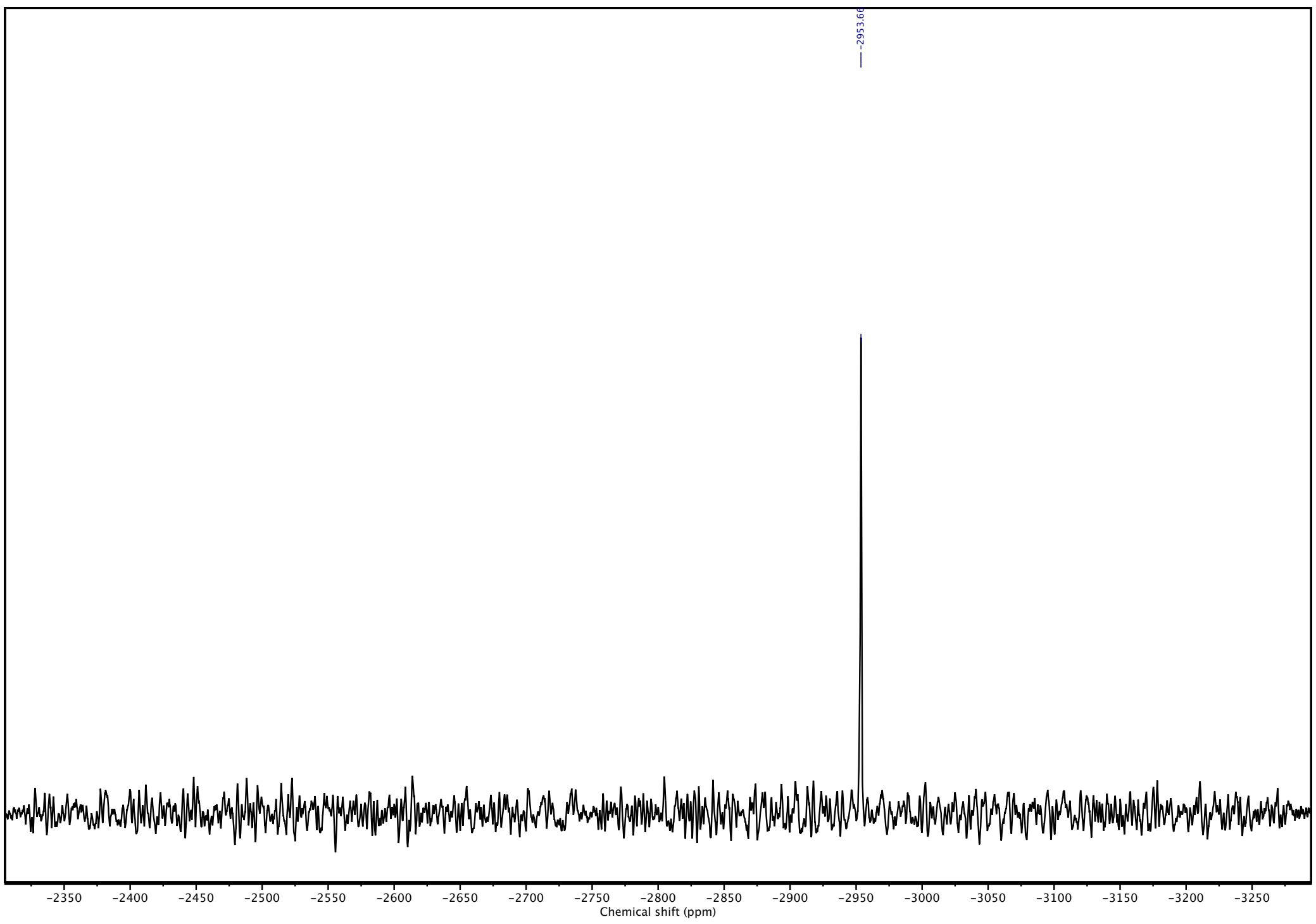

Figure S18. ^{195}Pt NMR spectra of [Pt(κ^2-terpy)Cl_2] (**4**) (85.9 MHz, DMSO-d_6, 298K).

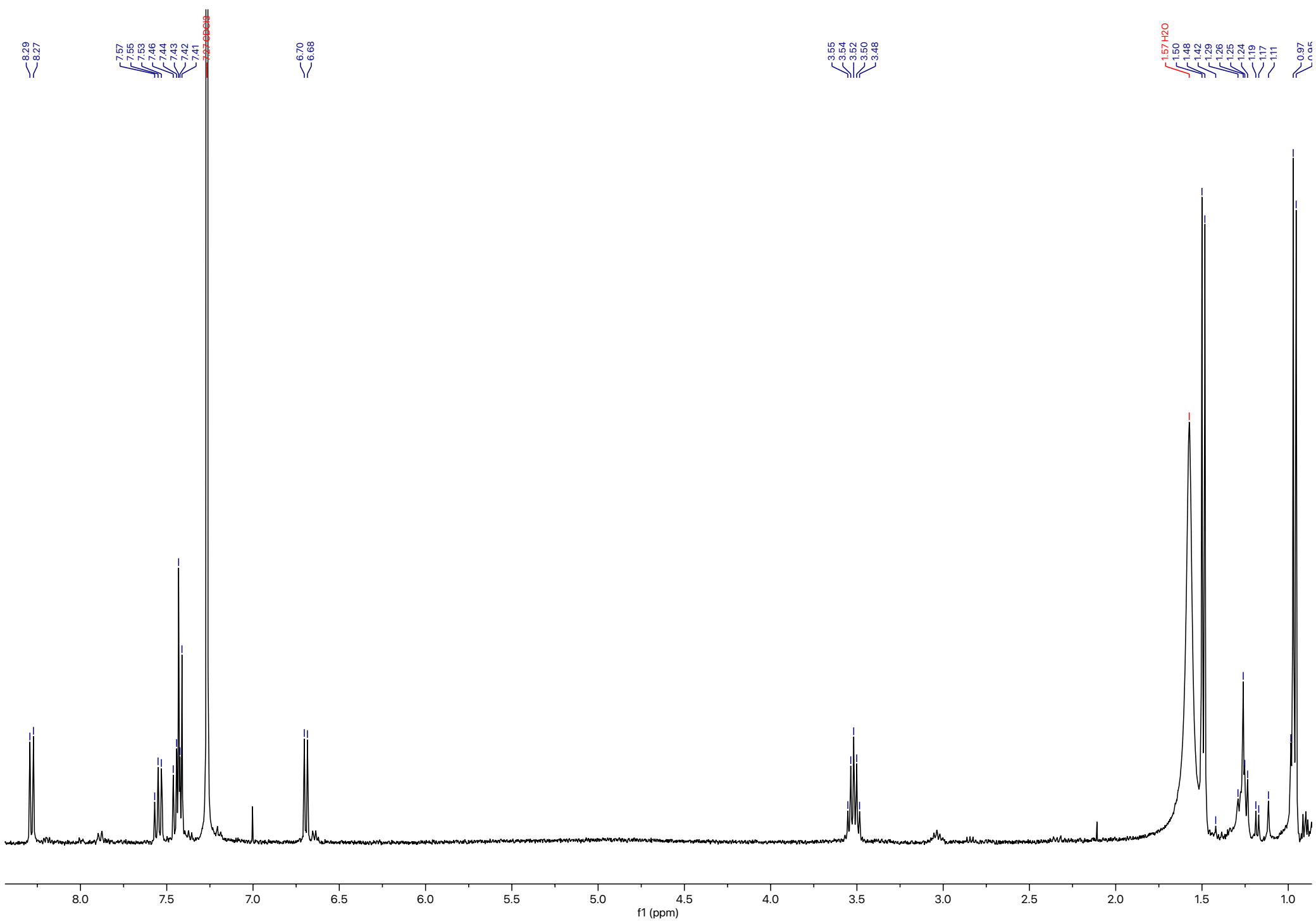

Figure S19. ^{1}H NMR spectra of [Pt(BIAN)Cl_2] (**5**) (400.1 MHz, $CDCl_3$, 298K).

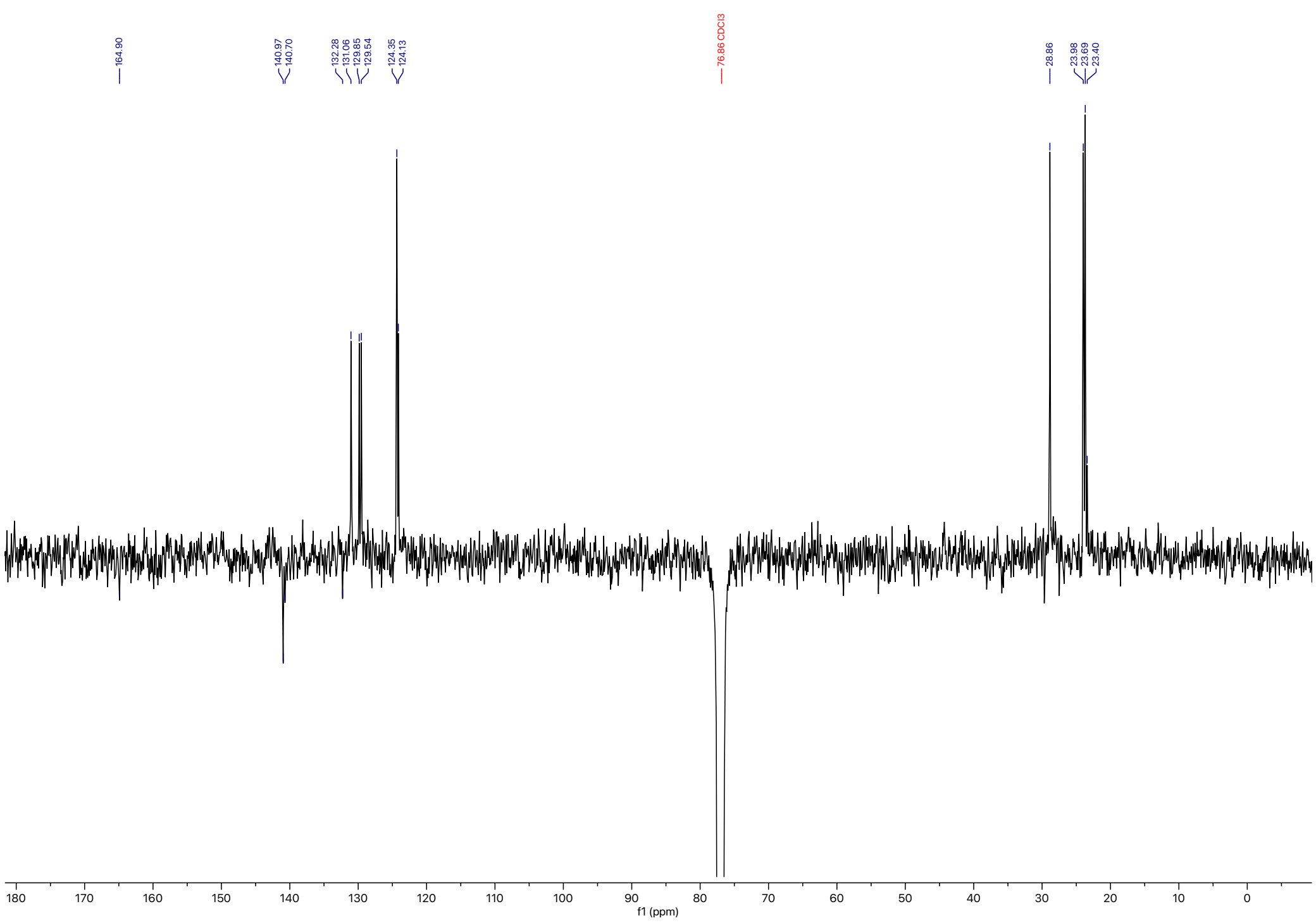

Figure S20. $^{13}C\{^{1}H\}$ NMR spectra of [Pt(BIAN)Cl_2] (**5**) (100.6 MHz, $CDCl_3$, 298K).

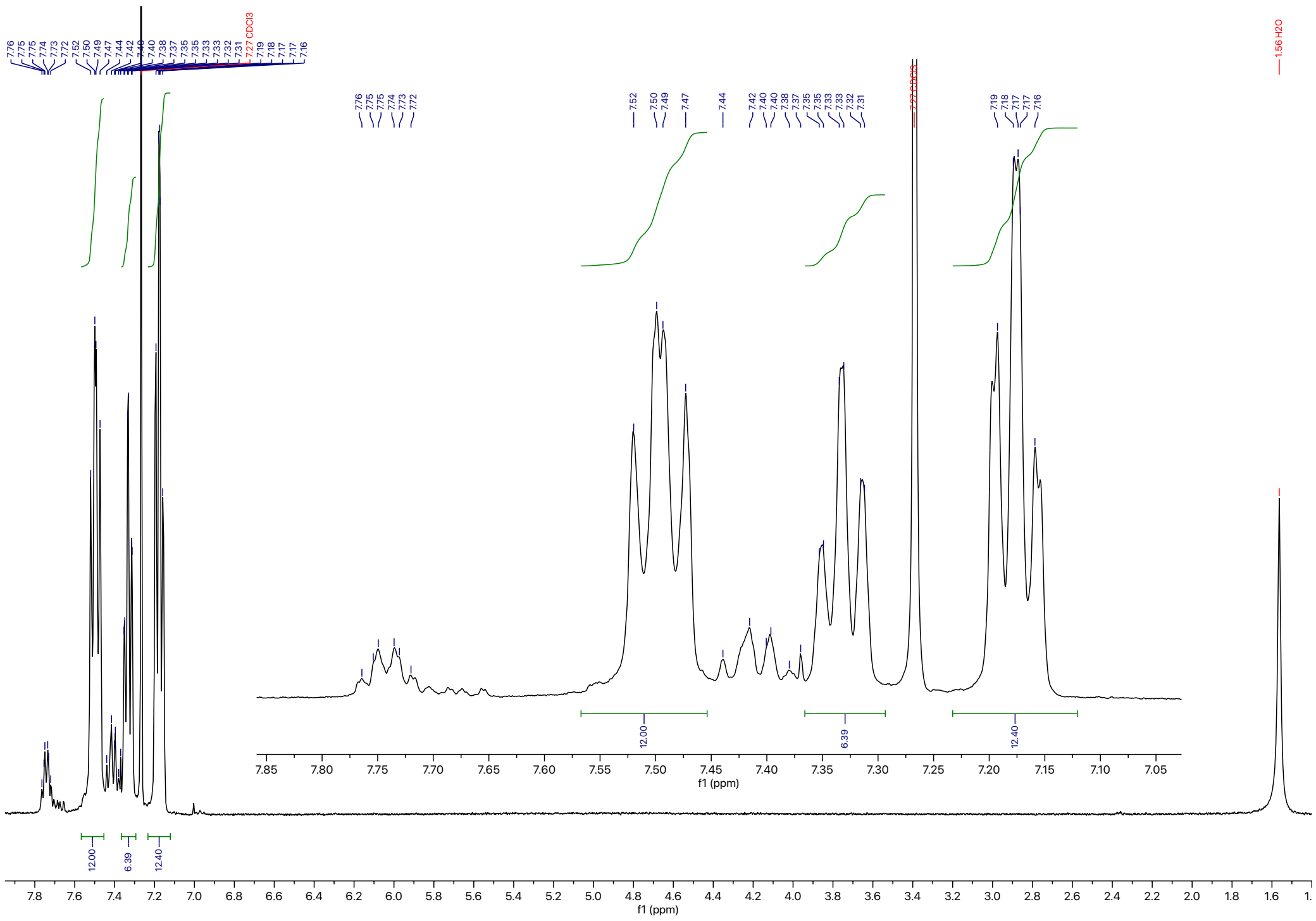

Figure S21. ^{1}H NMR spectra of [Pt(PPh_3)$_2$$Cl_2$] (**6**) (400.1 MHz, $CDCl_3$, 298K).

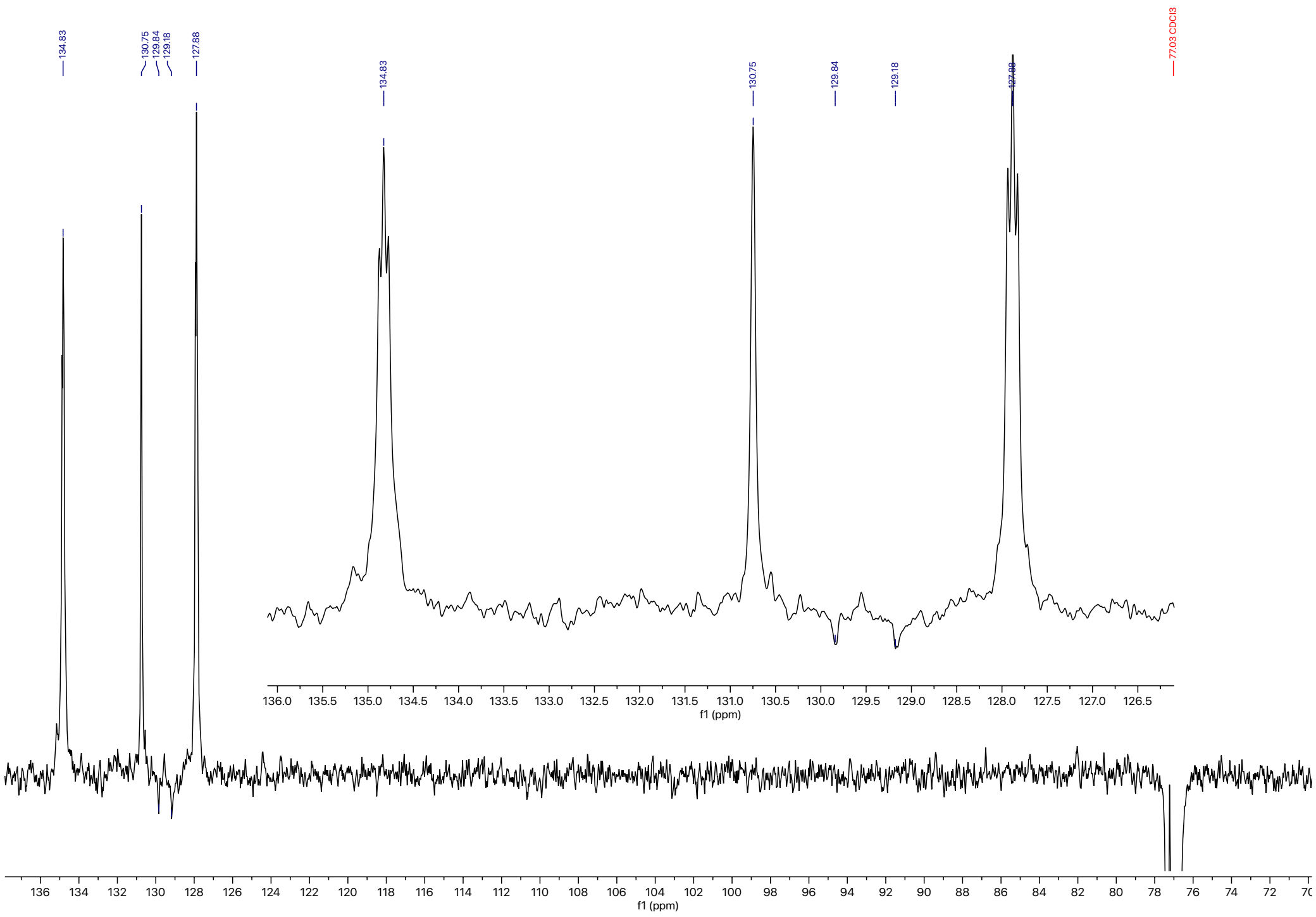

Figure S22. $^{13}C\{^{1}H\}$ NMR spectra of *cis*-[Pt(PPh_3)$_2$$Cl_2$] (**6**) (100.6 MHz, $CDCl_3$, 298K).

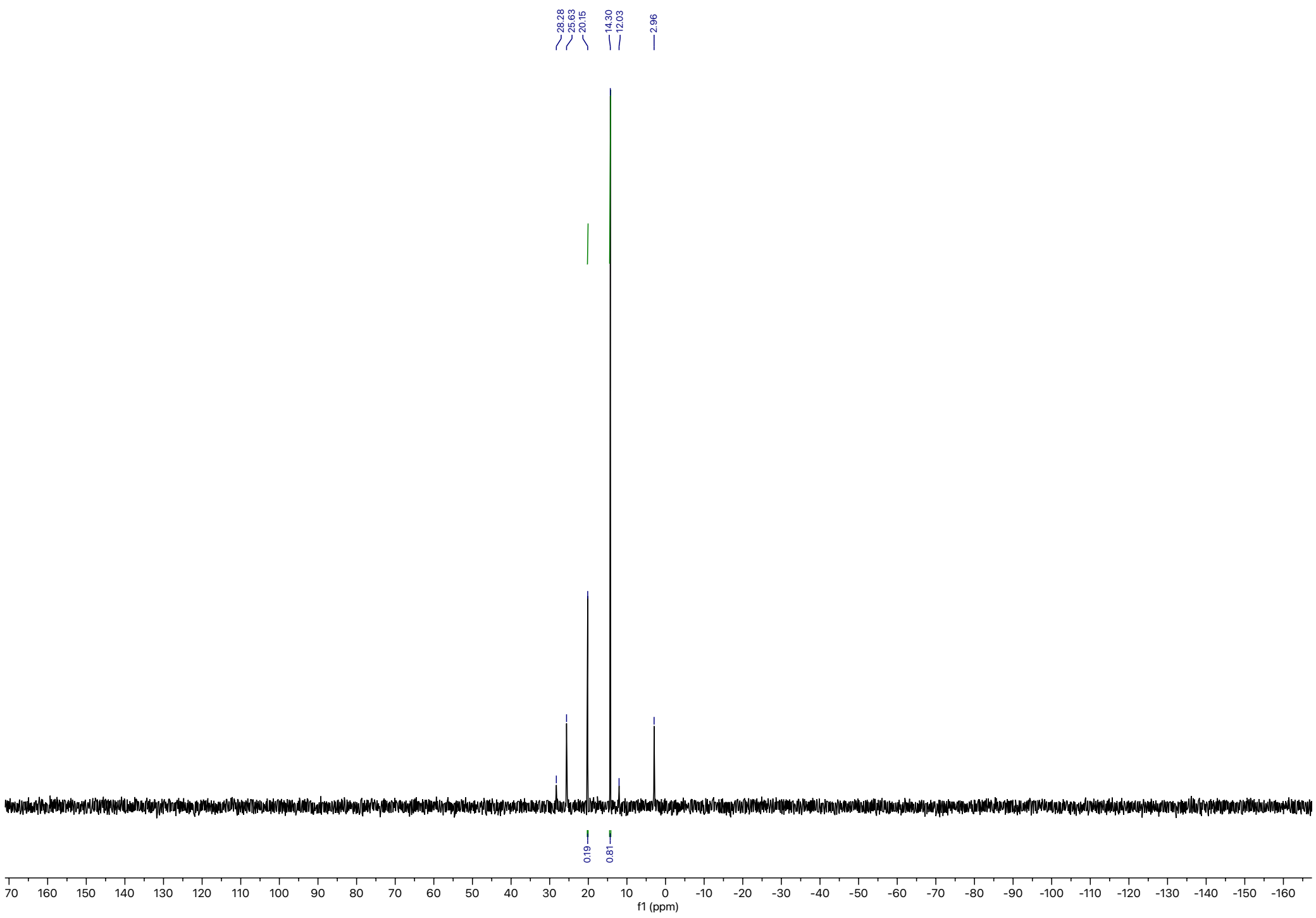

Figure S23. $^{31}P\{^{1}H\}$ NMR spectra of $[Pt(PPh_3)_2Cl_2]$ (**6**) (162.0 MHz, $CDCl_3$, 298K).

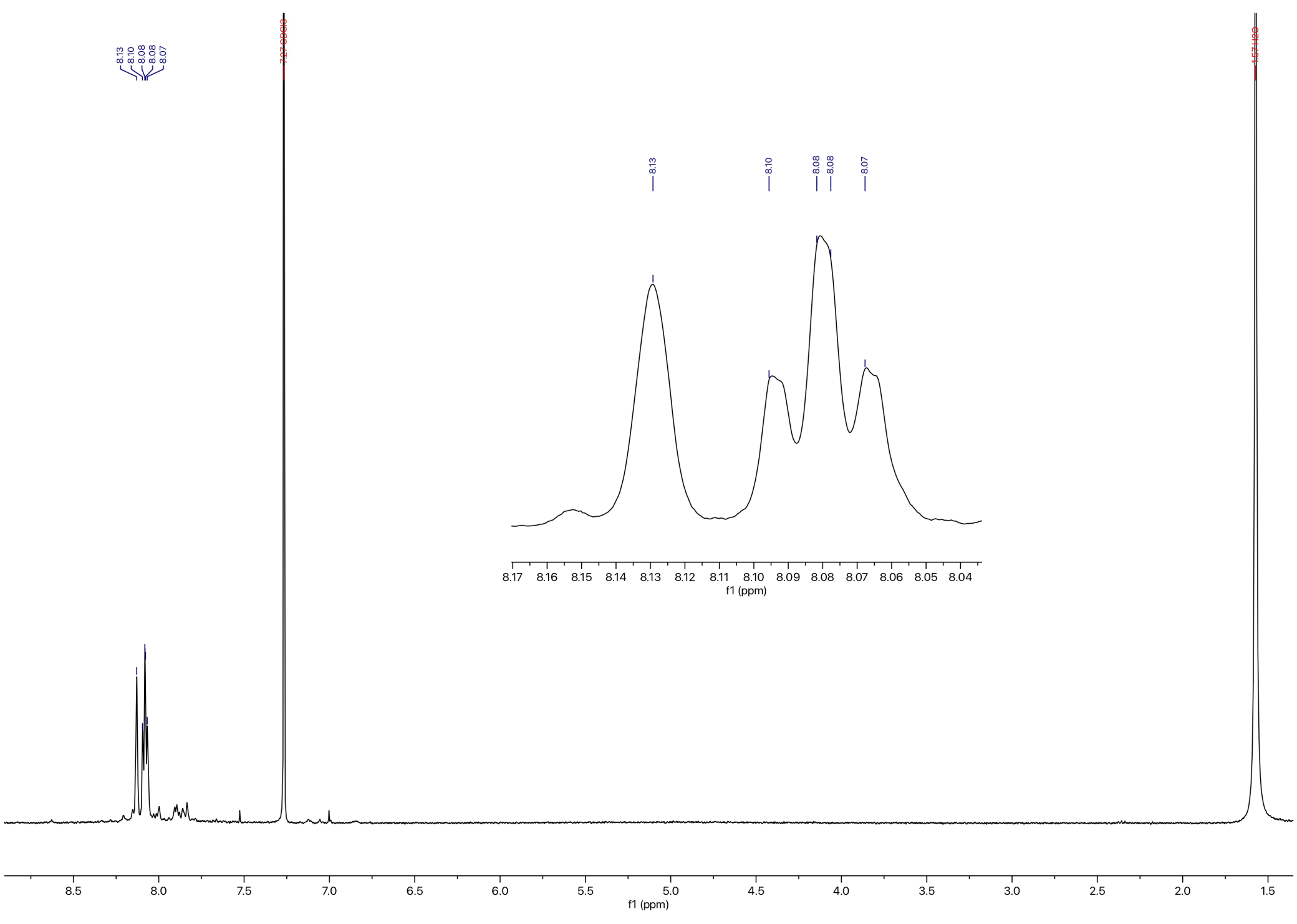

Figure S24. ^{1}H NMR spectra of $[Pt(P(3,5\text{-}(CF_3)_2Ph)_3)_2Cl_2]$ (**7**) (400.1 MHz, $CDCl_3$, 298K).

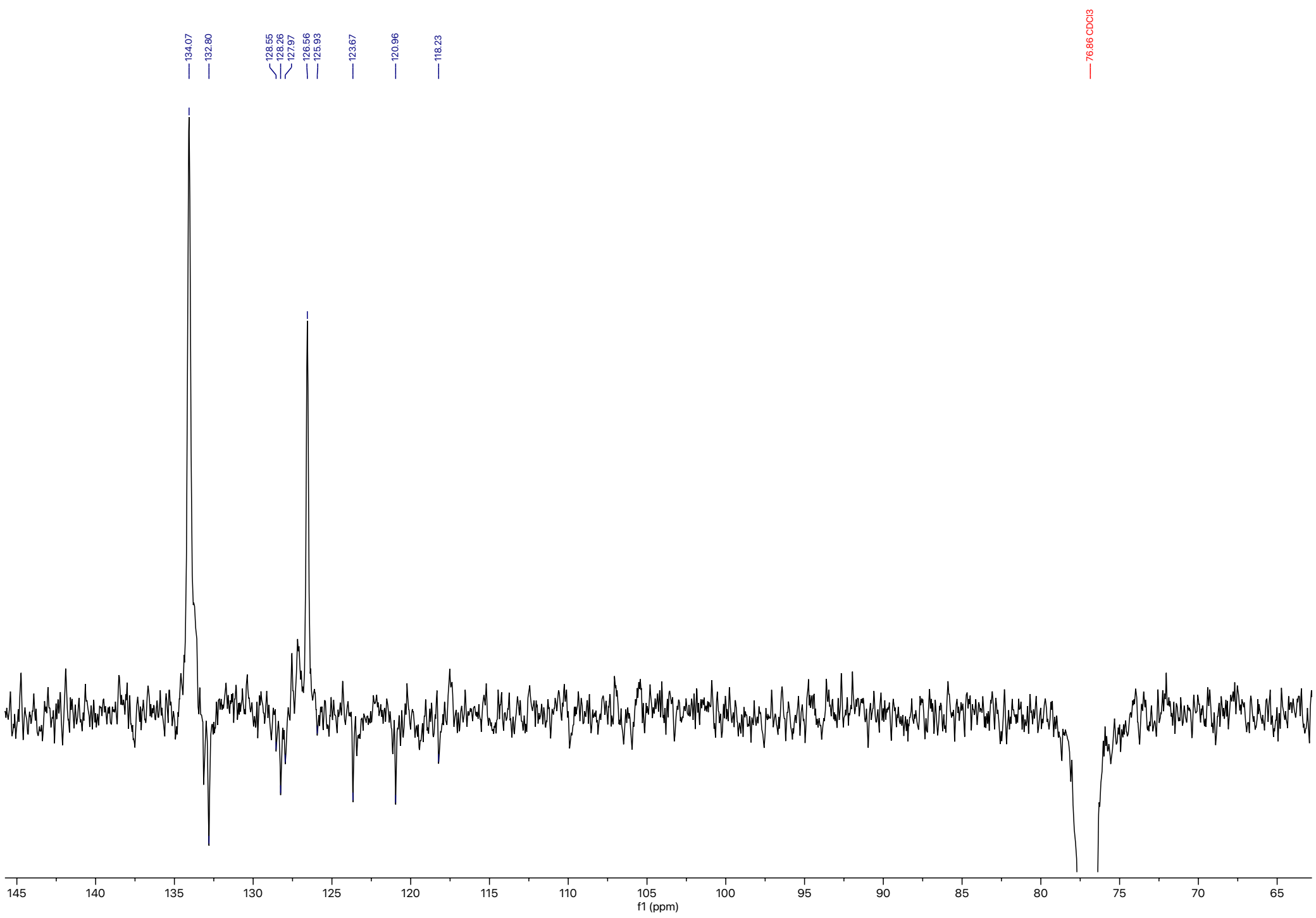

Figure S25. $^{13}C\{^1H\}$ NMR spectra of $[Pt(P(3,5-(CF_3)_2Ph)_3)_2Cl_2]$ (**7**) (100.6 MHz, $CDCl_3$, 298K).

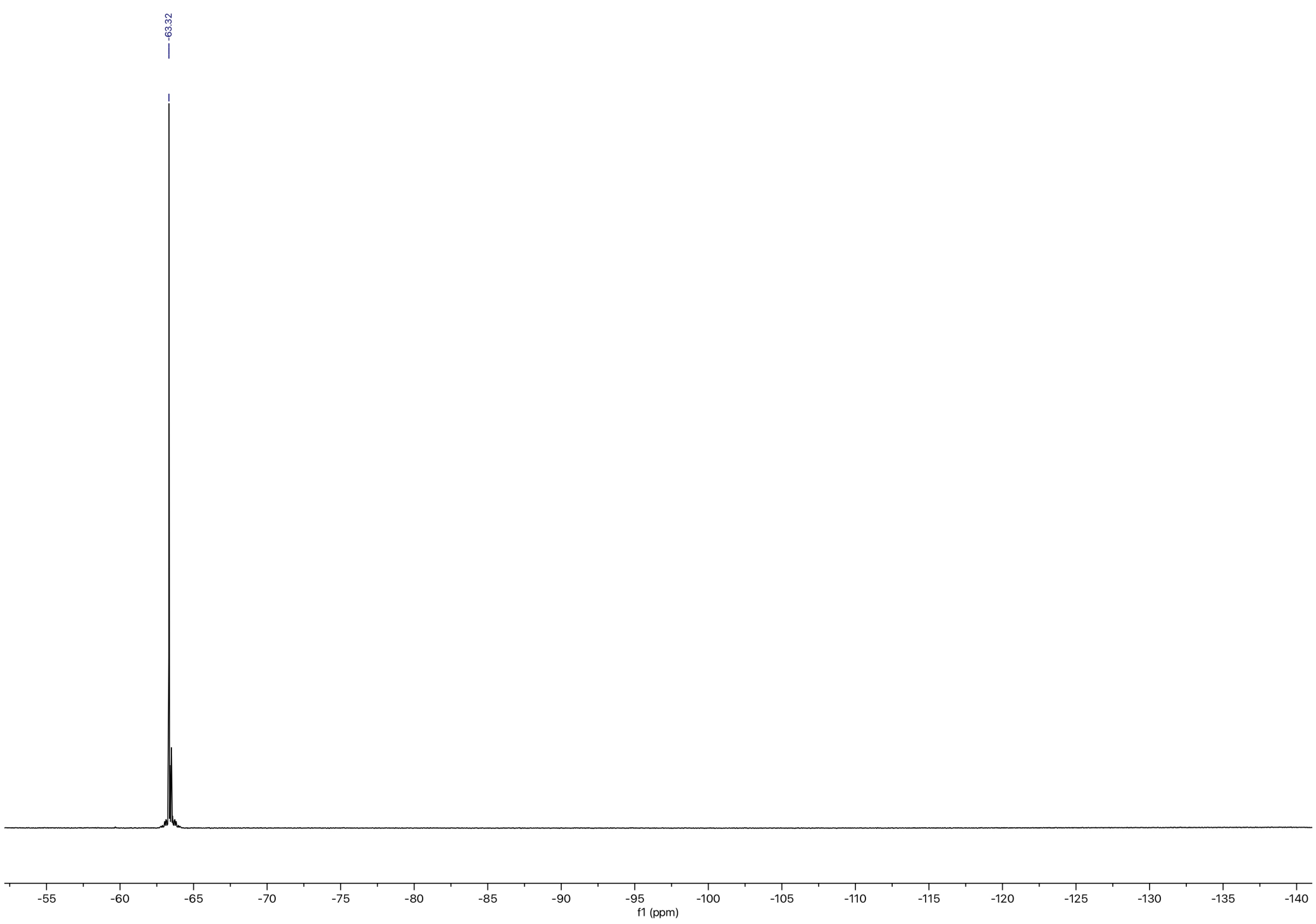

Figure S26. $^{19}F\{^1H\}$ NMR spectra of $[Pt(P(3,5-(CF_3)_2Ph)_3)_2Cl_2]$ (**7**) (376.9 MHz, $CDCl_3$, 298K).

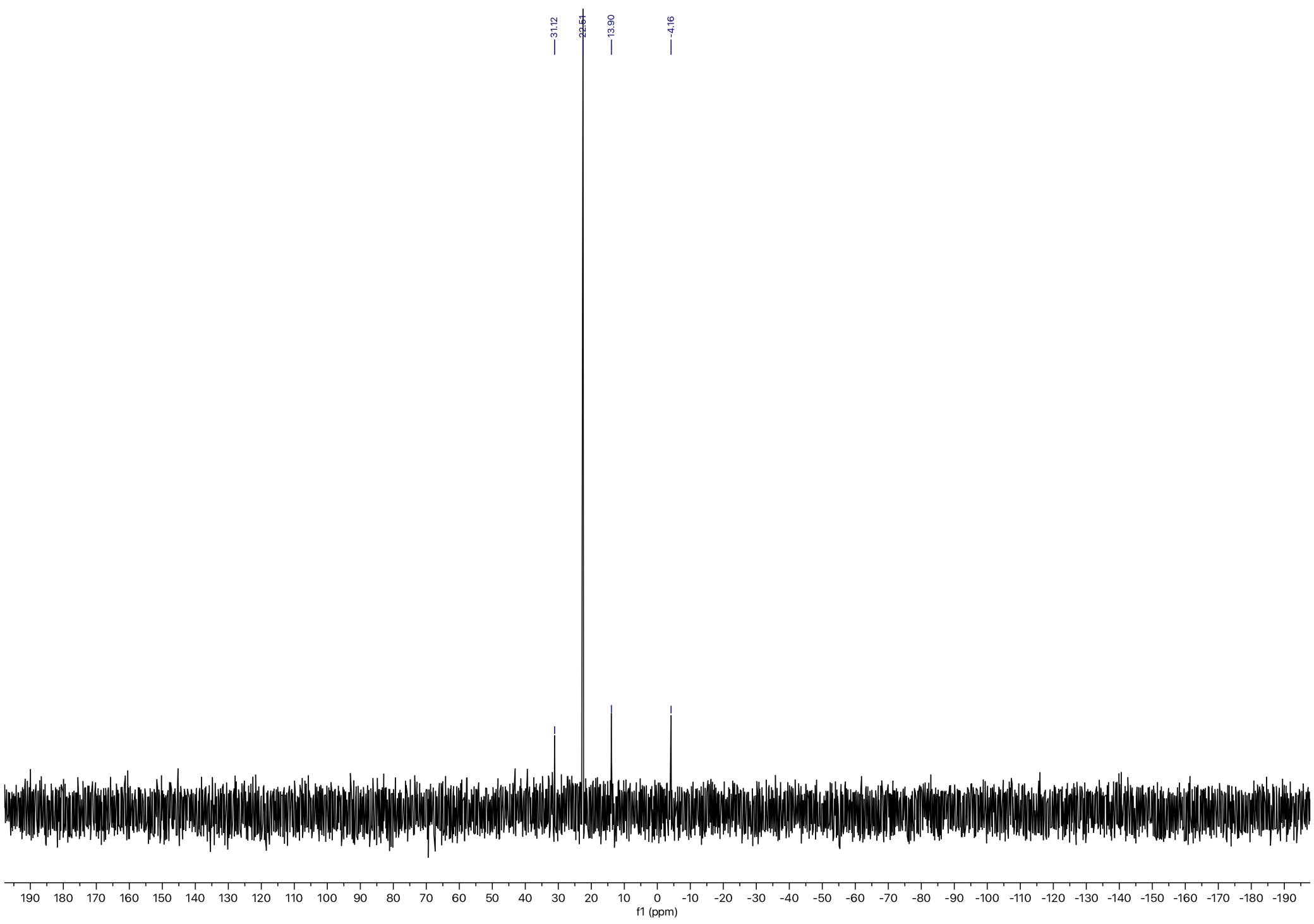

Figure S27. $^{31}P\{^{1}H\}$ NMR spectra of $[Pt(P(3,5\text{-}(CF_3)_2Ph)_3)_2Cl_2]$ (**7**) (162.0 MHz, $CDCl_3$, 298K).

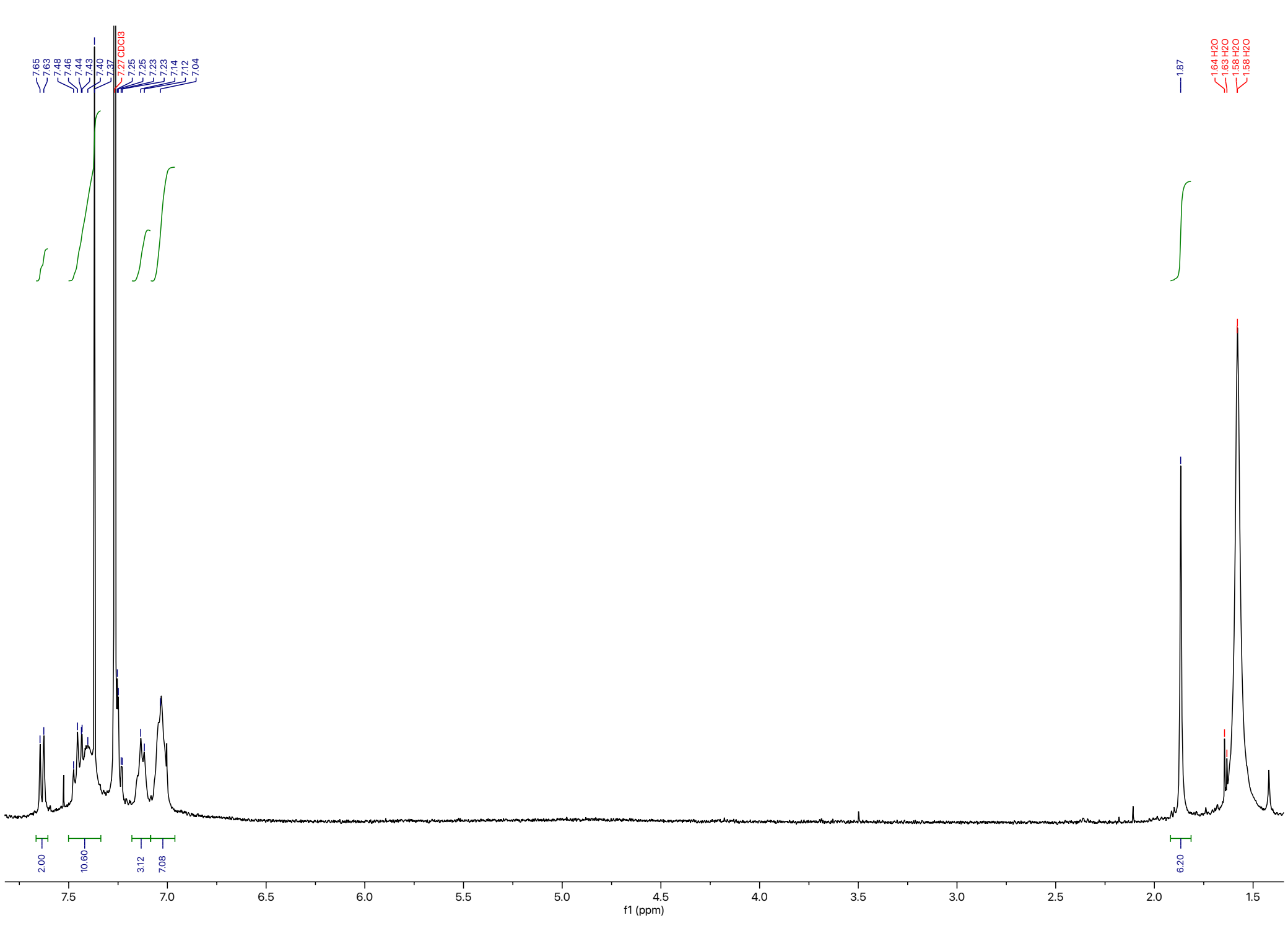

Figure S28. ^{1}H NMR spectra of $[Pt(Xantphos)Cl_2]$ (**8**) (400.1 MHz, $CDCl_3$, 298K).

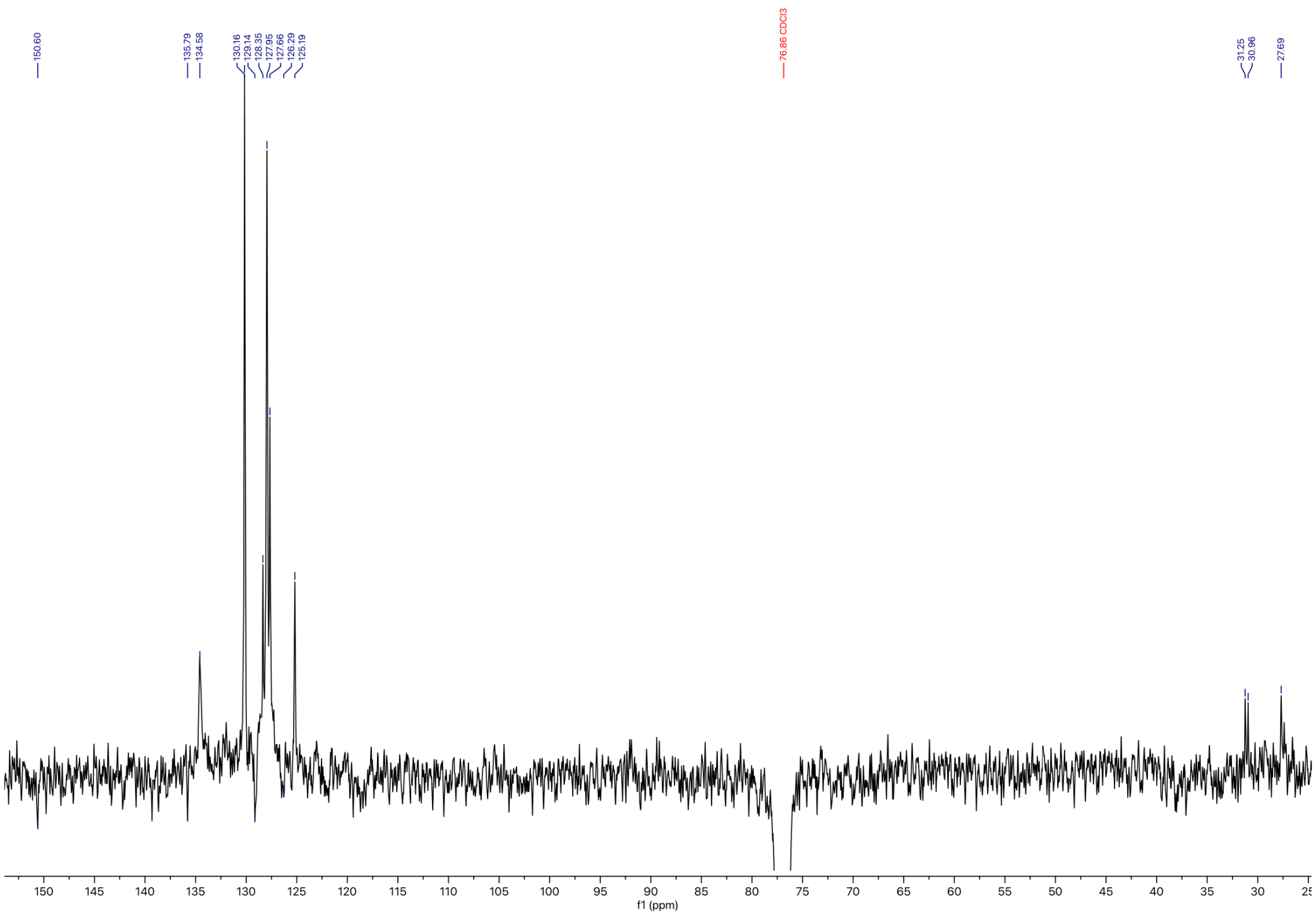

Figure S29. $^{13}C\{^{1}H\}$ NMR spectra of [Pt(Xantphos)Cl$_2$] (**8**) (100.6 MHz, $CDCl_3$, 298K).

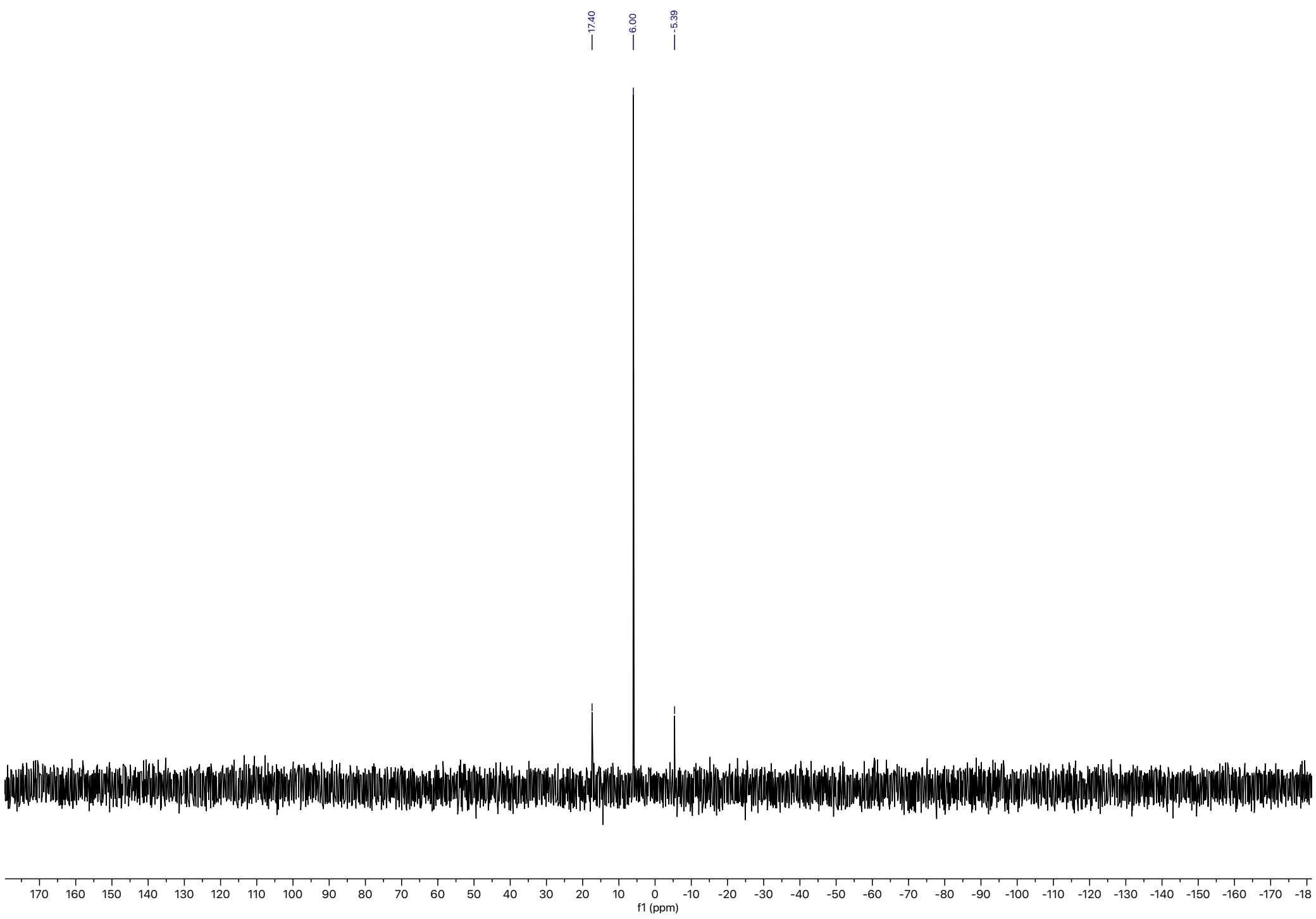

Figure S30. $^{31}P\{^{1}H\}$ NMR spectra of [Pt(Xantphos)Cl$_2$] (**8**) (162.0 MHz, $CDCl_3$, 298K).

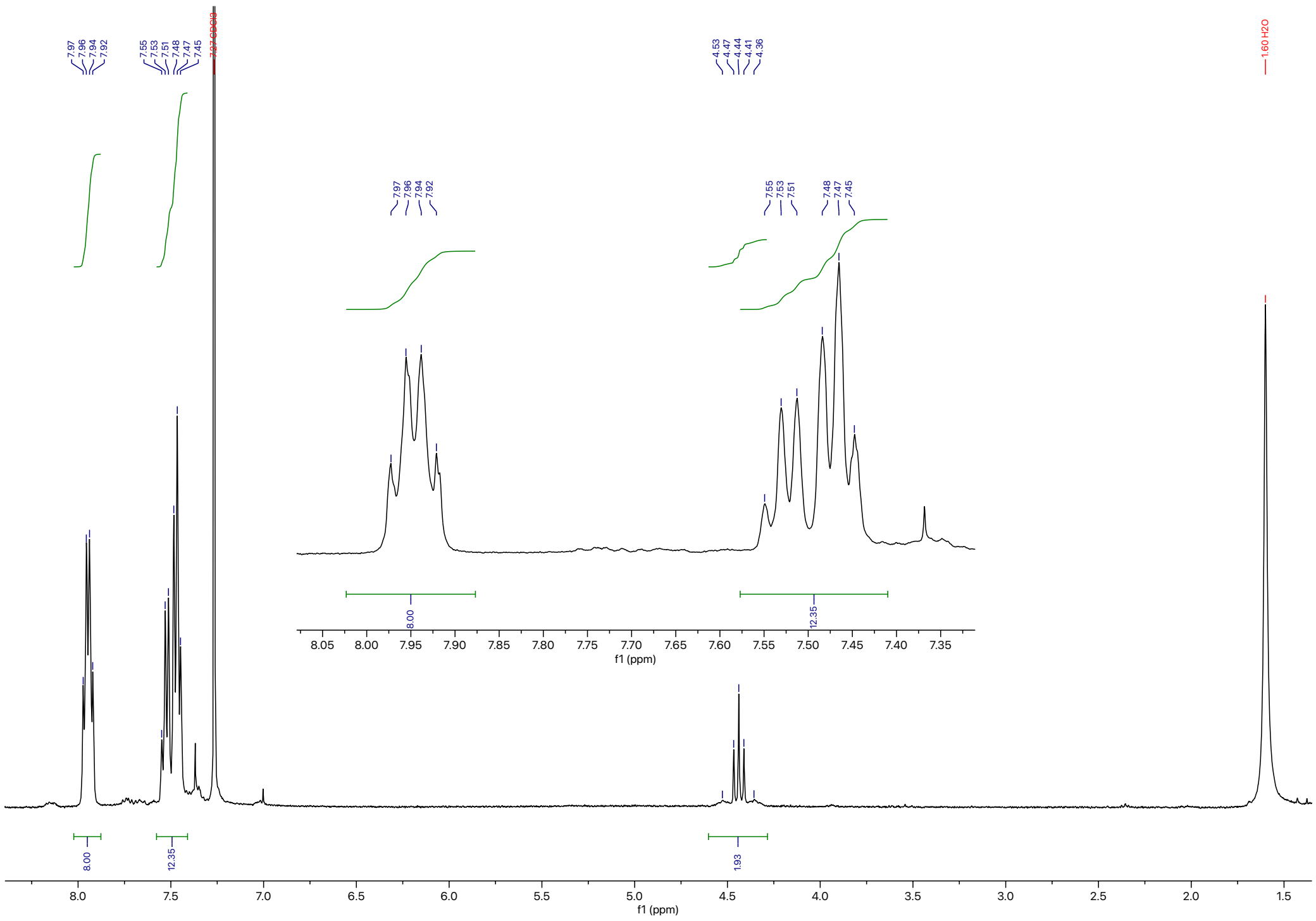

Figure S31. ^{1}H NMR spectra of [Pt(dppm)Cl_2] (**9**) (400.1 MHz, $CDCl_3$, 298K).

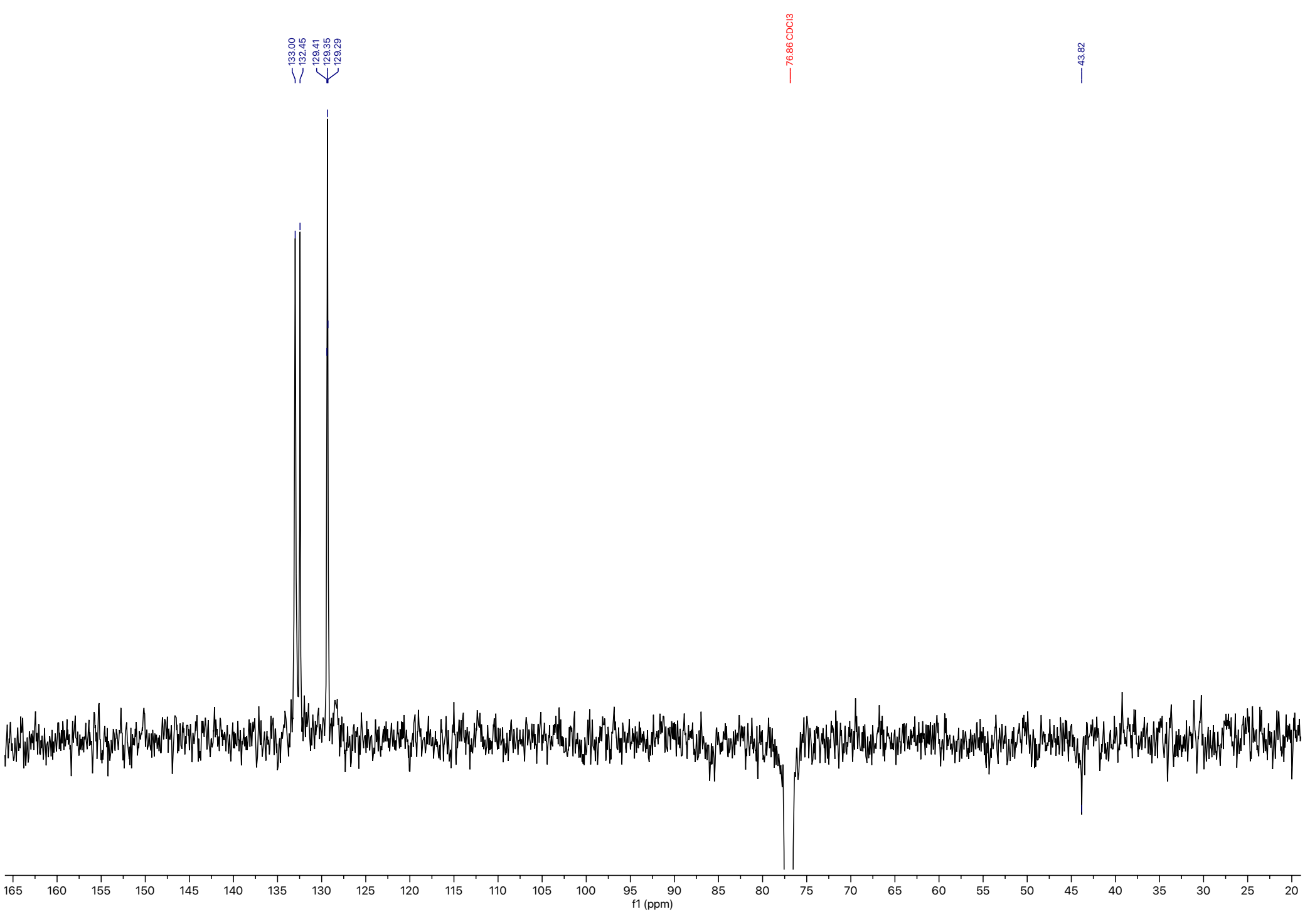

Figure S32. $^{13}C\{^{1}H\}$ NMR spectra of [Pt(dppm)Cl_2] (**9**) (100.6 MHz, $CDCl_3$, 298K).

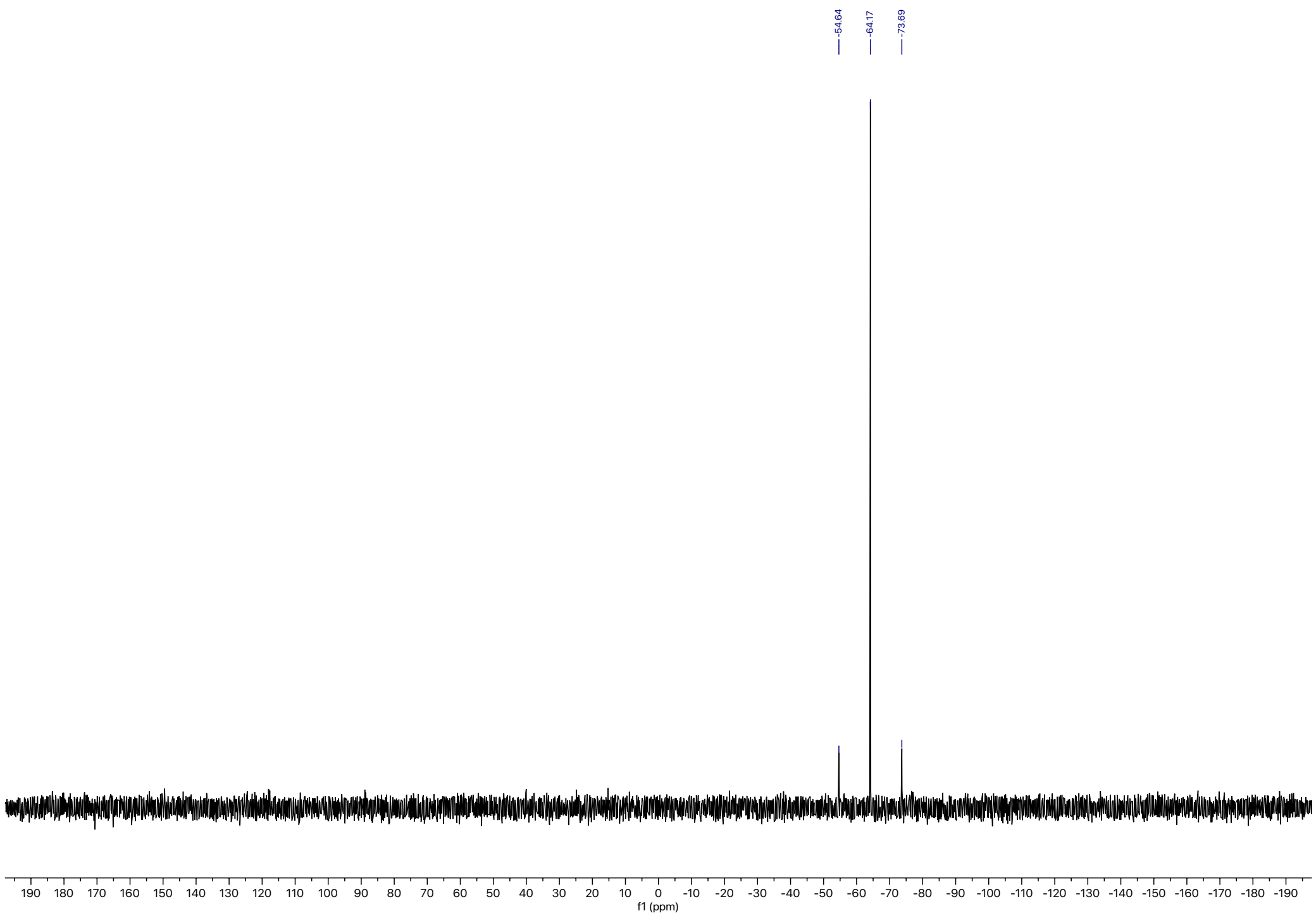

Figure S33. $^{31}P\{^{1}H\}$ NMR spectra of [Pt(dppm)Cl_2] (**9**) (162.0 MHz, $CDCl_3$, 298K).

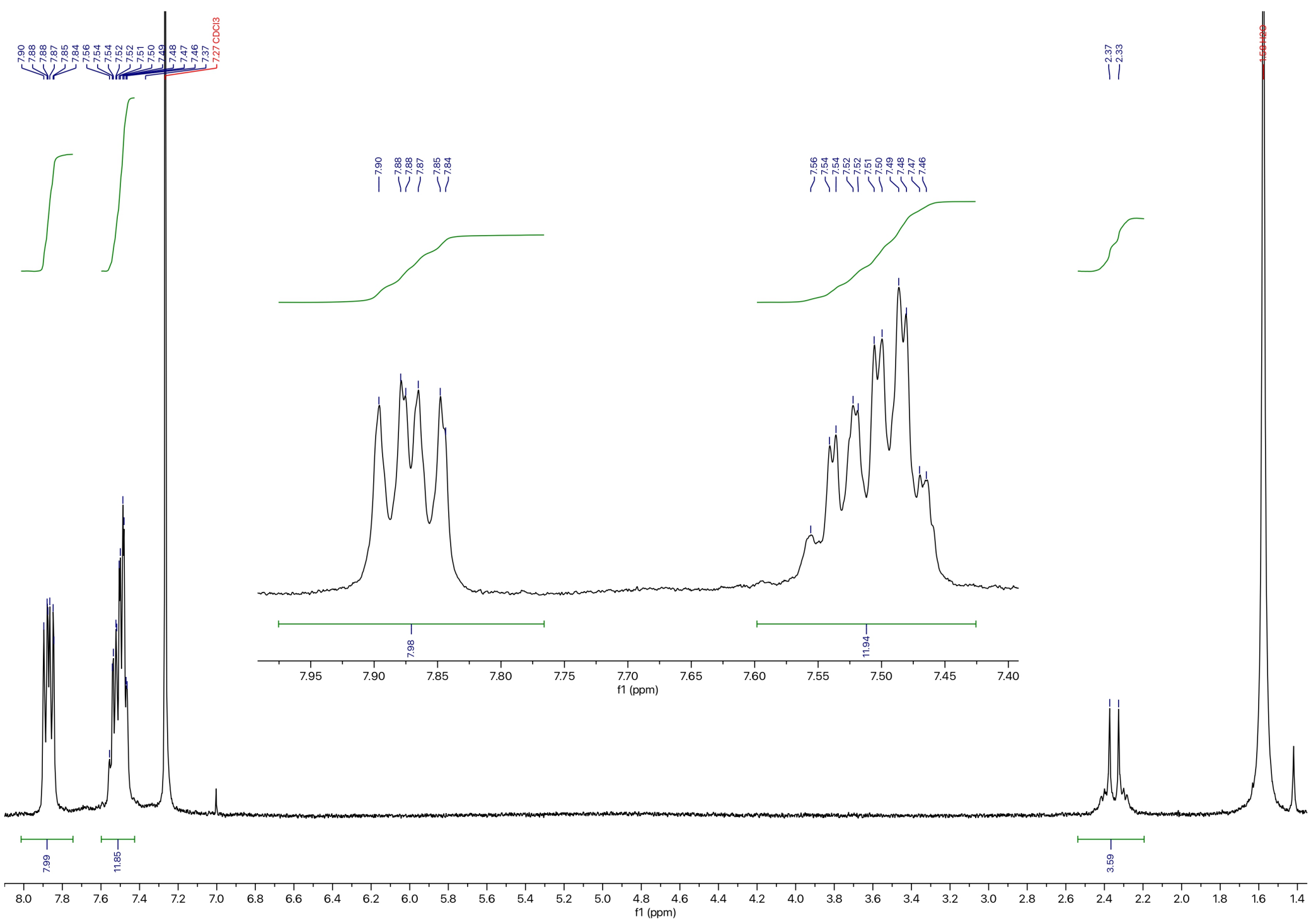

Figure S34. ^{1}H NMR spectra of [Pt(dppe)Cl_2] (**10**) (400.1 MHz, $CDCl_3$, 298K).

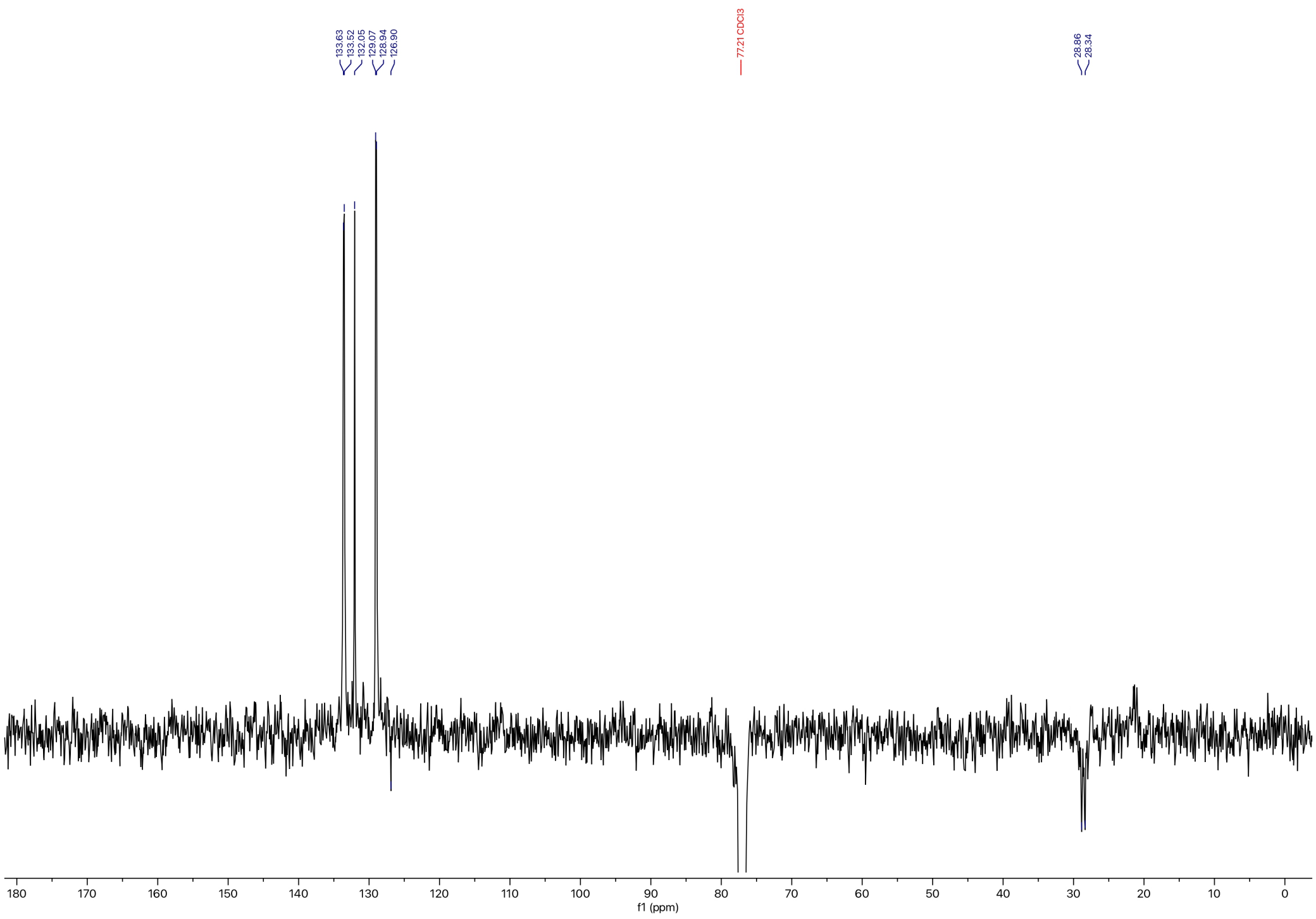

Figure S35. $^{13}C\{^1H\}$ NMR spectra of [Pt(dppe)Cl$_2$] (**10**) (100.6 MHz, $CDCl_3$, 298K).

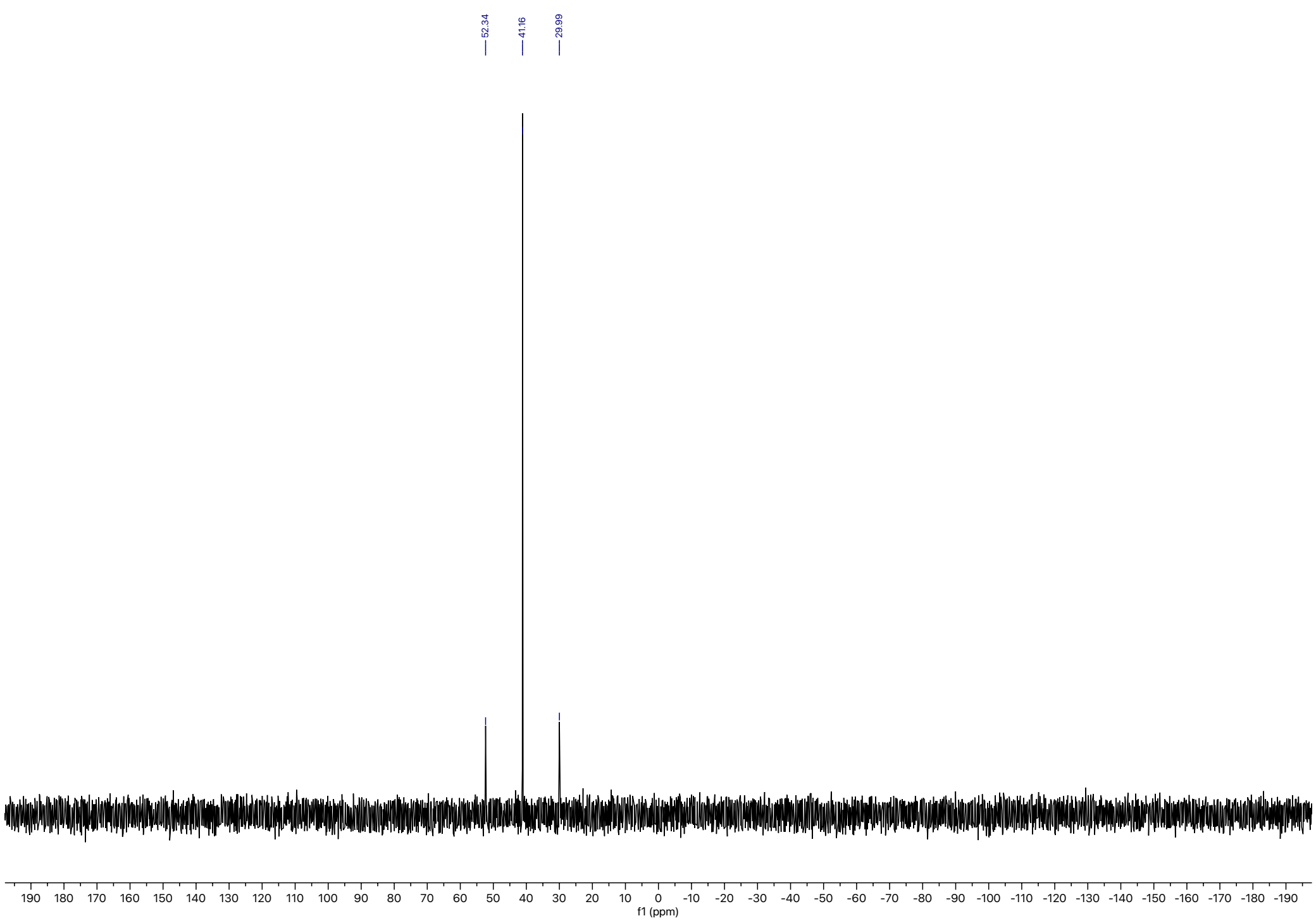

Figure S36. $^{31}P\{^1H\}$ NMR spectra of [Pt(dppe)Cl$_2$] (**10**) (162.0 MHz, $CDCl_3$, 298K).

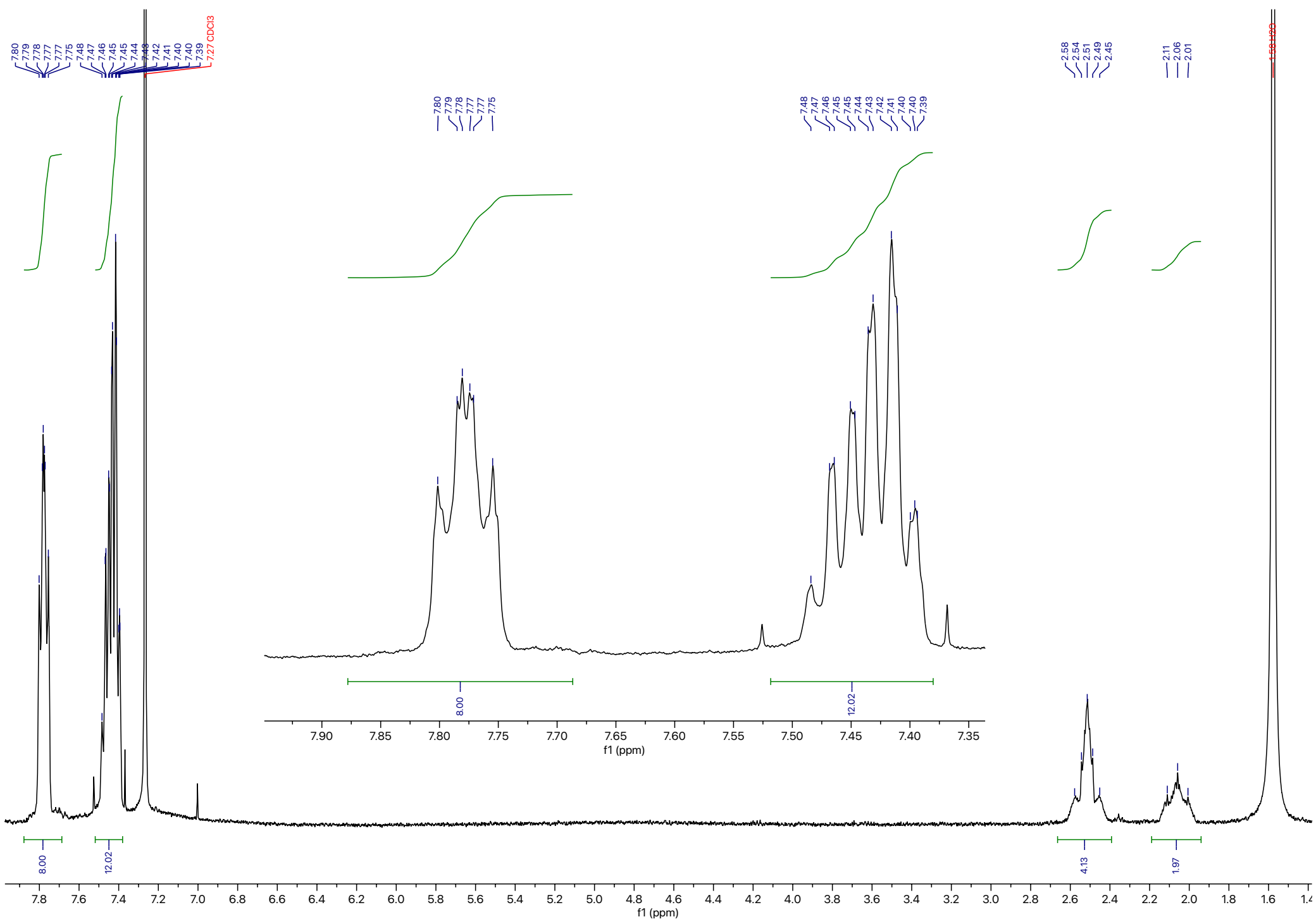

Figure S37. ^{1}H NMR spectra of [Pt(dppp)Cl_2] (**11**) (400.1 MHz, $CDCl_3$, 298K).

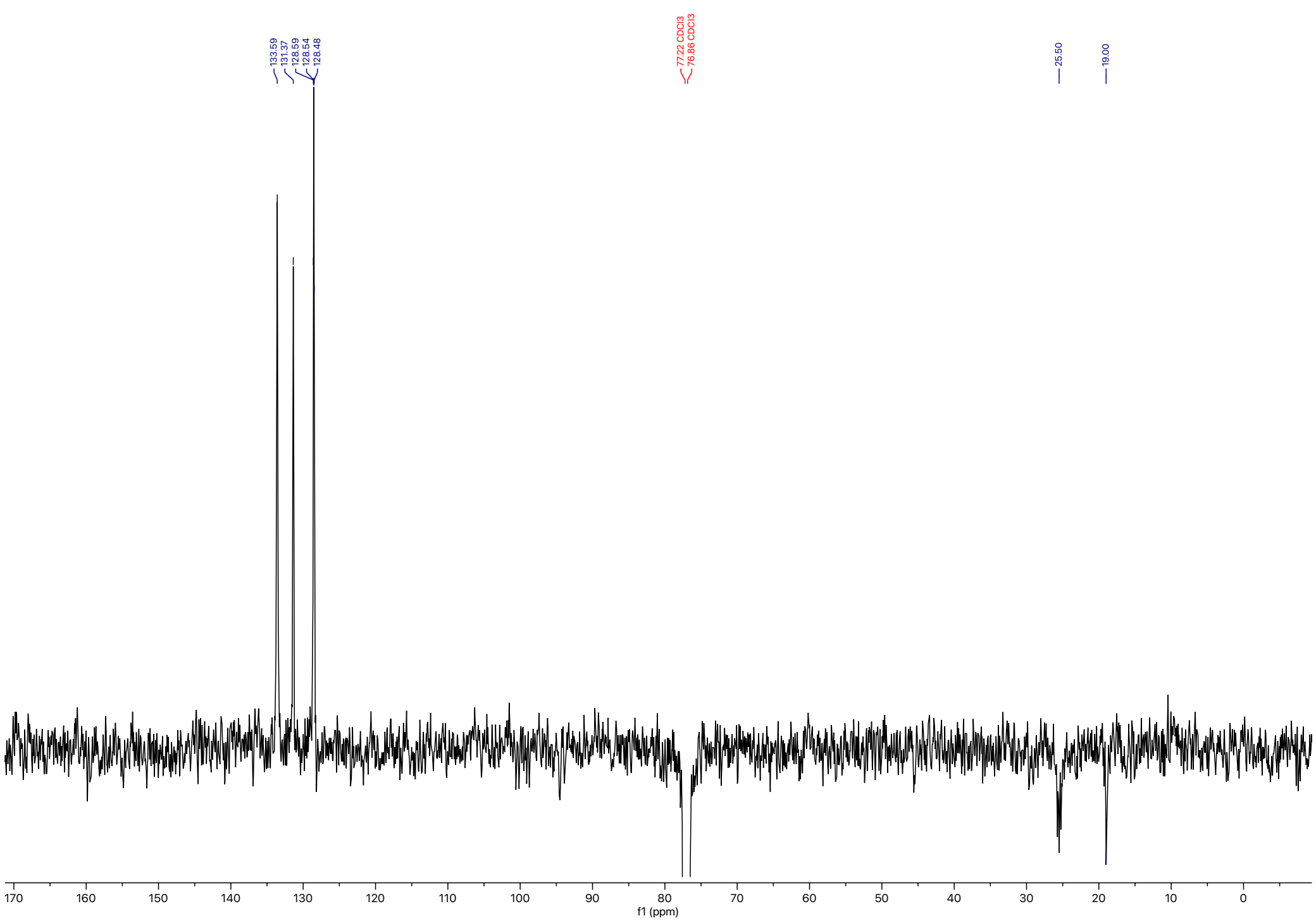

Figure S38. $^{13}C\{^{1}H\}$ NMR spectra of [Pt(dppp)Cl_2] (**11**) (100.6 MHz, $CDCl_3$, 298K).

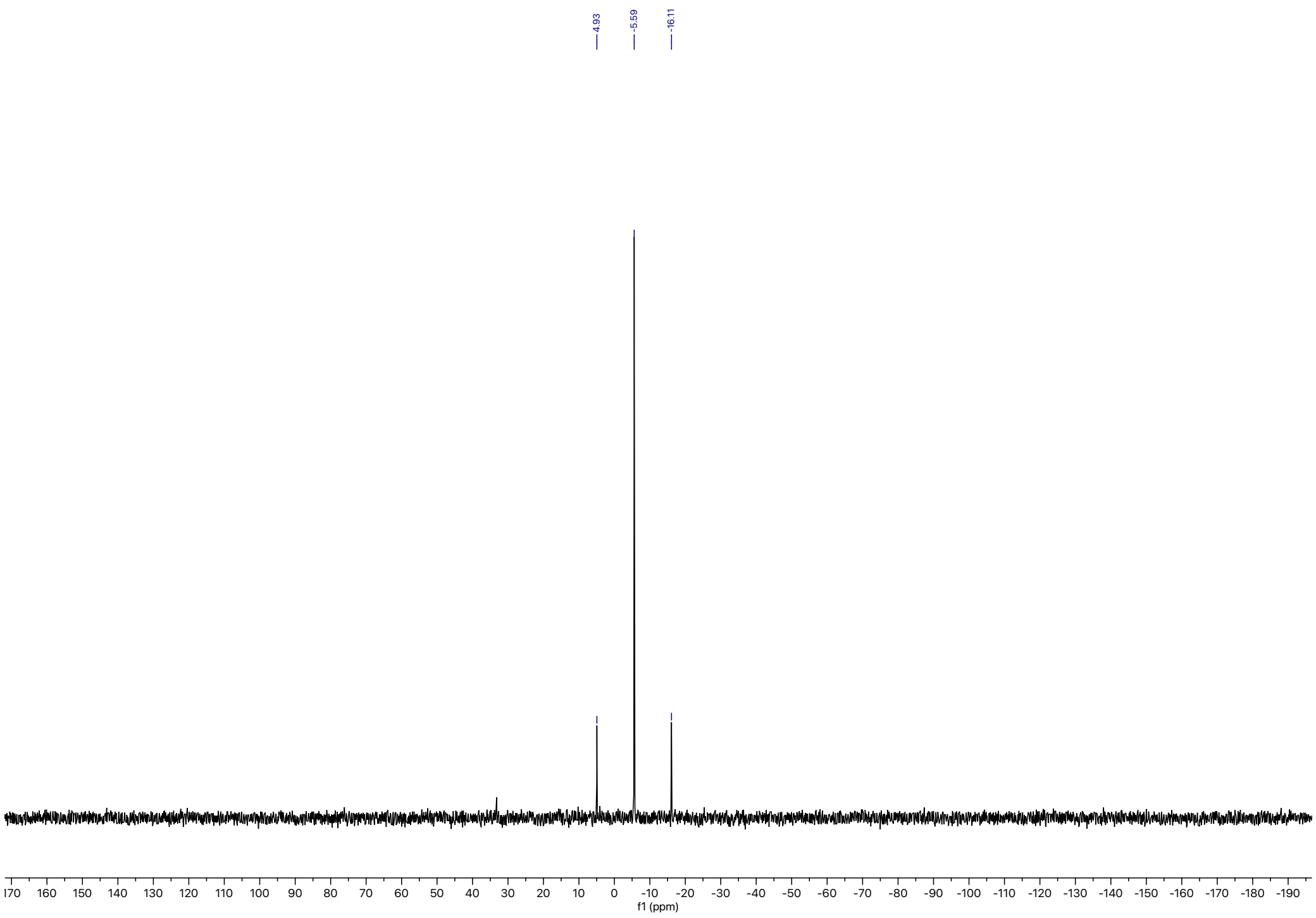

Figure S39. $^{31}P\{^{1}H\}$ NMR spectra of [Pt(dppp)Cl_2] (**11**) (162.0 MHz, $CDCl_3$, 298K).

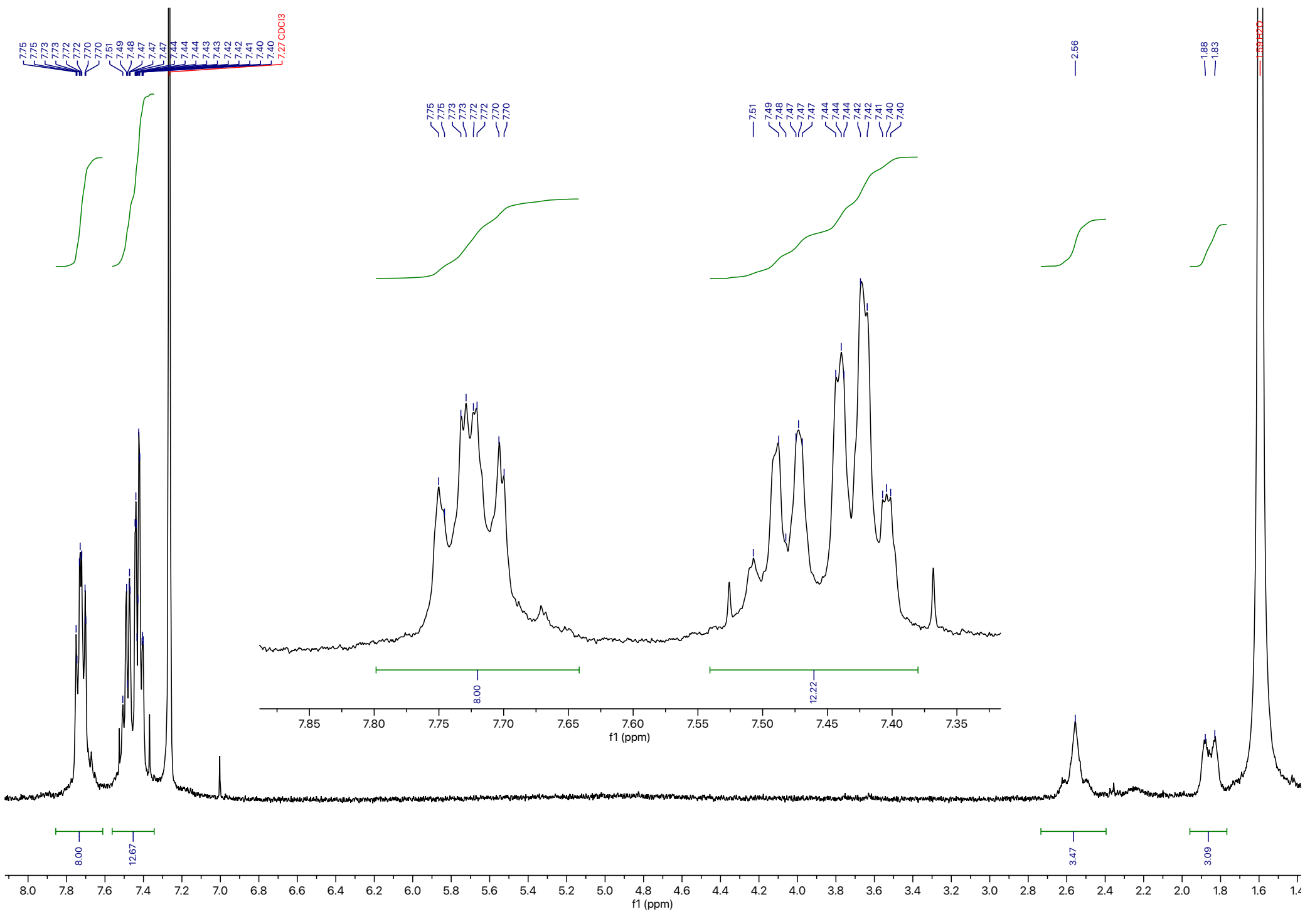

Figure S40. ^{1}H NMR spectra of [Pt(dppb)Cl_2] (**12**) (400.1 MHz, $CDCl_3$, 298K).

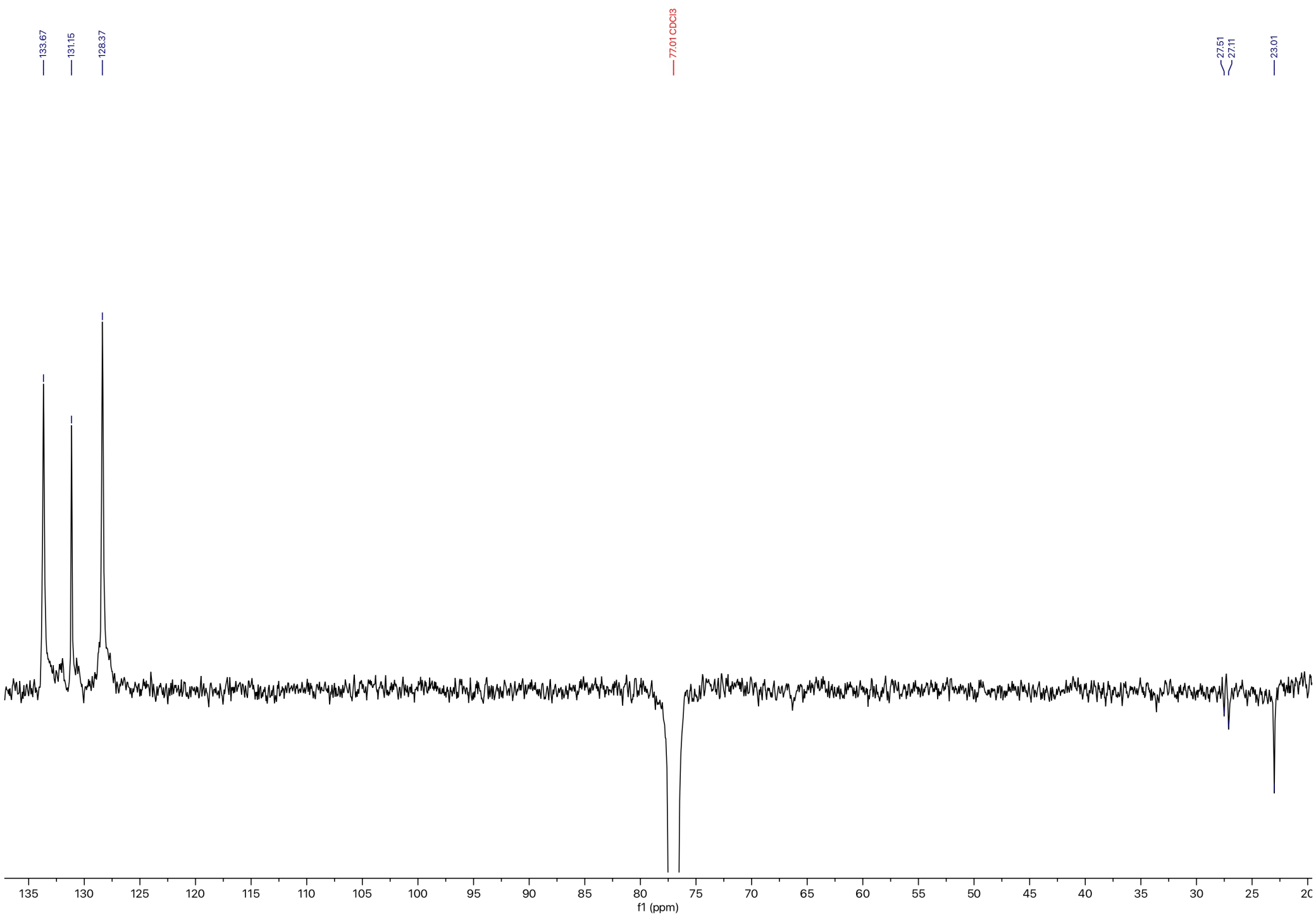

Figure S41. $^{13}C\{^1H\}$ NMR spectra of [Pt(dppb)Cl$_2$] (**12**) (100.6 MHz, $CDCl_3$, 298K).

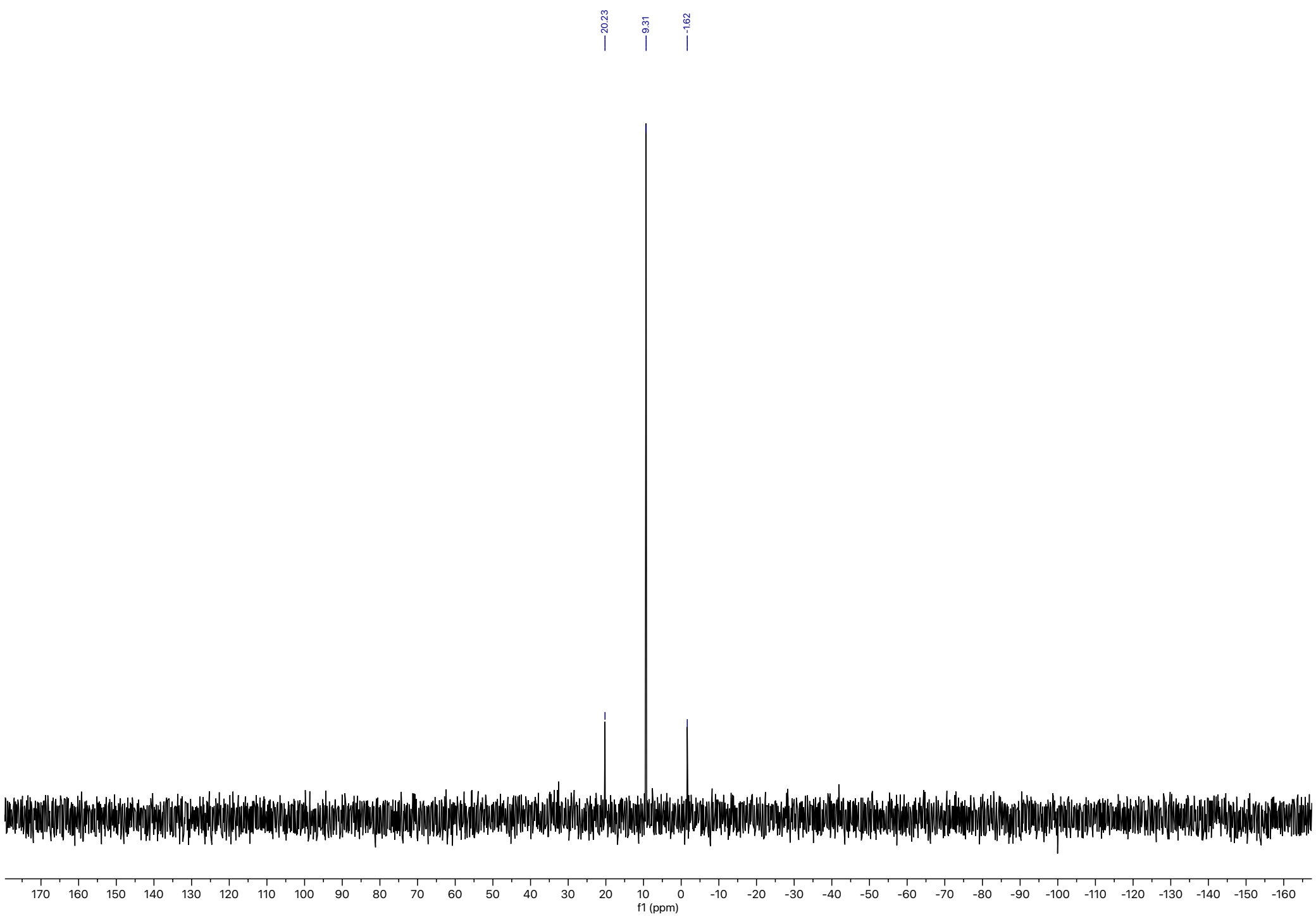

Figure S42. $^{31}P\{^1H\}$ NMR spectra of [Pt(dppb)Cl$_2$] (**12**) (162.0 MHz, $CDCl_3$, 298K).

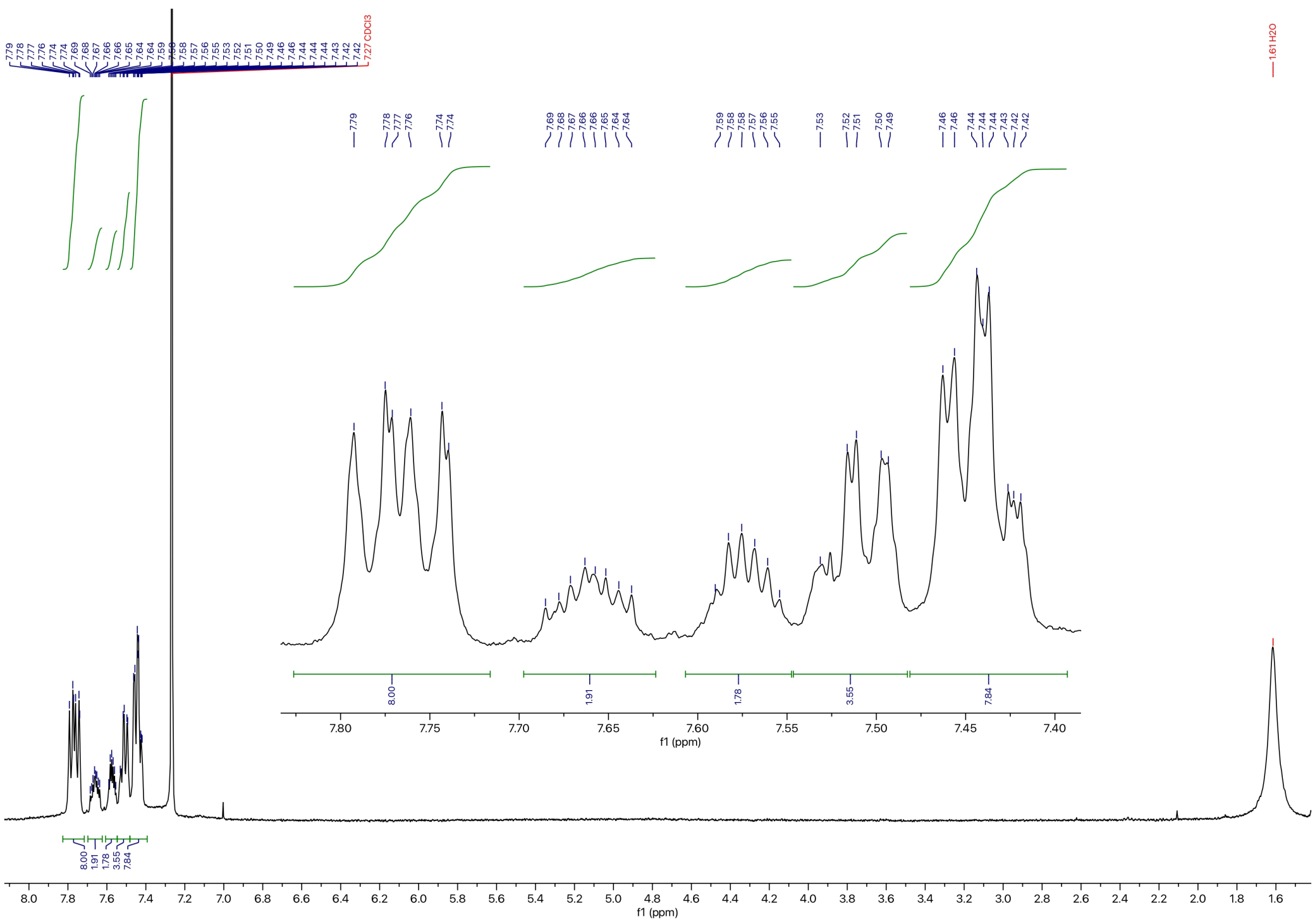

Figure S43. ^{1}H NMR spectra of [Pt(dppbz)Cl_2] (**13**) (400.1 MHz, $CDCl_3$, 298K).

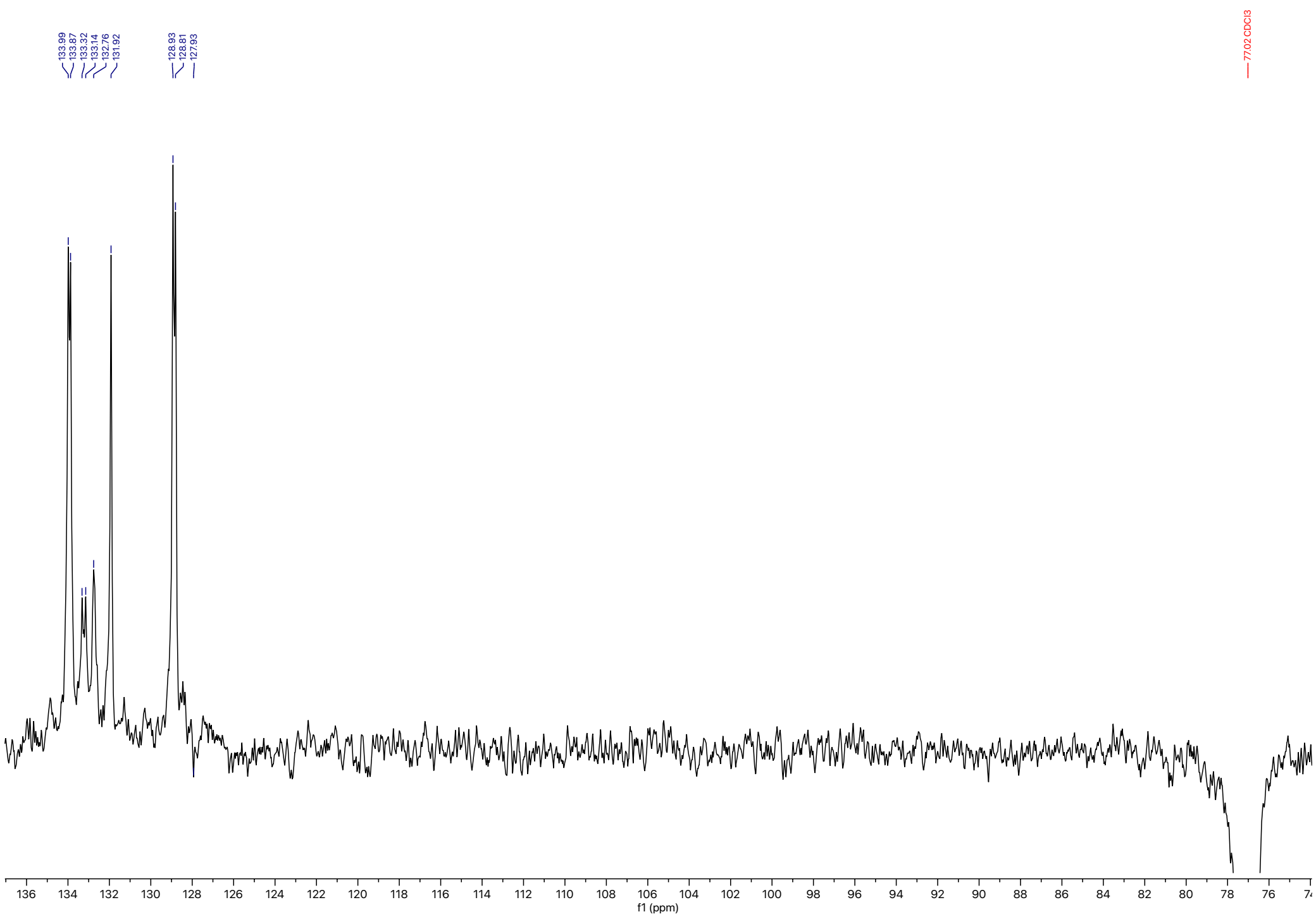

Figure S44. ^{13}C{^{1}H} NMR spectra of [Pt(dppbz)Cl_2] (**13**) (100.6 MHz, $CDCl_3$, 298K).

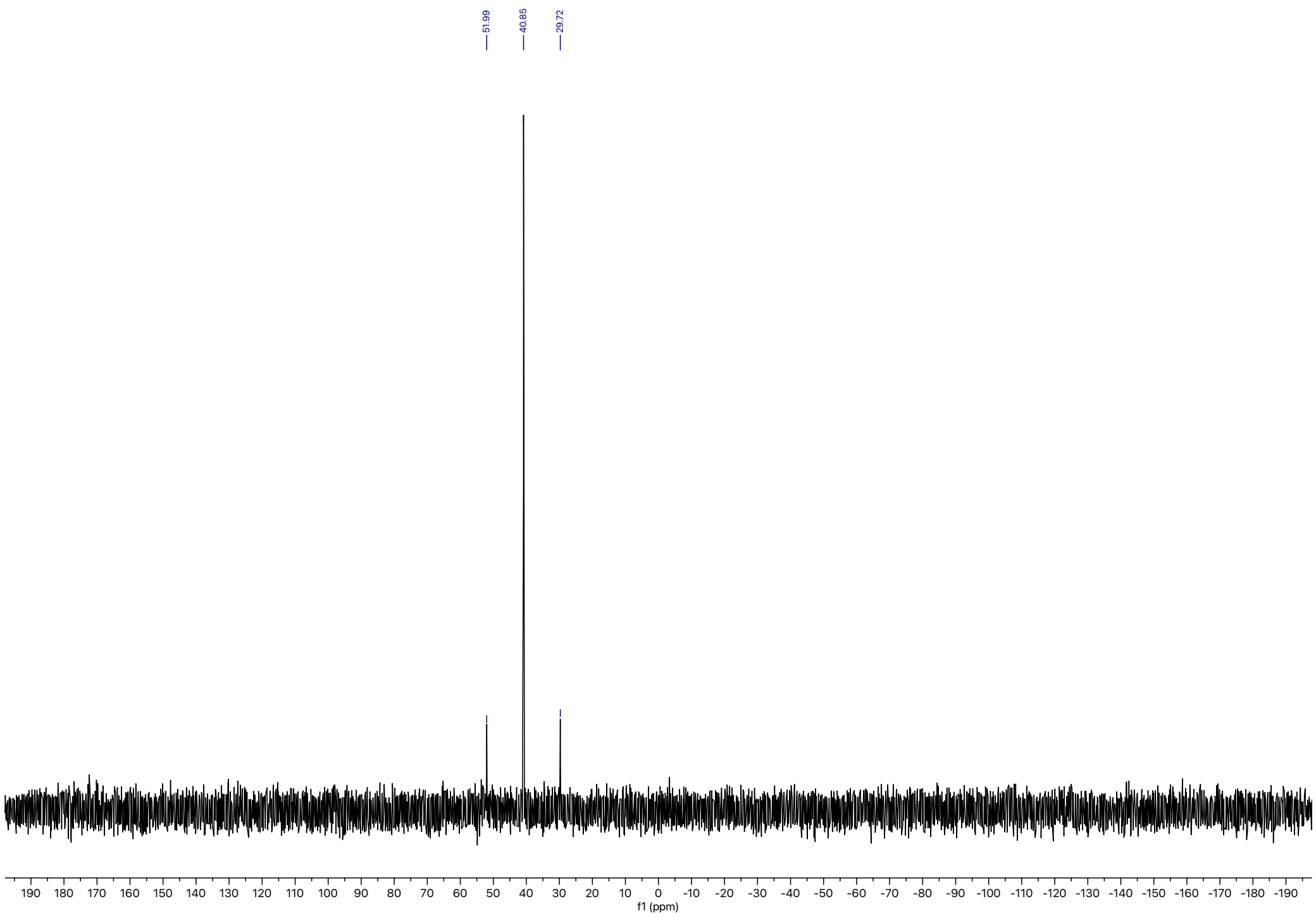

Figure S45. $^{31}P\{^{1}H\}$ NMR spectra of [Pt(dppbz)Cl$_2$] (**13**) (162.0 MHz, $CDCl_3$, 298K).

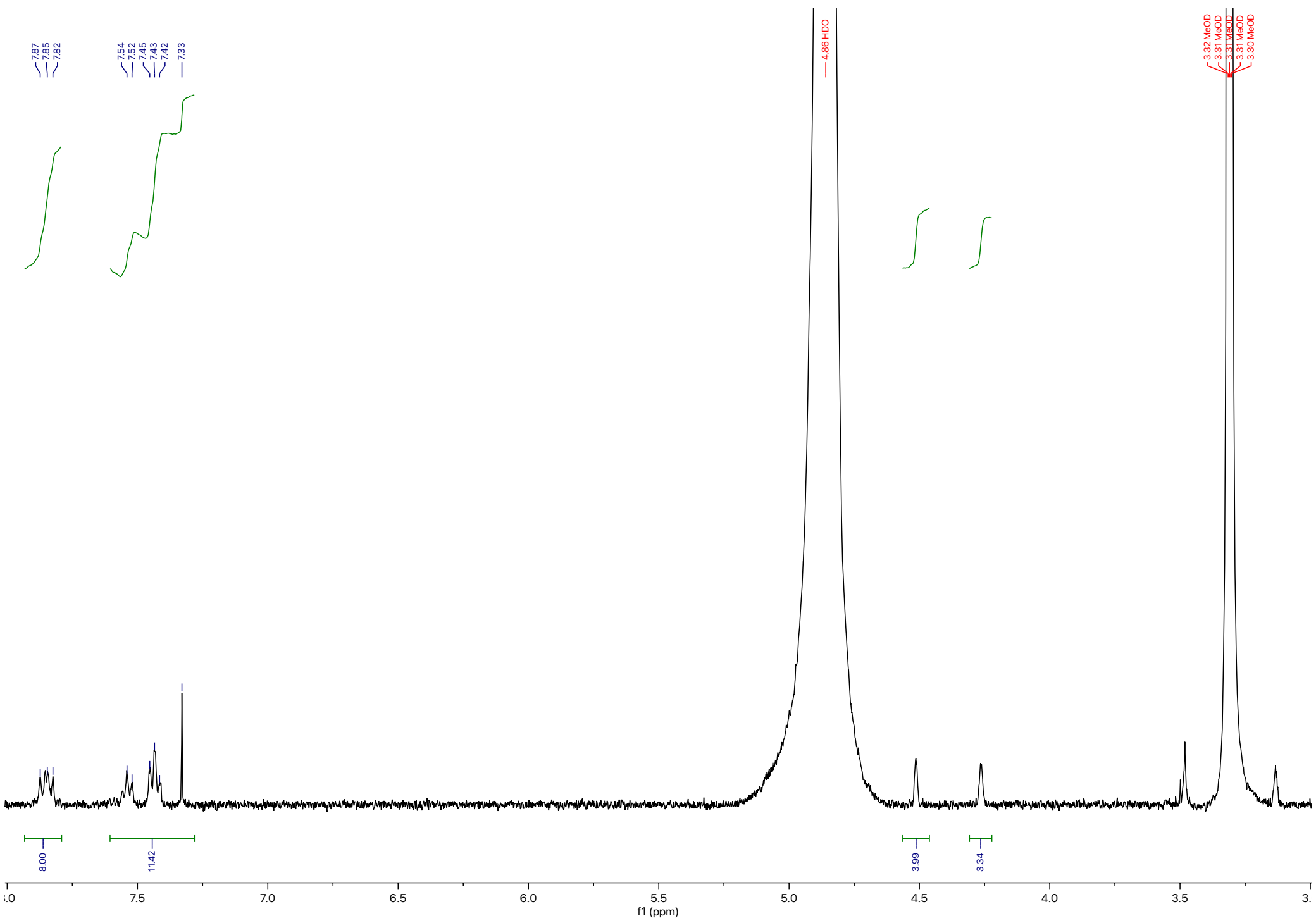

Figure S46. ^{1}H NMR spectra of [Pt(dppf)Cl$_2$] (**14**) (400.1 MHz, CD_3OD, 298K).

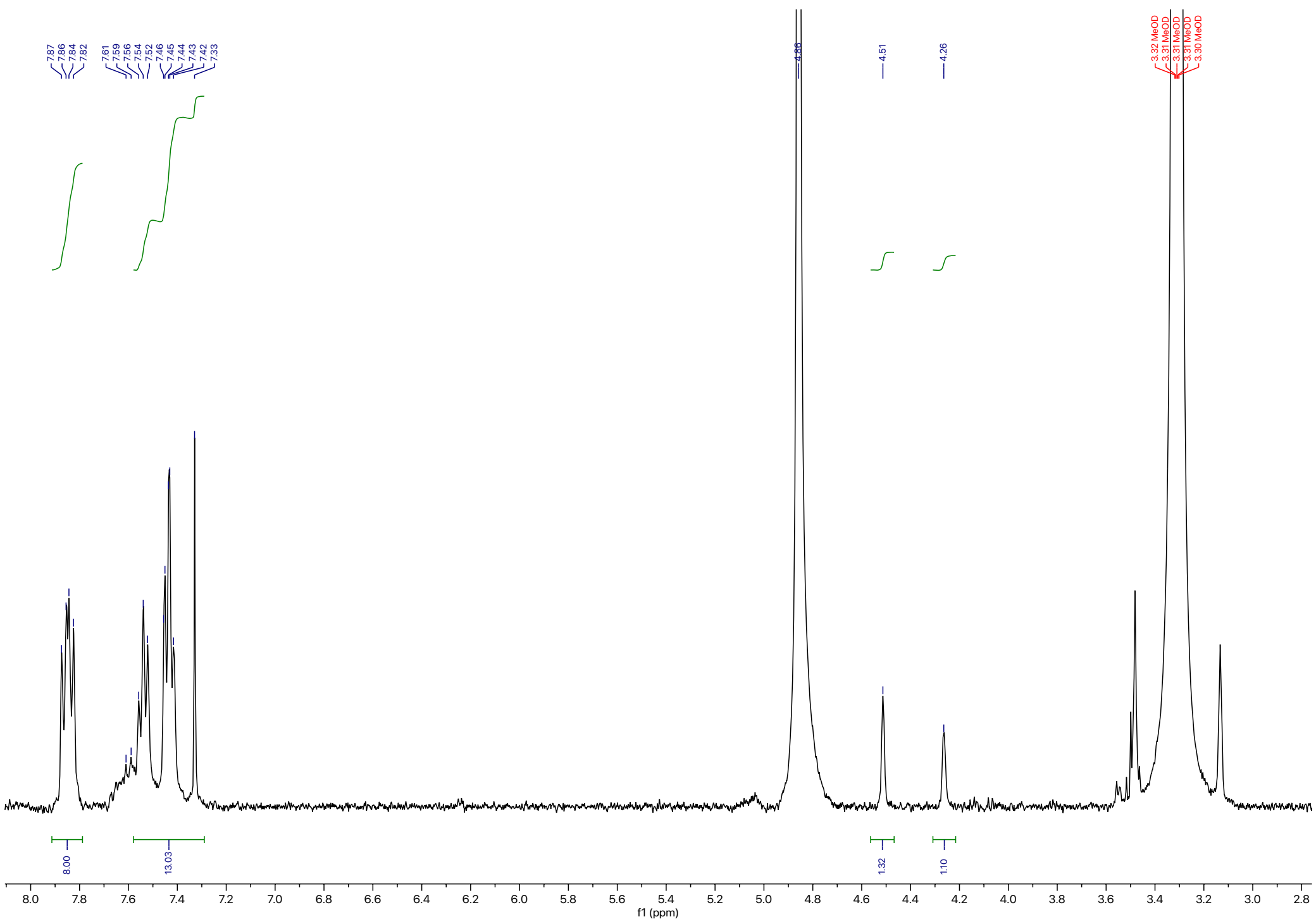

Figure S47. ^{1}H NMR water suppression spectra of [Pt(dppf)Cl_2] (**14**) (400.1 MHz, CD_3OD, 298K).

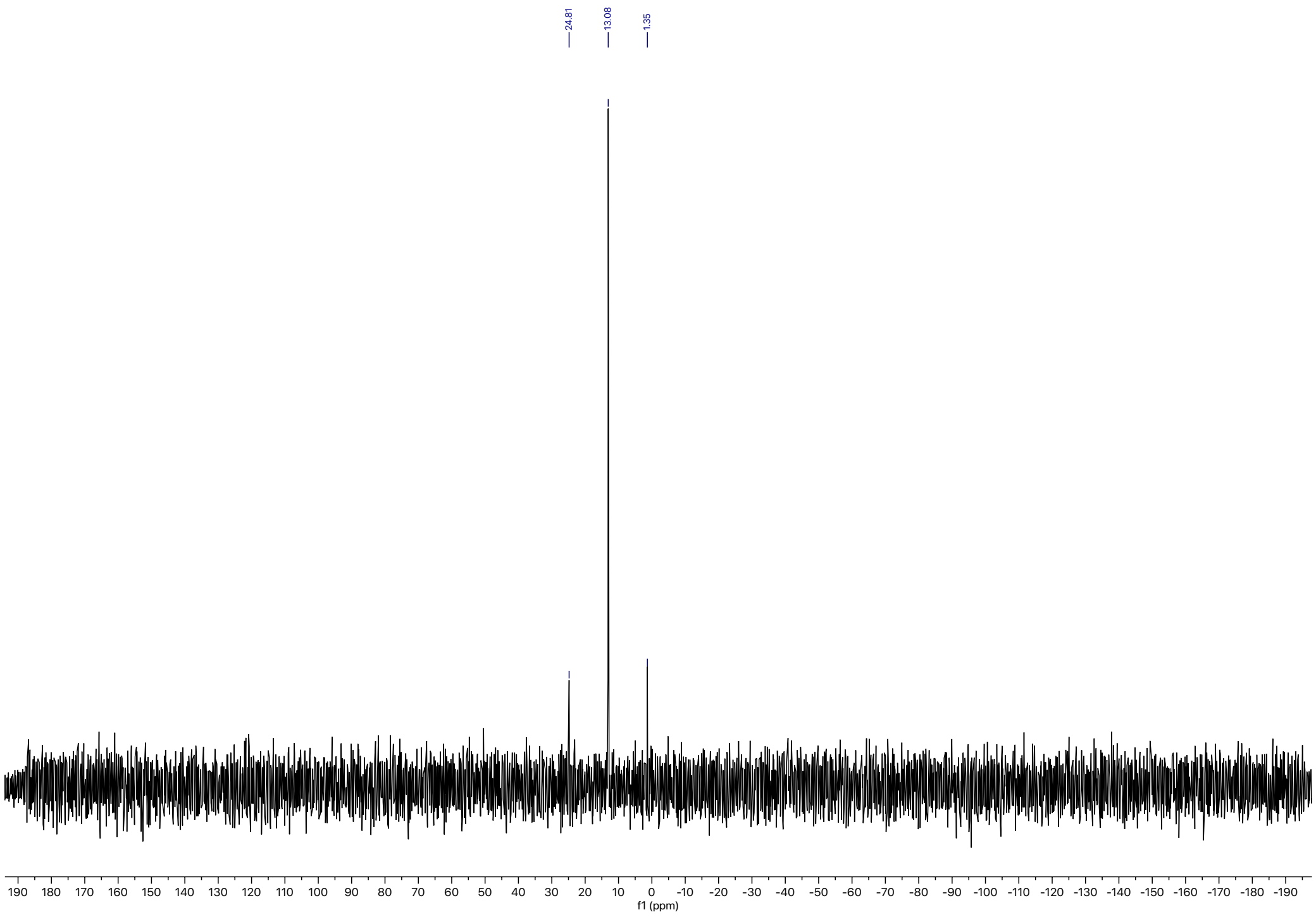

Figure S48. $^{31}P\{^{1}H\}$ NMR spectra of [Pt(dppf)Cl_2] (**14**) (162.0 MHz, CD_3OD, 298K).

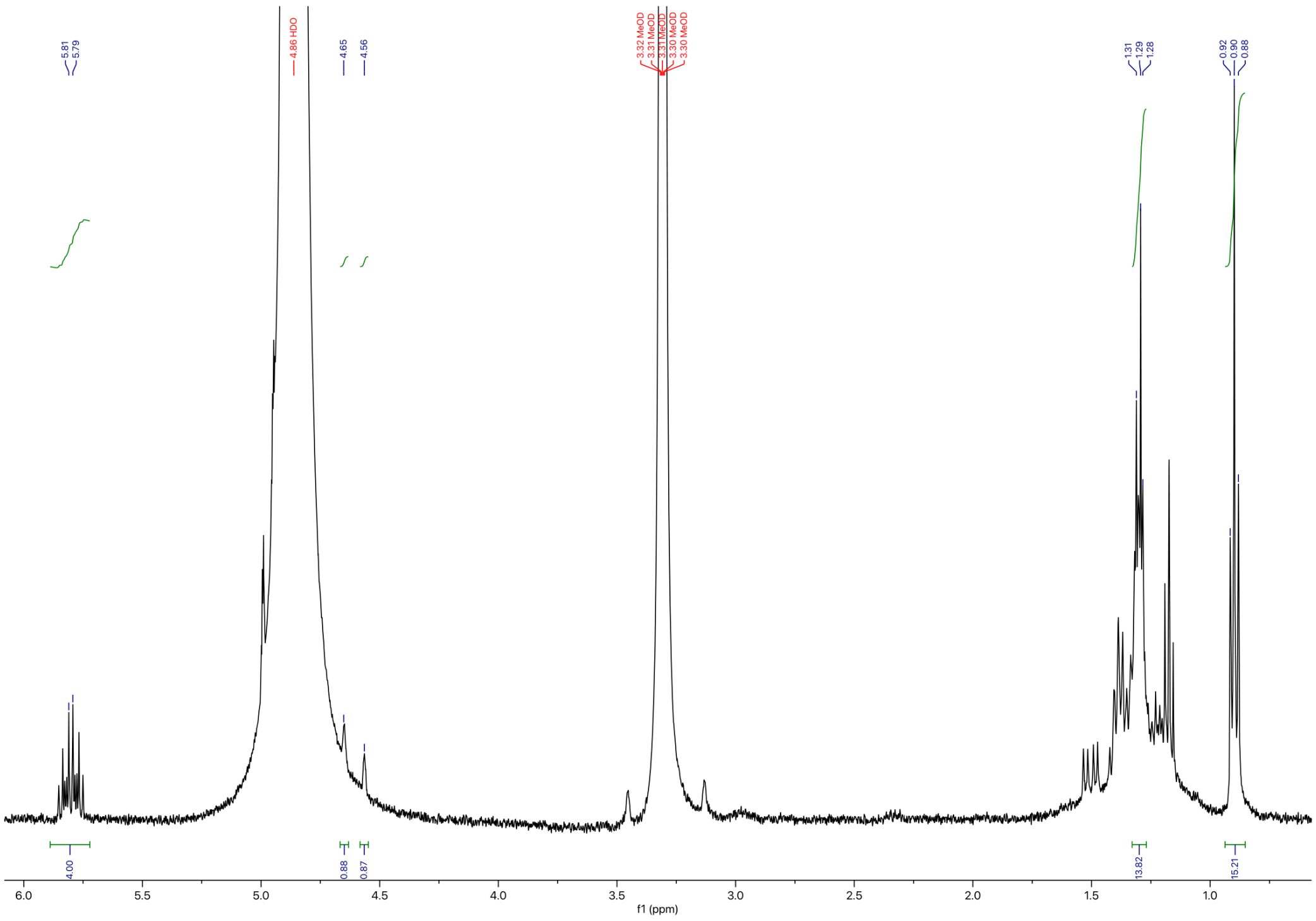

Figure S49. ^{1}H NMR spectra of [Pt(dippf)Cl$_2$] (**15**) (400.1 MHz, CD_3OD, 298K).

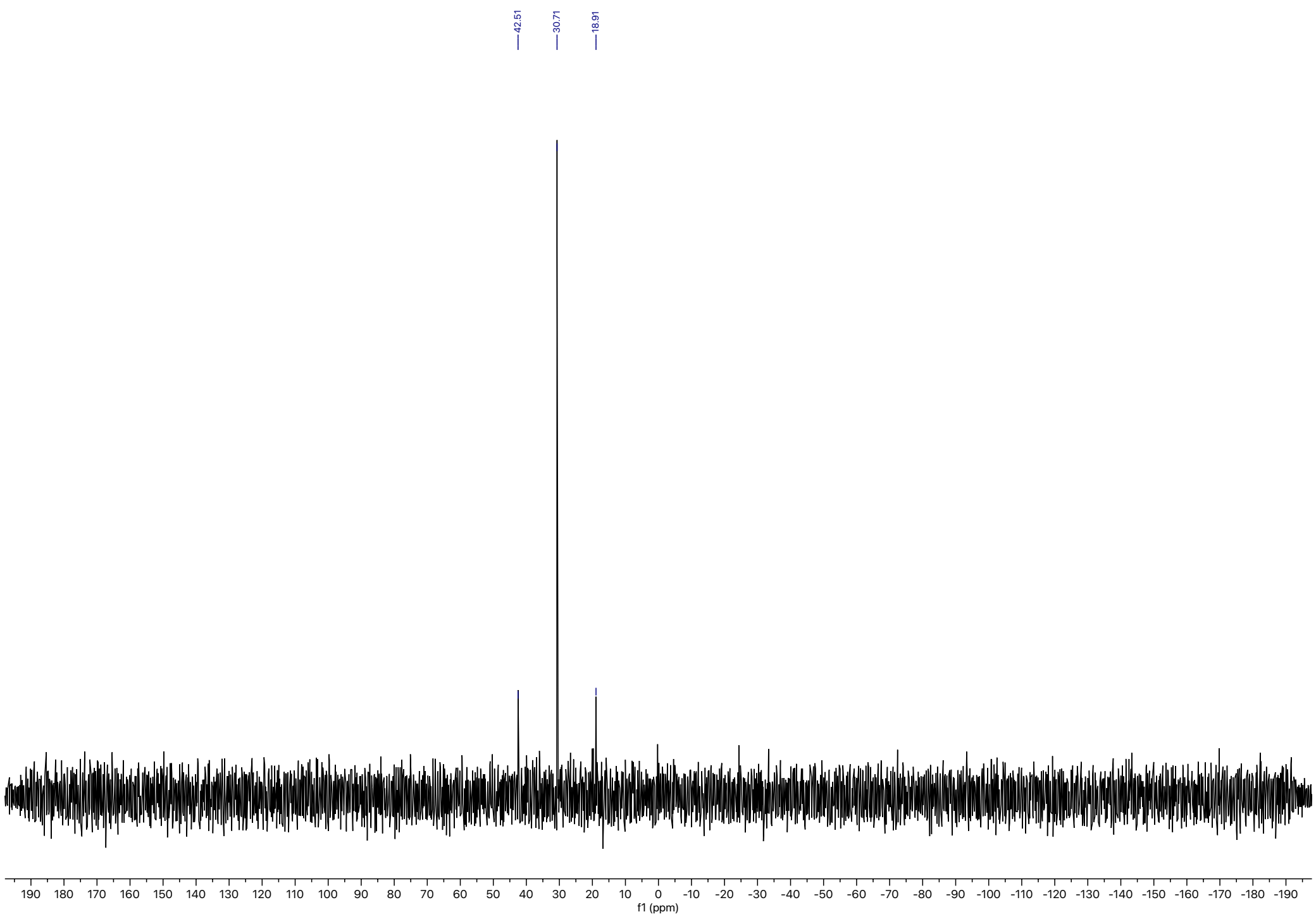

Figure S50. $^{31}P\{^{1}H\}$ NMR spectra of [Pt(dippf)Cl$_2$] (**15**) (162.0 MHz, CD_3OD, 298K).

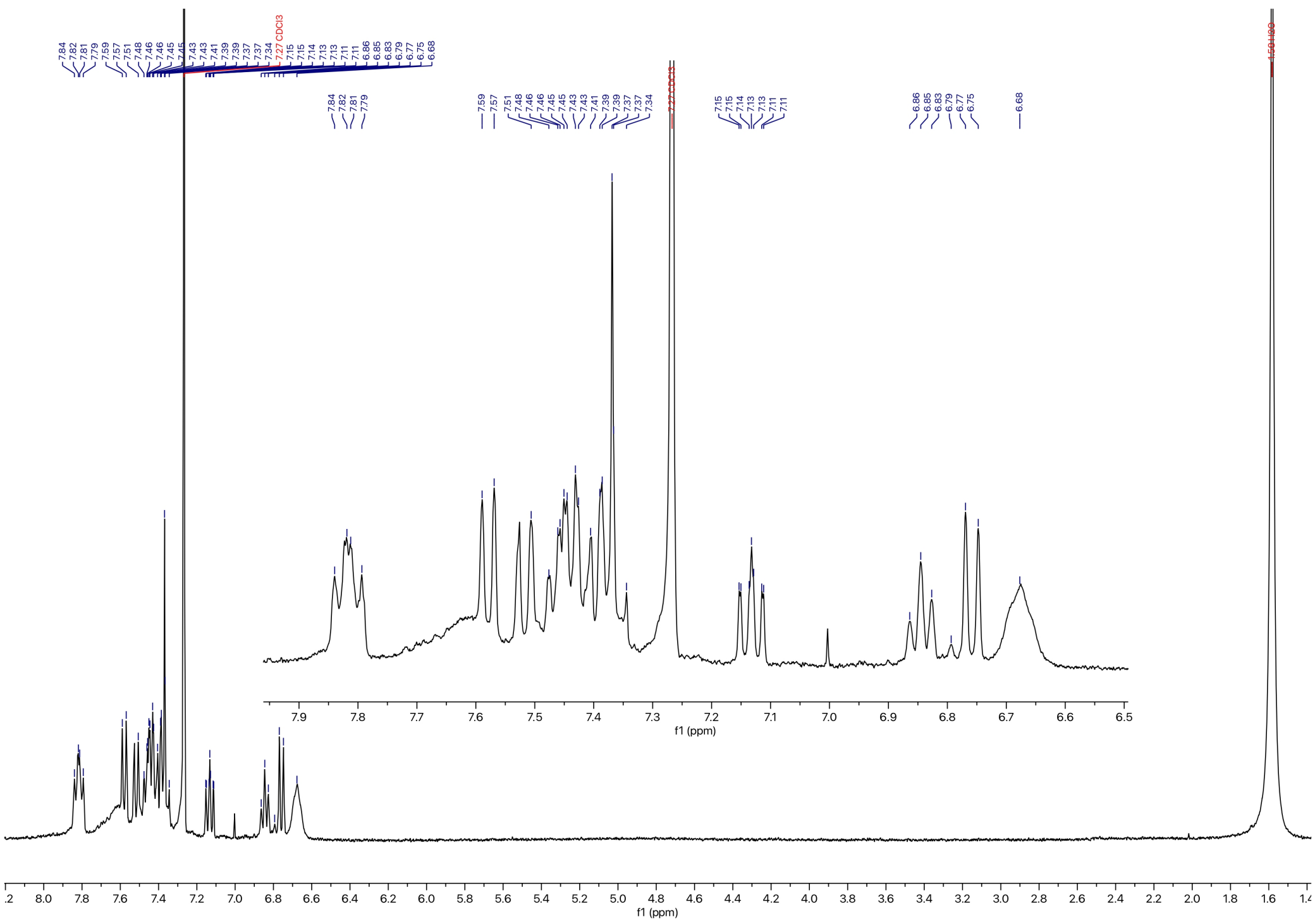

Figure S51. ^{1}H NMR spectra of [Pt((*R*)-BINAP)Cl_2] (**16**) (400.1 MHz, $CDCl_3$, 298K).

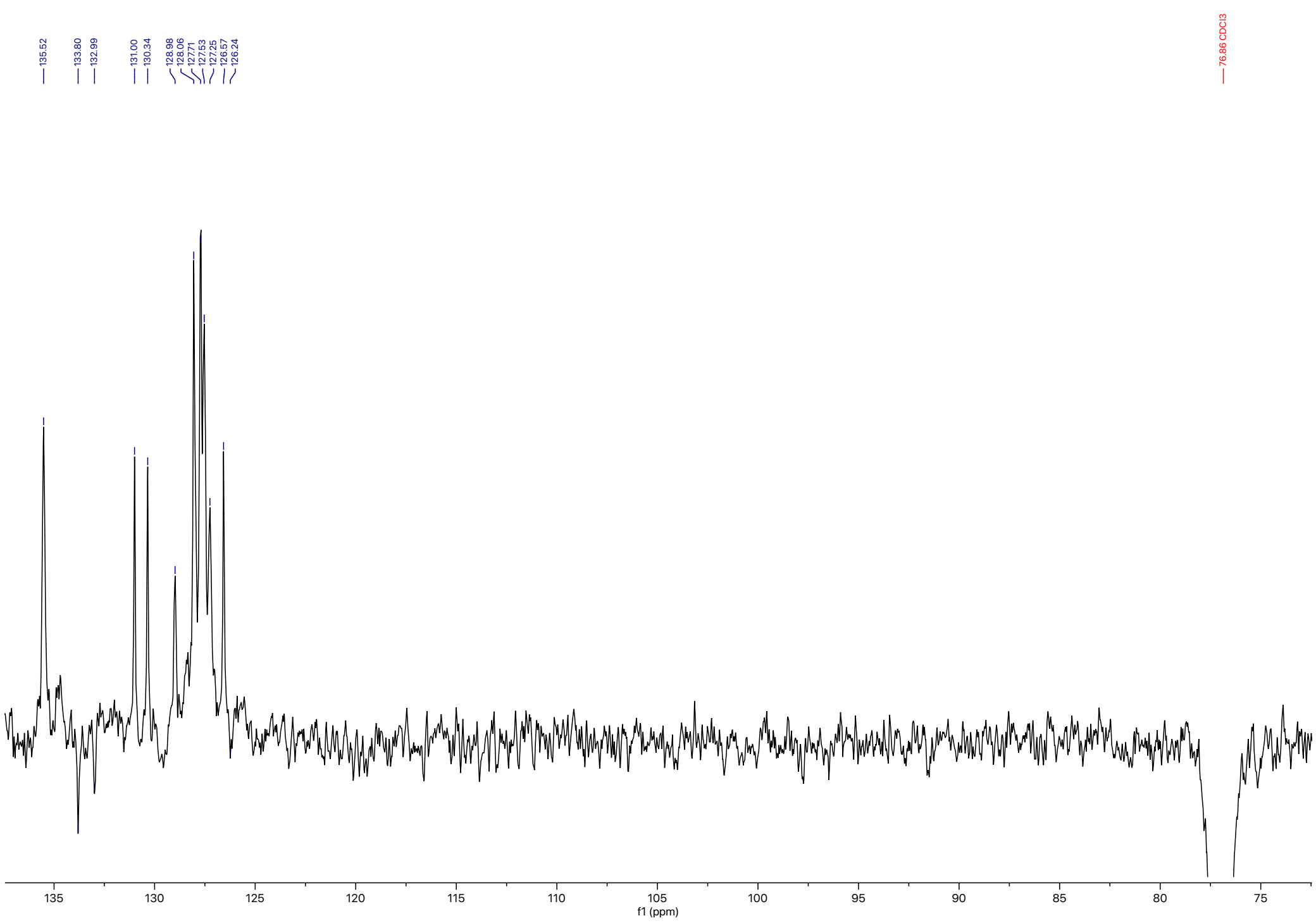

Figure S52. $^{13}C\{^{1}H\}$ NMR spectra of [Pt((*R*)-BINAP)Cl_2] (**16**) (100.6 MHz, $CDCl_3$, 298K).

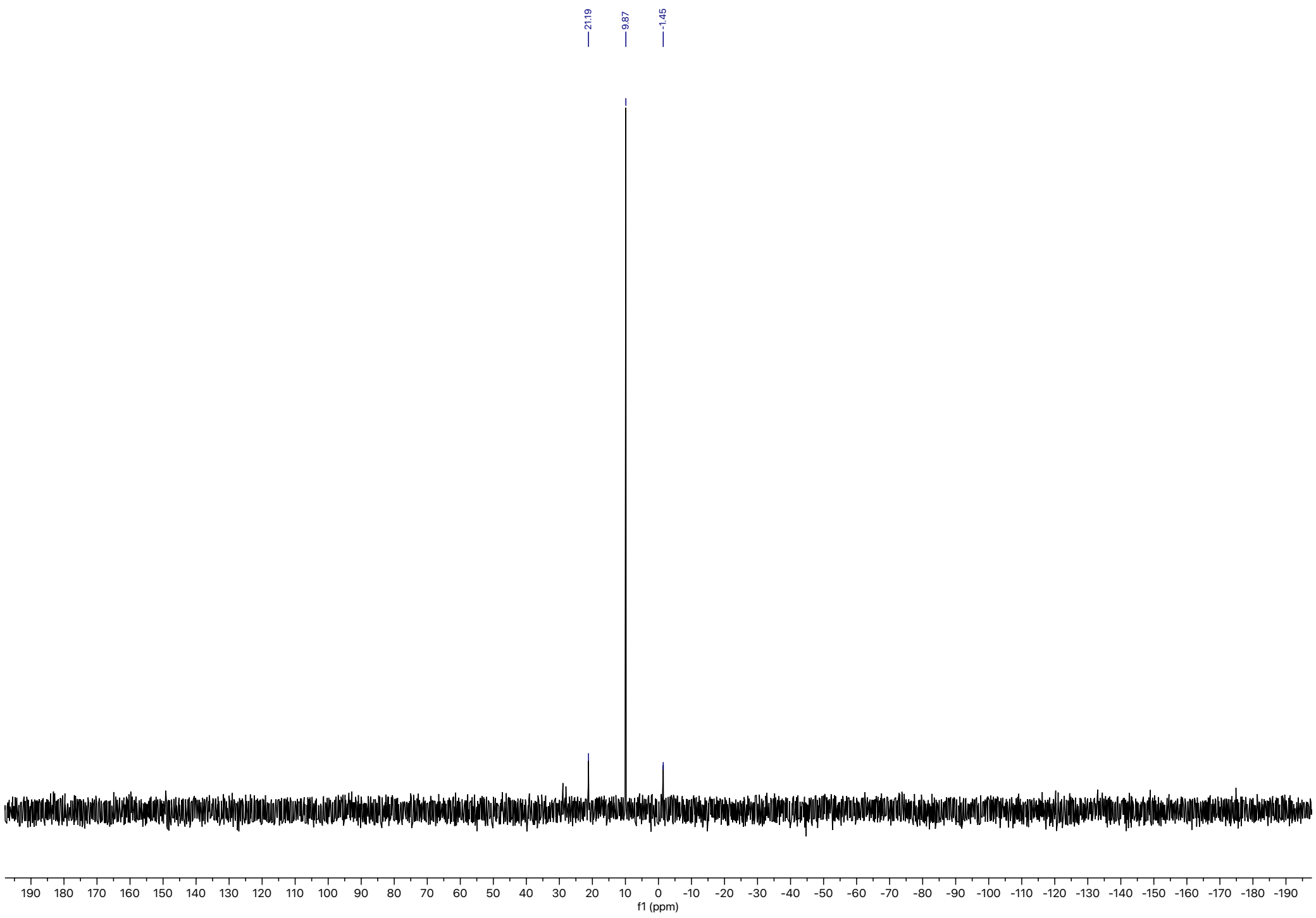

Figure S53. $^{31}P\{^{1}H\}$ NMR spectra of [Pt((*R*)-BINAP)Cl_2] (**16**) (162.0 MHz, $CDCl_3$, 298K).

Part 5. NMR's tests spectra's

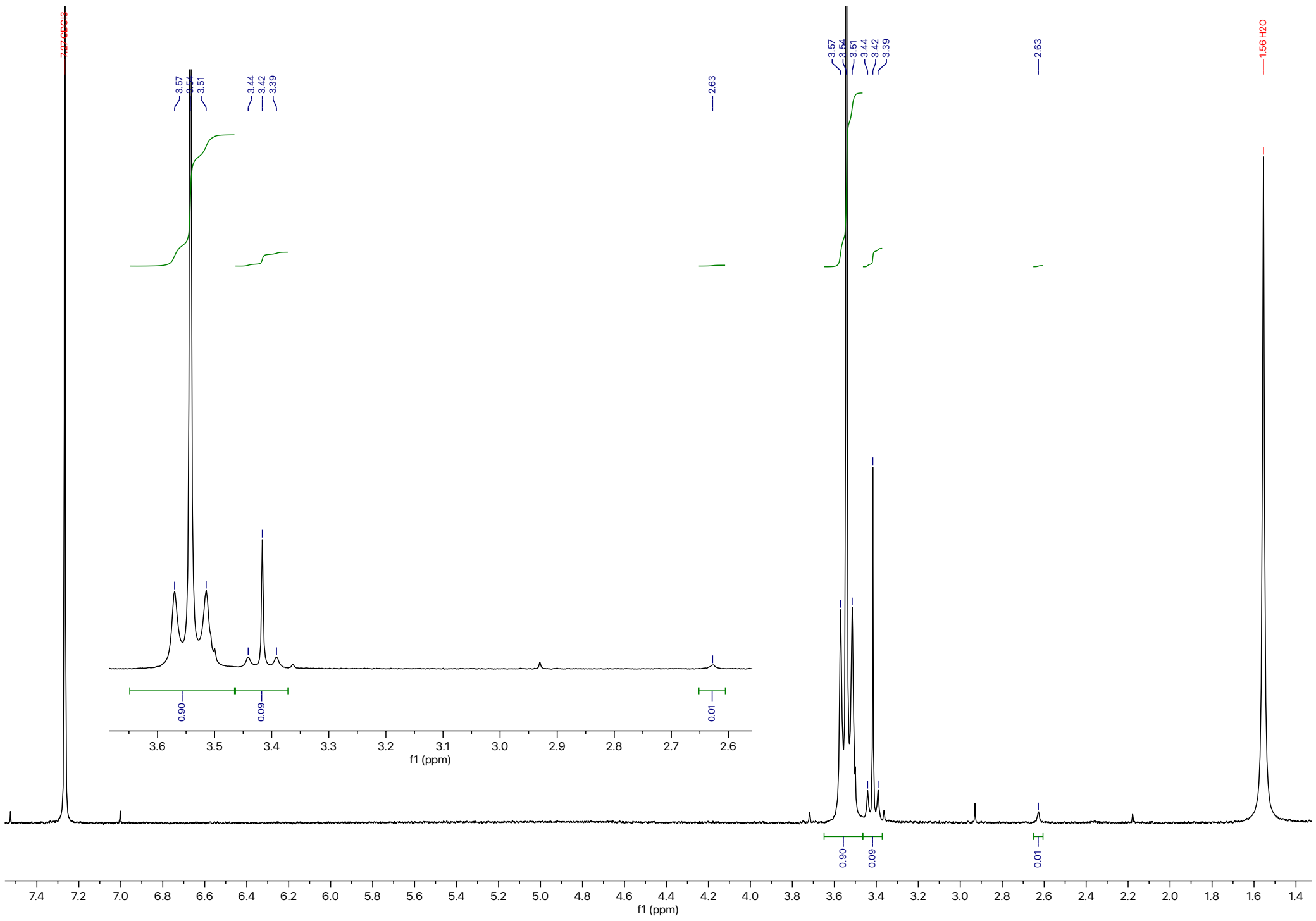

Figure S54. ^{1}H NMR spectra of reaction $PtCl_2$ with DSMO (1:2) (**1**), after 5 minutes of milling (400.1 MHz, $CDCl_3$, 298K).

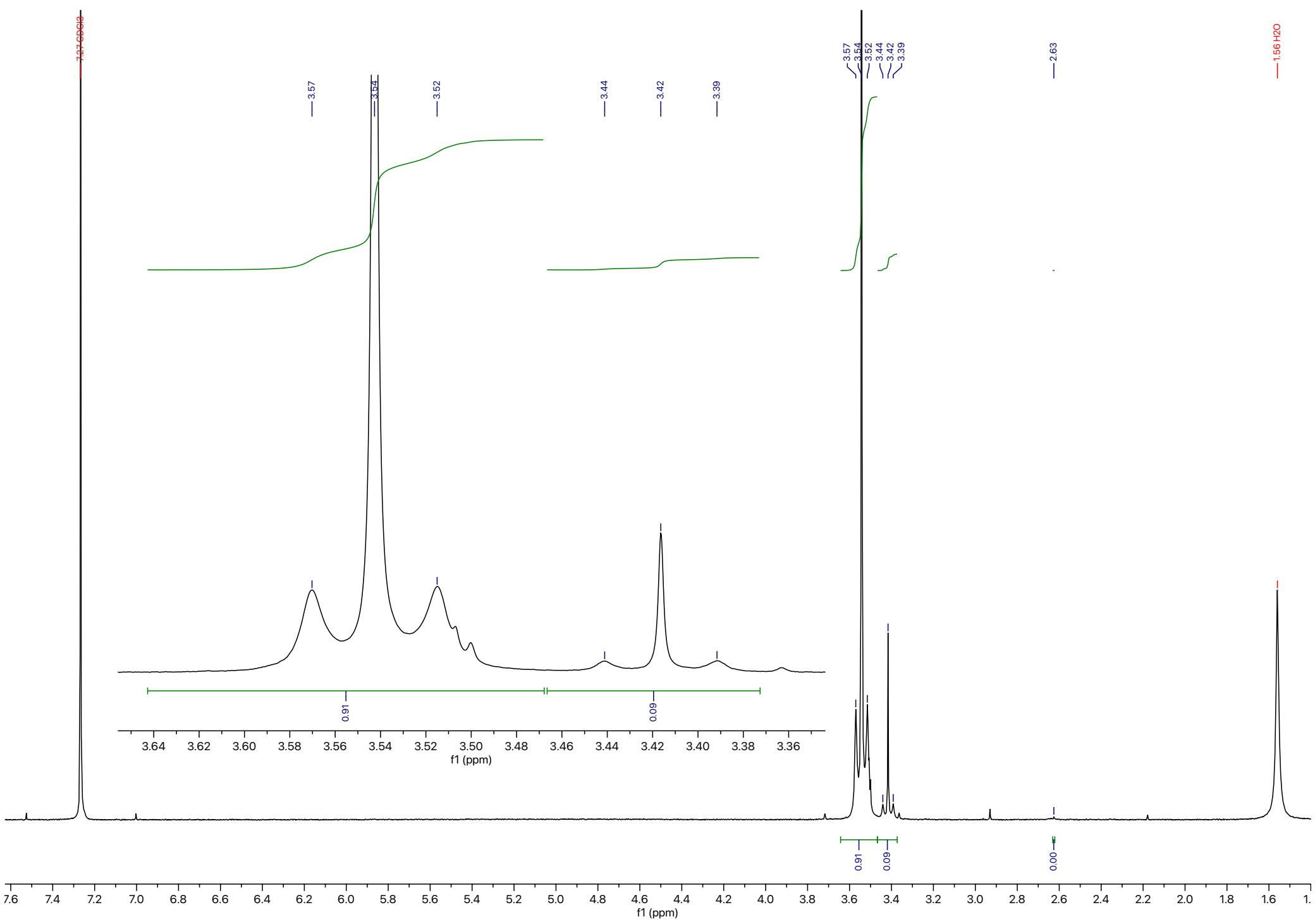

Figure S55. ^{1}H NMR spectra of reaction $PtCl_2$ with DSMO (1:2) (**1**), after 10 minutes of milling (400.1 MHz, $CDCl_3$, 298K).

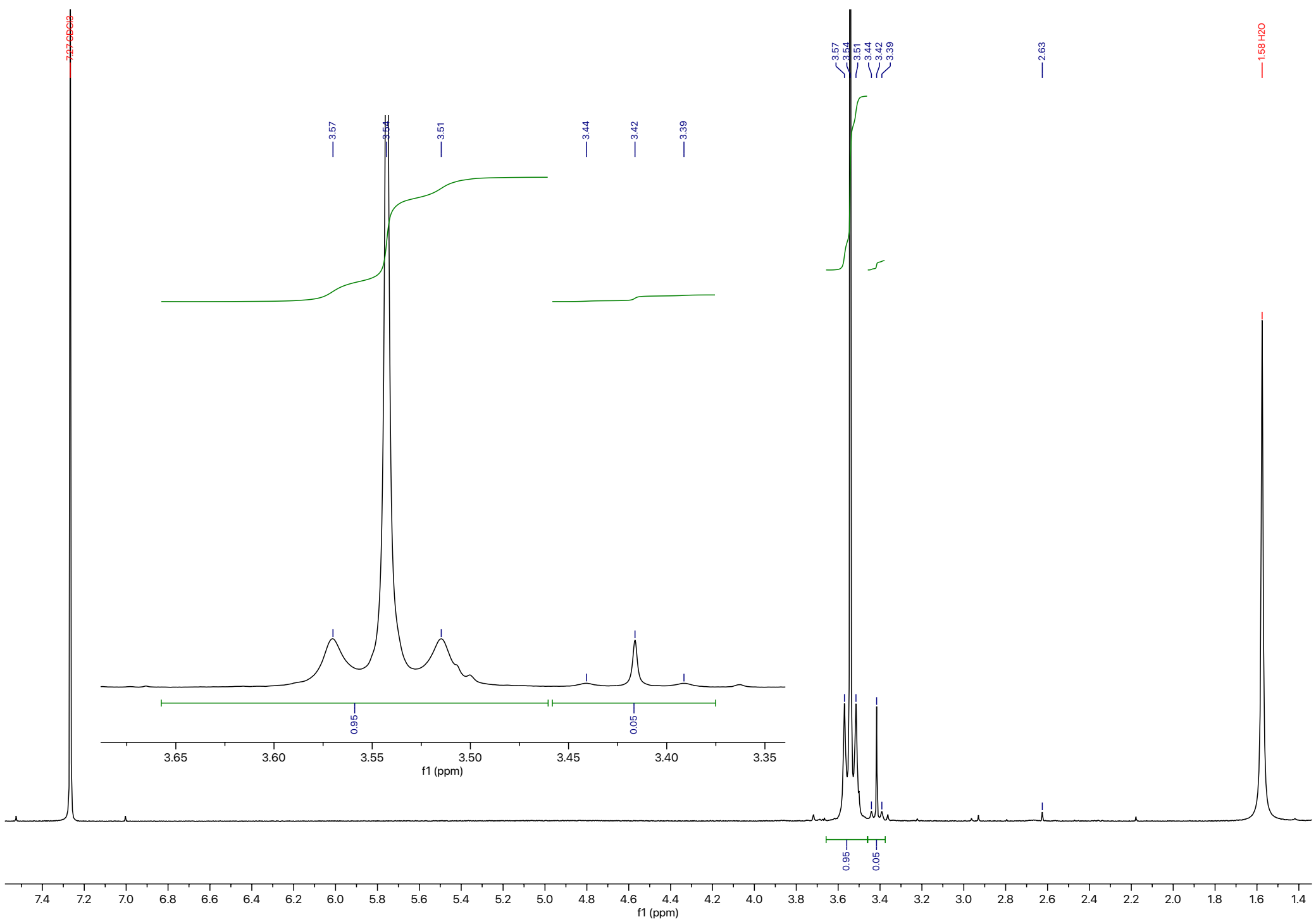

Figure S56. ^1H NMR spectra of reaction $PtCl_2$ with DSMO (1:2) (**1**), after 30 minutes of milling (400.1 MHz, $CDCl_3$, 298K).

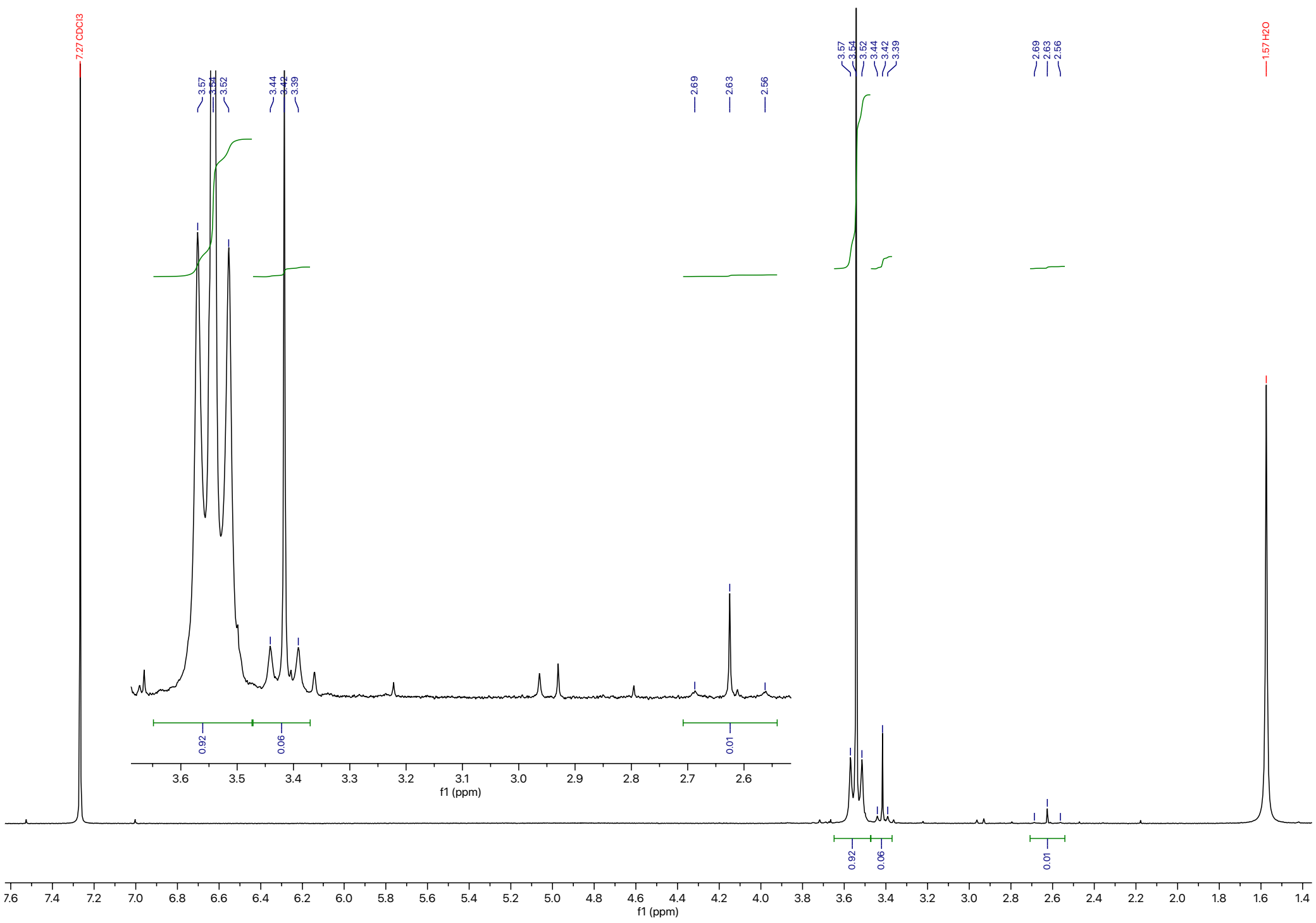

Figure S57. ^1H NMR spectra of reaction $PtCl_2$ with DSMO (1:2) (**1**), after 45 minutes of milling (400.1 MHz, $CDCl_3$, 298K).

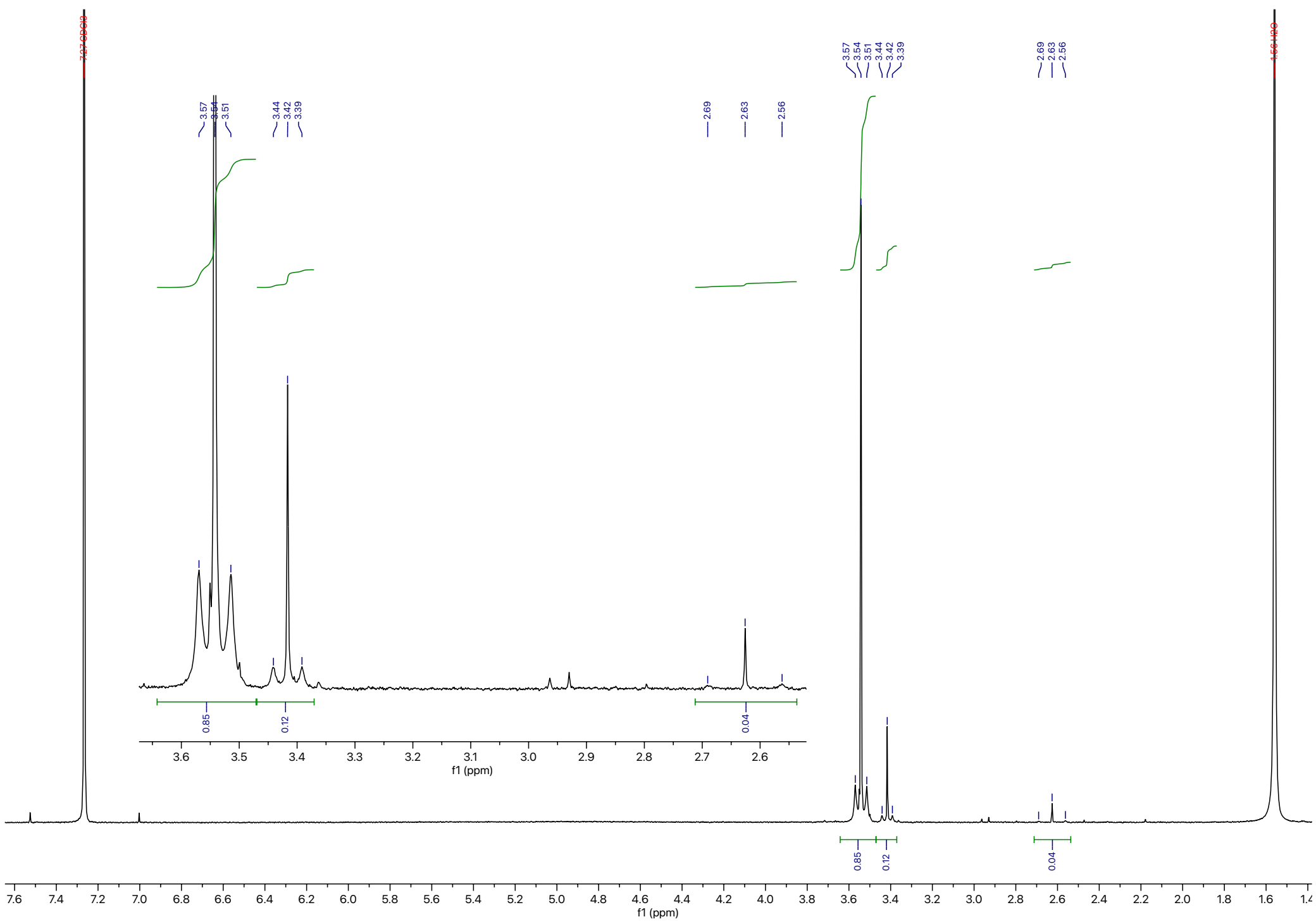

Figure S58. ^{1}H NMR spectra of reaction $PtCl_2$ with DSMO (1:2) (**1**), after 60 minutes of milling (400.1 MHz, $CDCl_3$, 298K).

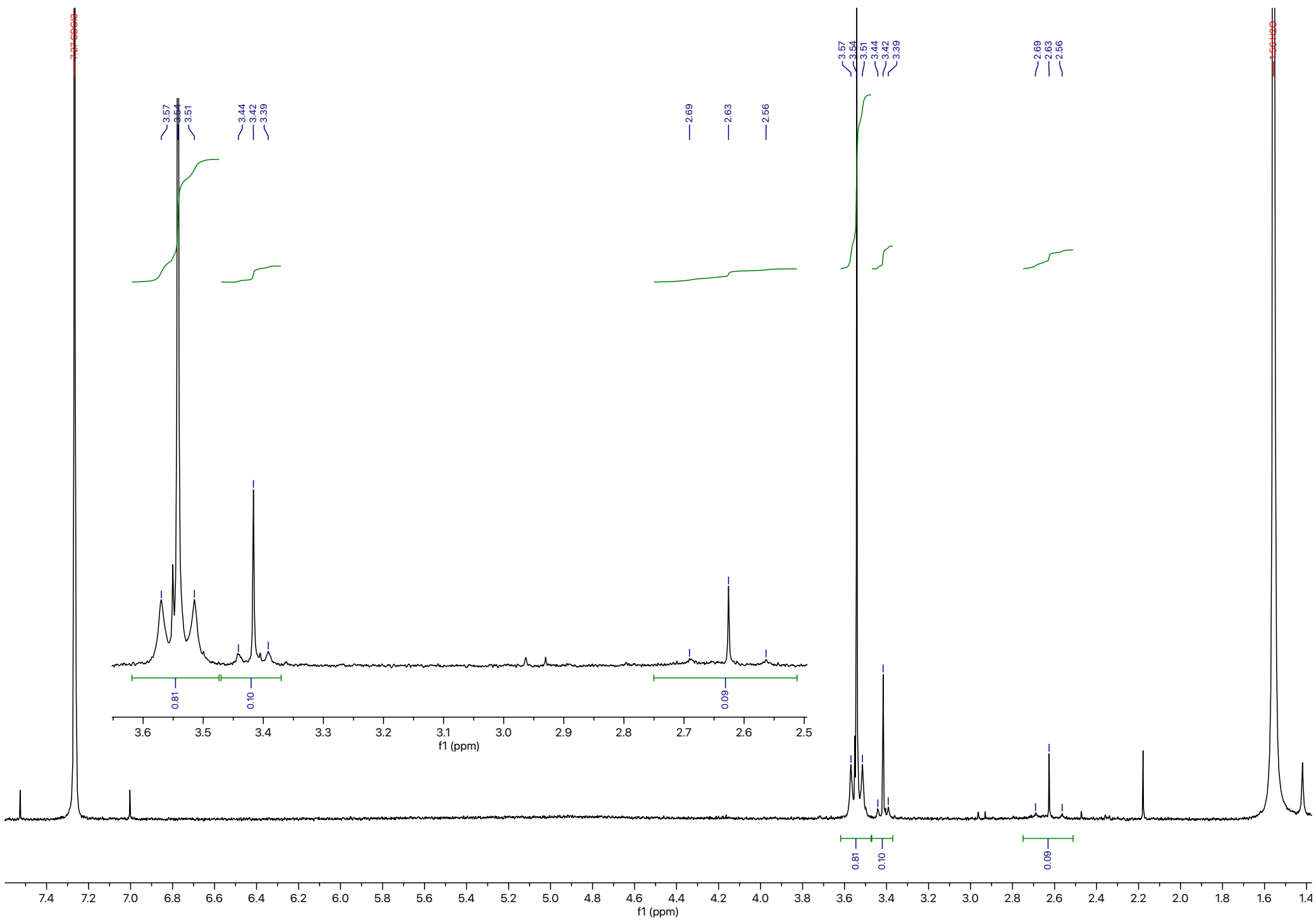

Figure S59. ^{1}H NMR spectra of reaction $PtCl_2$ with DSMO (1:2) (**1**), after 90 minutes of milling (400.1 MHz, $CDCl_3$, 298K).

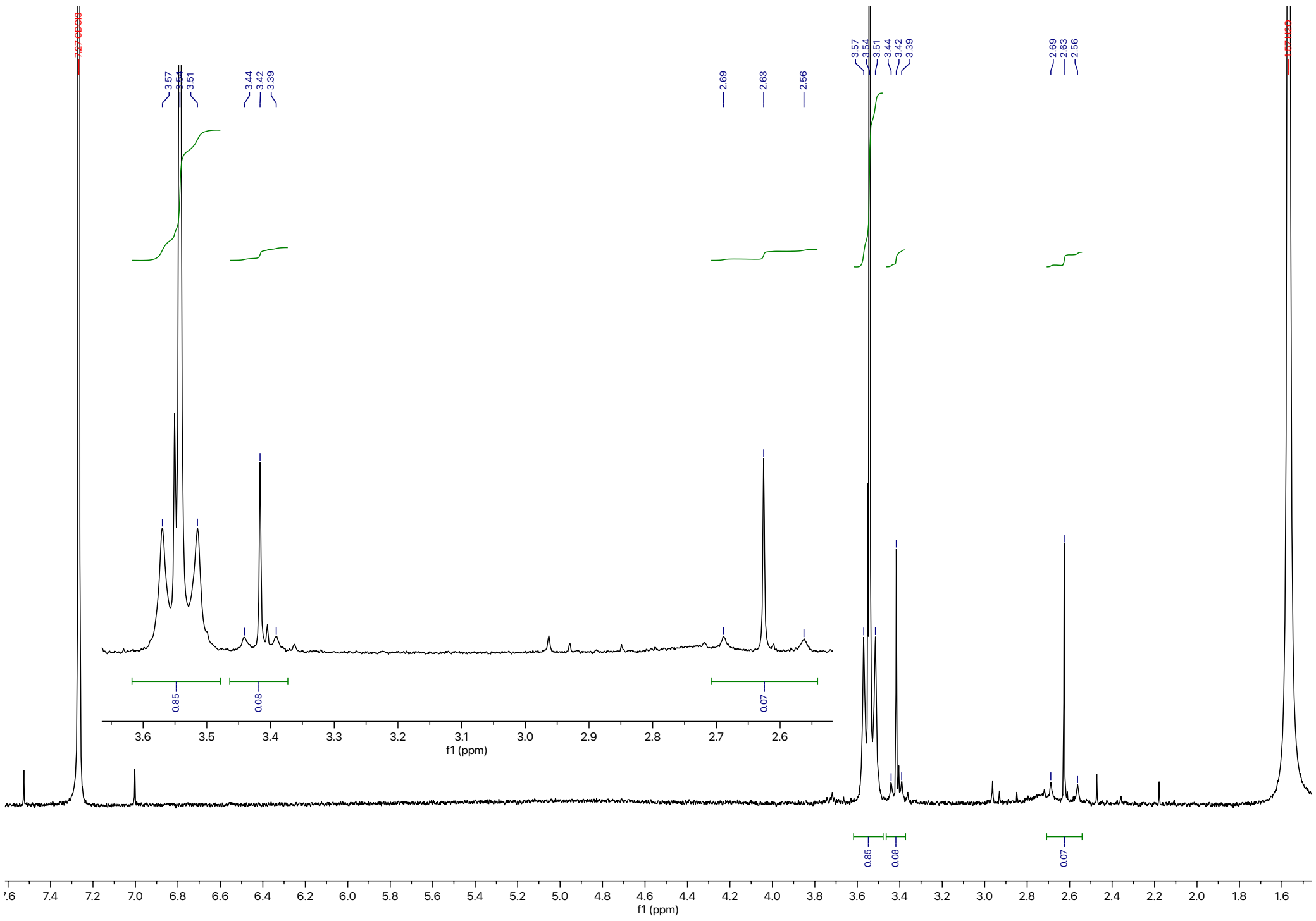

Figure S60. ^{1}H NMR spectra of reaction $PtCl_2$ with DSMO (1:2) (**1**), after 120 minutes of milling (400.1 MHz, $CDCl_3$, 298K).

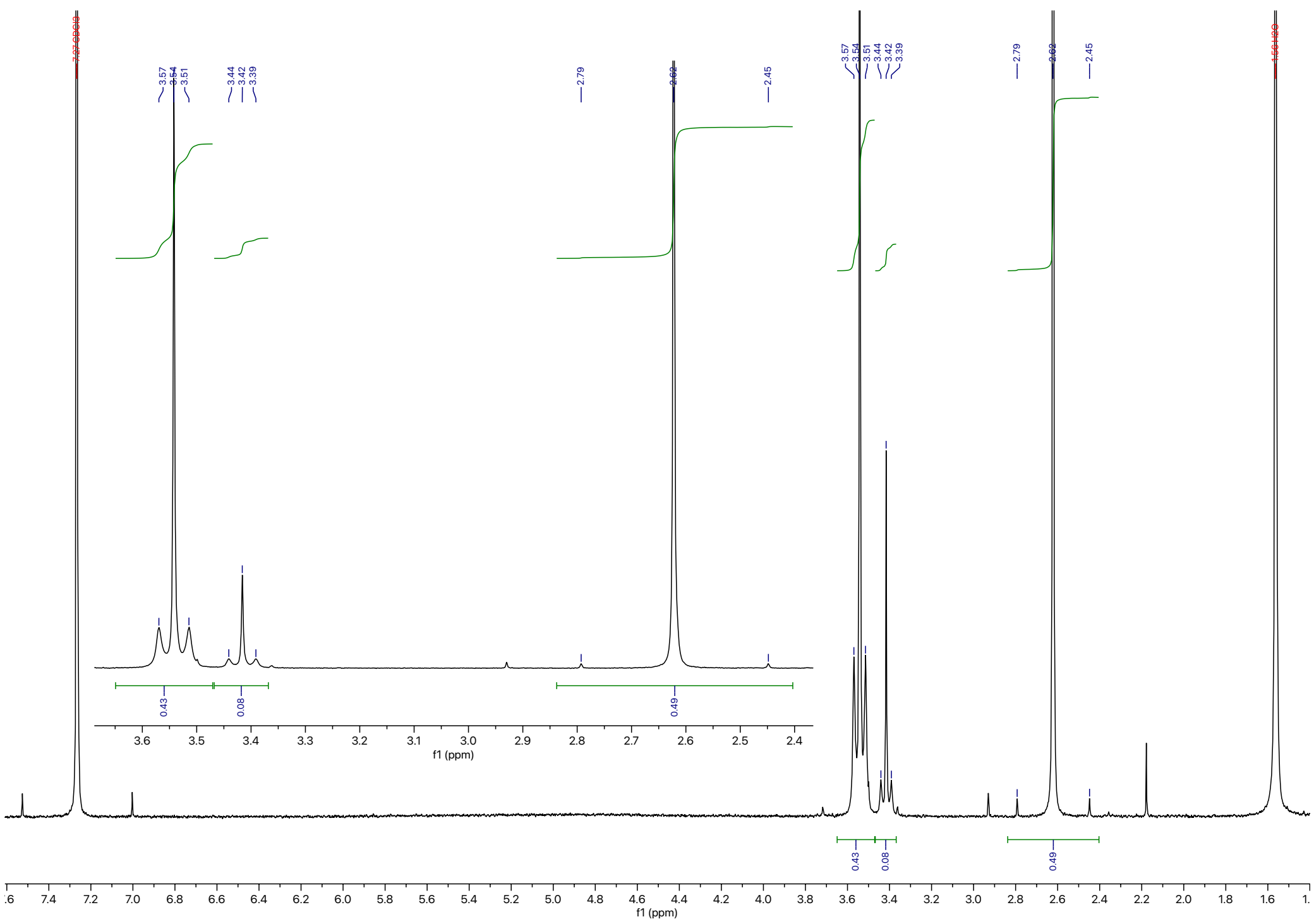

Figure S61. ^{1}H NMR spectra of reaction $K_2[PtCl_4]$ with DMSO (1:2) (**1**), after 5 minutes of milling (400.1 MHz, $CDCl_3$, 298K).

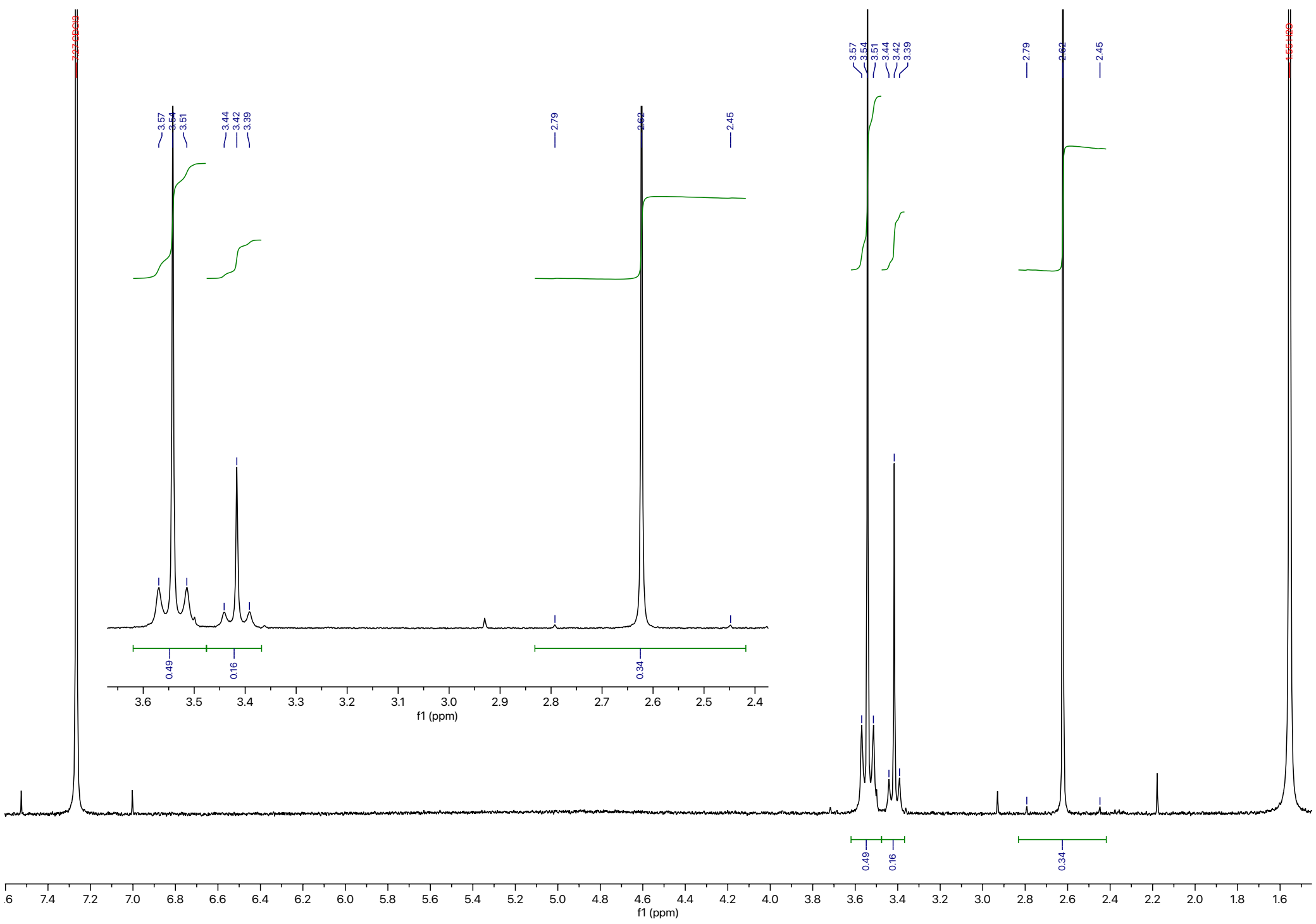

Figure S62. ^{1}H NMR spectra of reaction $K_2[PtCl_4]$ with DSMO (1:2) (**1**), after 10 minutes of milling (400.1 MHz, $CDCl_3$, 298K).

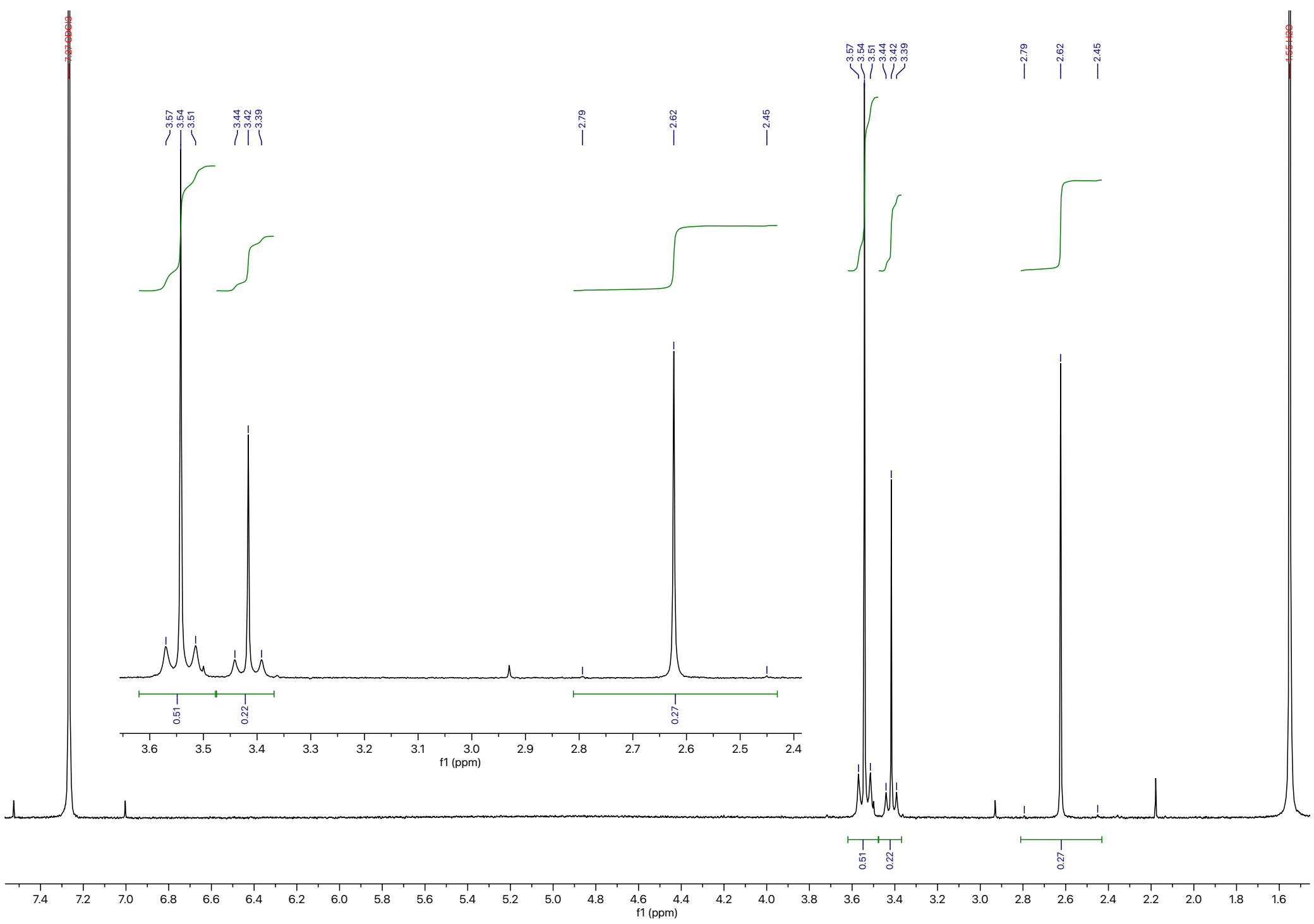

Figure S63. ^{1}H NMR spectra of reaction $K_2[PtCl_4]$ with DSMO (1:2) (**1**), after 15 minutes of milling (400.1 MHz, $CDCl_3$, 298K).

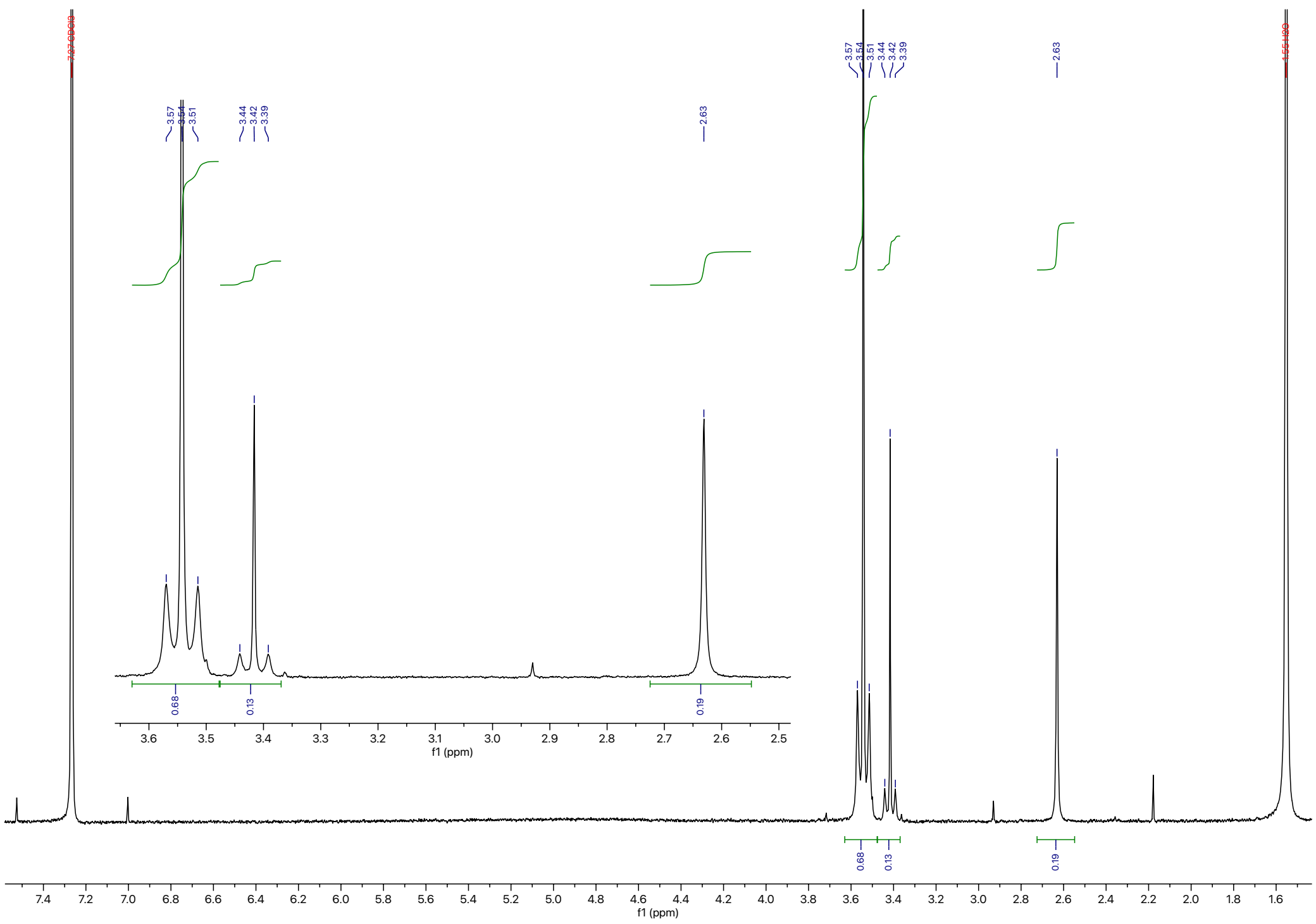

Figure S64. ^{1}H NMR spectra of reaction $K_2[PtCl_4]$ with DSMO (1:2) (**1**), after 45 minutes of milling (400.1 MHz, $CDCl_3$, 298K).

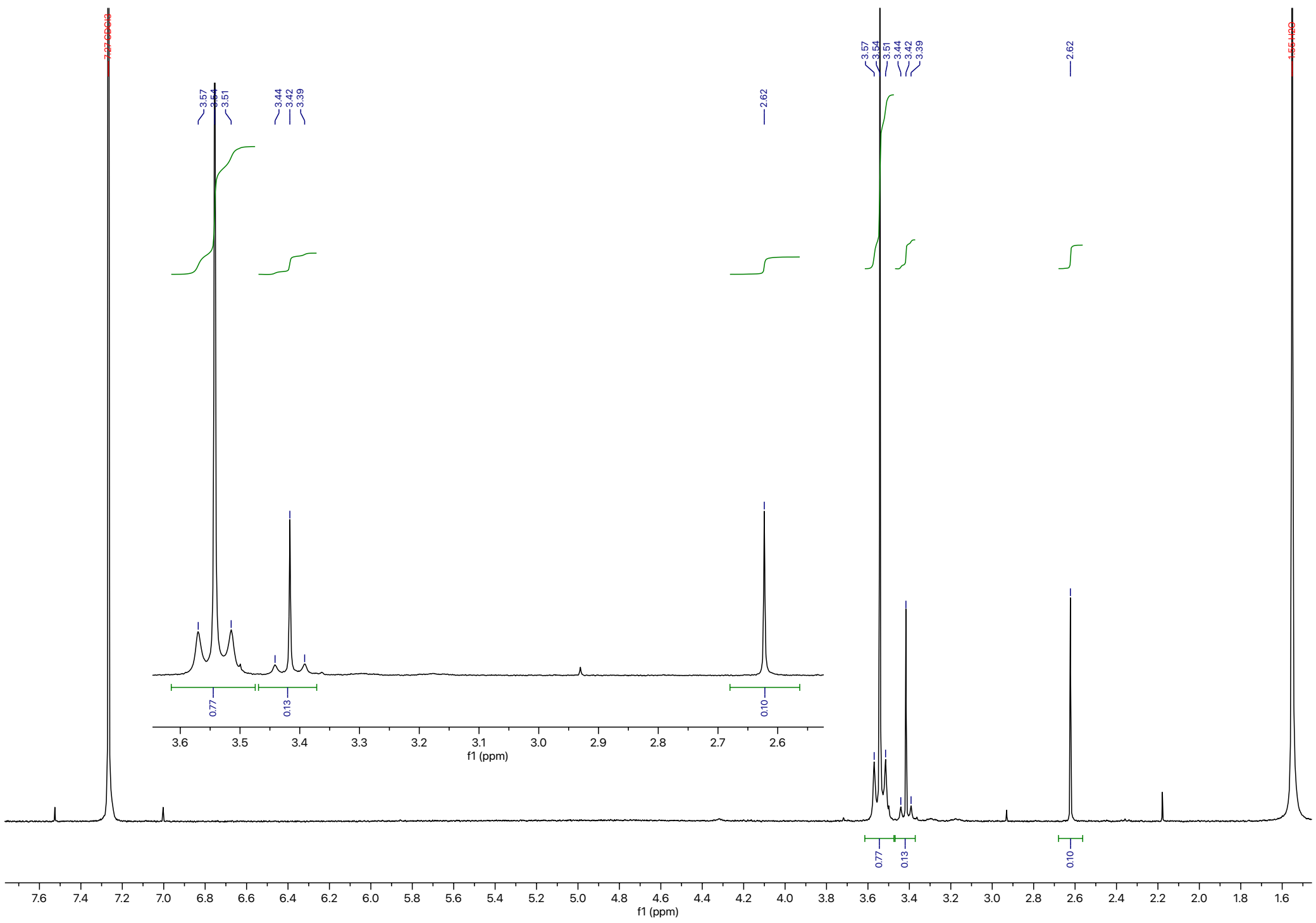

Figure S65. ^{1}H NMR spectra of reaction $K_2[PtCl_4]$ with DSMO (1:2) (**1**), after 60 minutes of milling (400.1 MHz, $CDCl_3$, 298K).

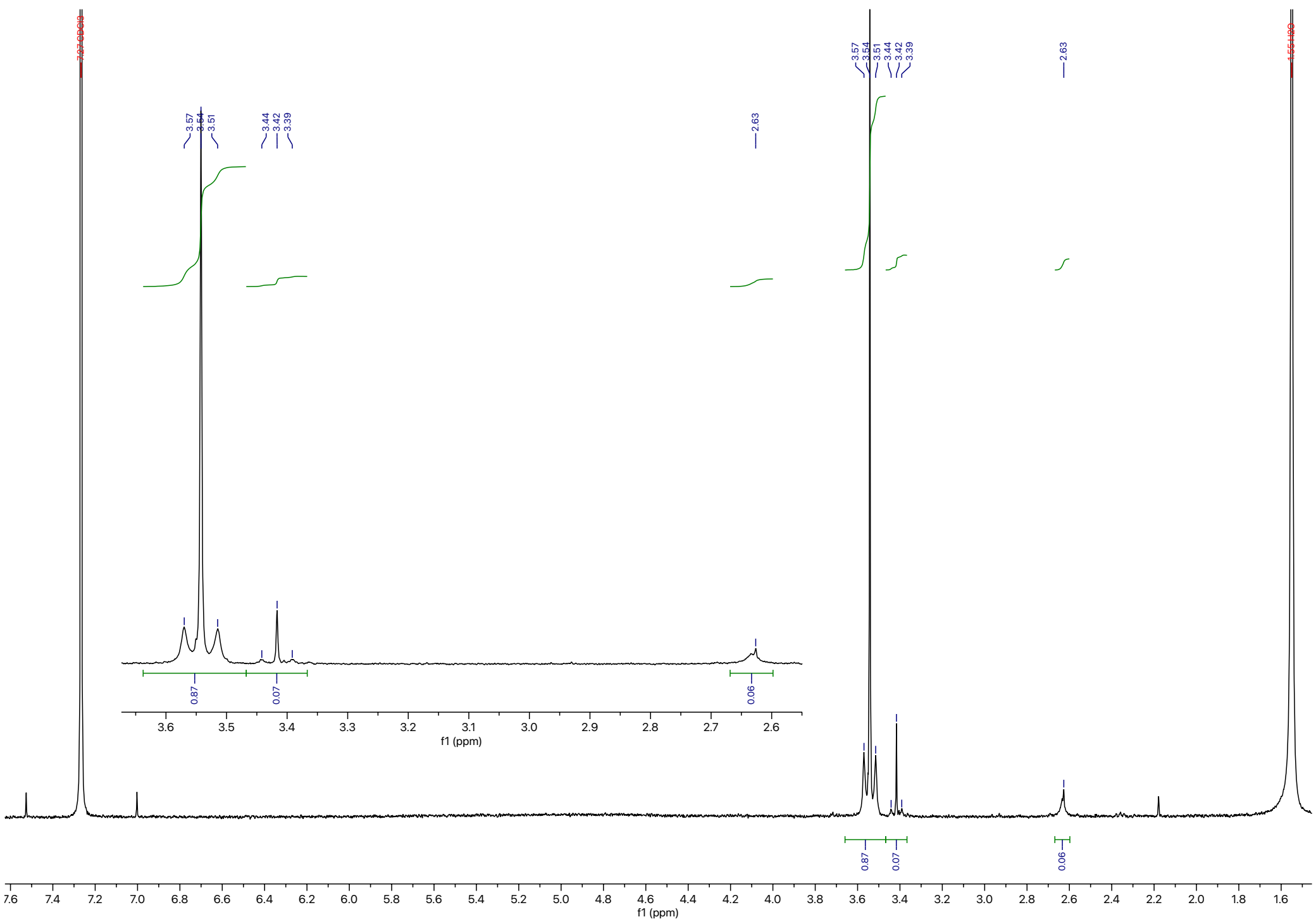

Figure S66. ^{1}H NMR spectra of reaction $K_2[PtCl_4]$ with DSMO (1:2) (**1**), after 90 minutes of milling (400.1 MHz, $CDCl_3$, 298K).

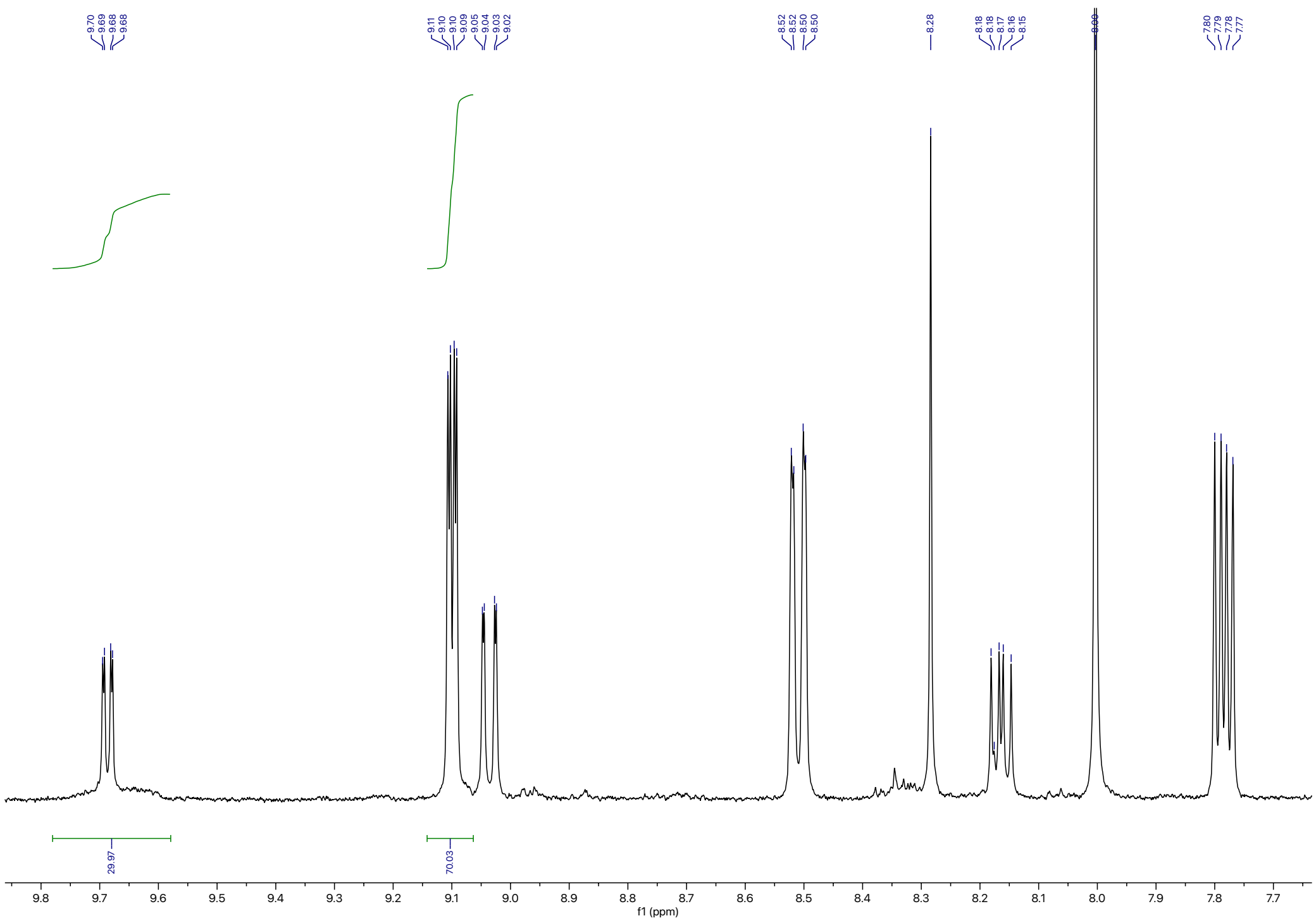

Figure S67. ^{1}H NMR spectra of reaction $K_2[PtCl_4]$ with phen (1:1) (**3**), after 120 minutes of milling (400.1 MHz, DMSO-d_6, 298K). $\chi_{(120)}$ = 30%.

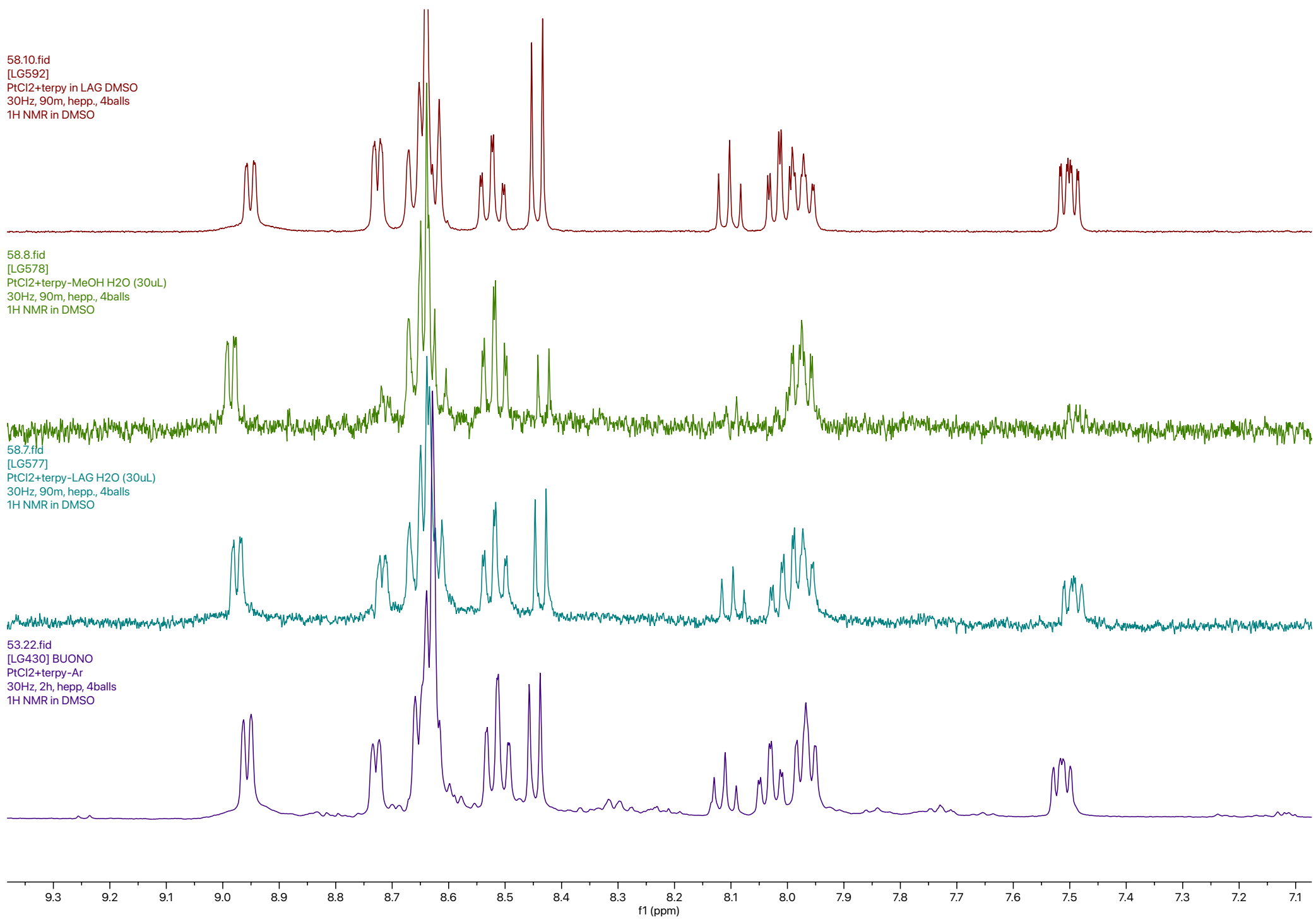

Figure S68. ^{1}H NMR (400.1 MHz, DMSO-d$_6$, 298K) spectra from the bottom to the top: PtCl$_2$+terpy-dry (purple); PtCl$_2$+terpy-LAG H$_2$O (marine blue); PtCl$_2$+terpy-LAG MeOH (green); PtCl$_2$+terpy-LAG DMSO (red). PP Epp. (2 mL), 4 zirconia balls, 90 minutes, 30 Hz, Ar.

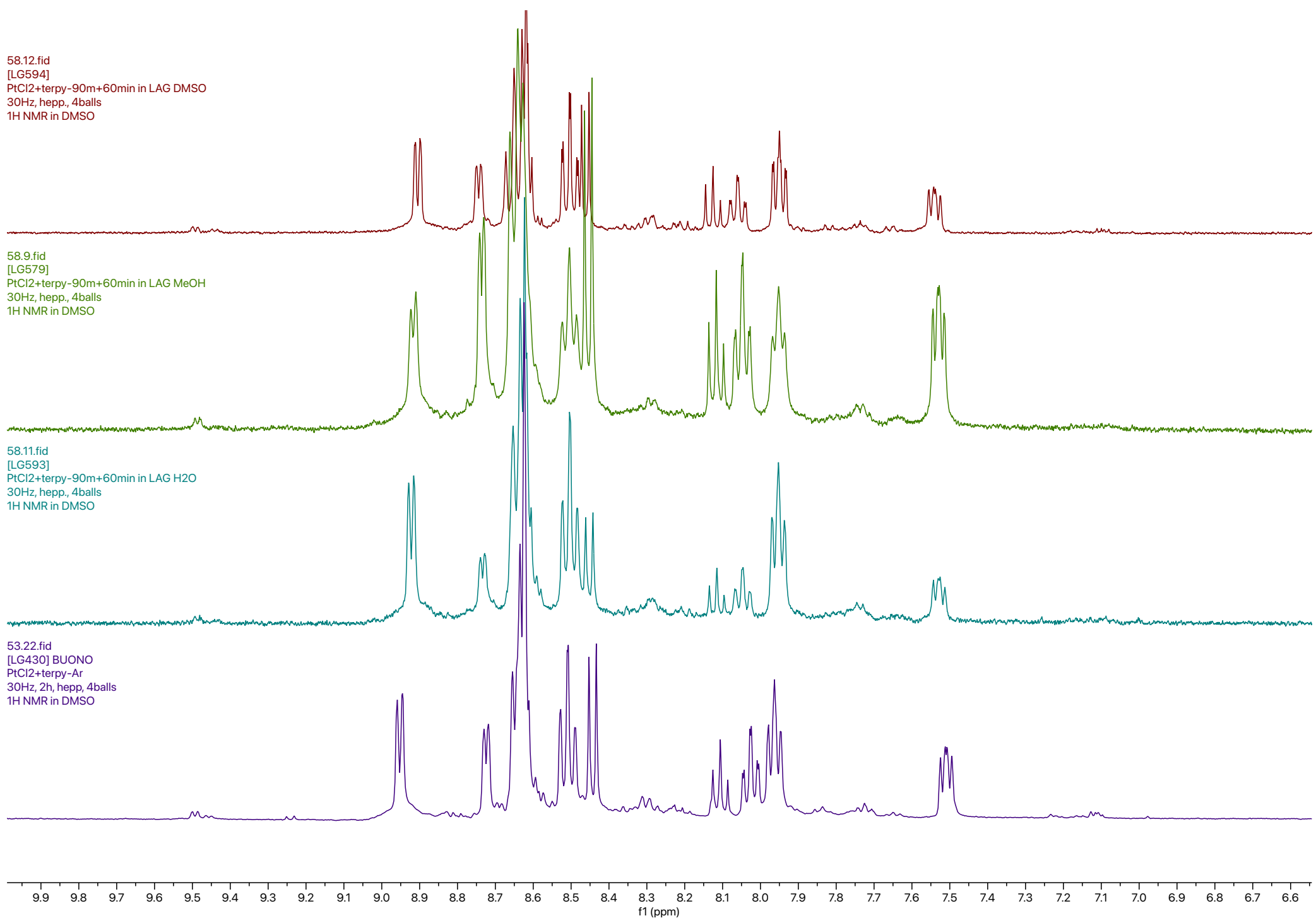

Figure S69. ^{1}H NMR (400.1 MHz, DMSO-d$_6$, 298K) spectra from the bottom to the top: PtCl$_2$+terpy-dry (purple); PtCl$_2$+terpy-then 60 min of LAG H$_2$O (marine blue); PtCl$_2$+terpy-then 60 min of LAG MeOH (green); PtCl$_2$+terpy-then 60 min of LAG DMSO (red). PP Epp. (2 mL), 4 zirconia balls, 90 minutes, 30 Hz, Ar. Then others 60 minutes with 50 µL of solvent.

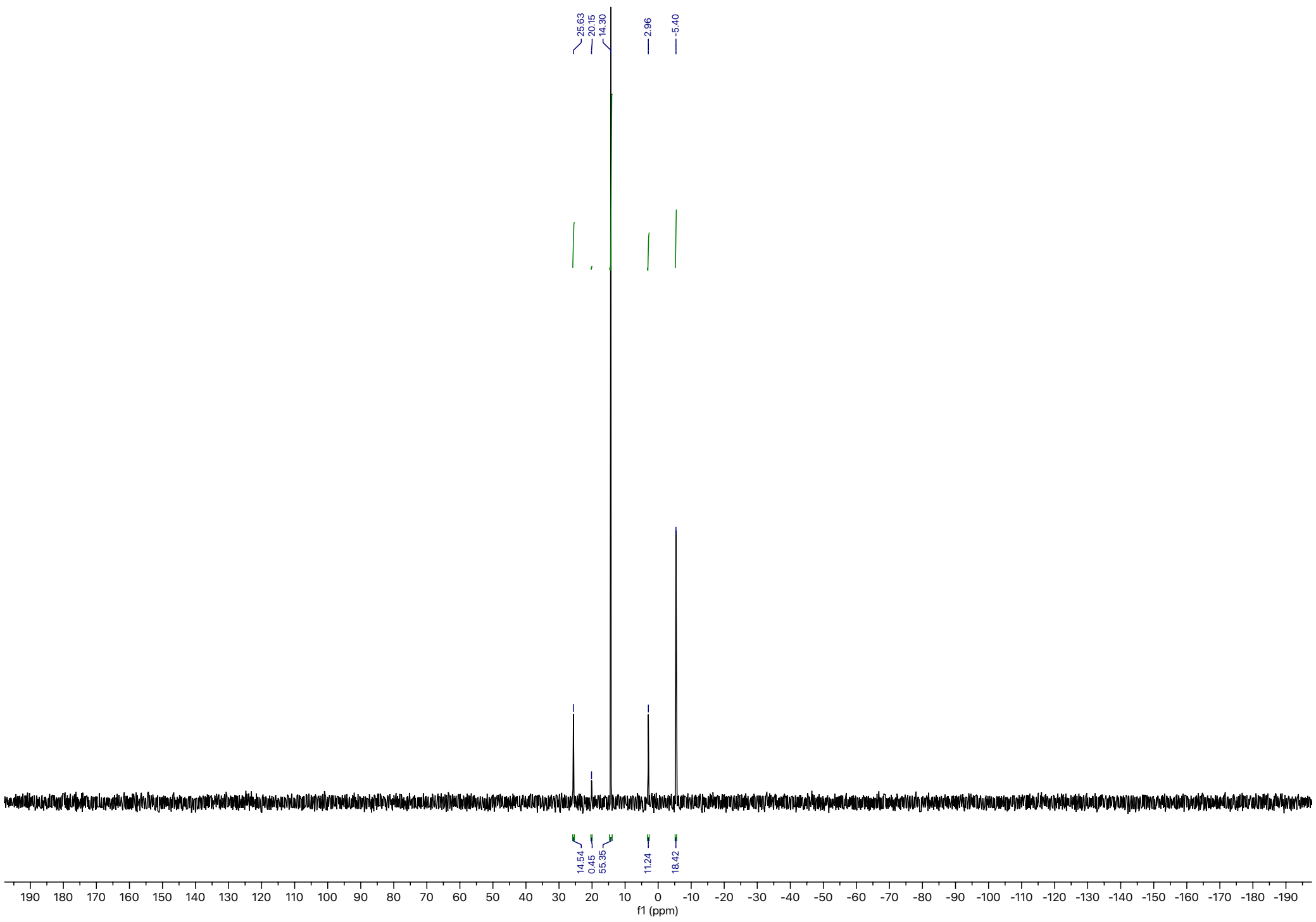

Figure S70. $^{31}P\{^{1}H\}$ NMR spectra of reaction $PtCl_2$ with PPh_3 (1:2) (**6**), after 5 minutes of milling (162.0 MHz, $CDCl_3$, 298K).

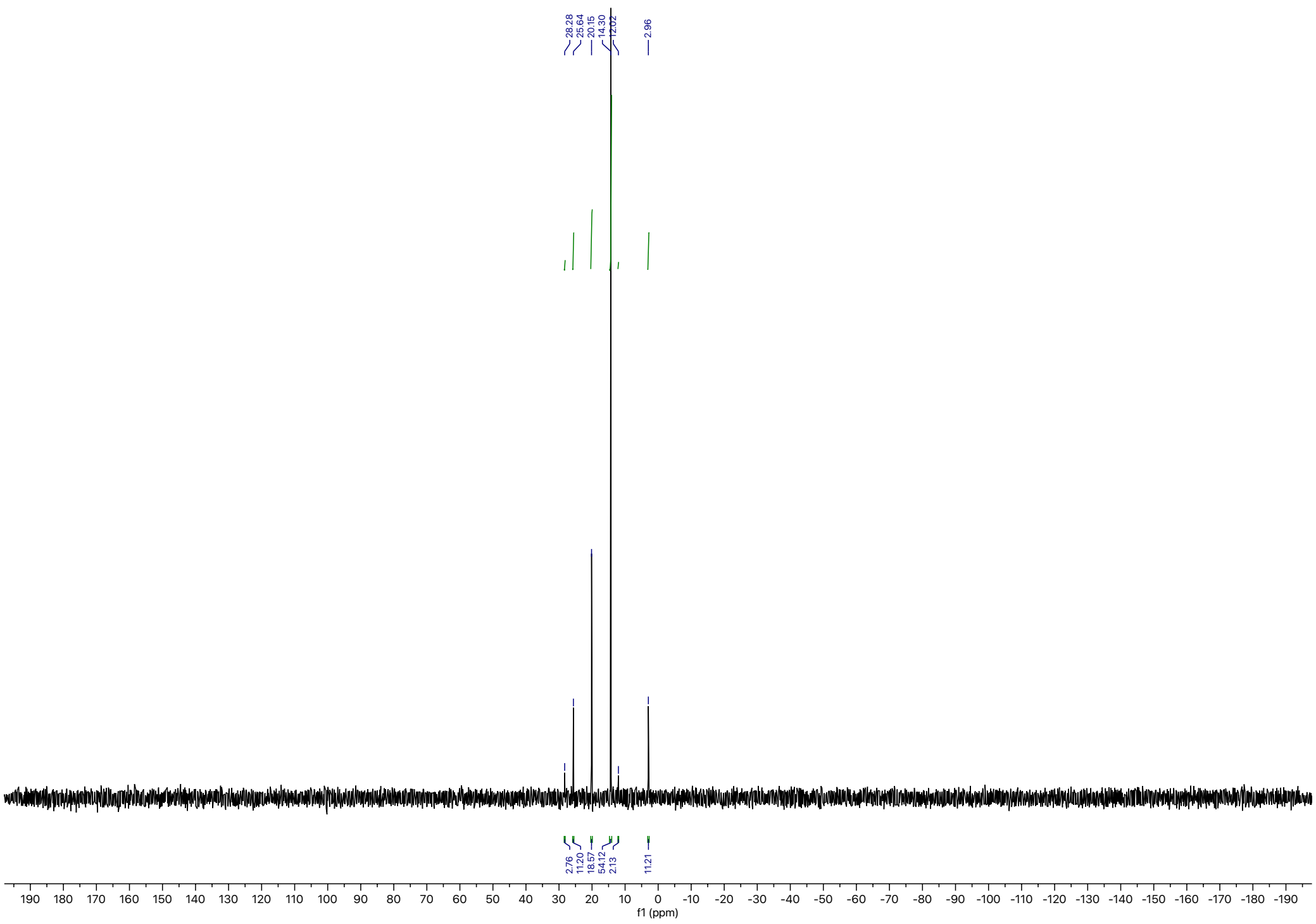

Figure S71. $^{31}P\{^{1}H\}$ NMR spectra of reaction $PtCl_2$ with PPh_3 (1:2) (**6**), after 10 minutes of milling (162.0 MHz, $CDCl_3$, 298K).

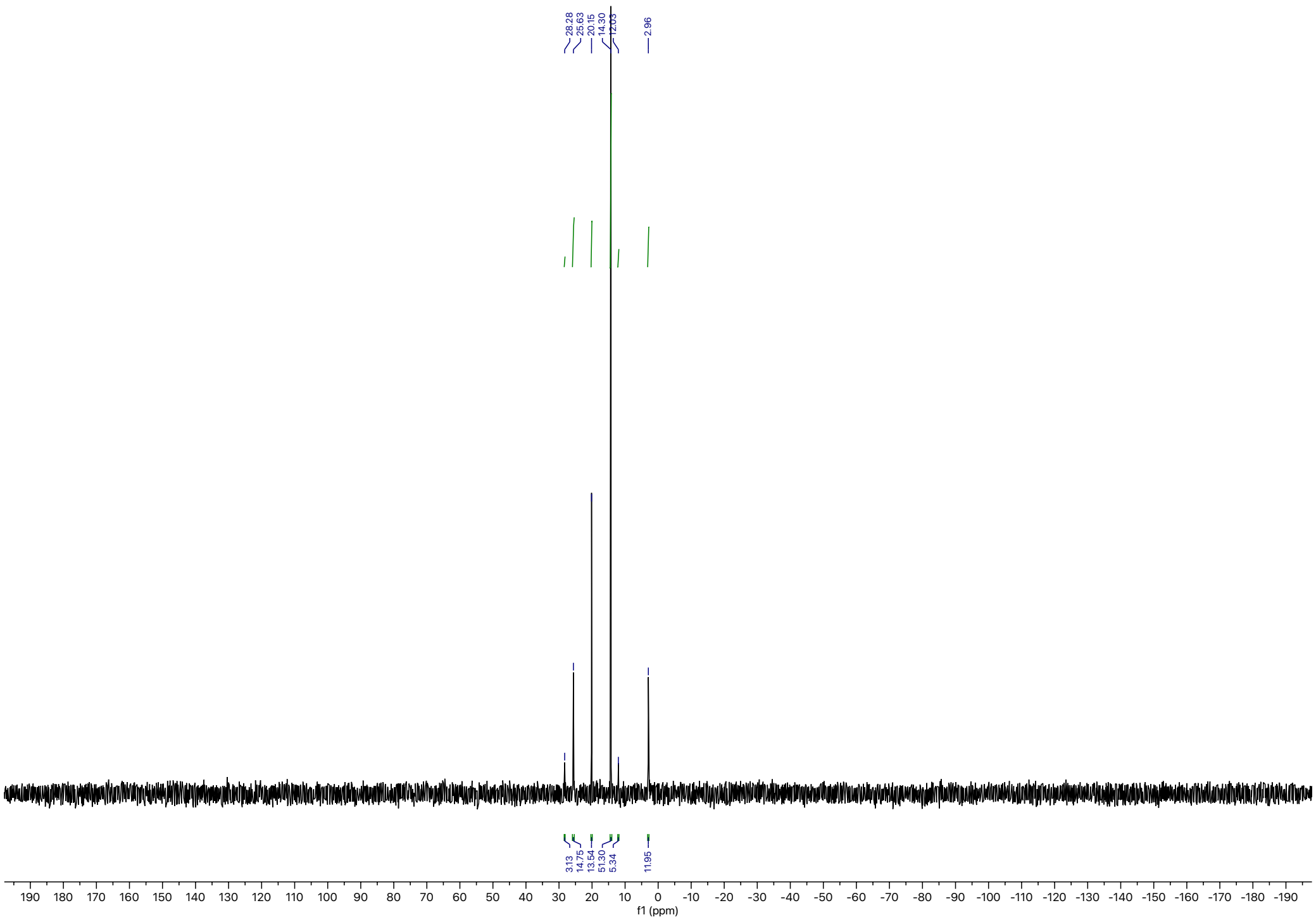

Figure S72. $^{31}P\{^{1}H\}$ NMR spectra of reaction $PtCl_2$ with PPh_3 (1:2) (**6**), after 15 minutes of milling (162.0 MHz, $CDCl_3$, 298K).

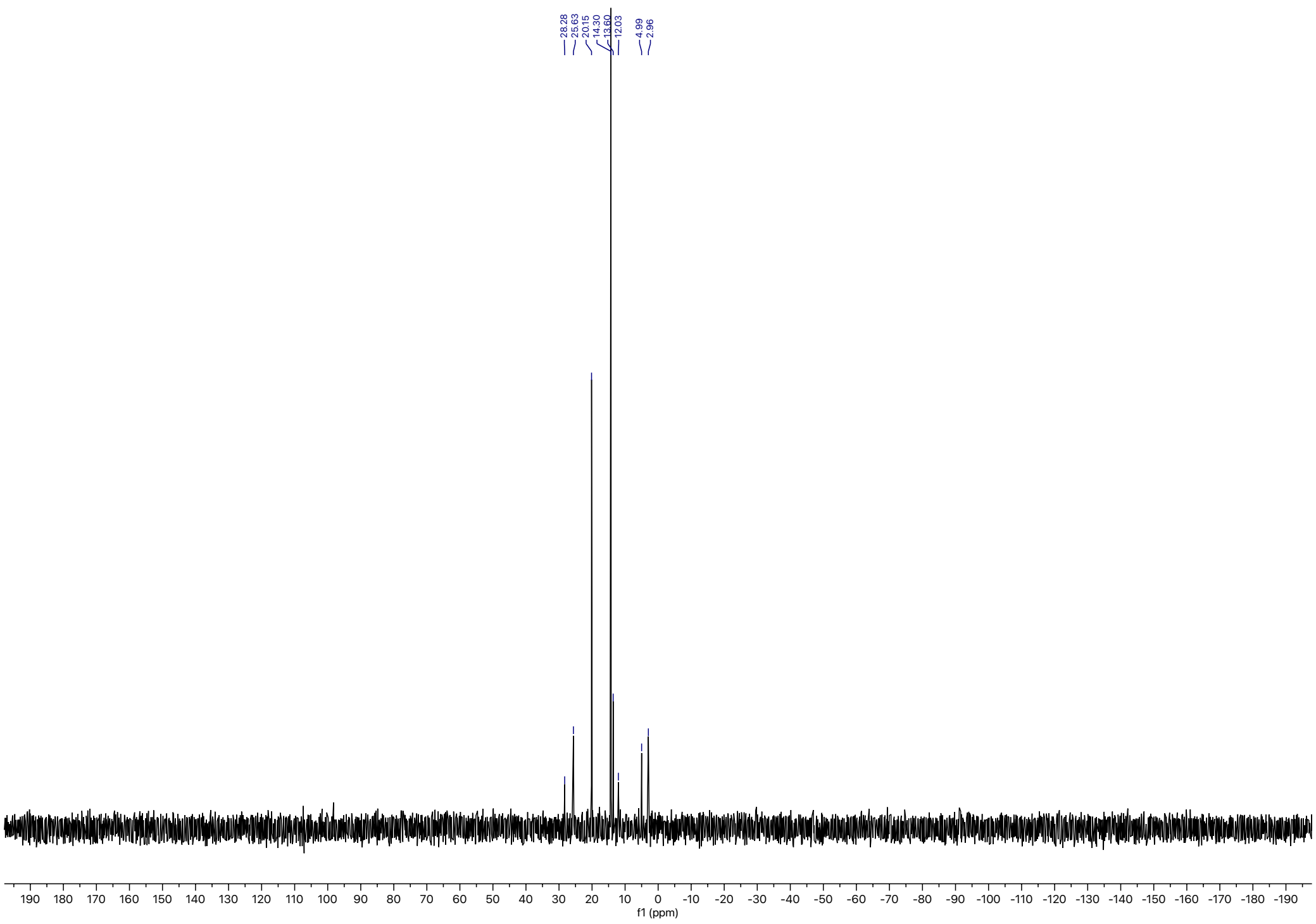

Figure S73. $^{31}P\{^{1}H\}$ NMR spectra of reaction $PtCl_2$ with PPh_3 (1:2) (**6**), after 30 minutes of milling (162.0 MHz, $CDCl_3$, 298K). Partial decomposition.

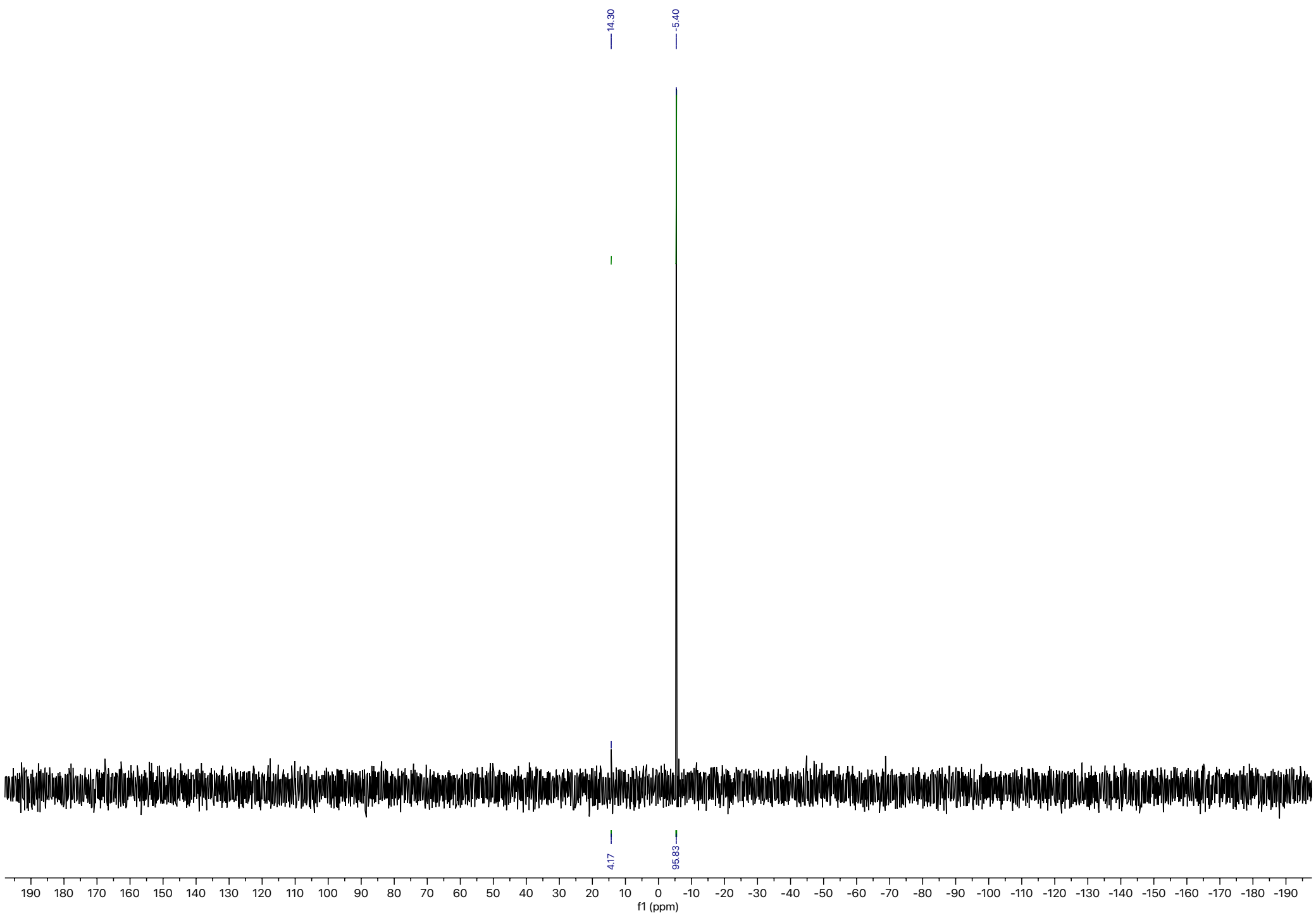

Figure S74. $^{31}P\{^{1}H\}$ NMR spectra of reaction $K_2[PtCl_4]$ with PPh_3 (1:2) (**6**), after 5 minutes of milling (162.0 MHz, $CDCl_3$, 298K).

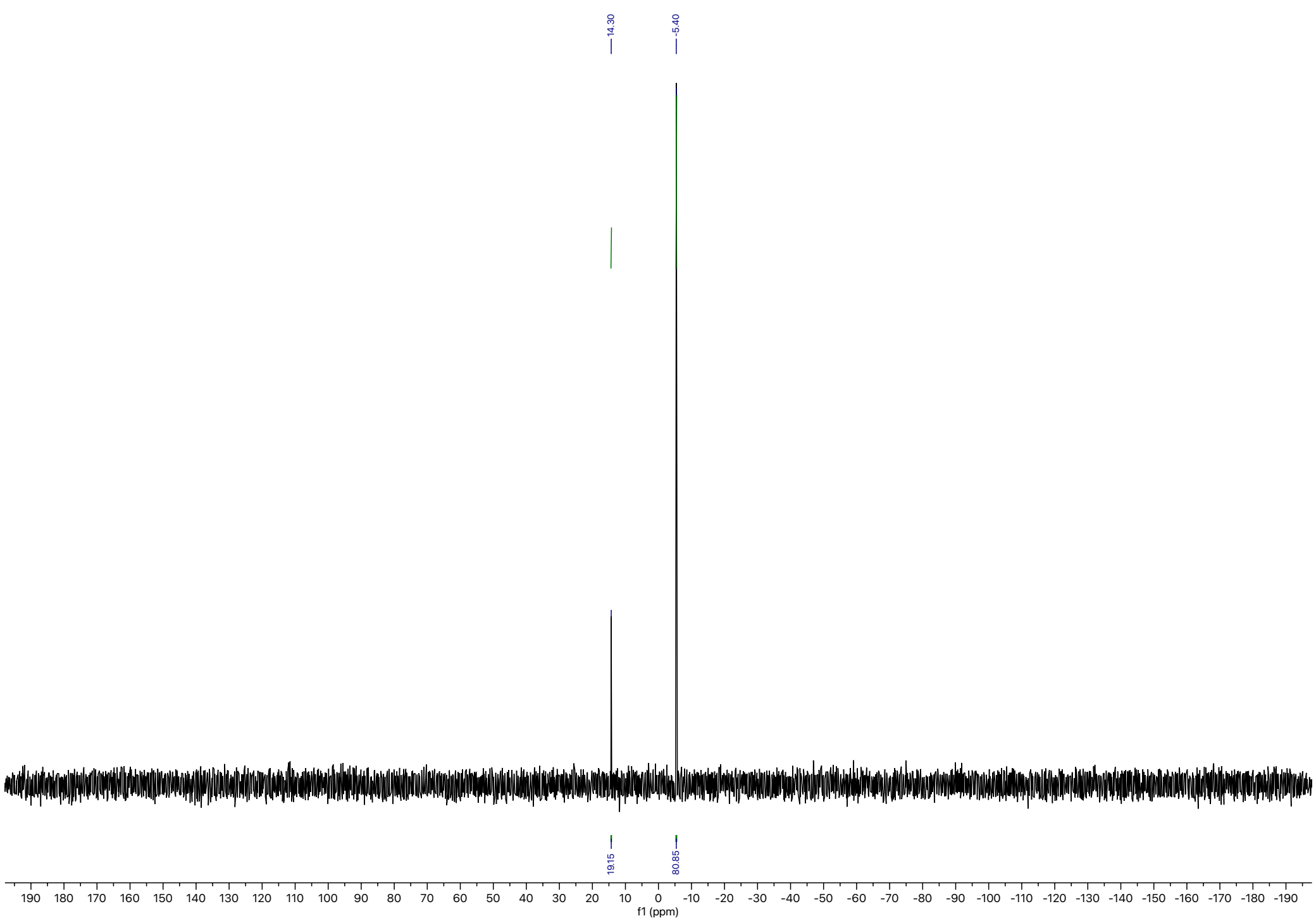

Figure S75. $^{31}P\{^{1}H\}$ NMR spectra of reaction $K_2[PtCl_4]$ with PPh_3 (1:2) (**6**), after 10 minutes of milling (162.0 MHz, $CDCl_3$, 298K).

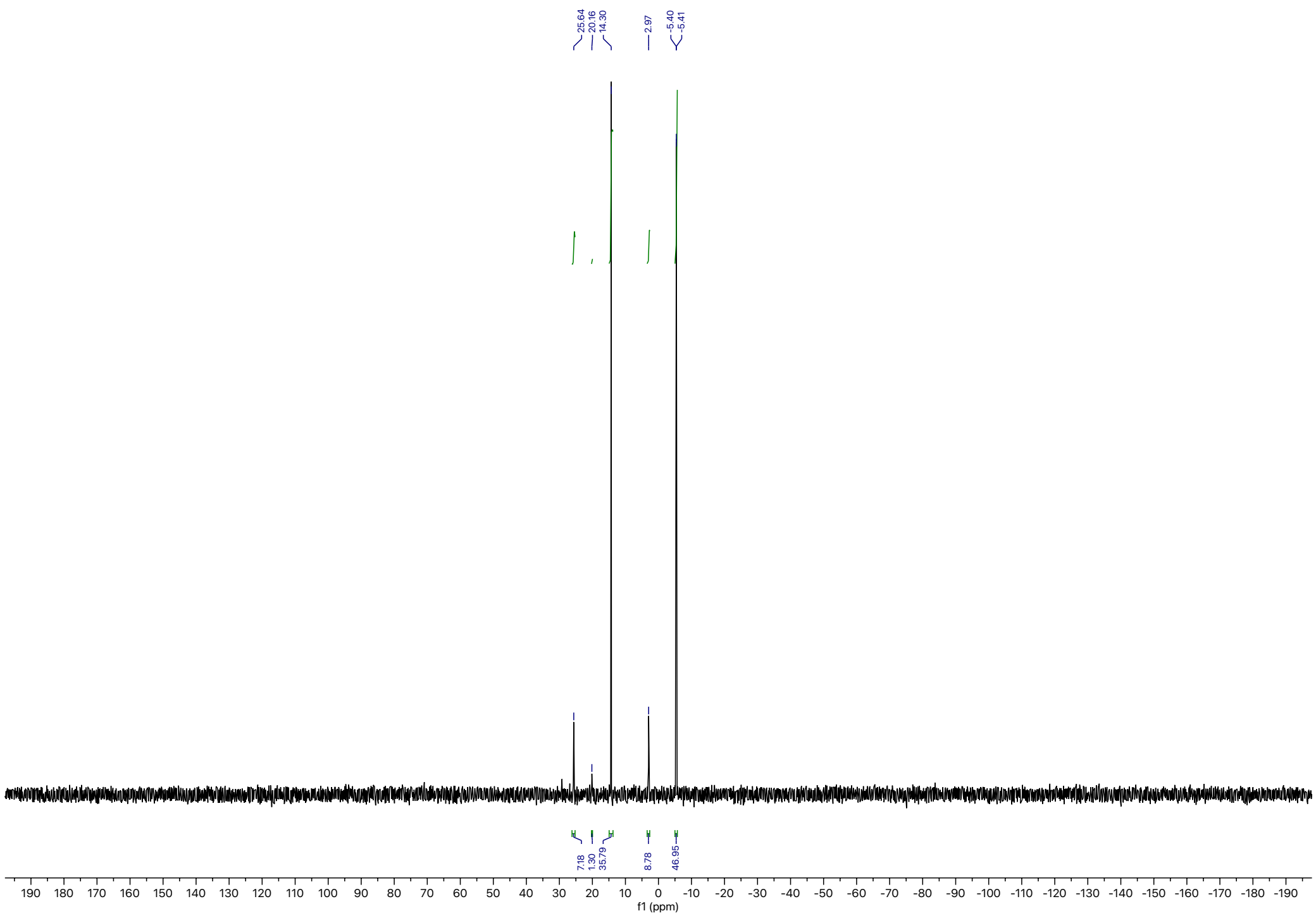

Figure S76. $^{31}P\{^1H\}$ NMR spectra of reaction $K_2[PtCl_4]$ with PPh_3 (1:2) (**6**), after 15 minutes of milling (162.0 MHz, $CDCl_3$, 298K).

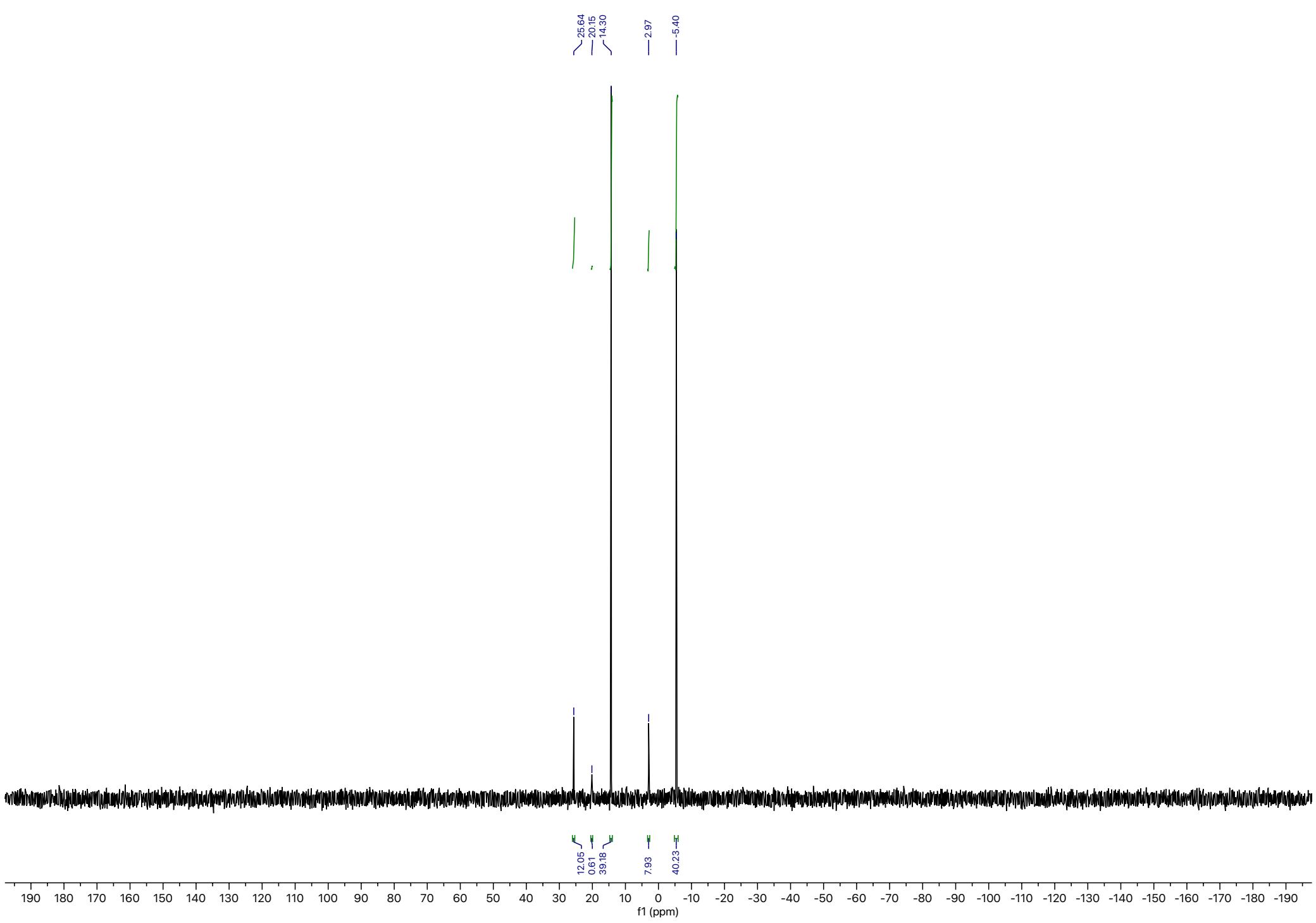

Figure S77. $^{31}P\{^1H\}$ NMR spectra of reaction $K_2[PtCl_4]$ with PPh_3 (1:2) (**6**), after 30 minutes of milling (162.0 MHz, $CDCl_3$, 298K).

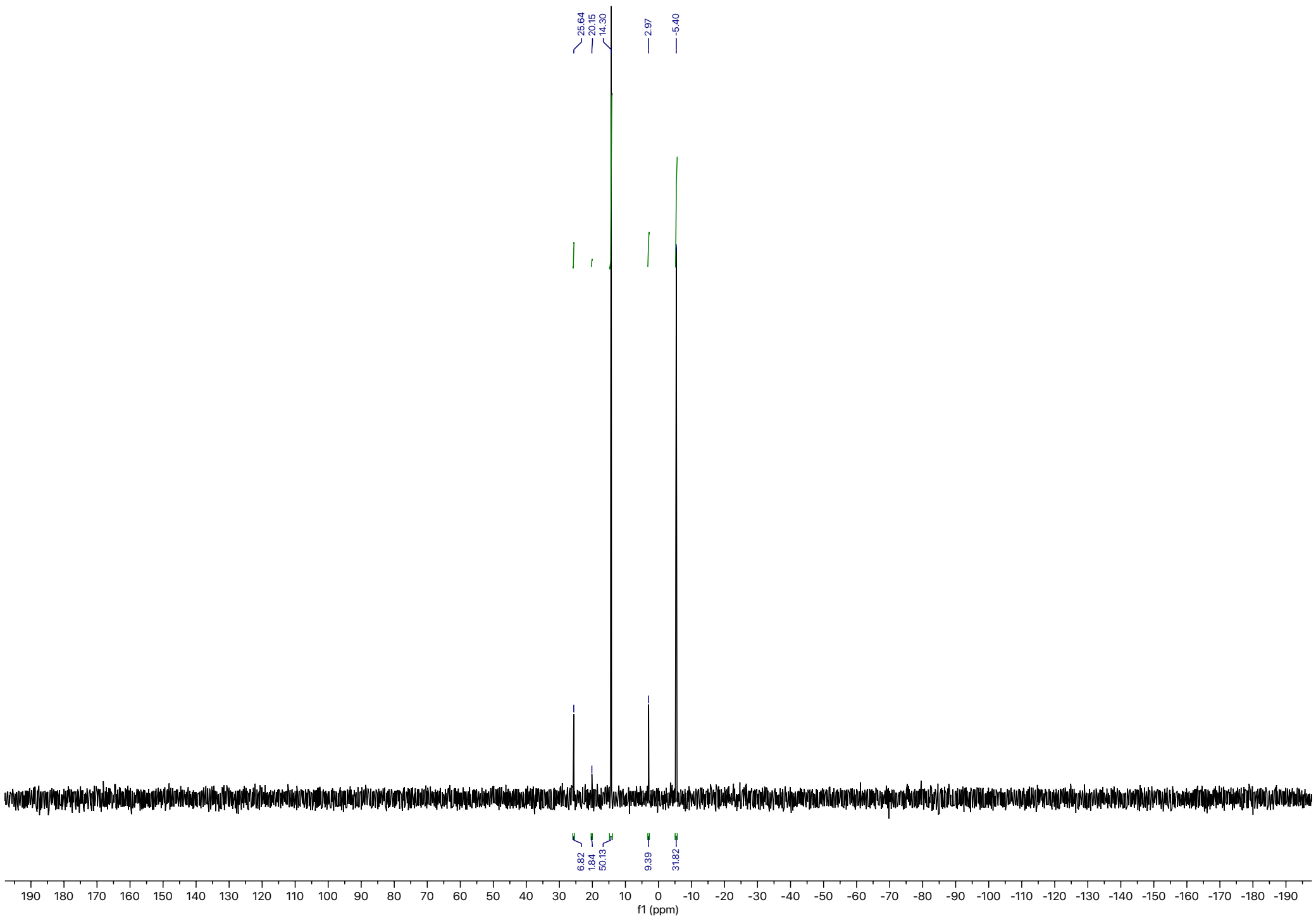

Figure S78. $^{31}P\{^1H\}$ NMR spectra of reaction $K_2[PtCl_4]$ with PPh_3 (1:2) (**6**), after 45 minutes of milling (162.0 MHz, $CDCl_3$, 298K).

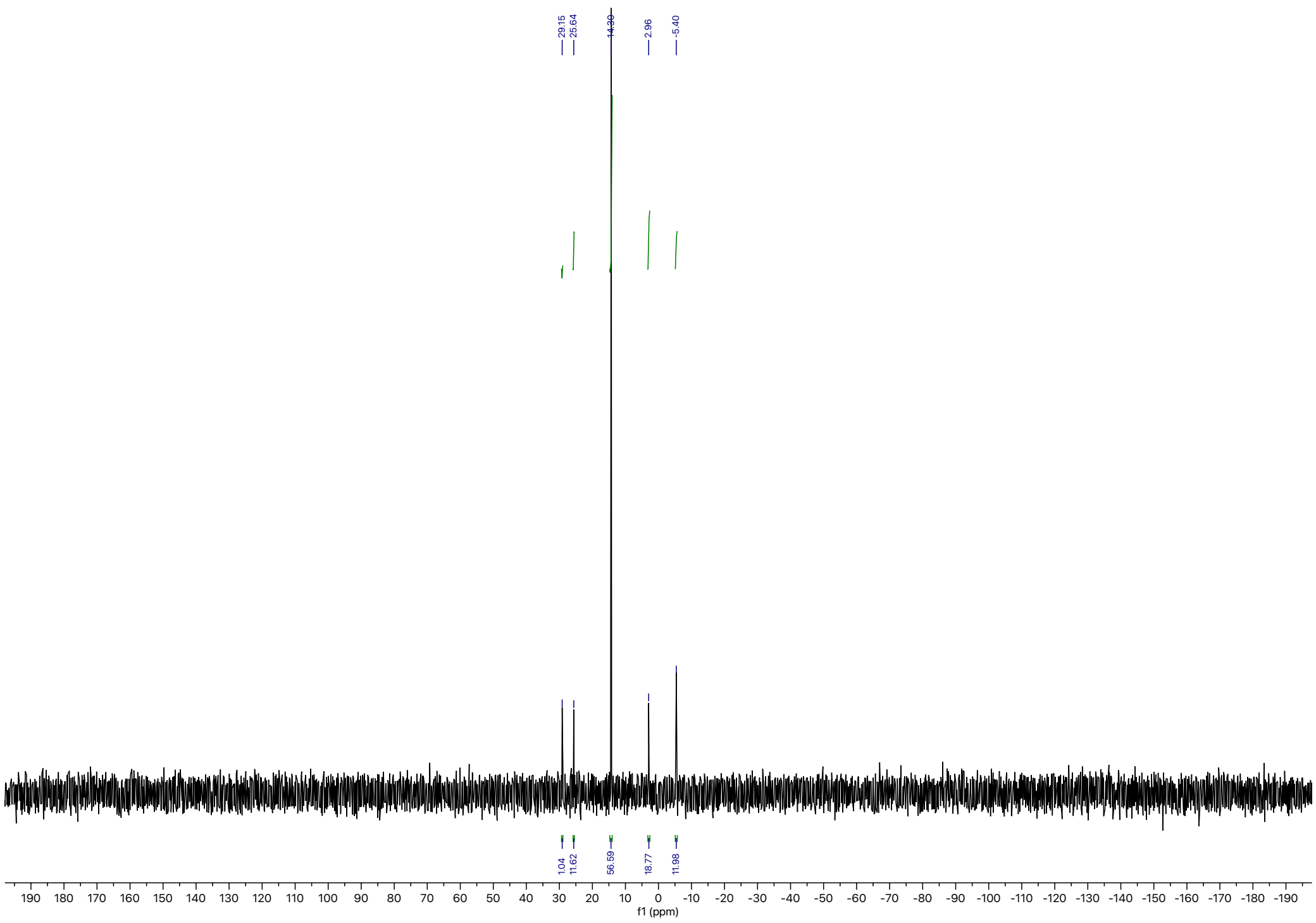

Figure S79. $^{31}P\{^1H\}$ NMR spectra of reaction $K_2[PtCl_4]$ with PPh_3 (1:2) (**6**), after 60 minutes of milling (162.0 MHz, $CDCl_3$, 298K).

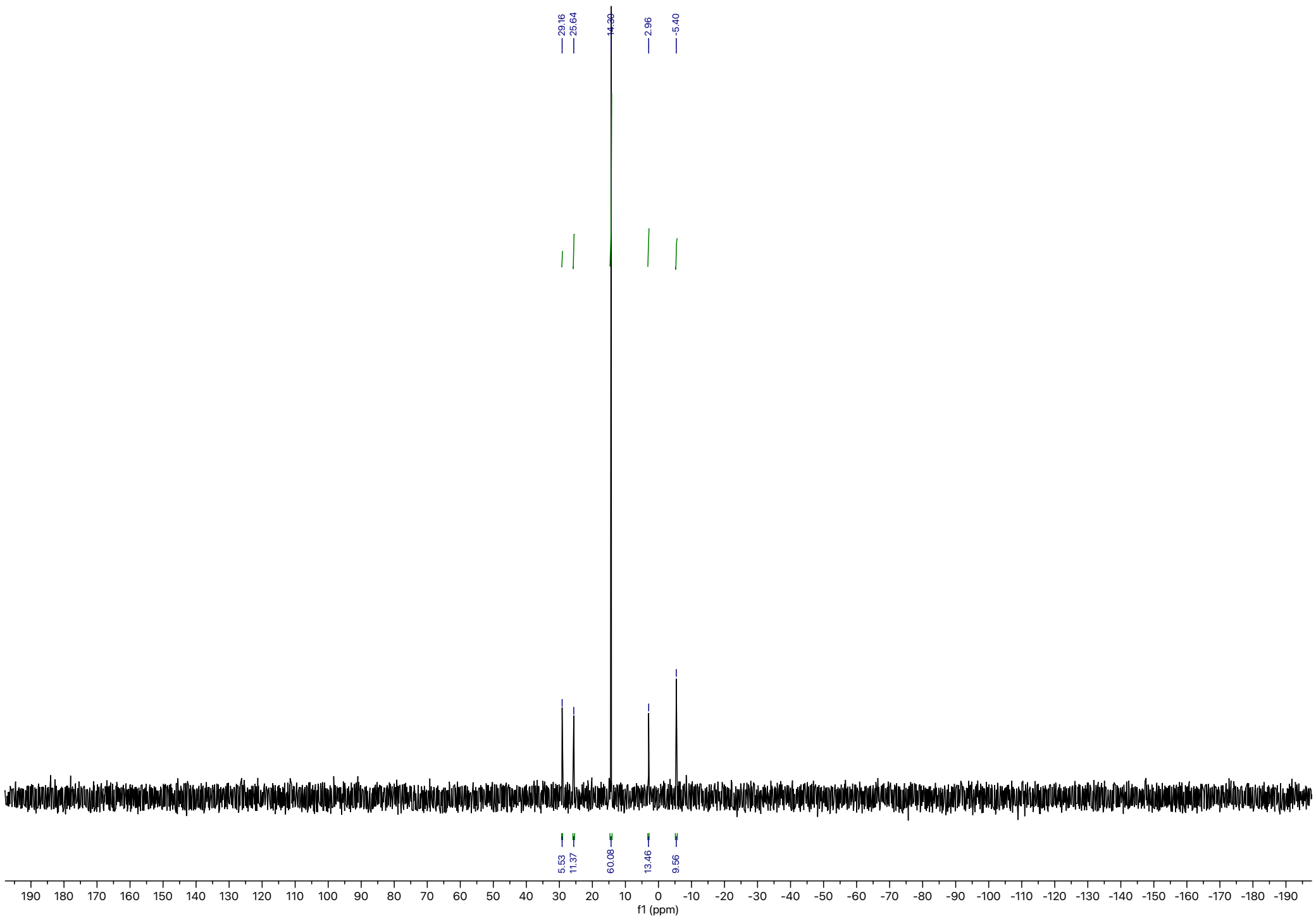

Figure S80. $^{31}P\{^{1}H\}$ NMR spectra of reaction $K_2[PtCl_4]$ with PPh_3 (1:2) (**6**), after 90 minutes of milling (162.0 MHz, $CDCl_3$, 298K).

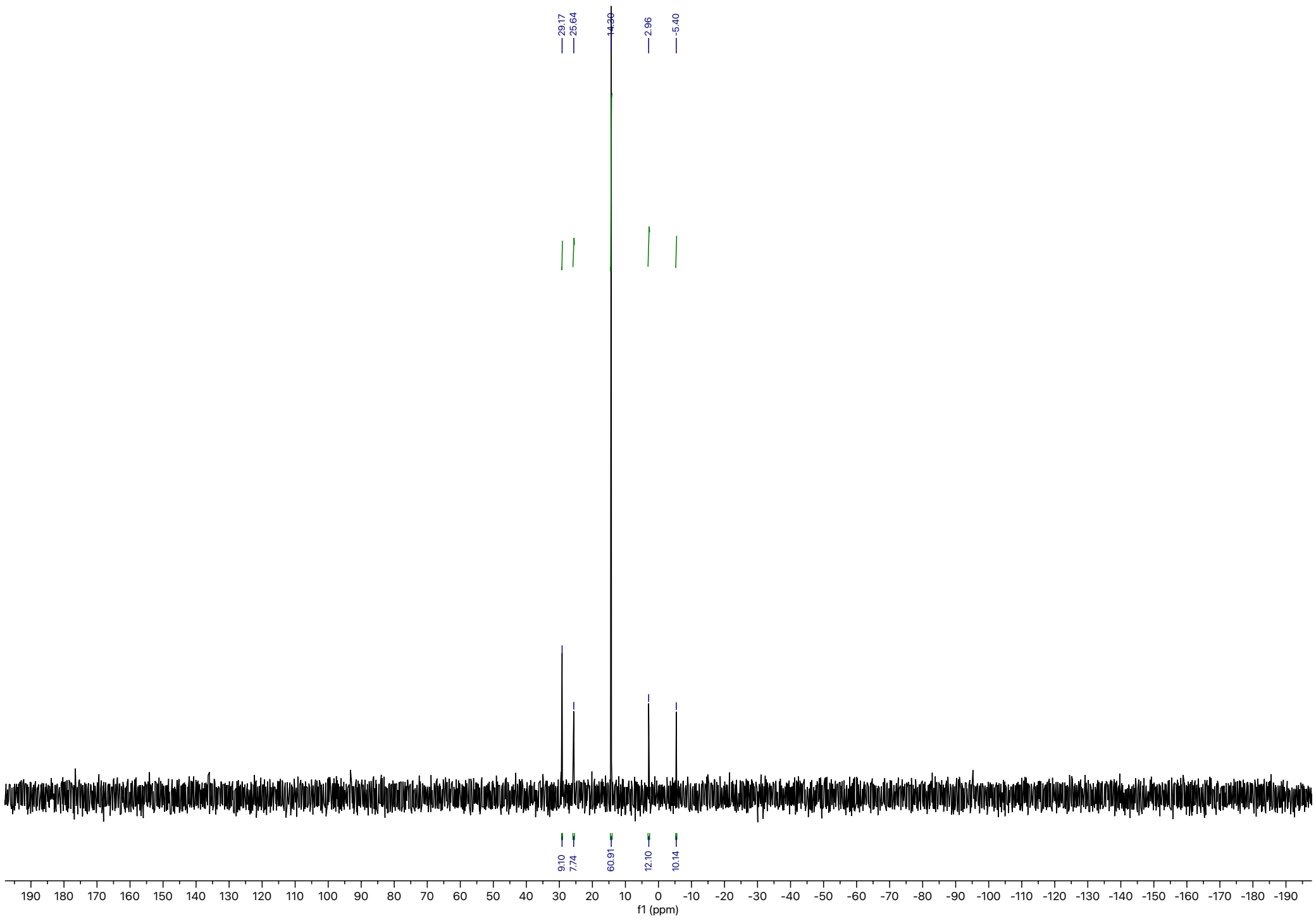

Figure S81. $^{31}P\{^{1}H\}$ NMR spectra of reaction $K_2[PtCl_4]$ with PPh_3 (1:2) (**6**), after 120 minutes of milling (162.0 MHz, $CDCl_3$, 298K).

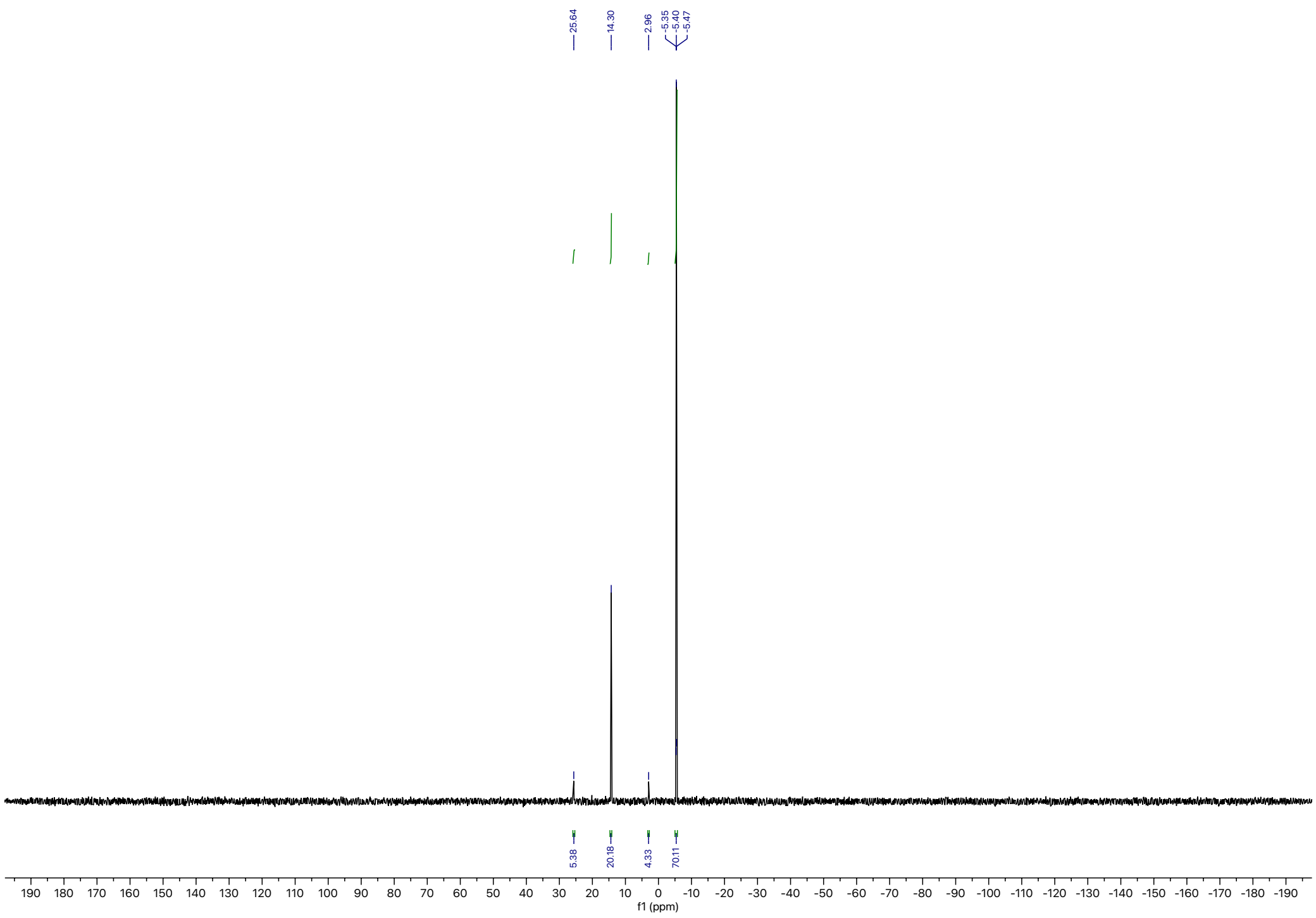

Figure S82. $^{31}P\{^1H\}$ NMR spectra of tube reaction $PtCl_2$ with PPh_3 (1:2) (**6**), after 5 minutes in the tube (162.0 MHz, $CDCl_3$, 298K).

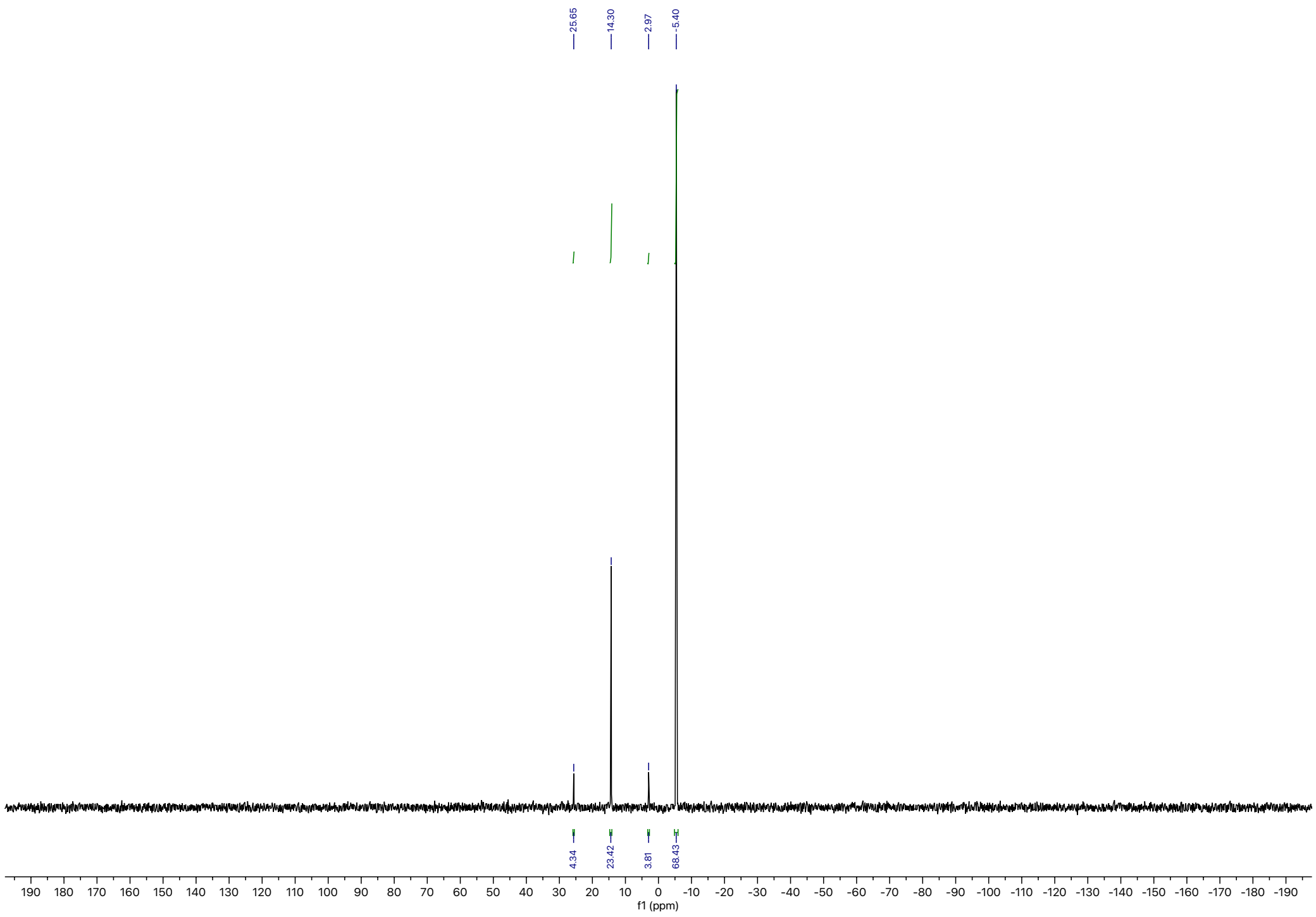

Figure S83. $^{31}P\{^1H\}$ NMR spectra of tube reaction $PtCl_2$ with PPh_3 (1:2) (**6**), after 60 minutes in the tube (162.0 MHz, $CDCl_3$, 298K).

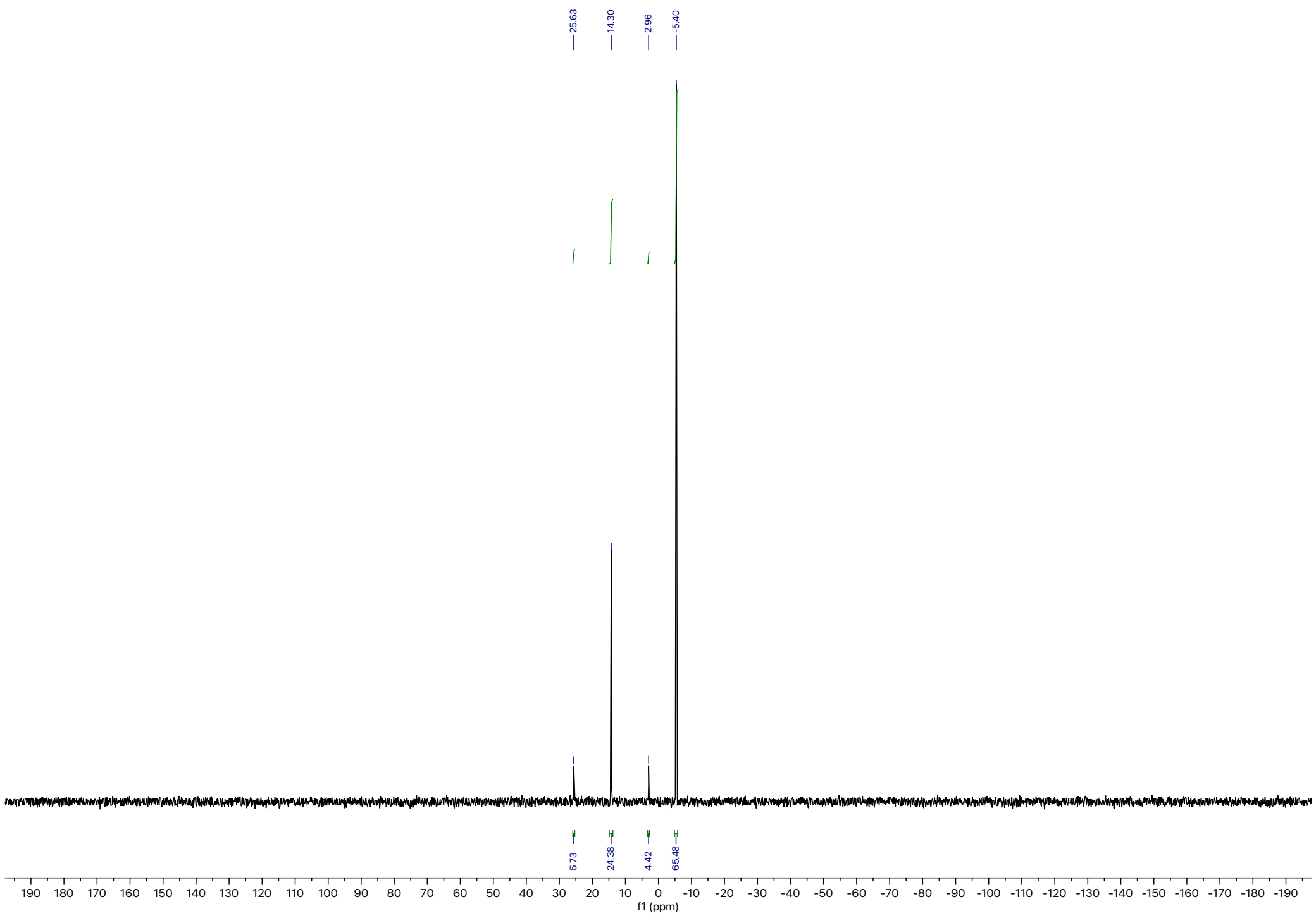

Figure S84. $^{31}P\{^1H\}$ NMR spectra of tube reaction $PtCl_2$ with PPh_3 (1:2) (**6**), after 120 minutes in the tube (162.0 MHz, $CDCl_3$, 298K).

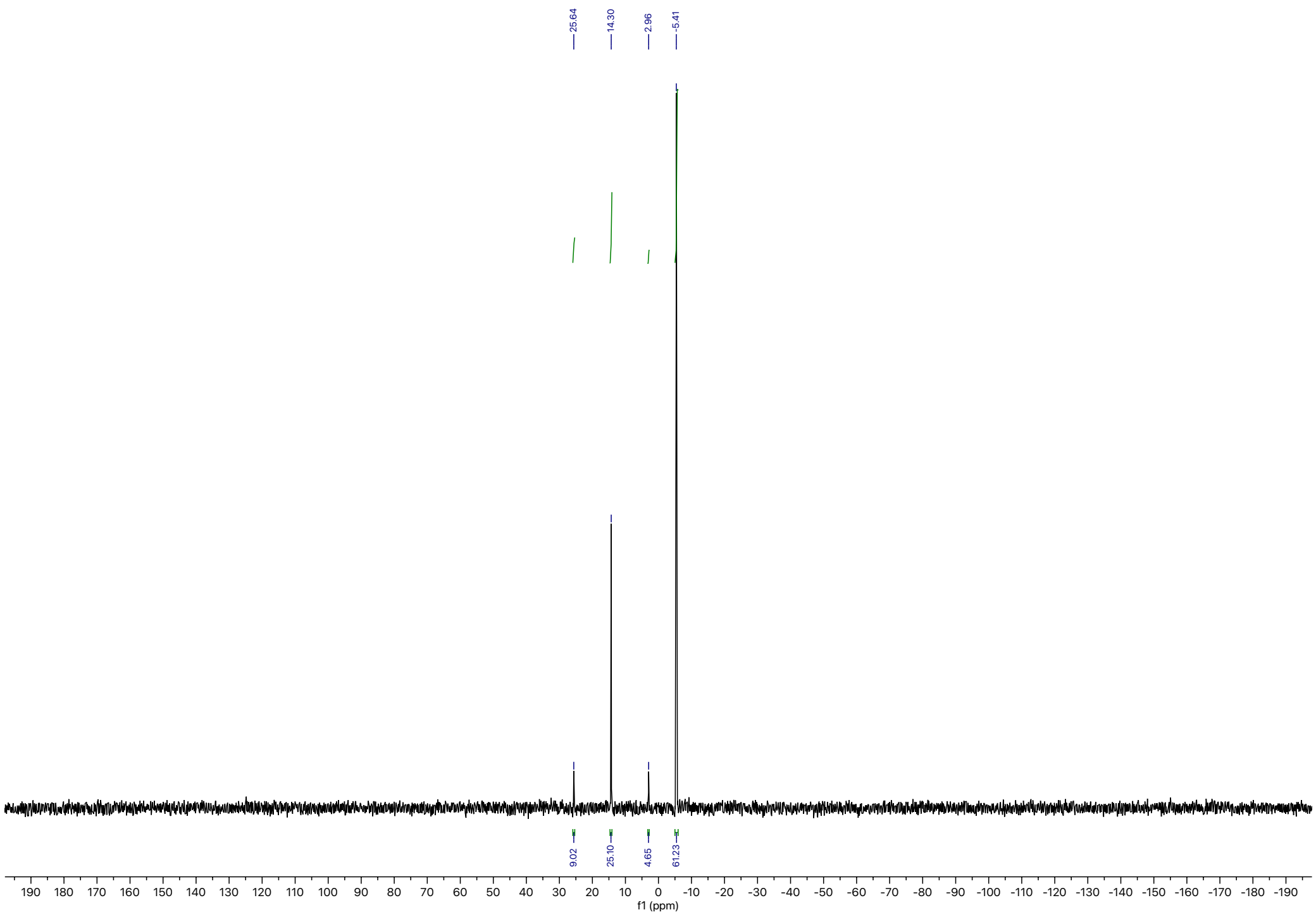

Figure S85. $^{31}P\{^1H\}$ NMR spectra of tube reaction $PtCl_2$ with PPh_3 (1:2) (**6**), after 8 hours in the tube (162.0 MHz, $CDCl_3$, 298K).

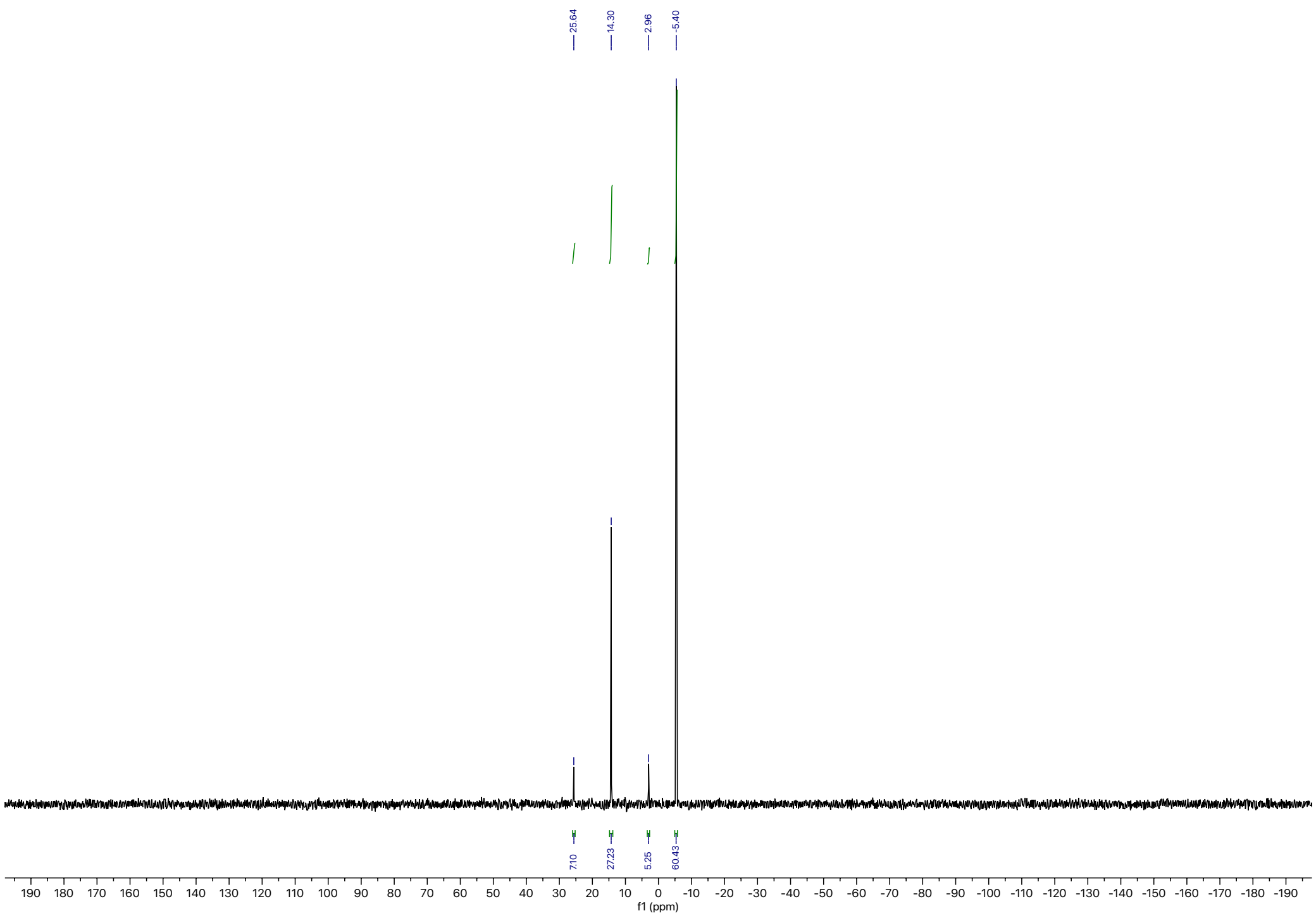

Figure S86. $^{31}P\{^{1}H\}$ NMR spectra of tube reaction $PtCl_2$ with PPh_3 (1:2) (**6**), after 1 day in the tube (162.0 MHz, $CDCl_3$, 298K).

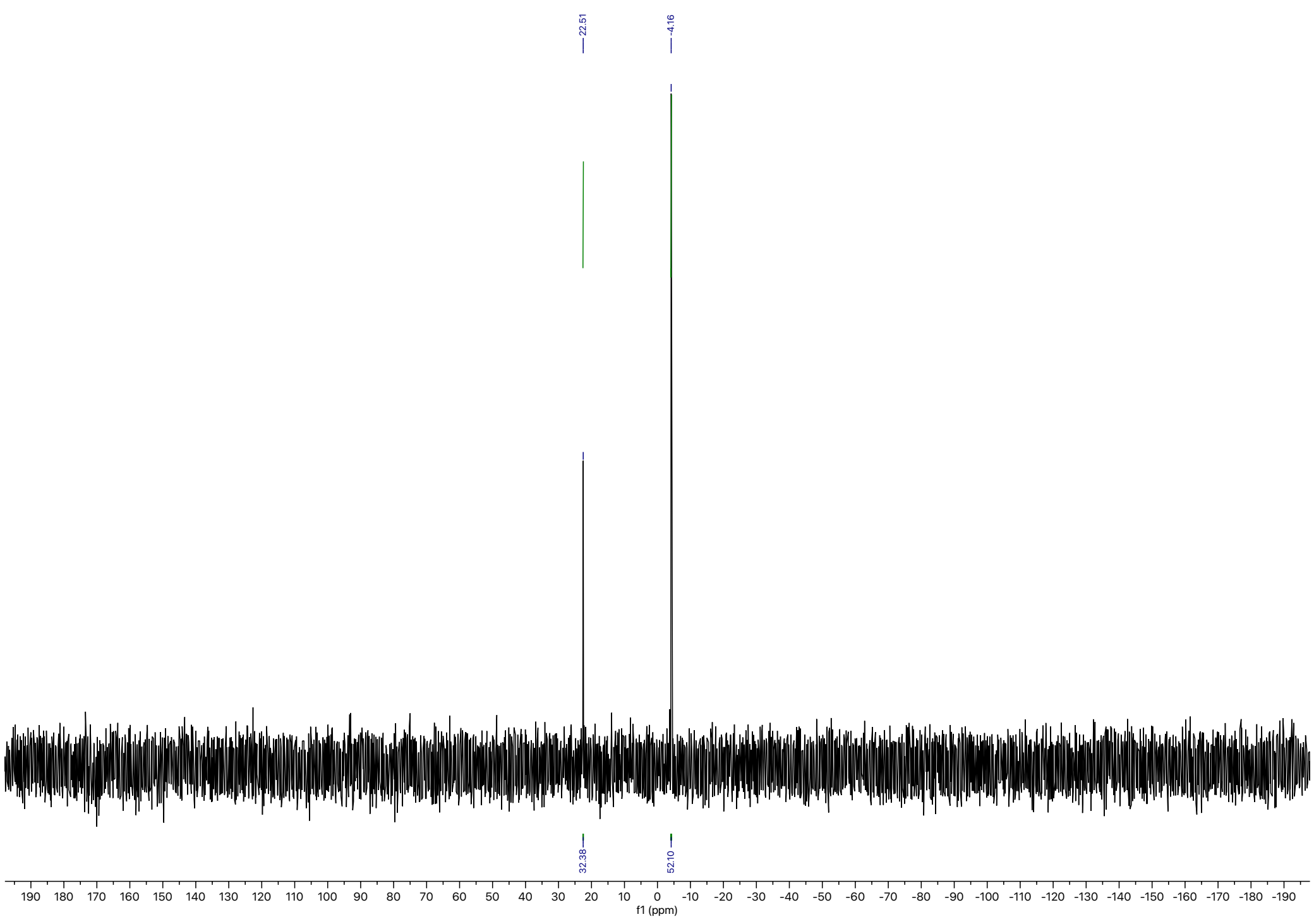

Figure S87. $^{31}P\{^{1}H\}$ NMR spectra of reaction $K_2[PtCl_4]$ with $P(3,5\text{-}(CF_3)_2Ph)_3$ (1:2) (**7**), after 120 minutes of milling (162.0 MHz, $CDCl_3$, 298K). $\chi_{(120)}$ = 32%.

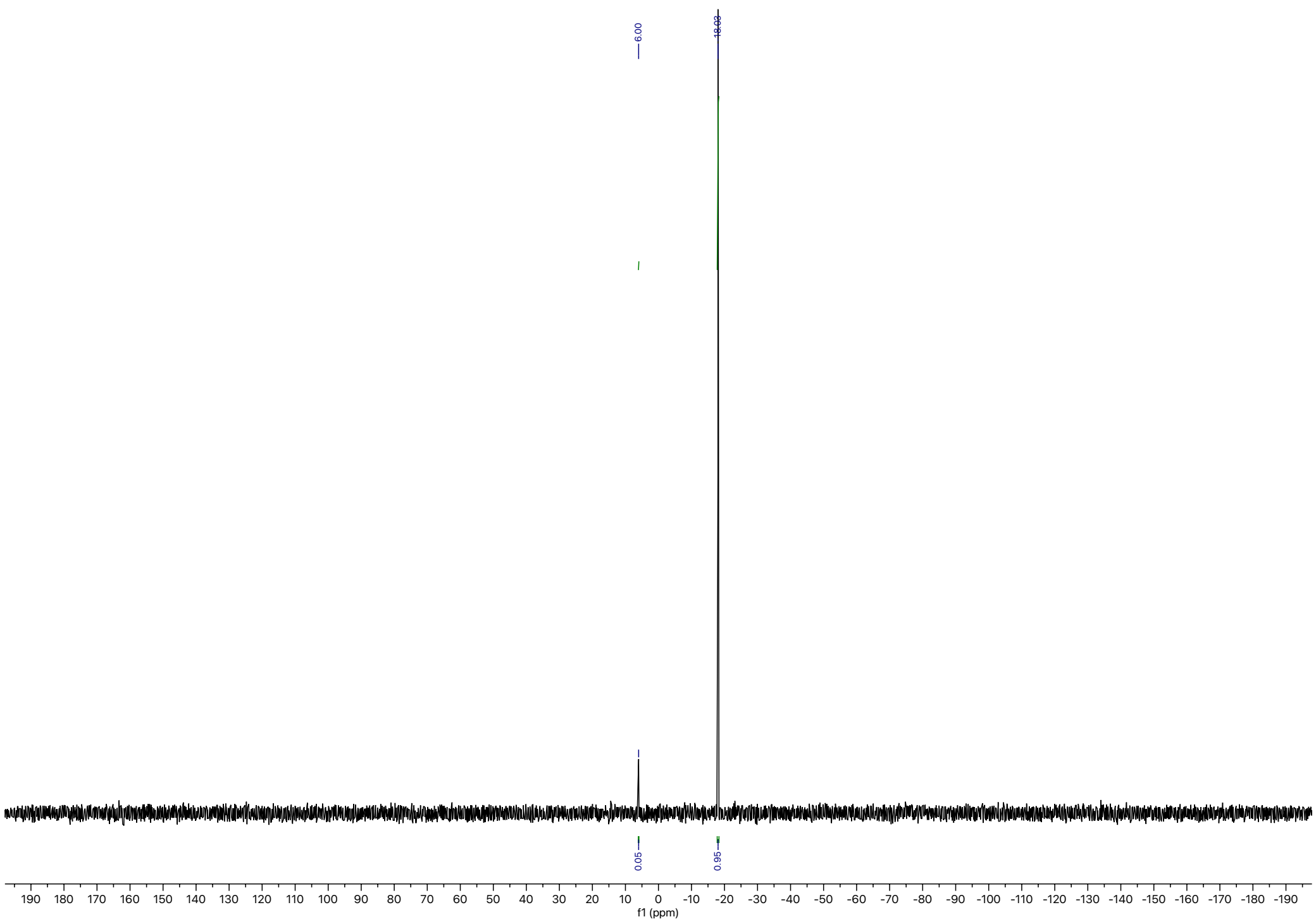

Figure S88. $^{31}P\{^1H\}$ NMR spectra of reaction $K_2[PtCl_4]$ with Xantphos (1:1) (**8**), after 120 minutes of milling (162.0 MHz, $CDCl_3$, 298K). $\chi_{(240)}$ = 5%.

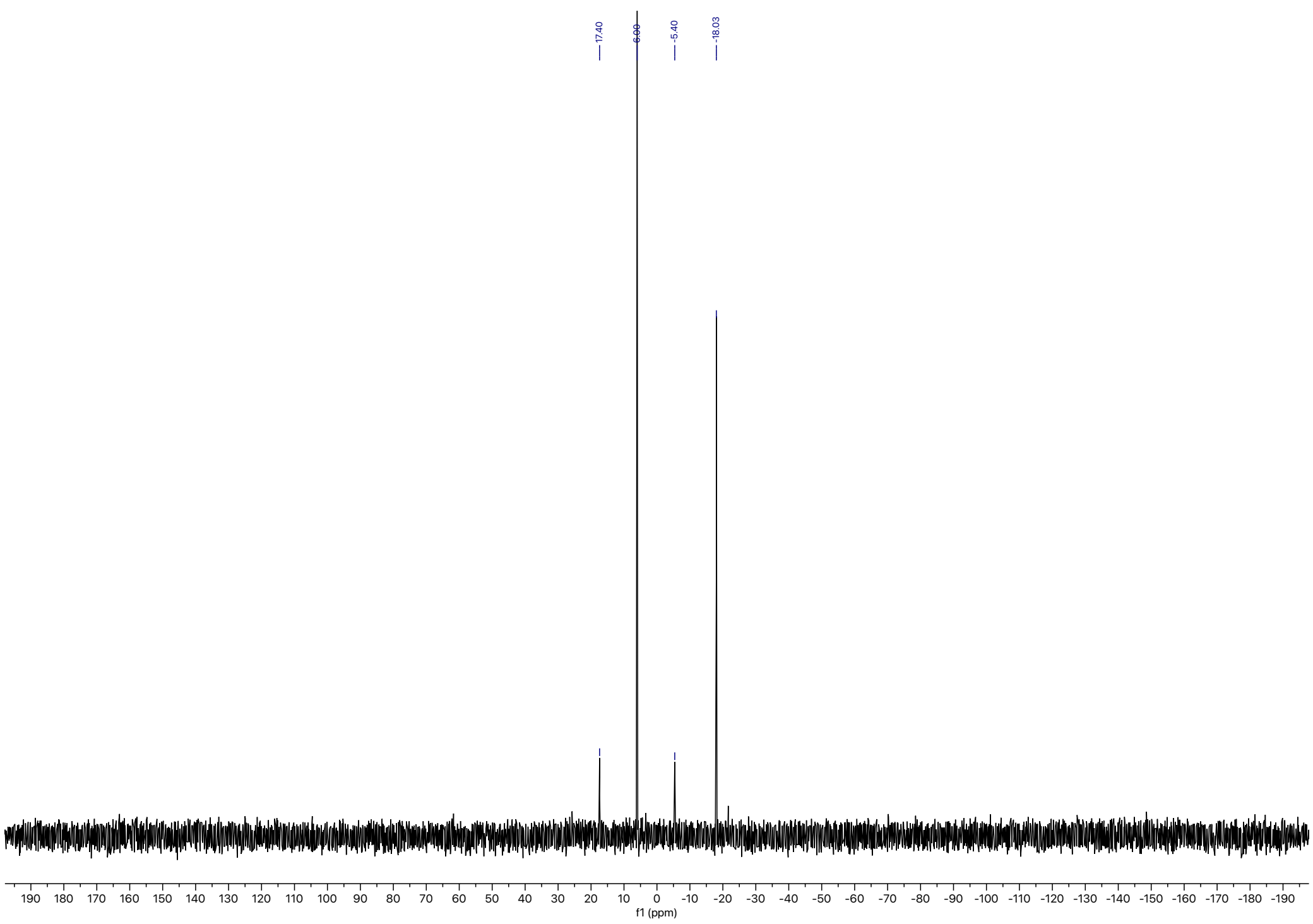

Figure S89. $^{31}P\{^1H\}$ NMR spectra of reaction $K_2[PtCl_4]$ with Xantphos (1:1) (**8**), after 120 minutes of milling in SSjar (162.0 MHz, $CDCl_3$, 298K). $\chi_{(240)}$ = 57%.

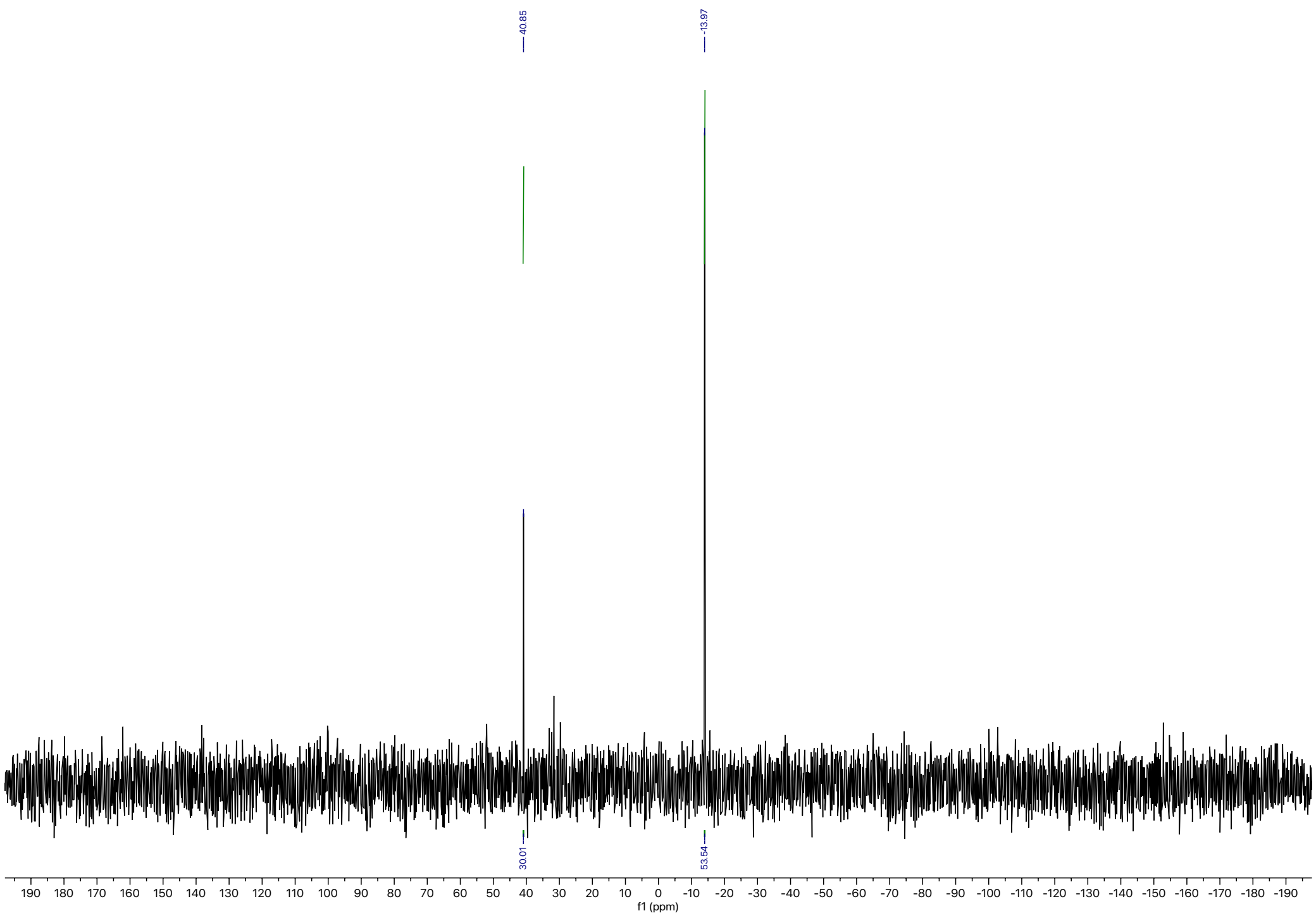

Figure S90. $^{31}P\{^{1}H\}$ NMR spectra of $K_2[PtCl_4]$ + dppbz (**13**) (162.0 MHz, $CDCl_3$, 298K). $\chi_{(120)}$ = 30%.

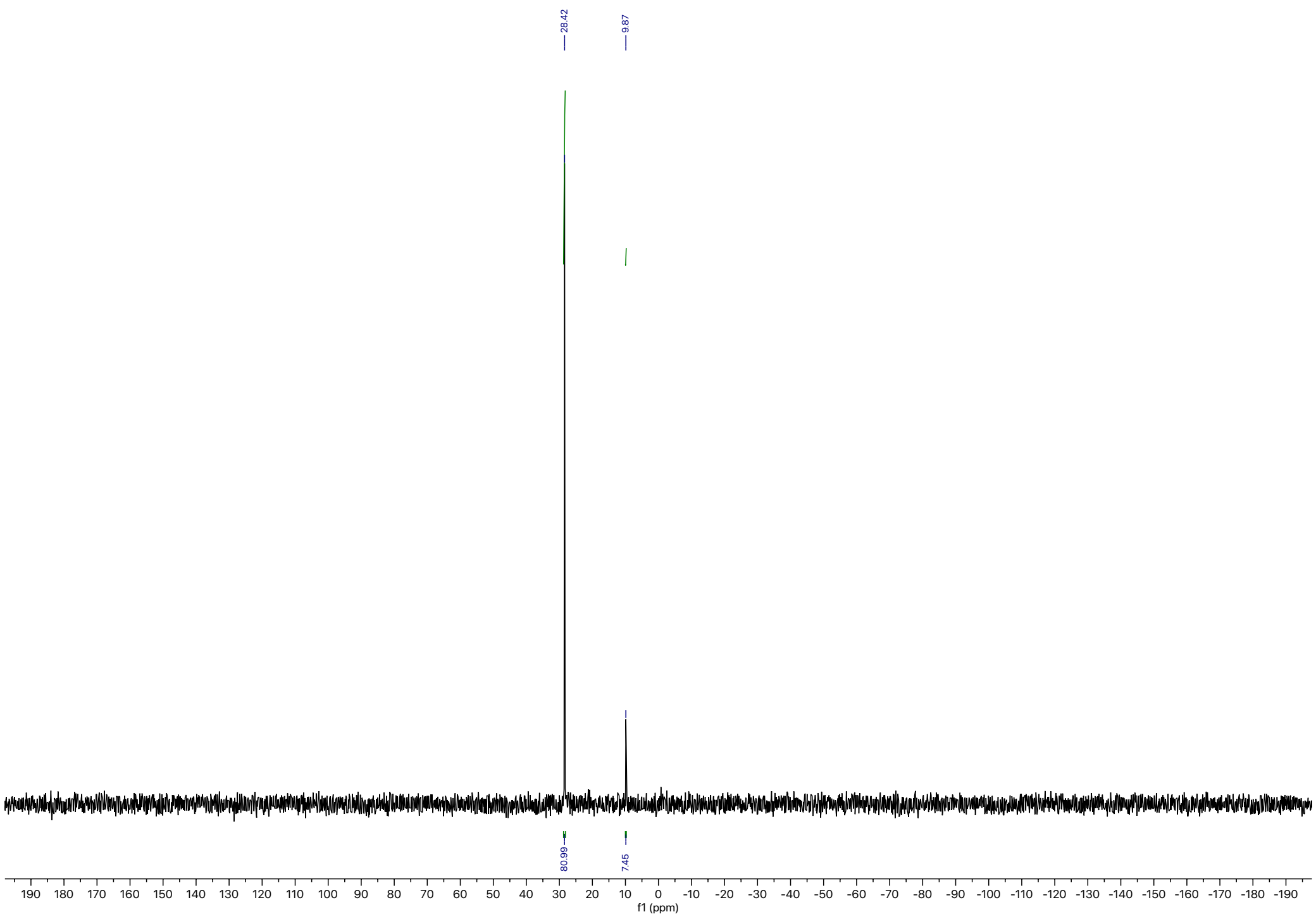

Figure S91. $^{31}P\{^{1}H\}$ NMR spectra of $K_2[PtCl_4]$ + (*R*)-BINAP (**16**) (162.0 MHz, $CDCl_3$, 298K).

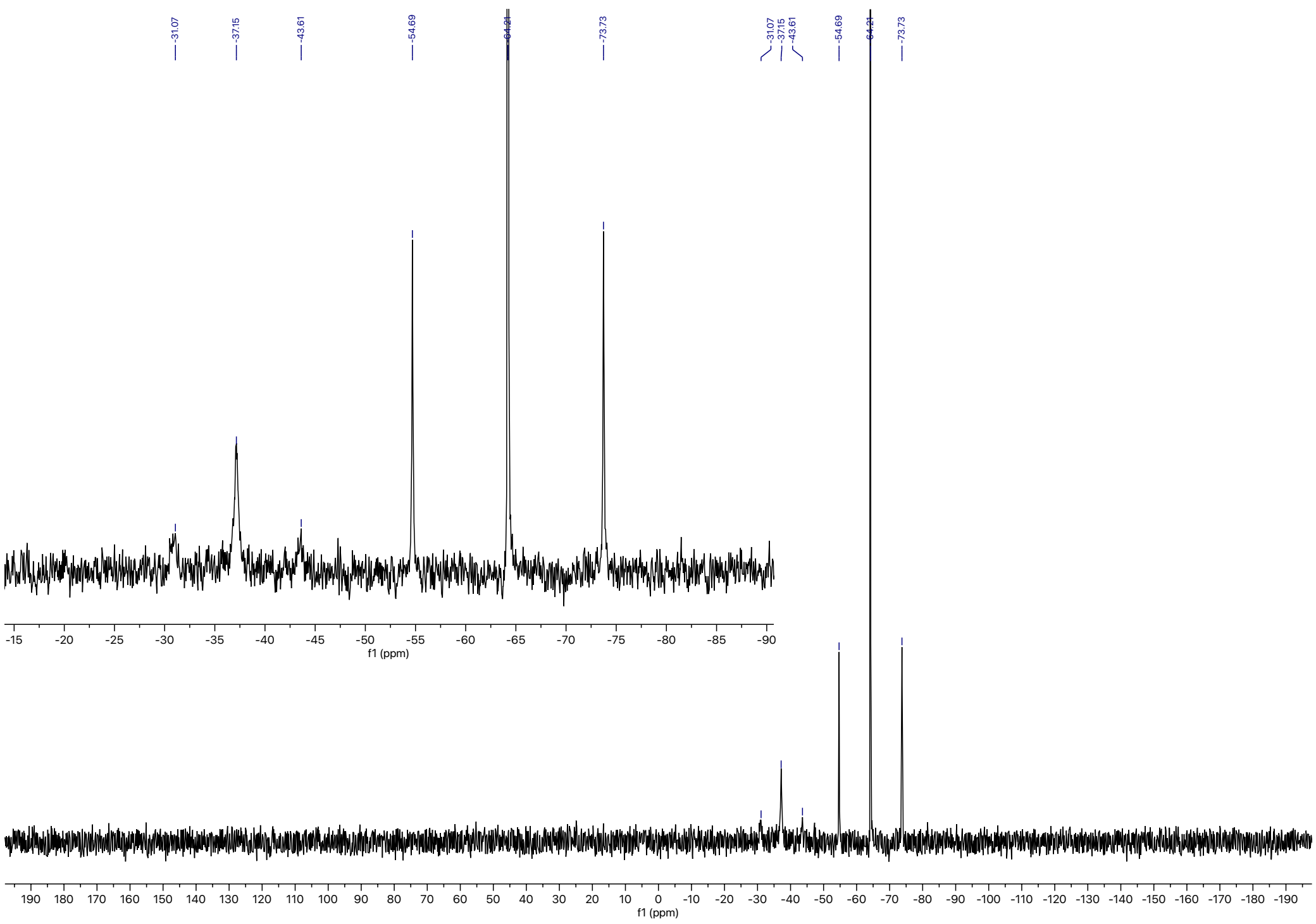

Figure S92. $^{31}P\{^{1}H\}$ NMR spectra of $PtCl_2$ + 1.2 equiv. dppm (**9**) (162.0 MHz, $CDCl_3$, 298K).

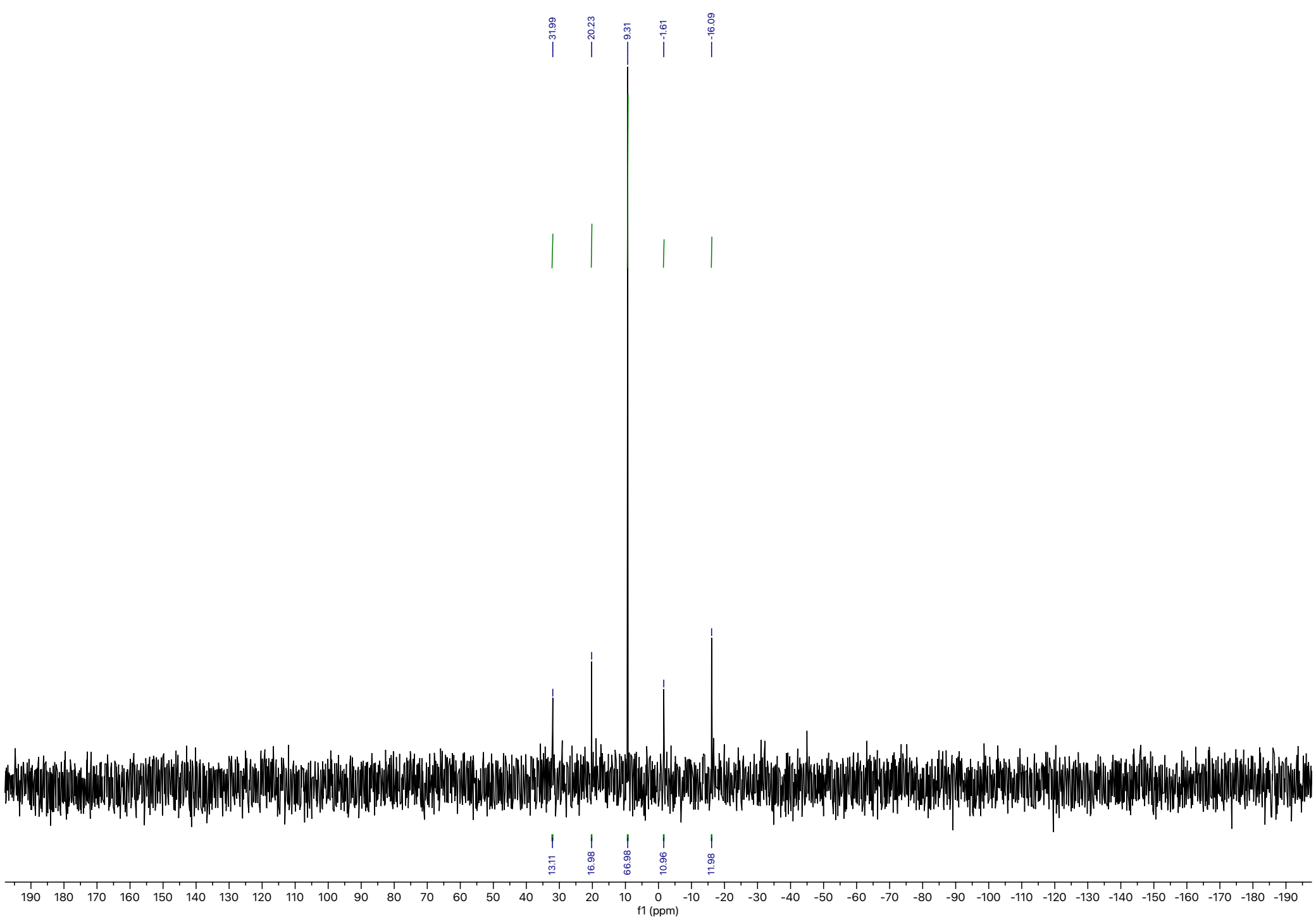

Figure S93. $^{31}P\{^{1}H\}$ NMR spectra of $PtCl_2$ + 1.2 equiv. dppb (**12**) (162.0 MHz, $CDCl_3$, 298K).

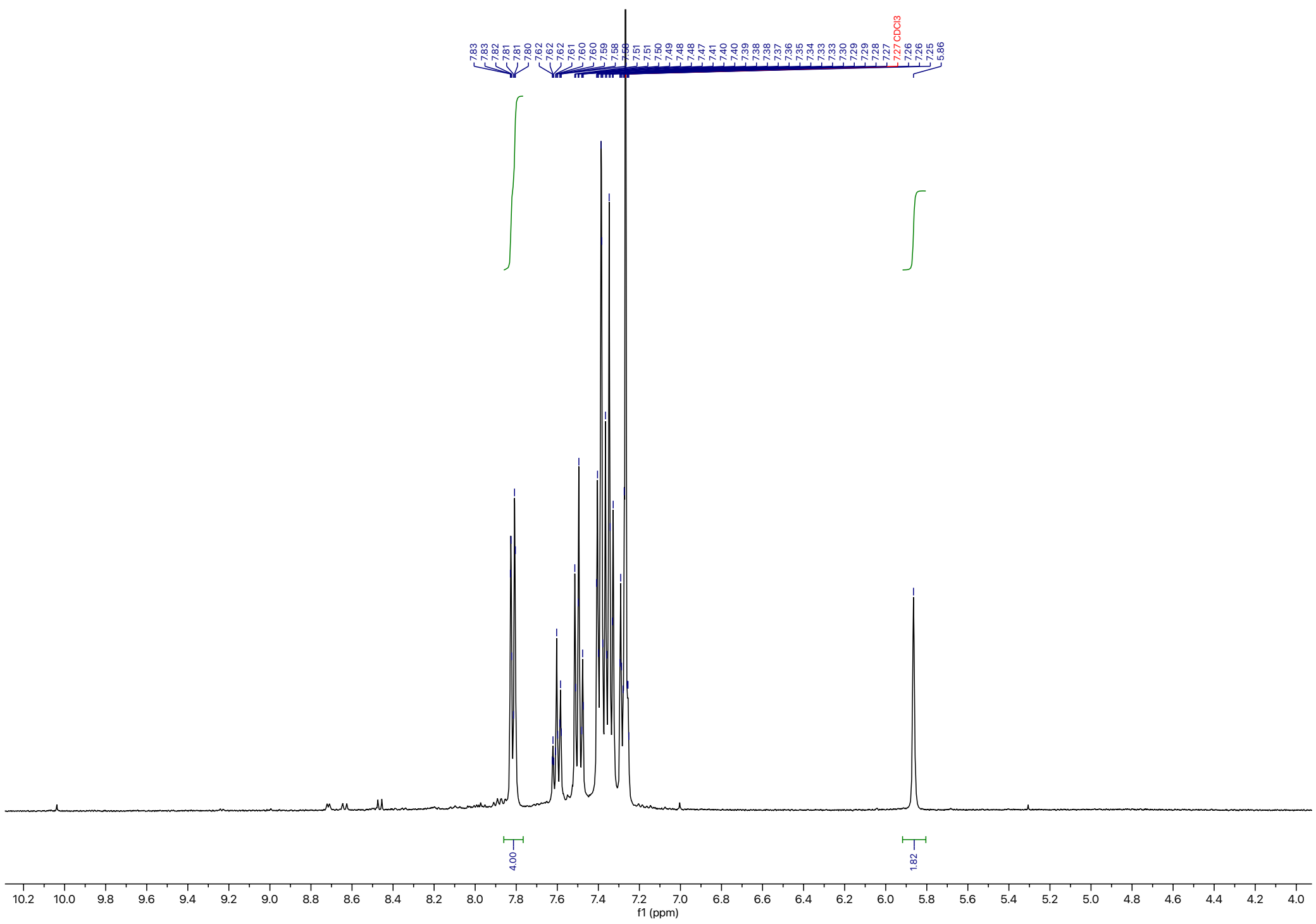

Figure S94. ^{1}H NMR spectra of [Pt(κ^2-terpy)Cl_2] (**4**) with 25 mg of diphenylmethanol, 250 mg of Oxone® and 150 mg of Na_3PO_4 (400.1 MHz, $CDCl_3$, 298K).

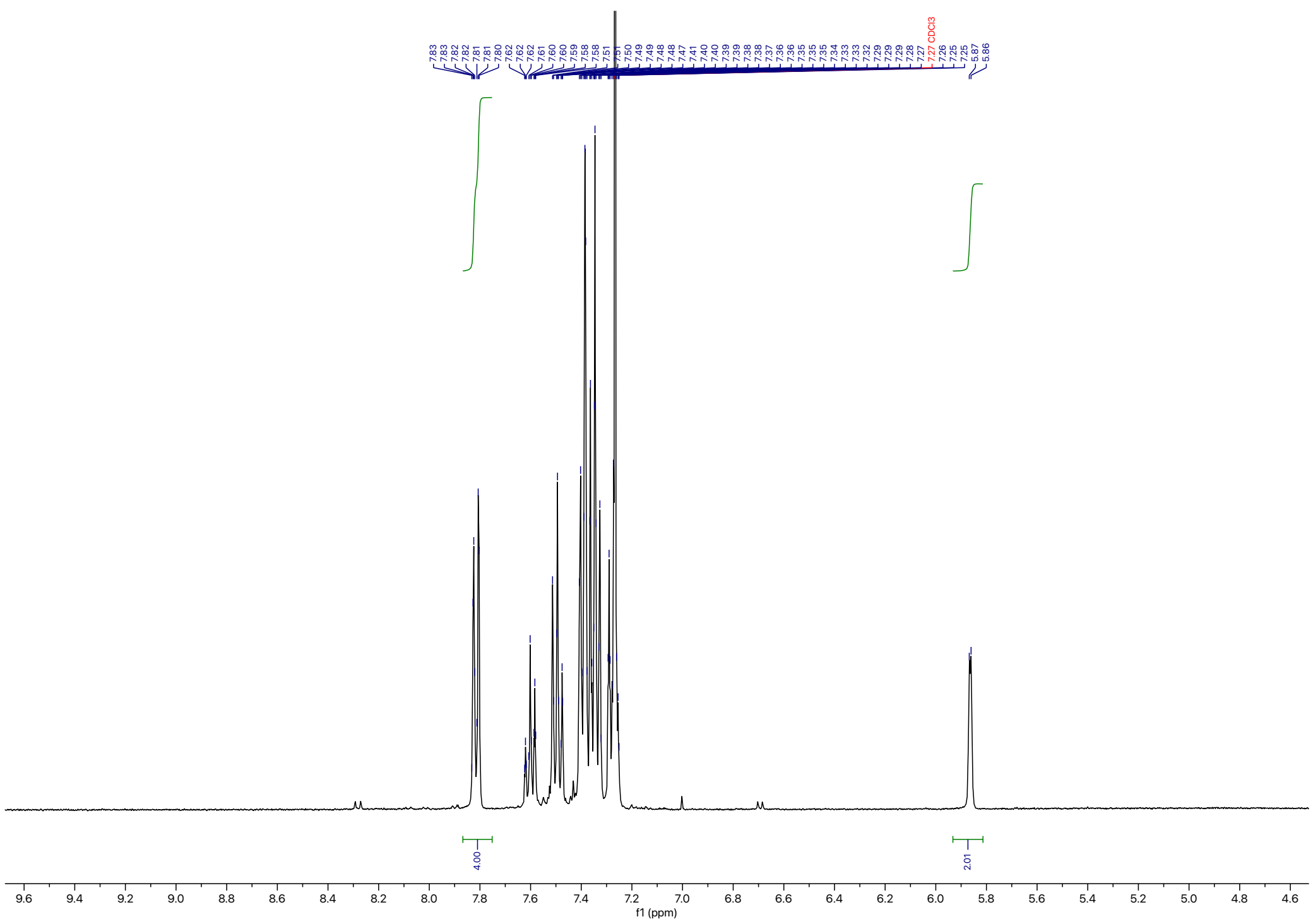

Figure S95. ^{1}H NMR spectra of [Pt(BIAN)Cl_2] (**5**) with 25 mg of diphenylmethanol, 250 mg of Oxone® and 150 mg of Na_3PO_4 (400.1 MHz, $CDCl_3$, 298K).

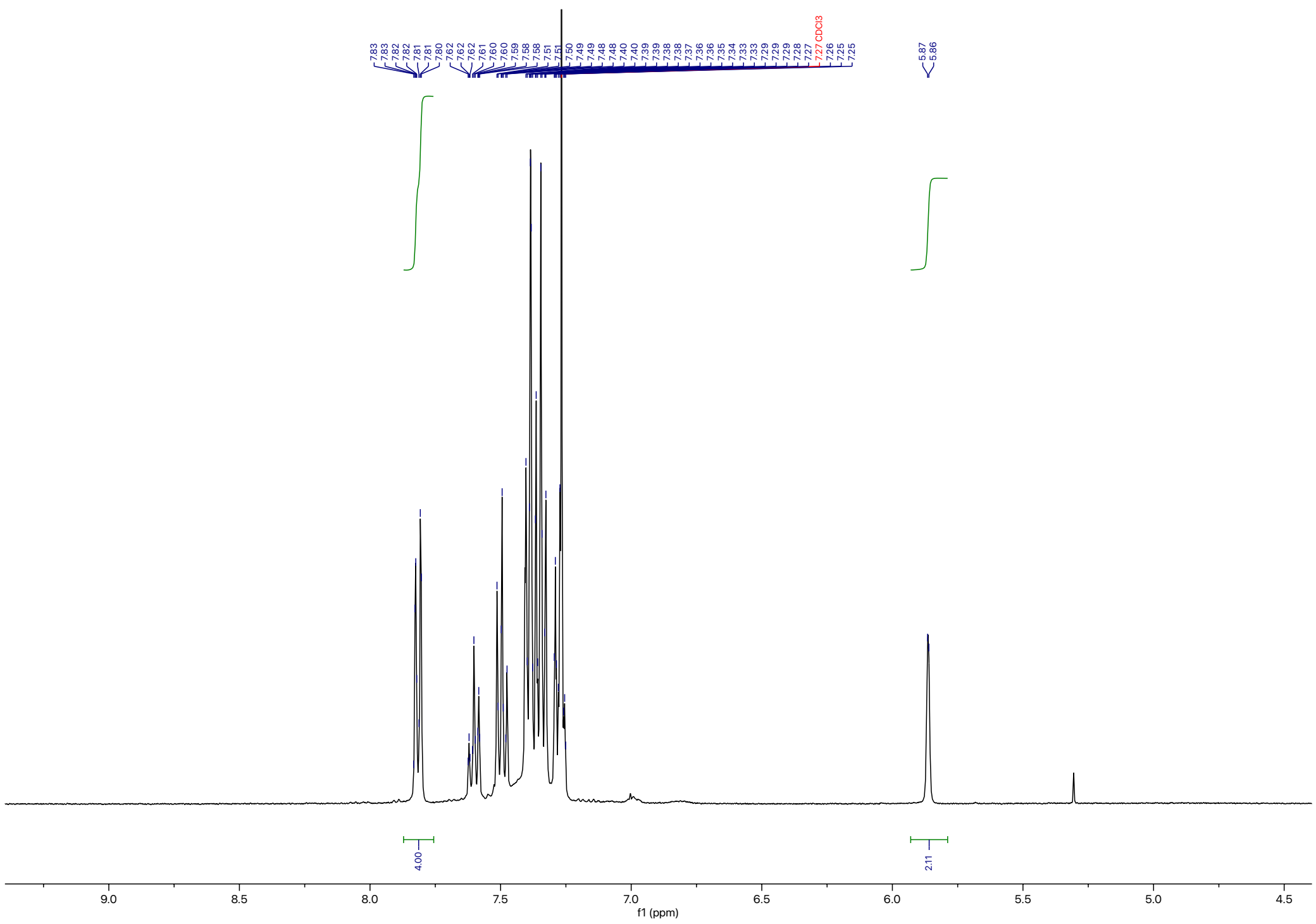

Figure S96. ¹H NMR spectra of [Pt(Xantphos)Cl_2] (**8**) with 25 mg of diphenylmethanol, 250 mg of Oxone® and 150 mg of Na_3PO_4 (400.1 MHz, $CDCl_3$, 298K).

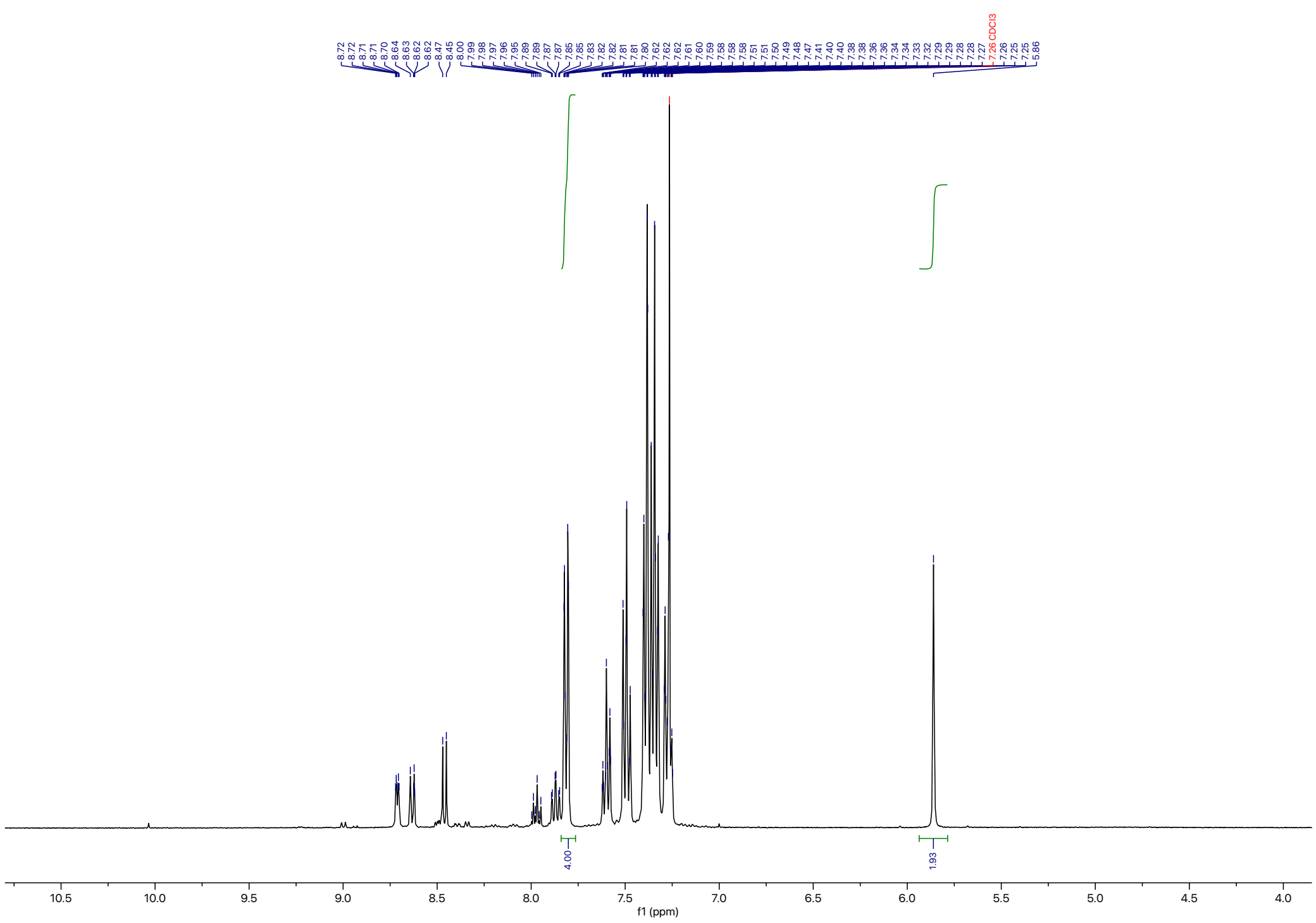

Figure S97. ¹H NMR spectra of $PtCl_2$ (3.7 mg) + terpy (3.3 mg) (**4**) with 25 mg of diphenylmethanol, 250 mg of Oxone® and 150 mg of Na_3PO_4 (400.1 MHz, $CDCl_3$, 298K).

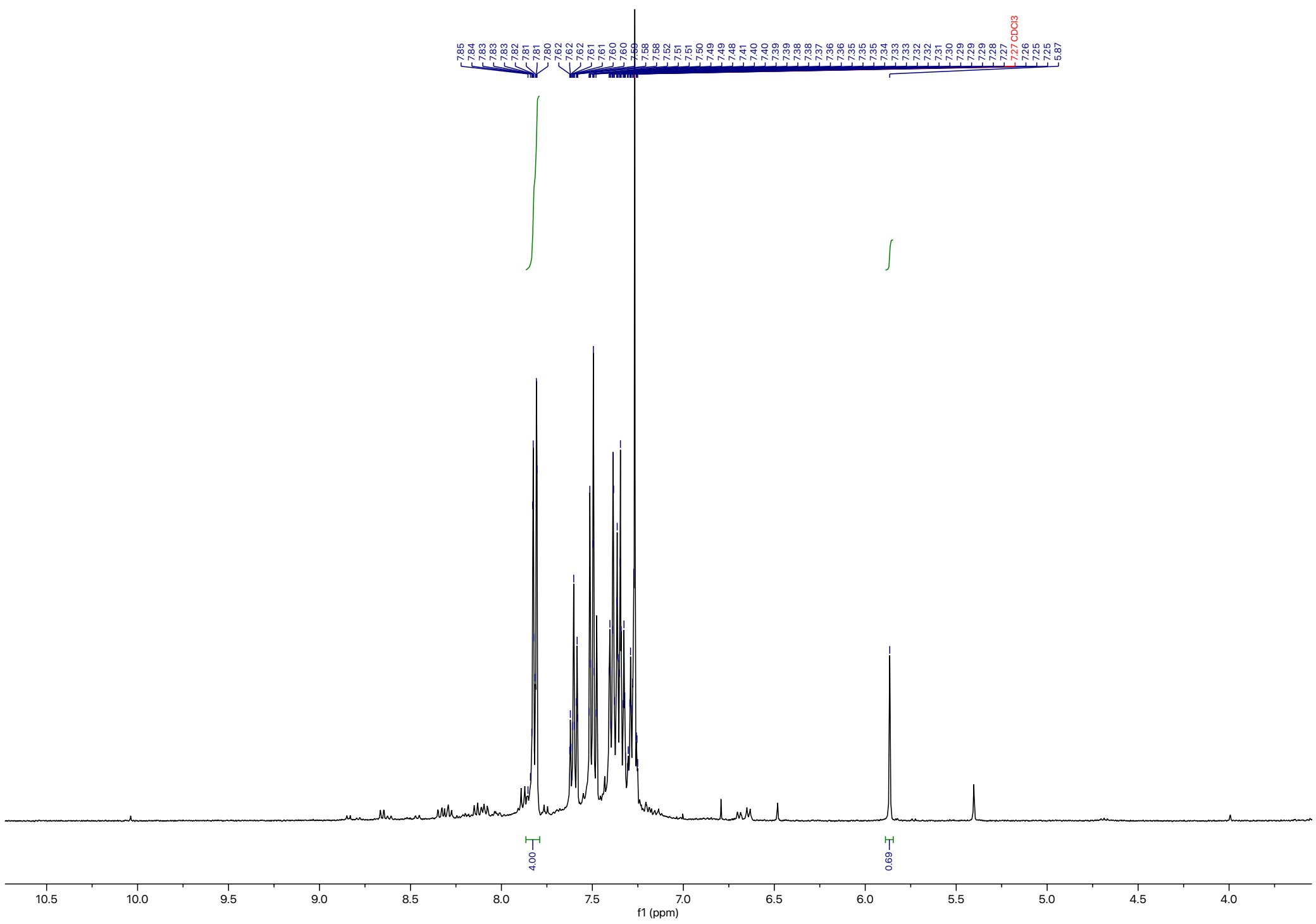

Figure S98. ^{1}H NMR spectra of $PtCl_2$ (3.7 mg) + BIAN (7.0 mg) (**5**) with 25 mg of diphenylmethanol, 250 mg of Oxone® and 150 mg of Na_3PO_4 (400.1 MHz, $CDCl_3$, 298K).

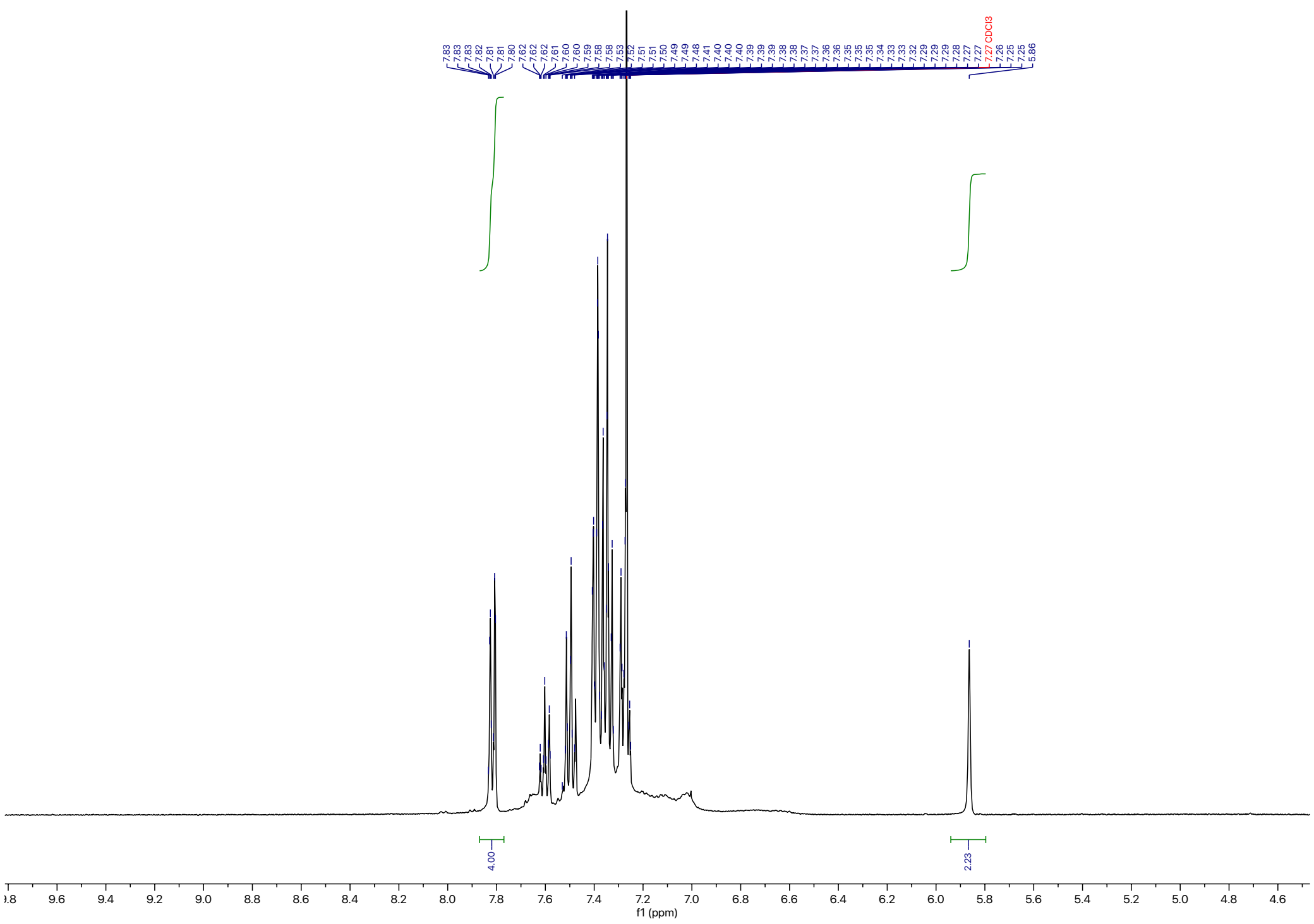

Figure S99. ^{1}H NMR spectra of $PtCl_2$ (3.7 mg) + Xantphos (8.1 mg) (**8**) with 25 mg of diphenylmethanol, 250 mg of Oxone® and 150 mg of Na_3PO_4 (400.1 MHz, $CDCl_3$, 298K).

Part 6. FTIR spectra's

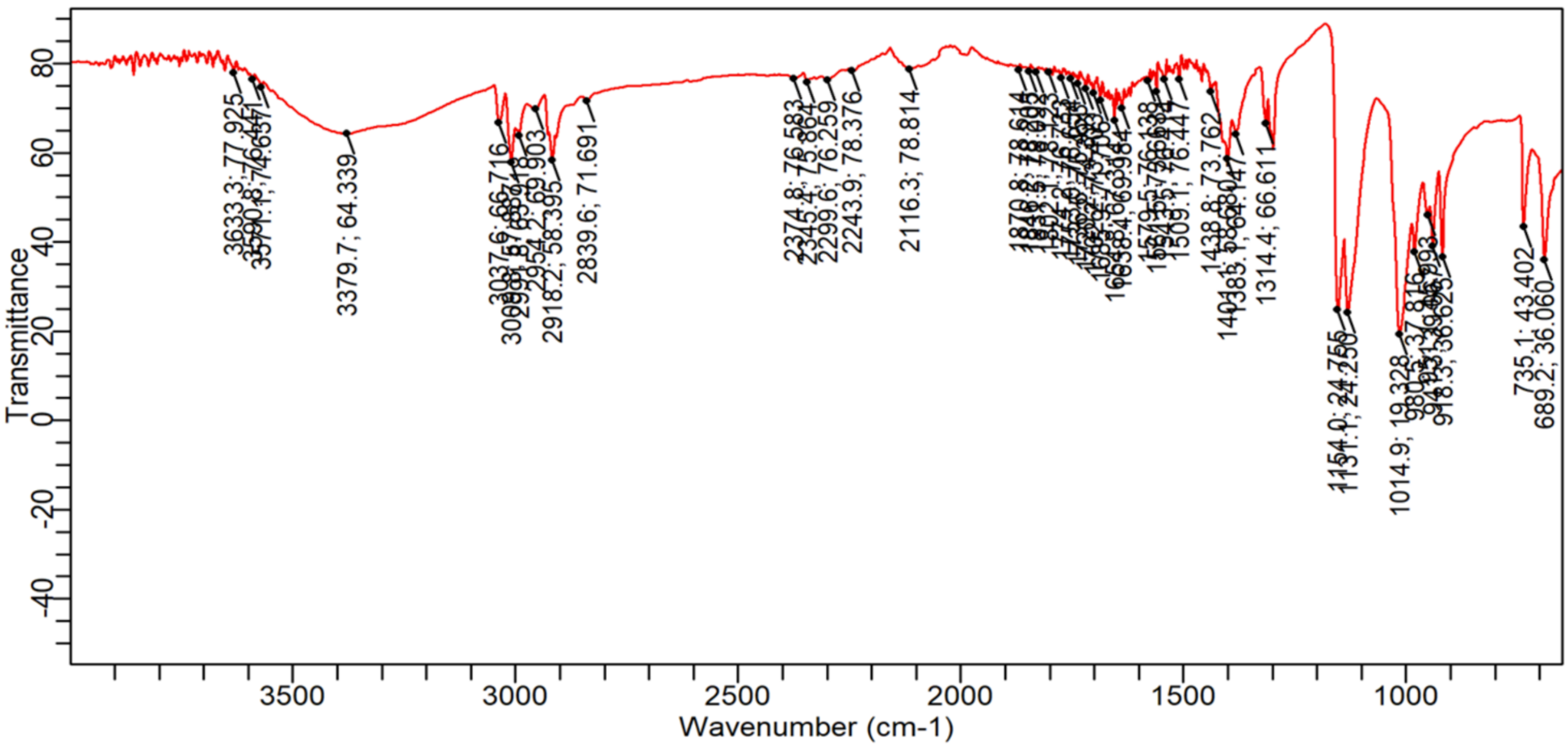

Figure S100. FTIR-ATR of $[Pt(DMSO)_2Cl_2]$ (**1**).

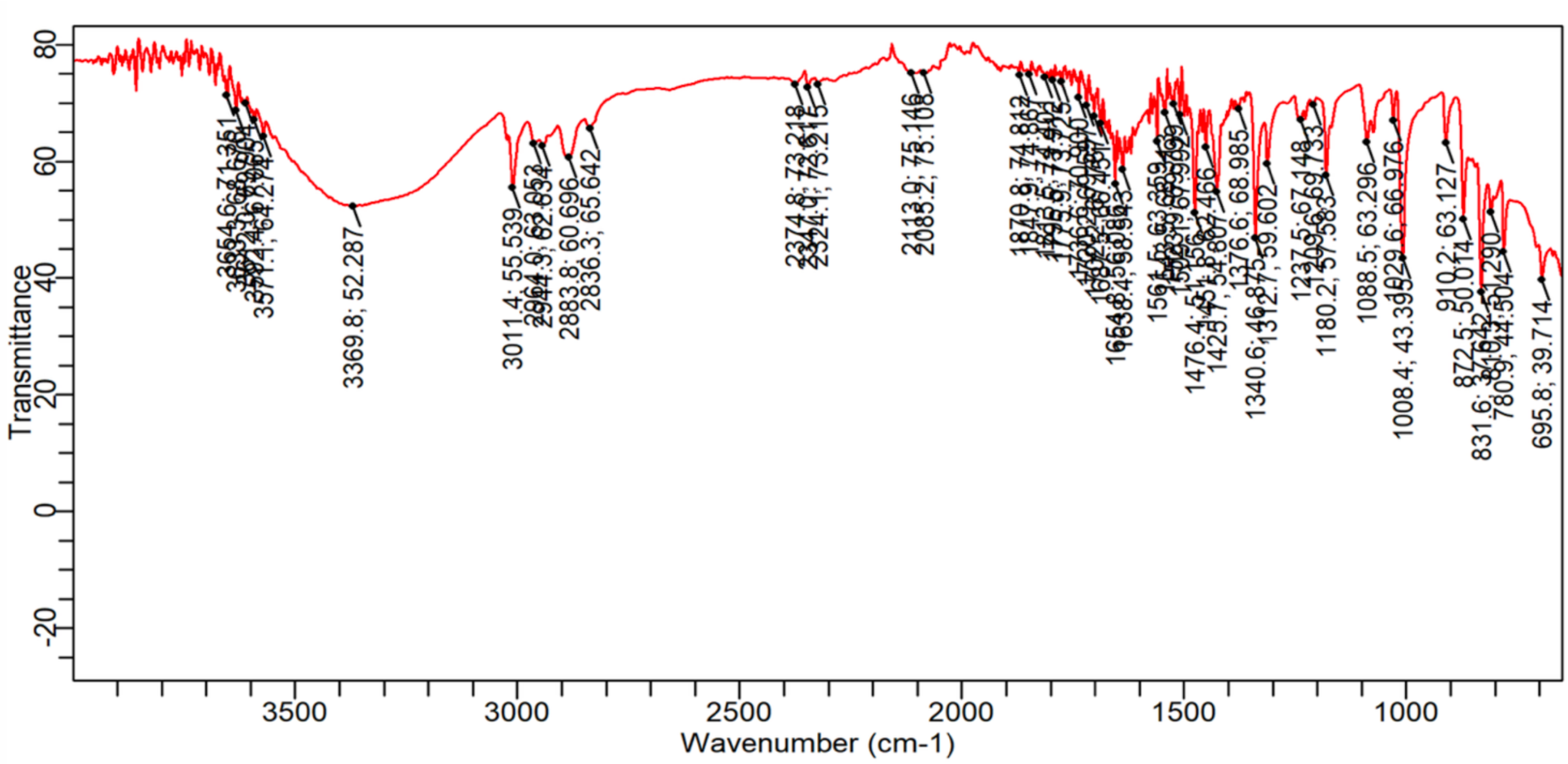

Figure S101. FTIR-ATR of $[Pt(COD)Cl_2]$ (**2**).

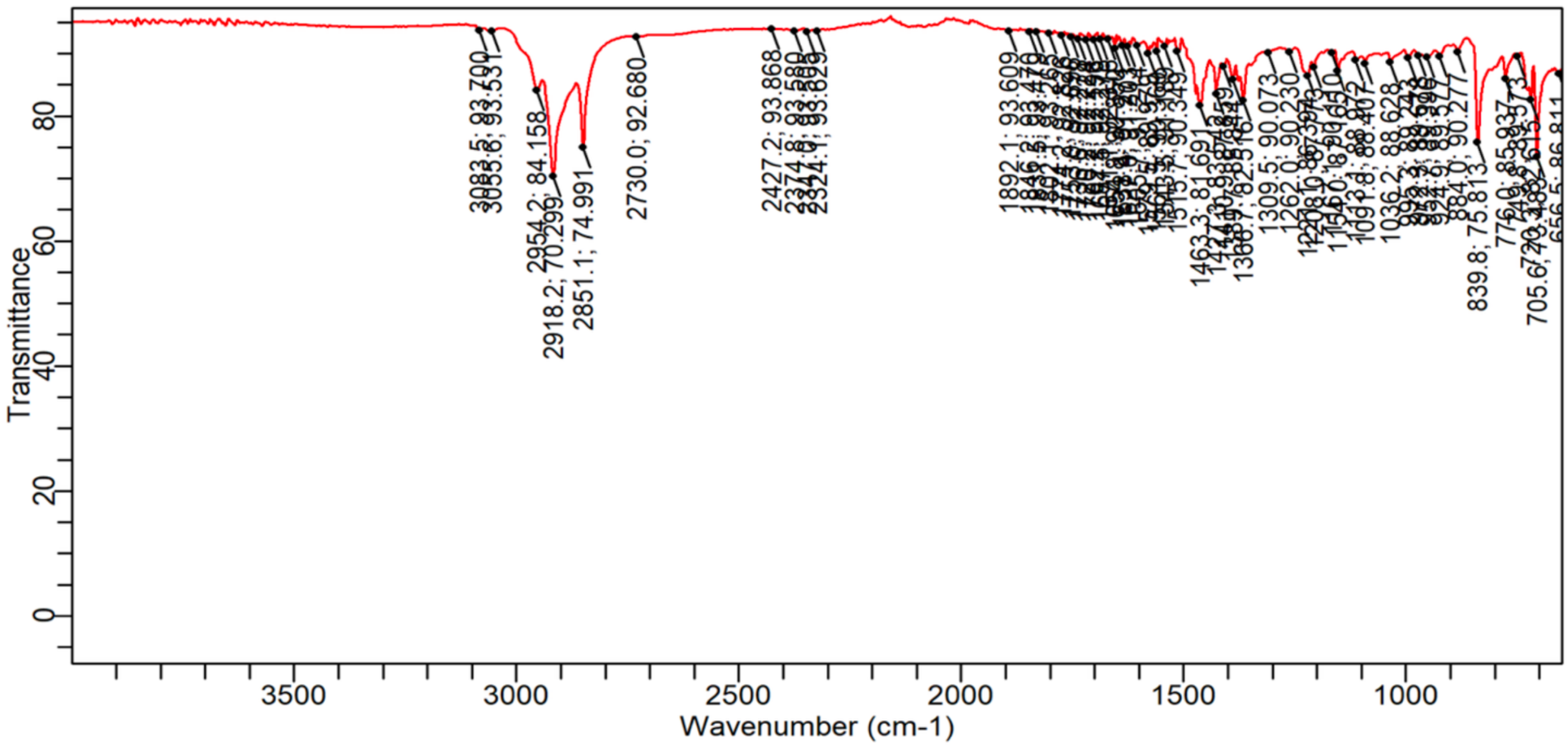

Figure S102. FTIR-ATR of [Pt(phen)Cl_2] (**3**).

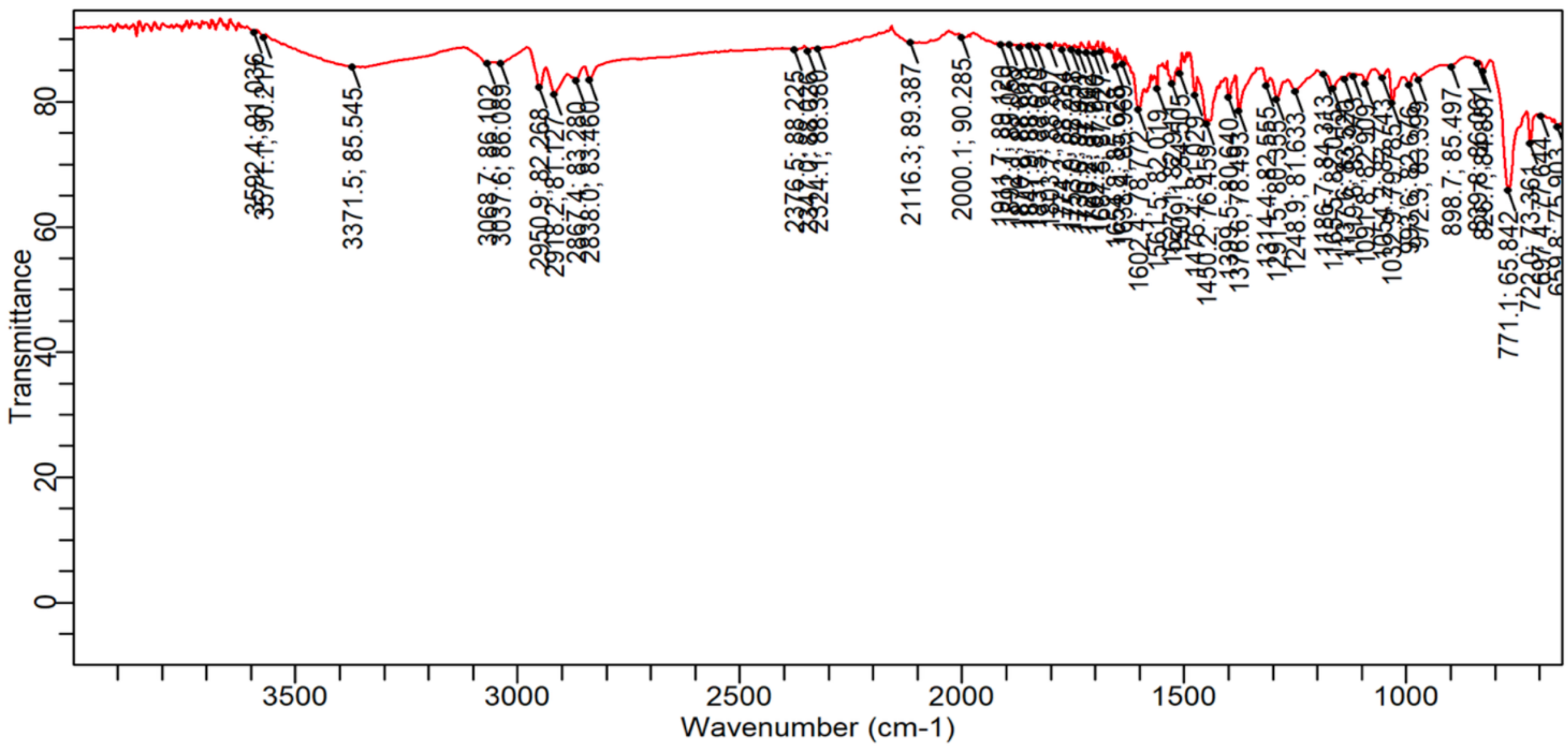

Figure S103. FTIR-ATR of [Pt(κ^2-terpy)Cl_2] (**4**).

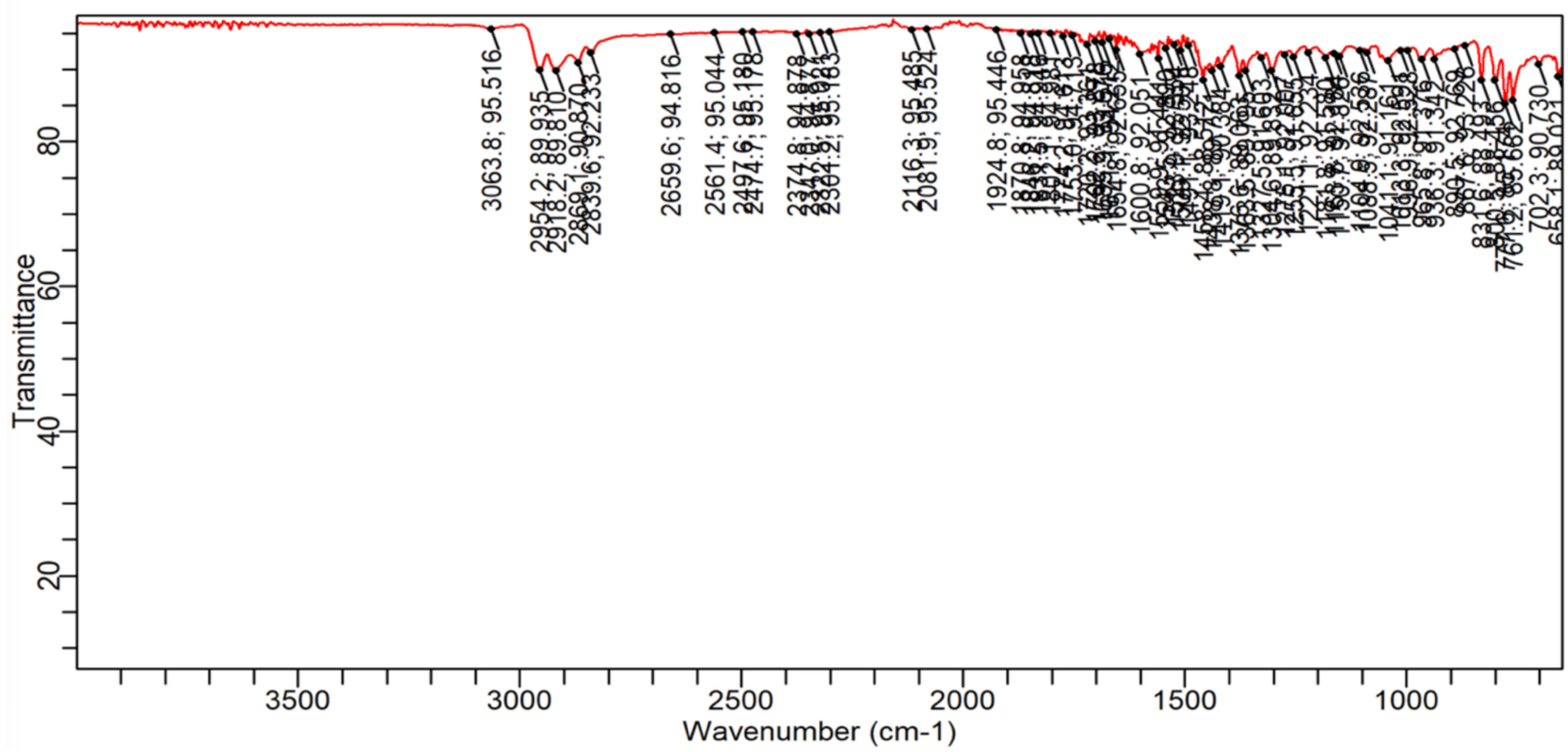

Figure S104. FTIR-ATR of [Pt(BIAN)Cl_2] (**5**).

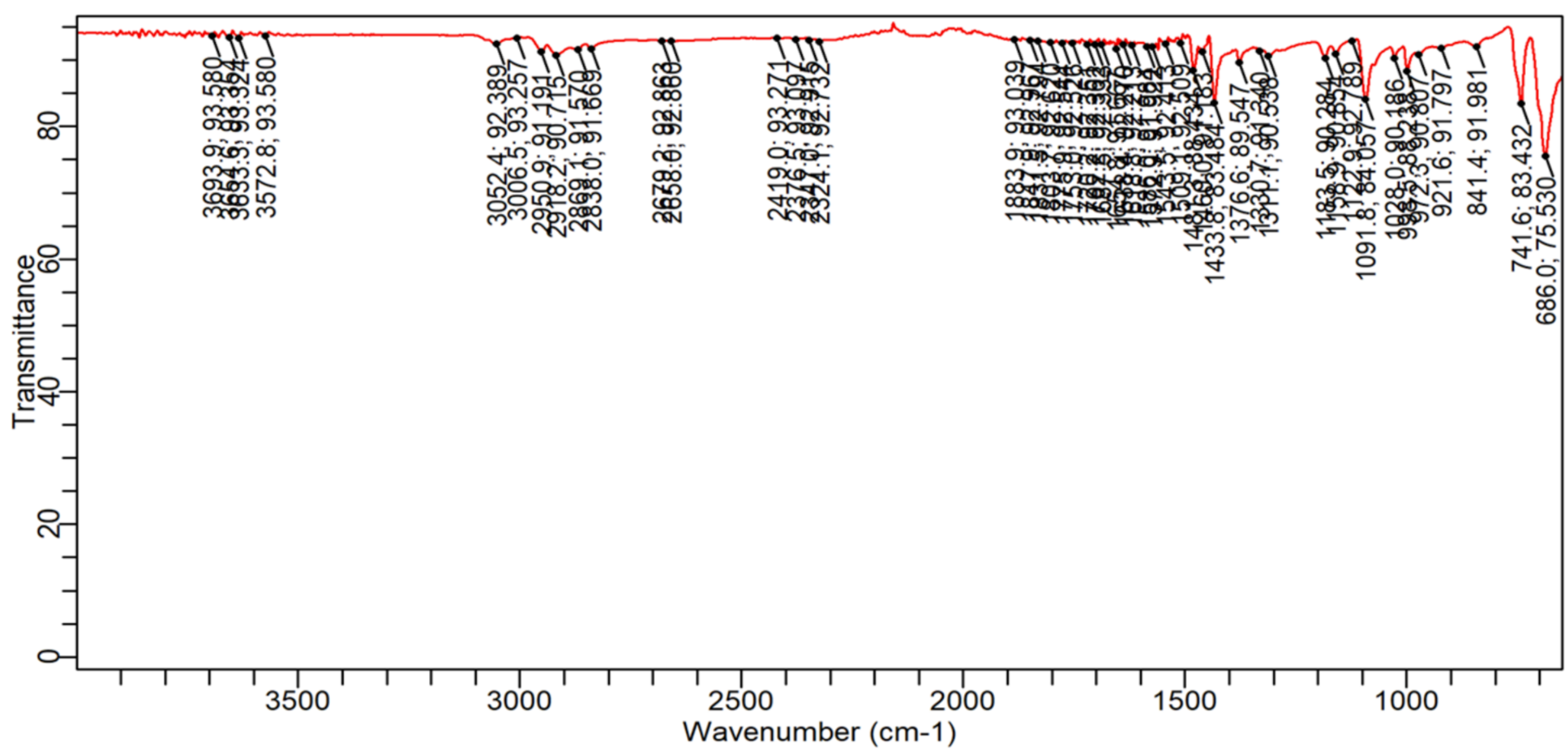

Figure S105. FTIR-ATR of [Pt(PPh_3)$_2Cl_2$] (**6**).

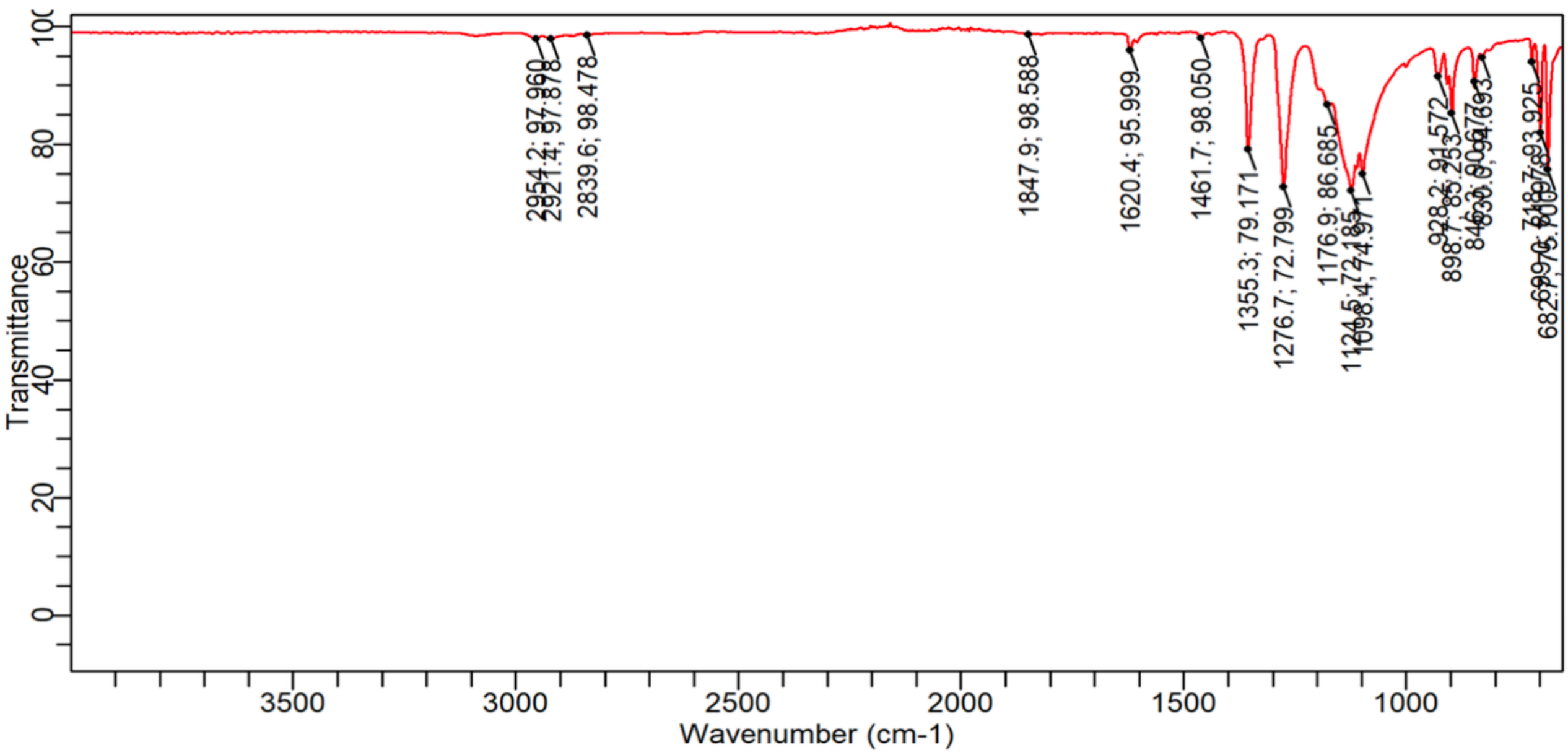

Figure S106. FTIR-ATR of [Pt(P(3,5-$(CF_3)_2$Ph$)_3)_2Cl_2$] (**7**).

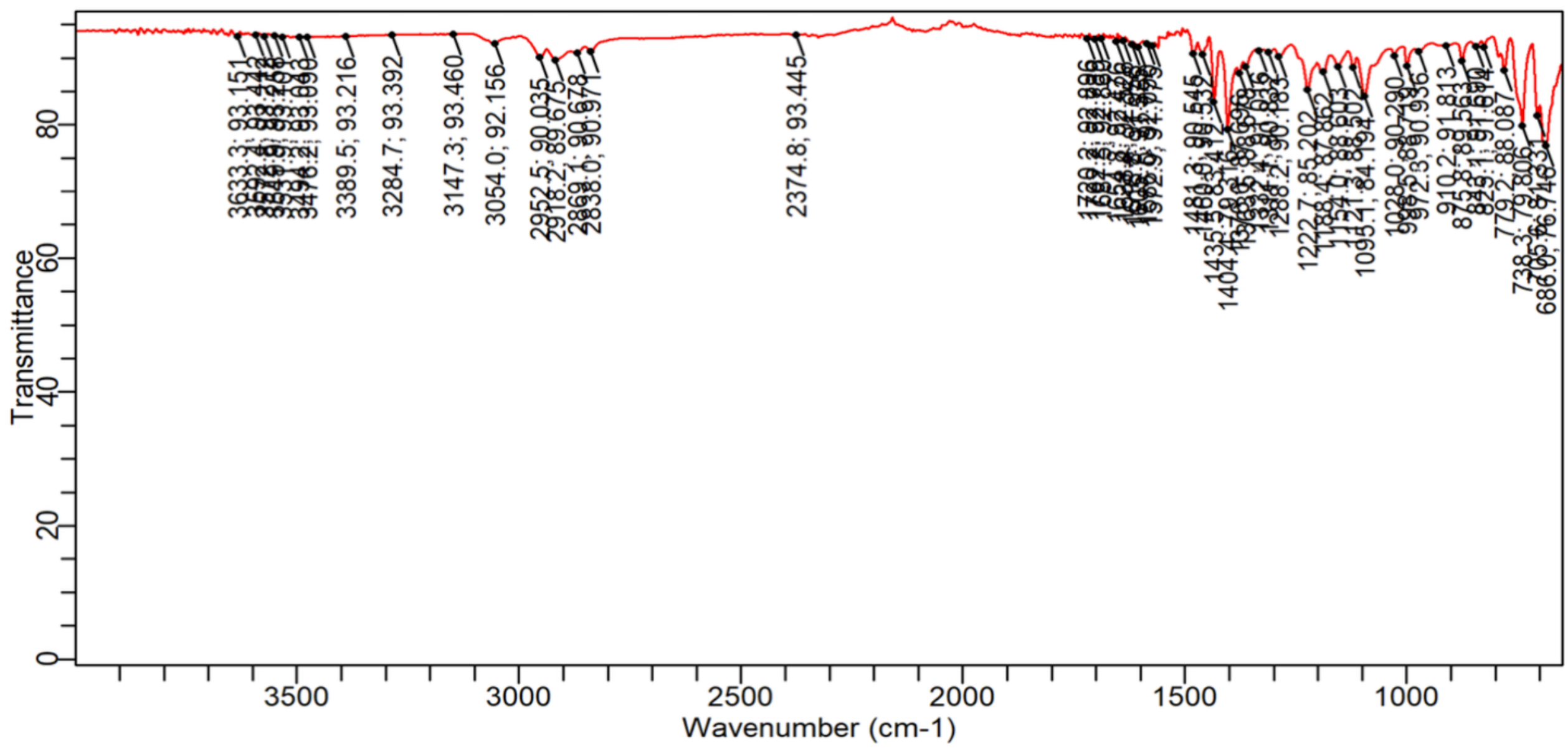

Figure S107. FTIR-ATR of [Pt(Xantphos)Cl_2] (**8**).

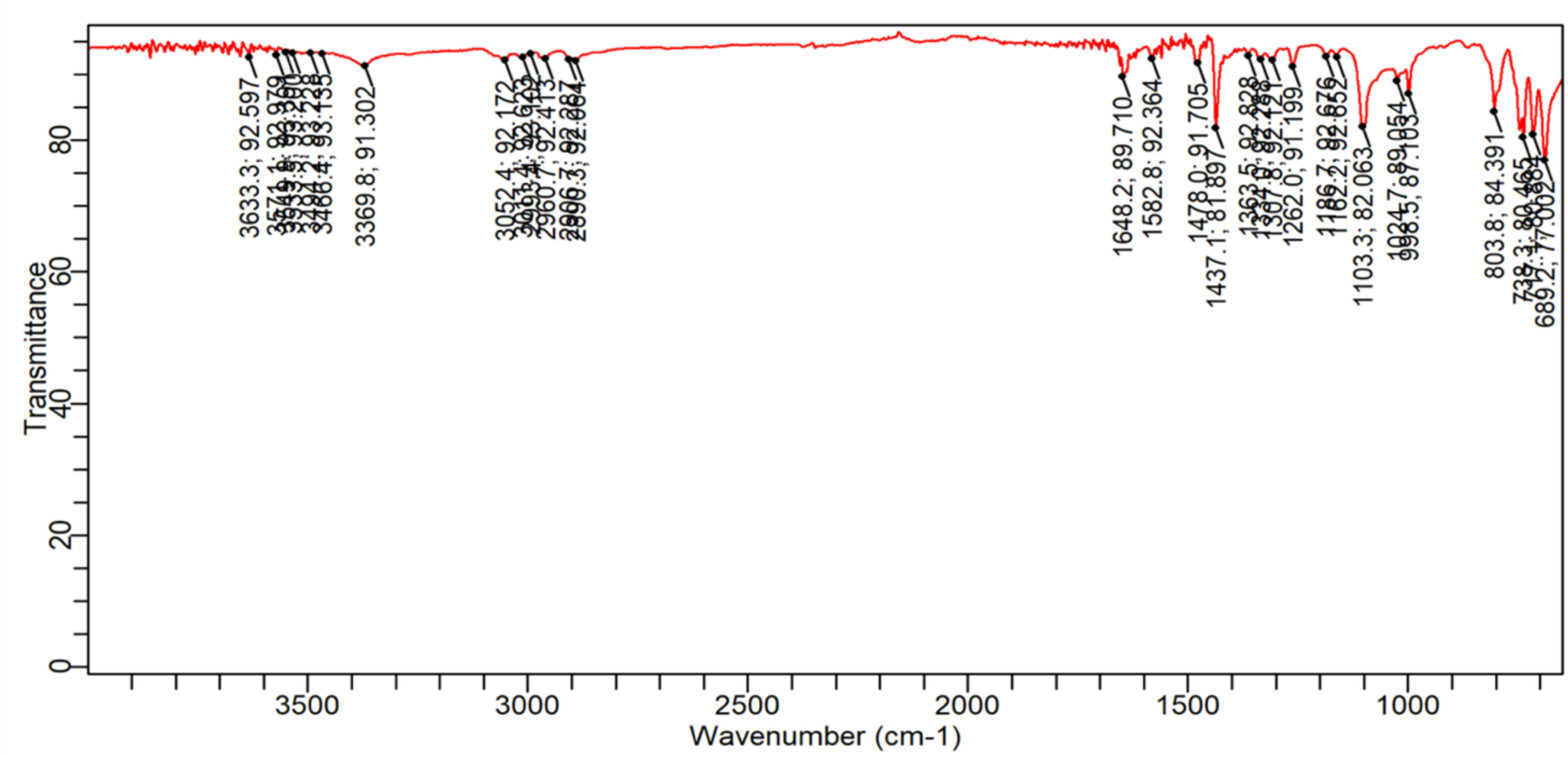

Figure S108. FTIR-ATR of [Pt(dppm)Cl_2] (**9**).

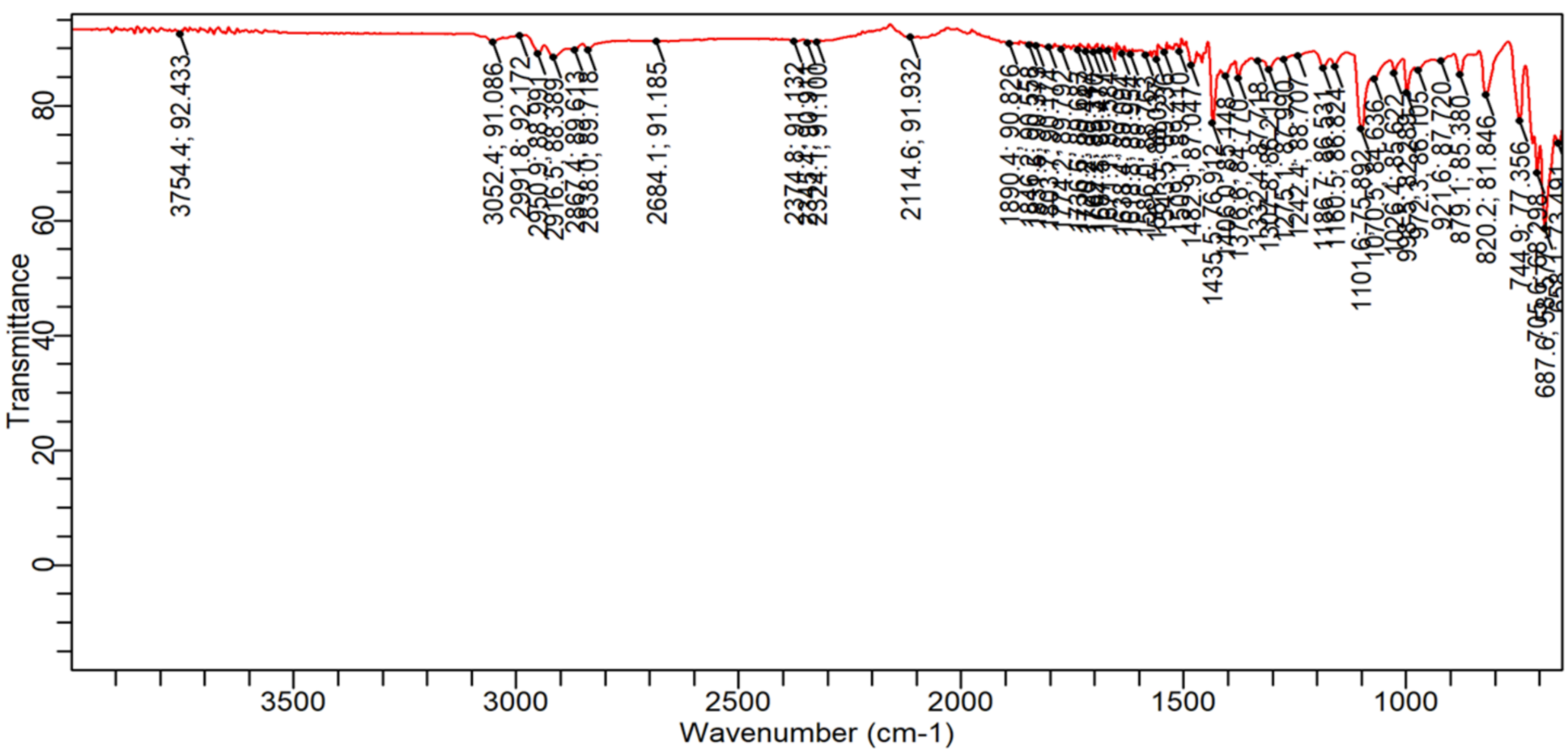

Figure S109. FTIR-ATR of [Pt(dppe)Cl_2] (**10**).

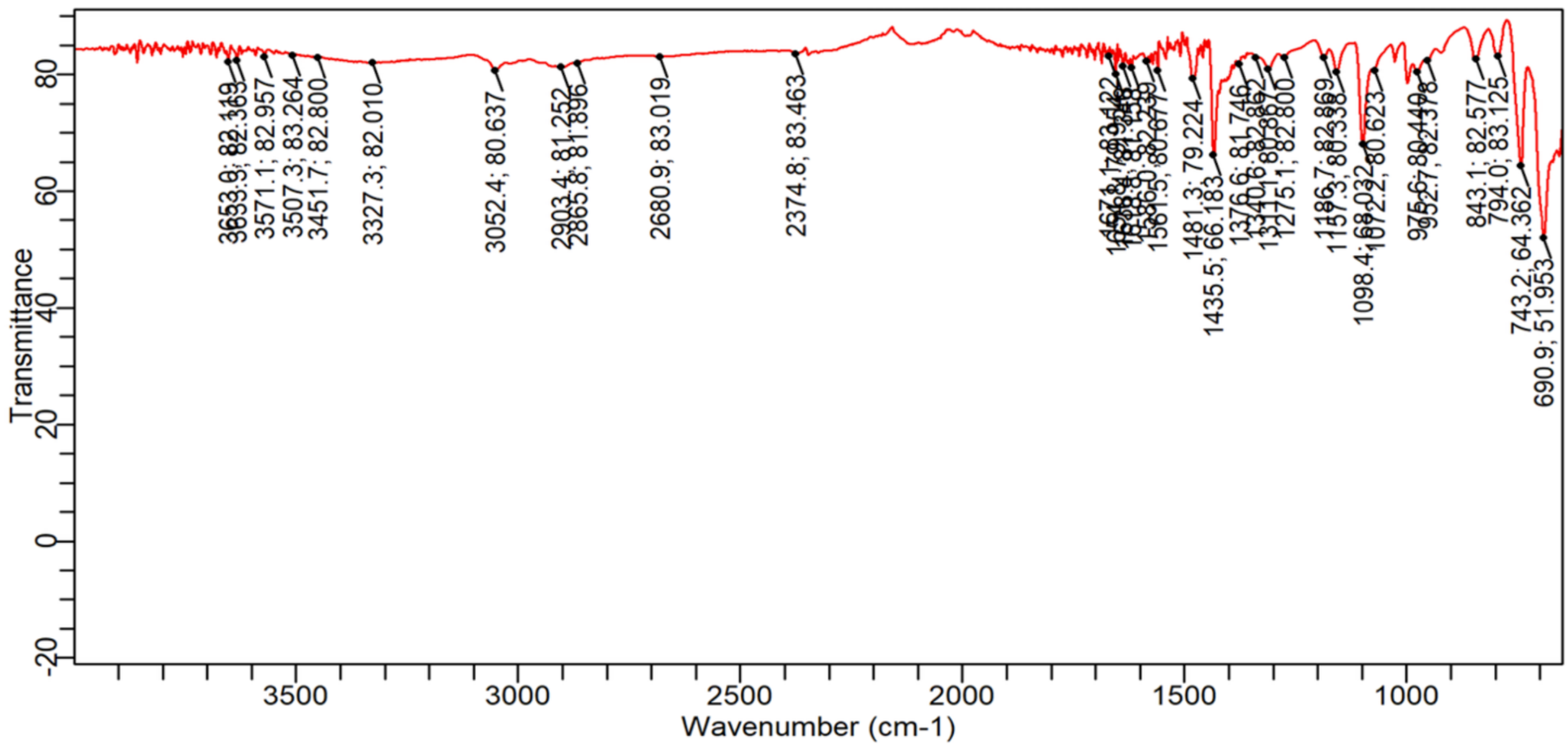

Figure S110. FTIR-ATR of [Pt(dppp)Cl_2] (**11**).

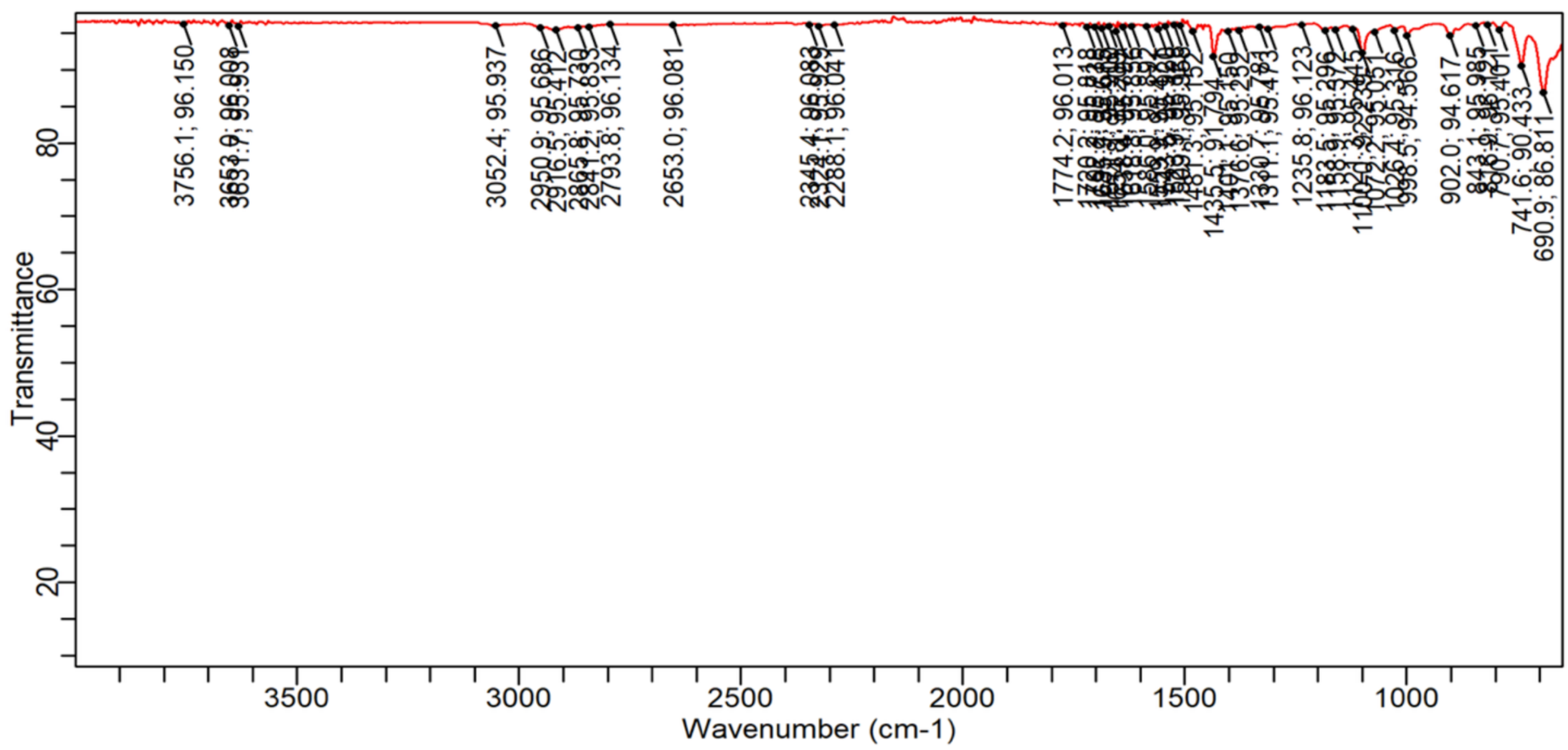

Figure S111. FTIR-ATR of [Pt(dppb)Cl_2] (**12**).

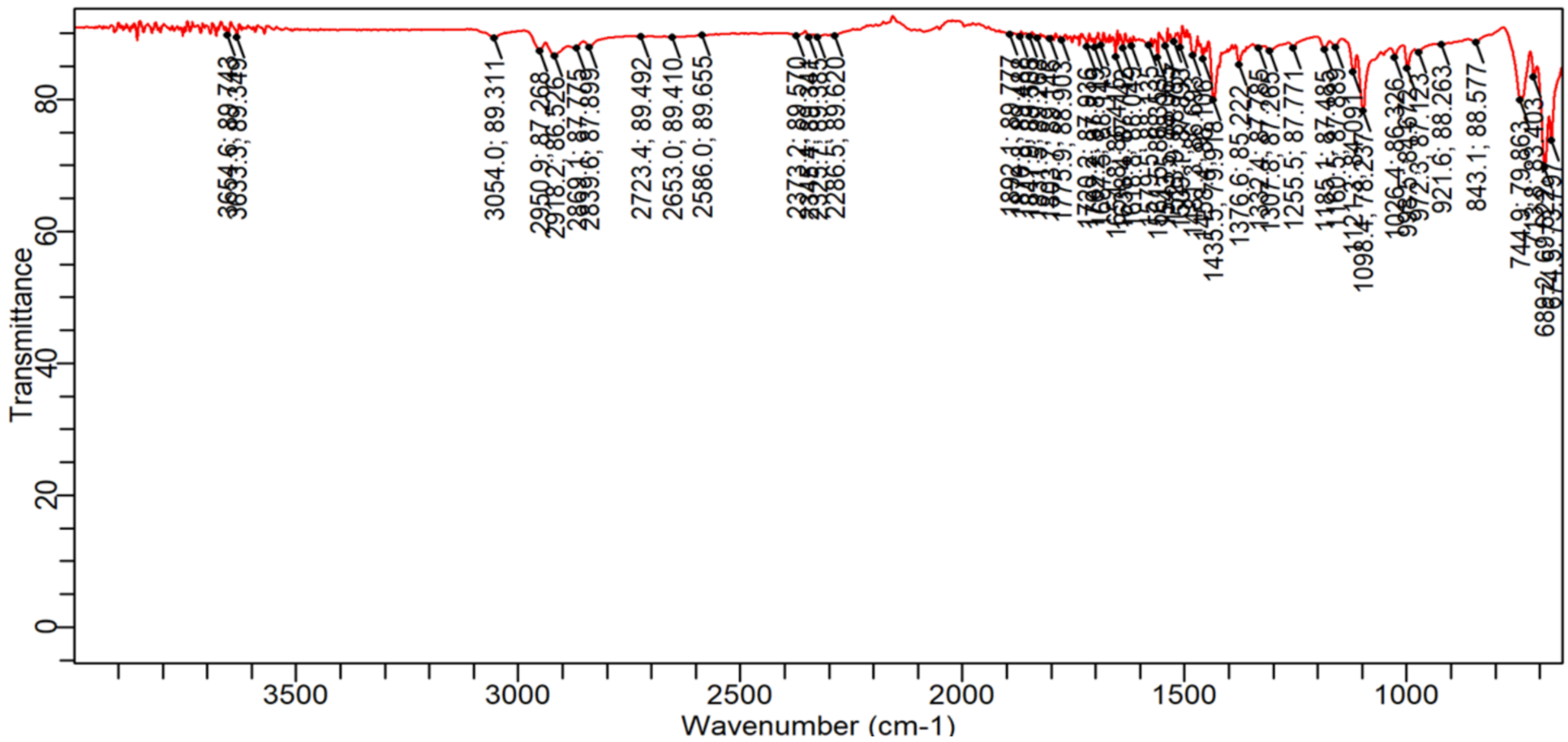

Figure S112. FTIR-ATR of [Pt(dppbz)Cl_2] (**13**).

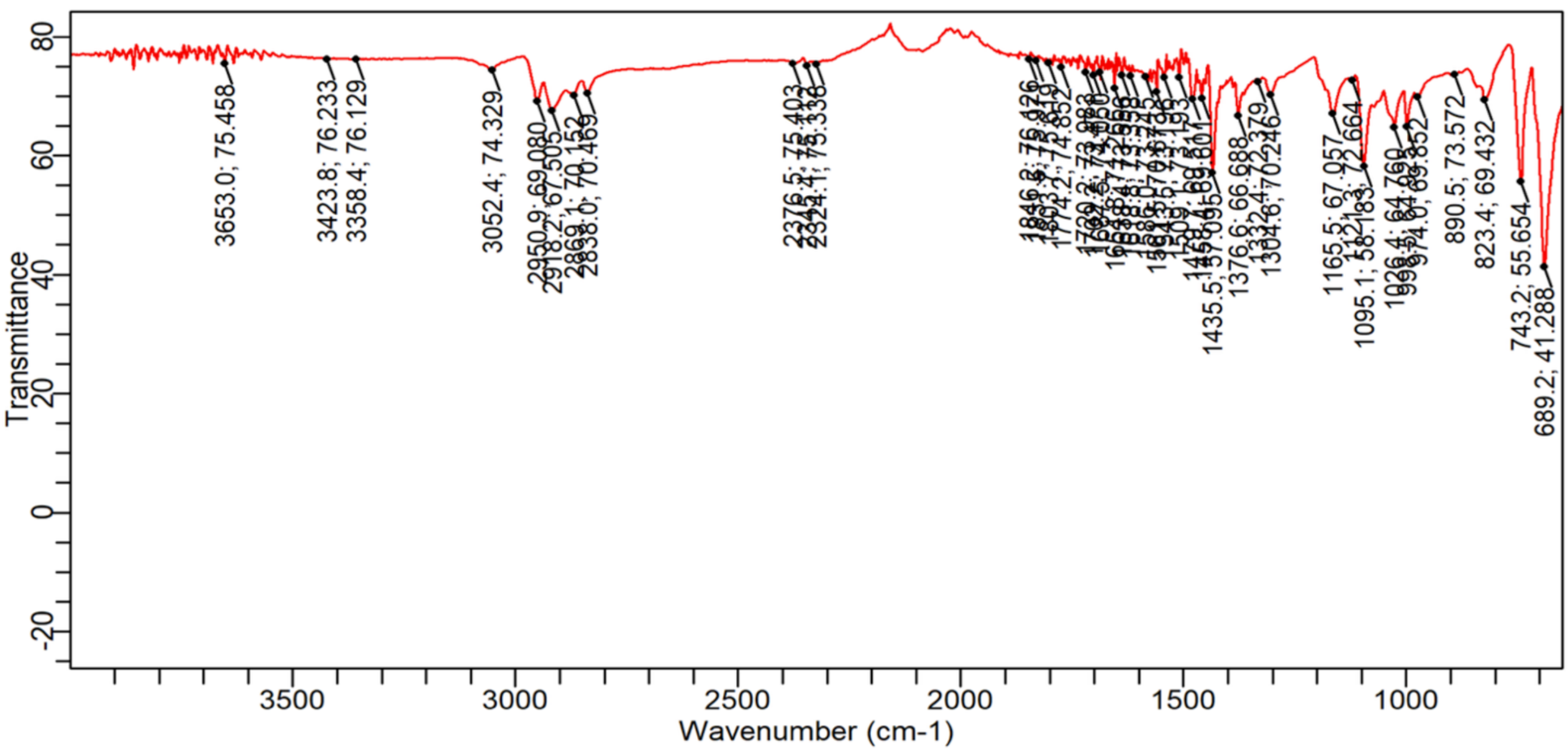

Figure S113. FTIR-ATR of [Pt(dppf)Cl_2] (**14**).

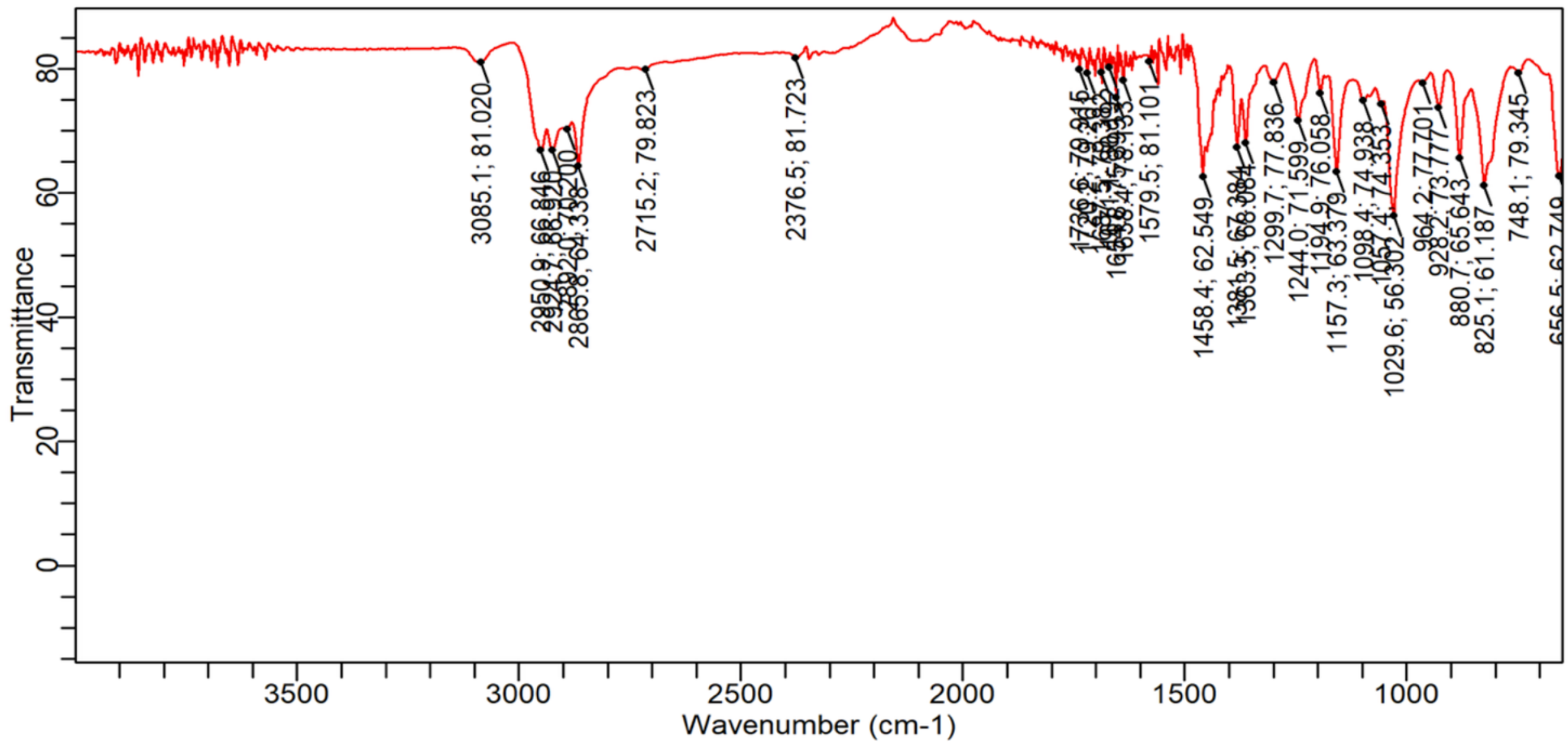

Figure S114. FTIR-ATR of [Pt(dippf)Cl_2] (**15**).

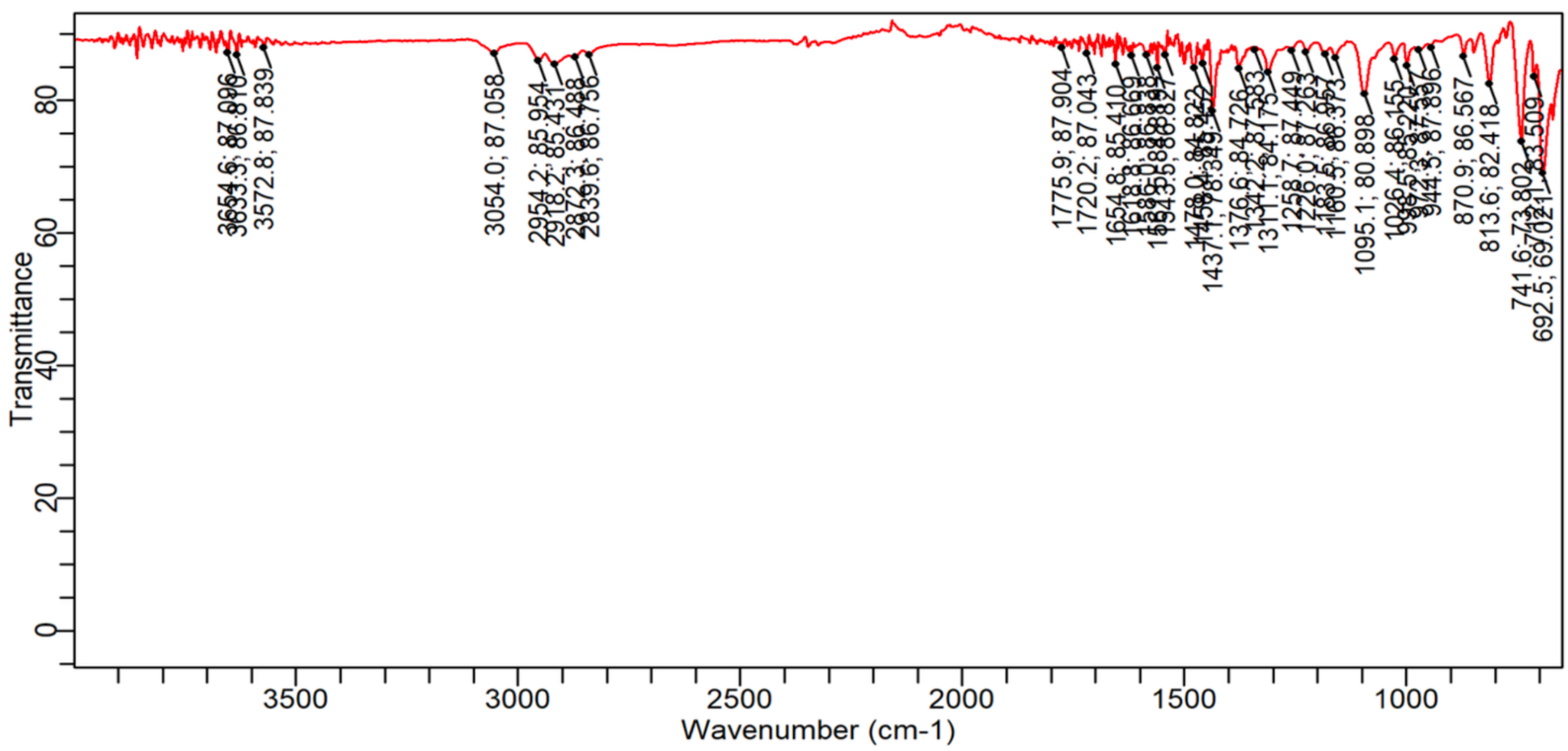

Figure S115. FTIR-ATR of [Pt((R)-BINAP)Cl_2] (**16**).

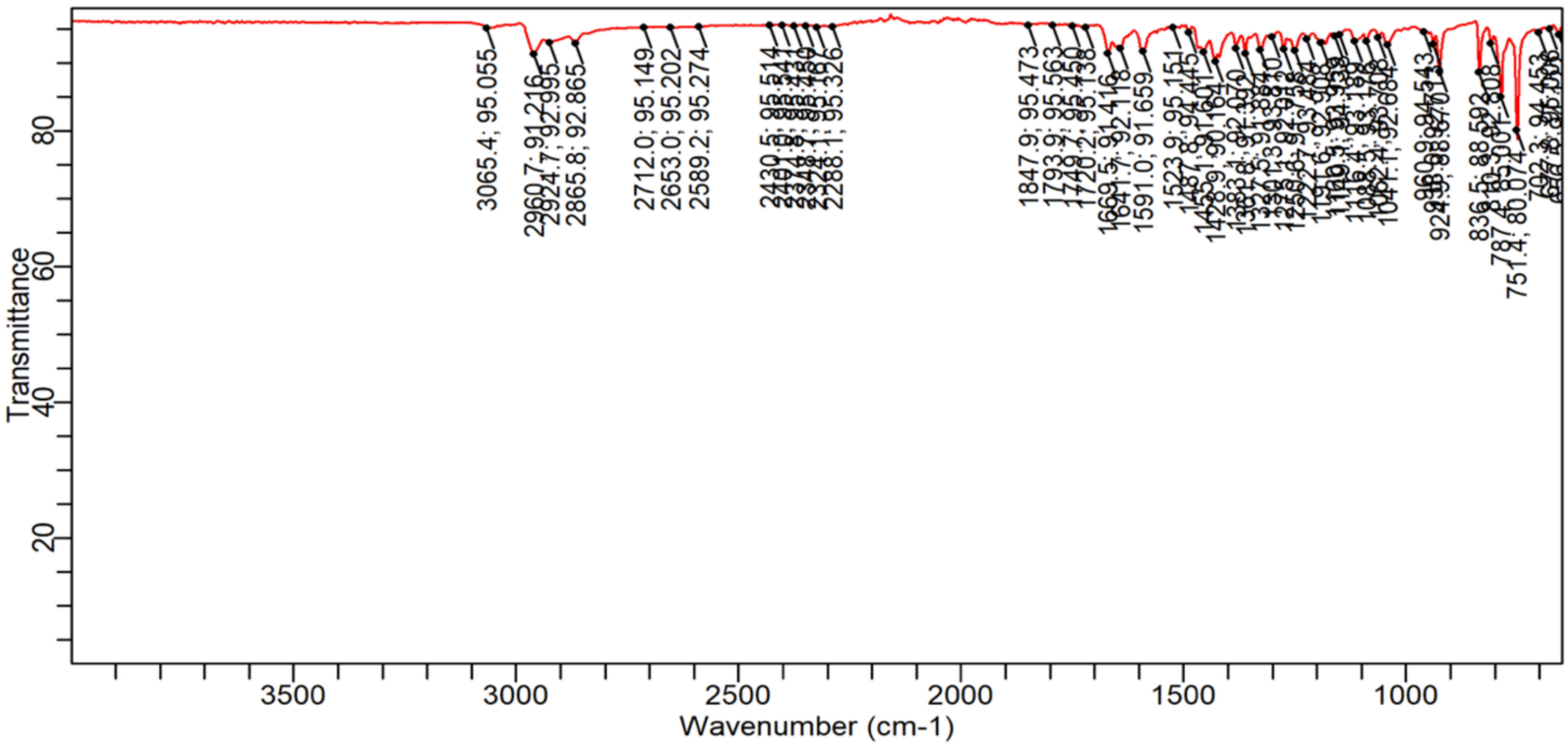

Figure S116. FTIR-ATR of free BIAN.

Part 7. XRPD analysis

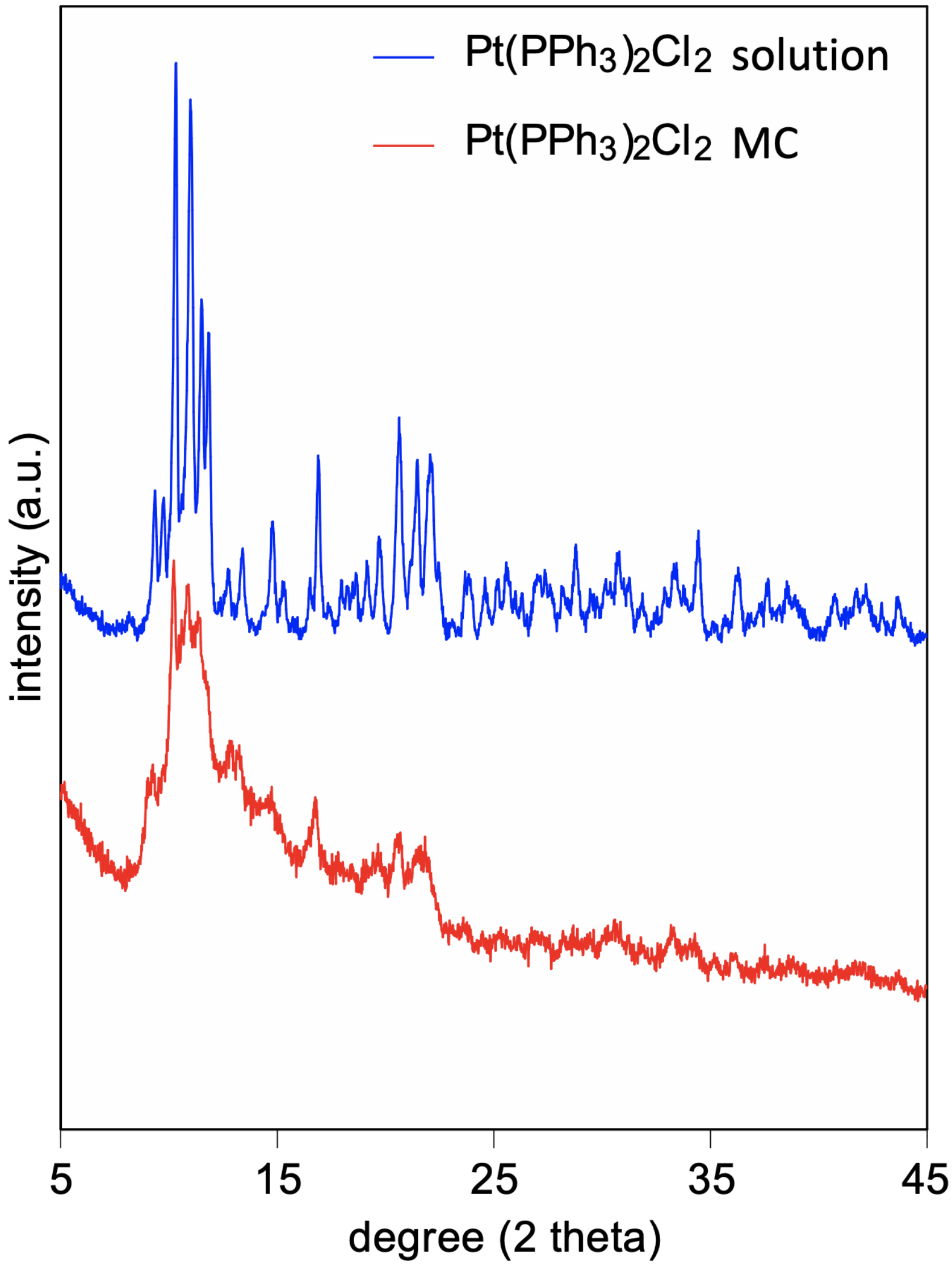

Figure S117. XRPD analysis of [Pt(PPh_3)$_2$$Cl_2$] (**6**) obtain via solution in blue, and via ball mill in red.

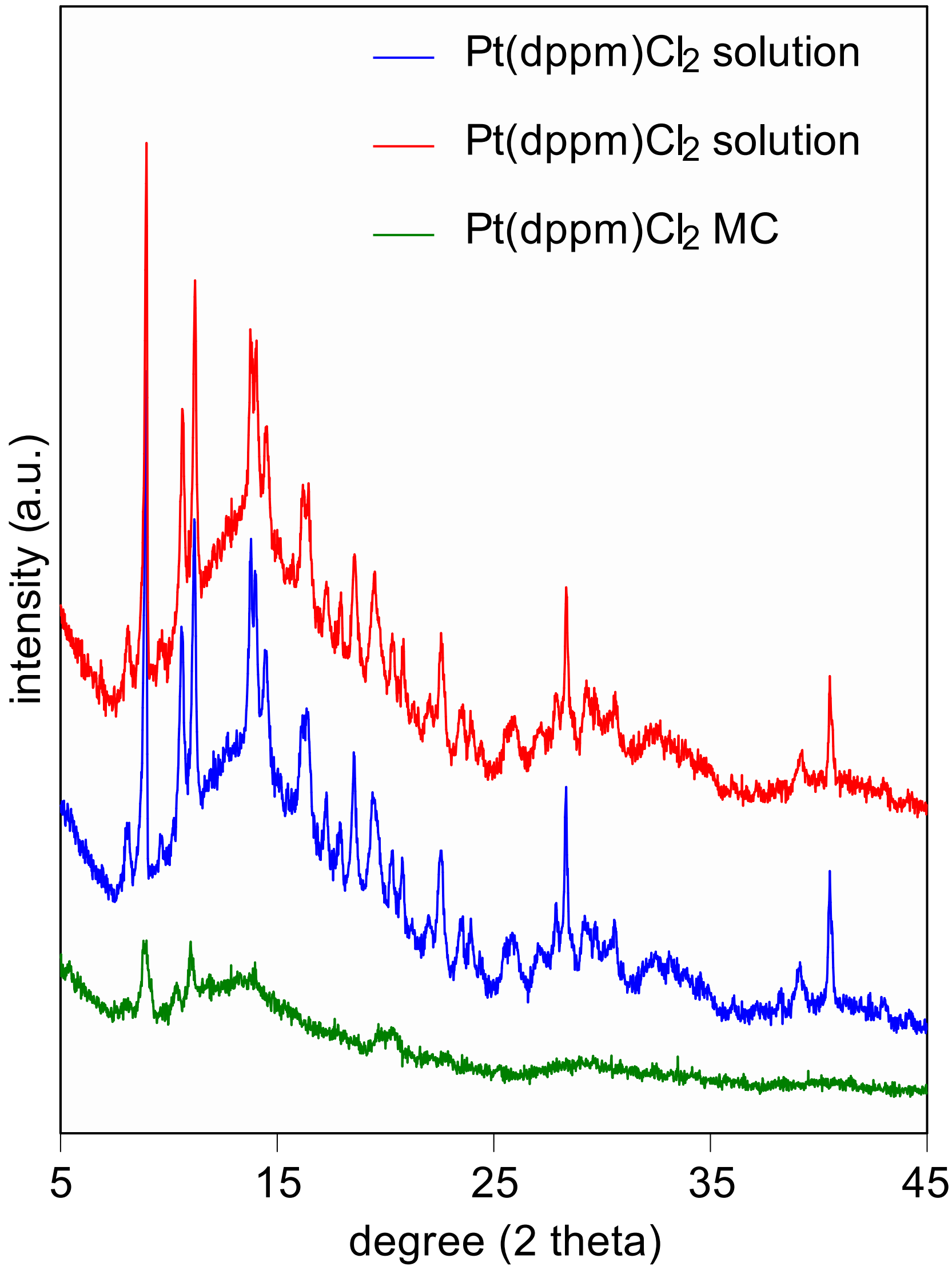

Figure S118. XRPD analysis of [Pt(dppm)Cl_2] (**9**) obtained from solution in red and blue, and obtain via ball-mill in green.

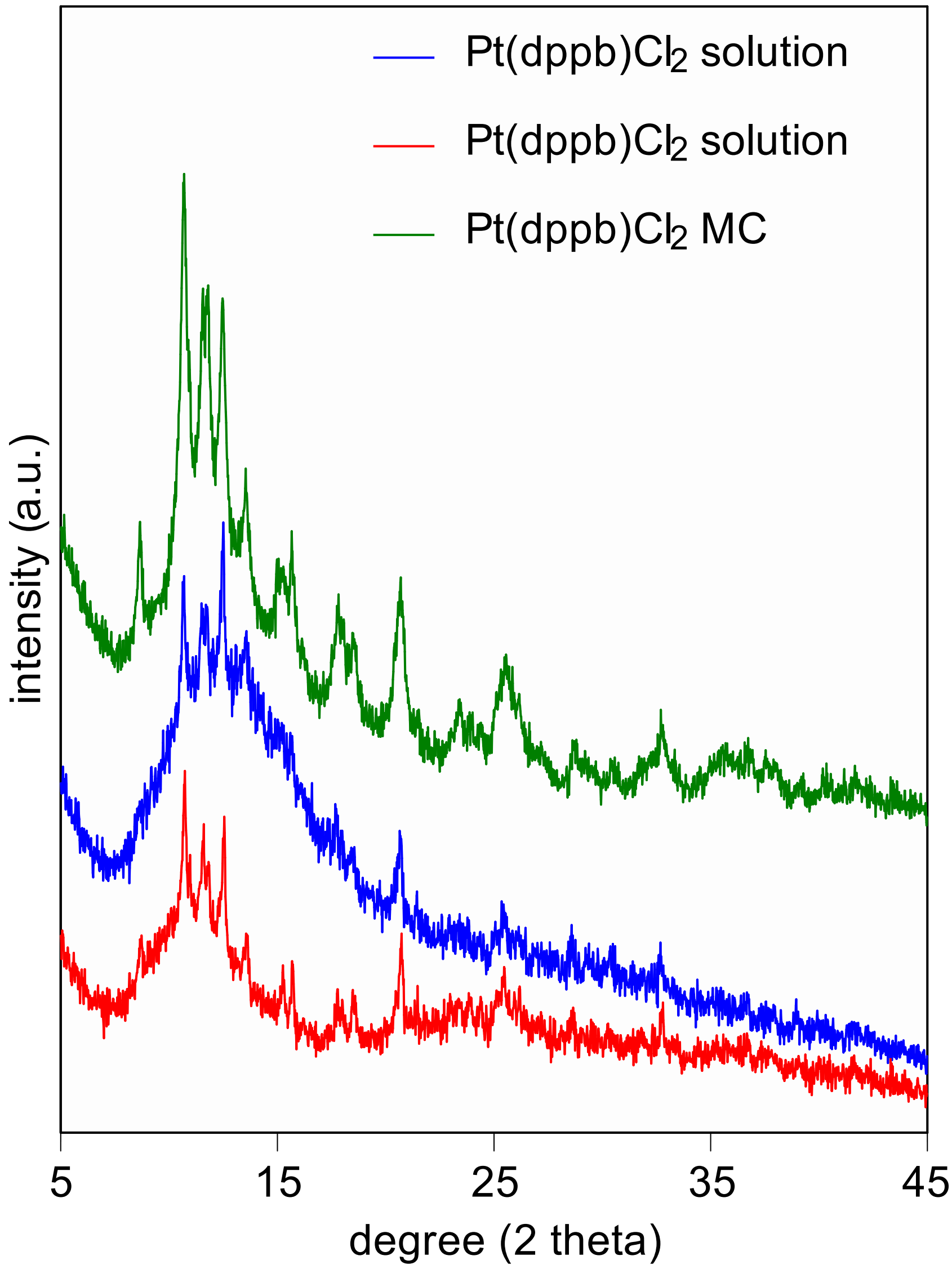

Figure S119. XRPD analysis of [Pt(dppb)Cl_2] (**12**) obtained from solution in red and blue, and obtain via ball-mill in green.

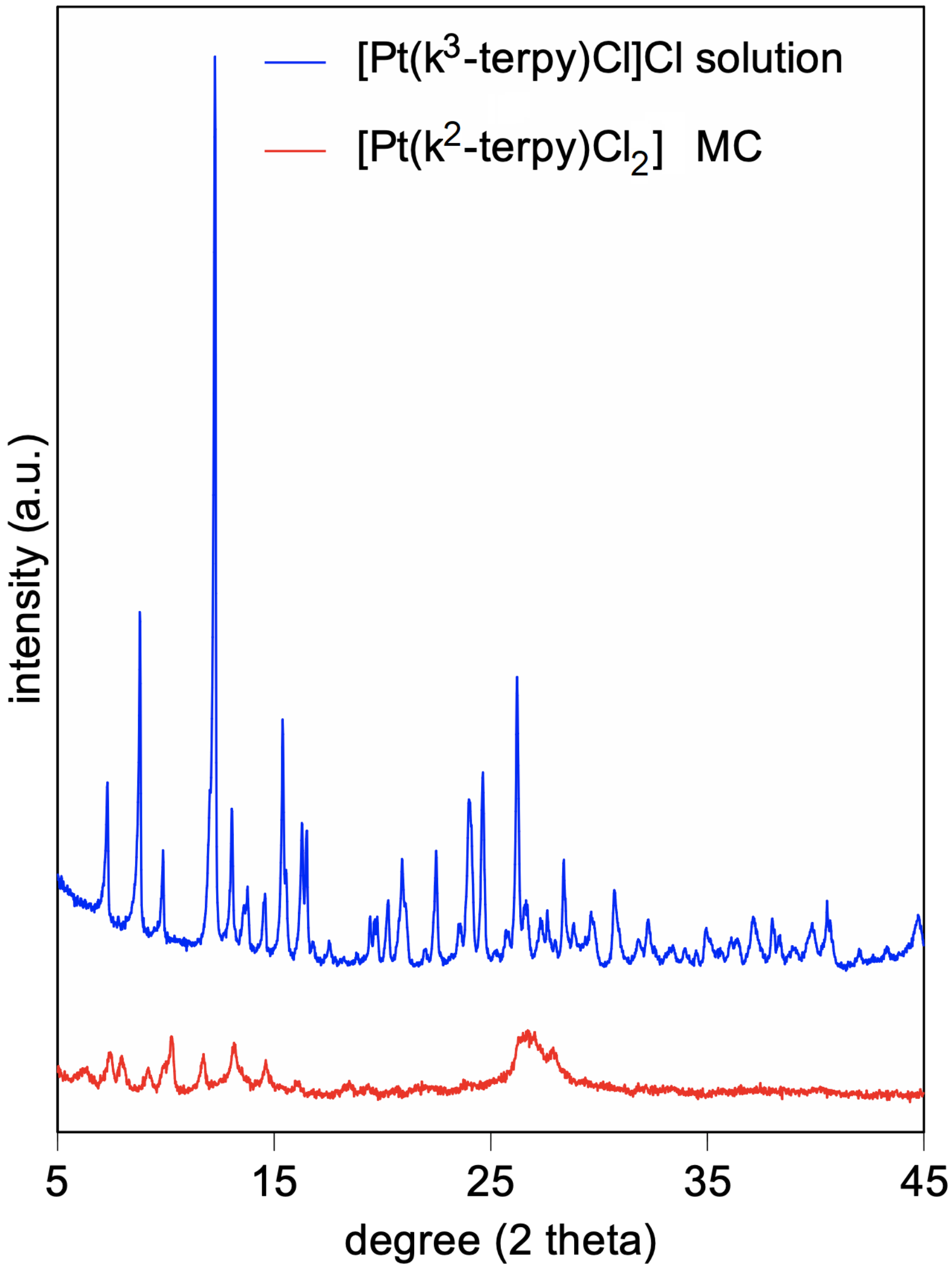

Figure S120. XRPD analysis of [Pt(κ^3-terpy)Cl]Cl obtained from solution in red, and [Pt(κ^2-terpy)Cl_2] (**4**) obtain via ball-mill in blue.

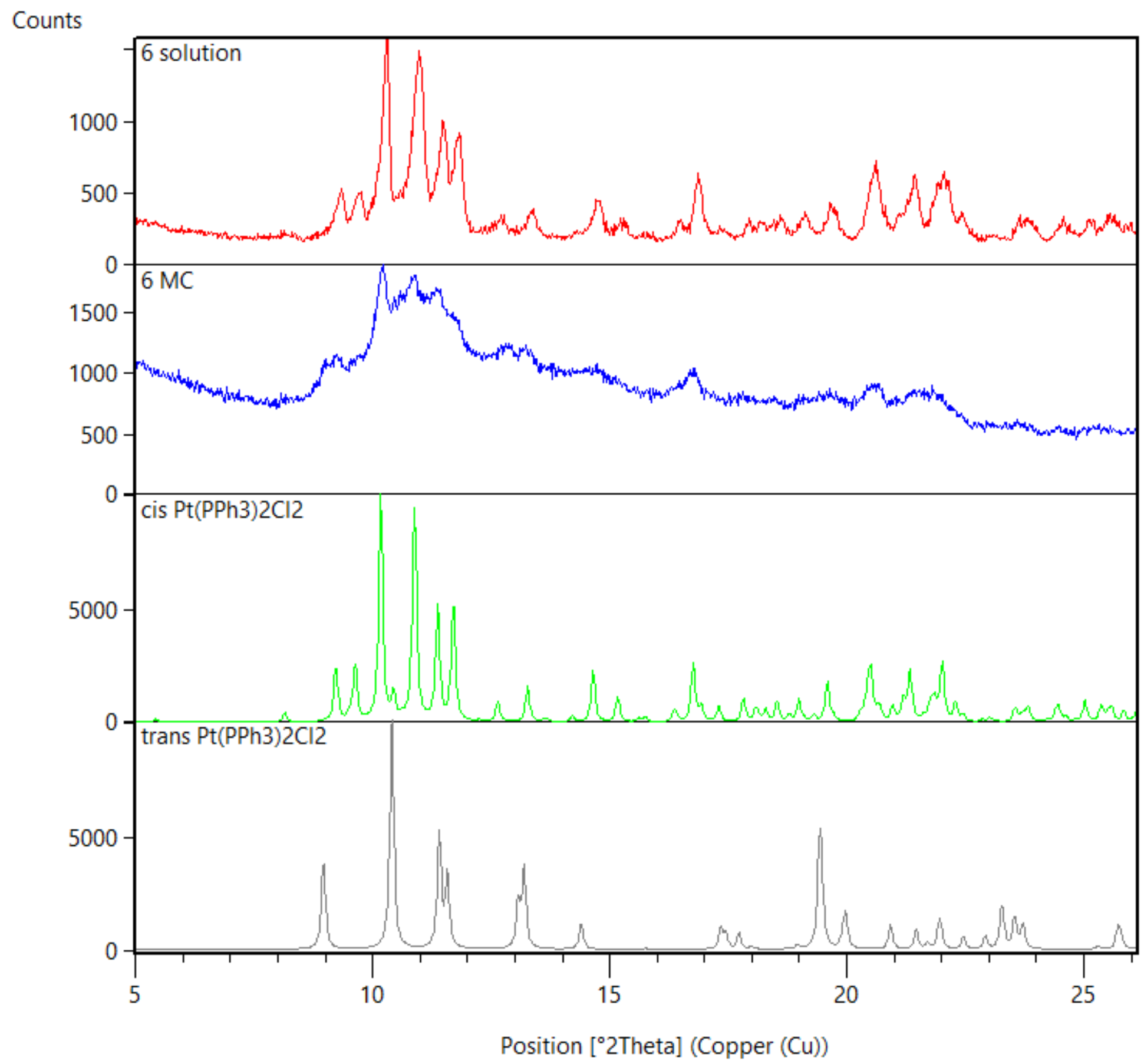

Figure S121. Experimental XRD patterns of [Pt(PPh_3)$_2$$Cl_2$] (**6**) obtained by solution and mechanochemical synthesis, compared with the powder diffraction patterns calculated from the CSD crystal structures of the cis and trans isomers using Mercury (CCDC).

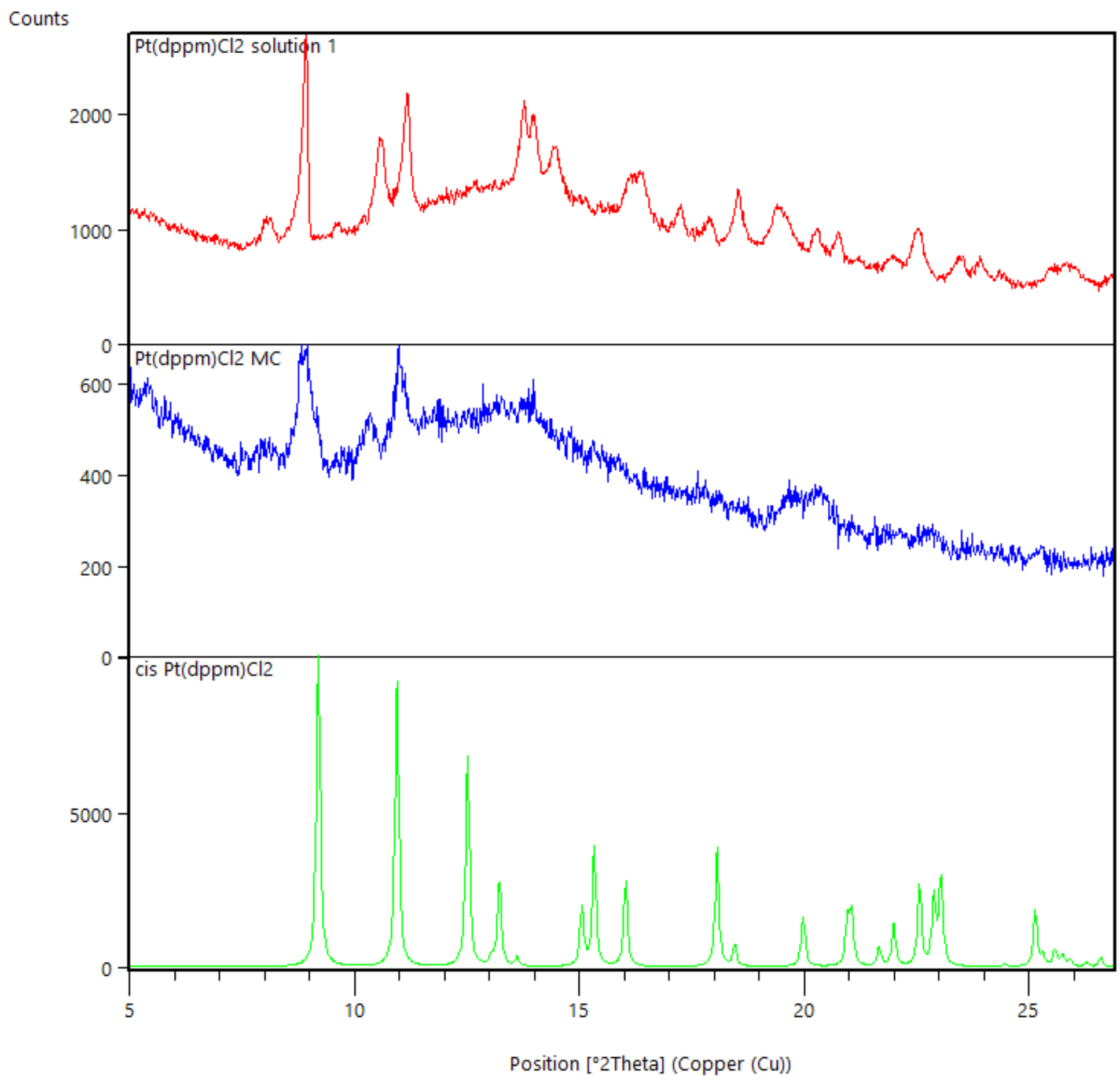

Figure S122. Experimental XRD patterns of [Pt(dppm)Cl_2] (**9**) obtained under different synthetic conditions, compared with the powder diffraction pattern calculated from the CSD crystal structure of *cis*-[Pt(dppm)Cl_2] using Mercury (CCDC).

Part 8. Other pictures

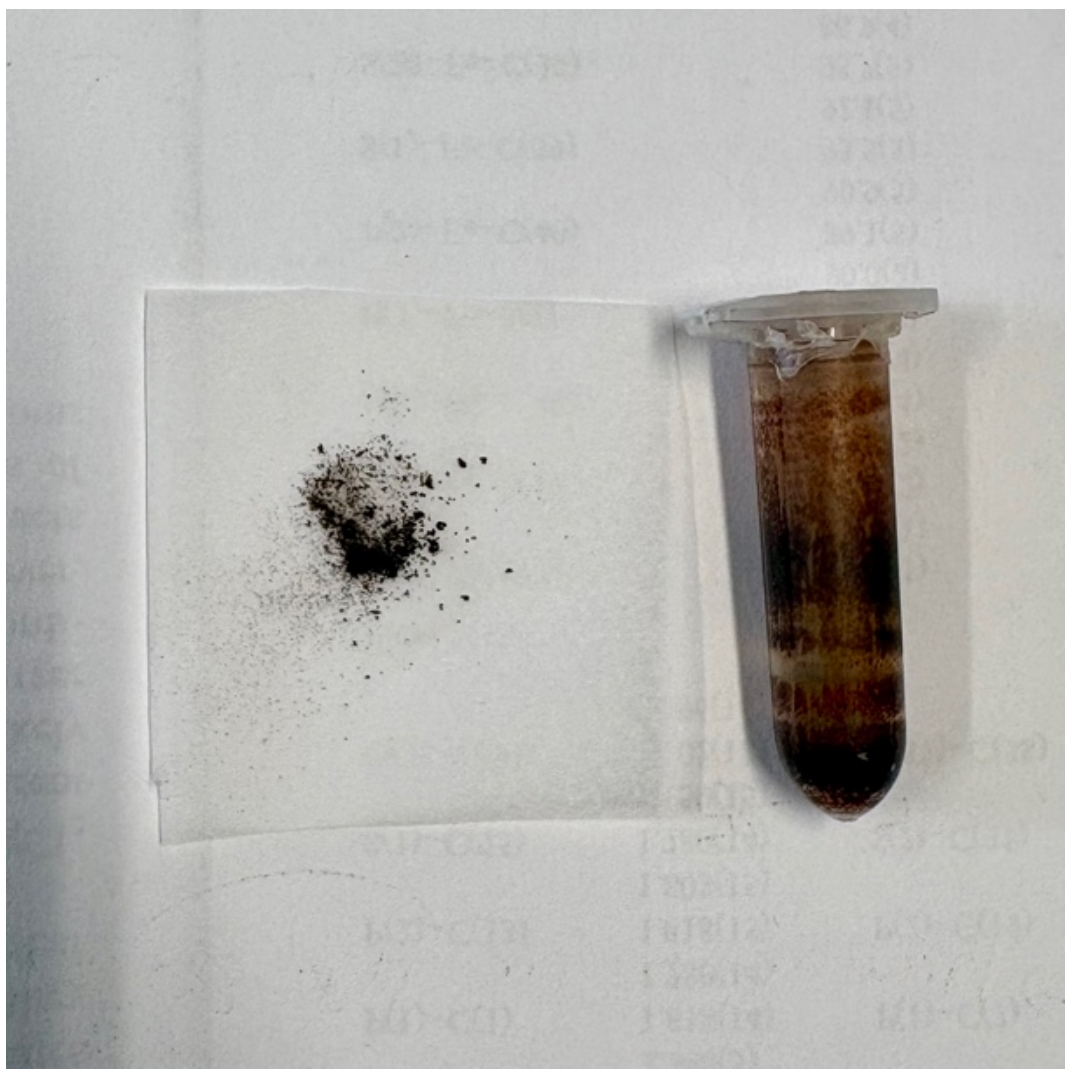

Figure S123. Post reaction mixture of $PtCl_2$ with 2,2';6',2"-terpyridine (1:1) (**4**) after 90 minutes of milling.

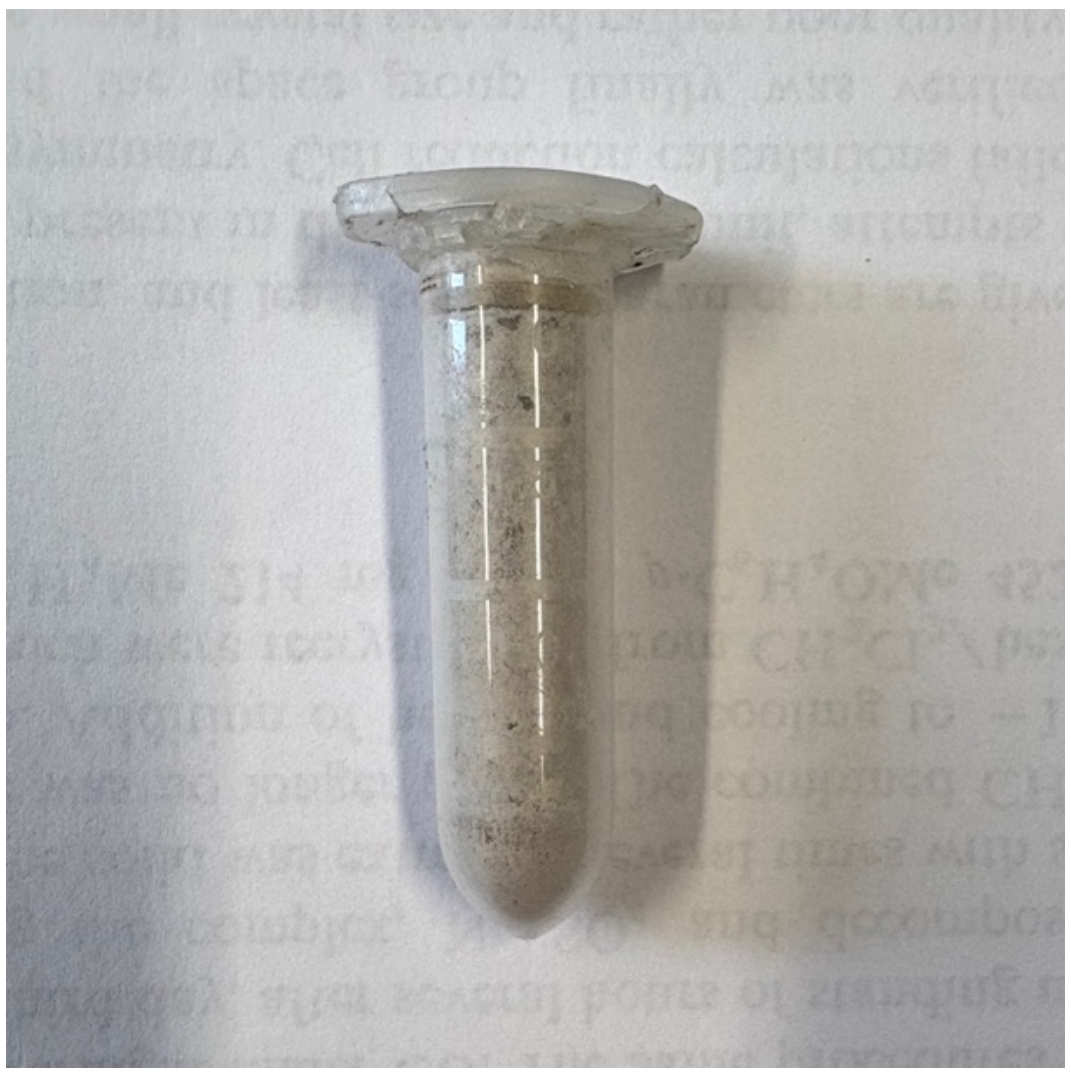

Figure S124. Post reaction mixture of $PtCl_2$ with PPh_3 (1:2) (**6**) after 15 minutes of milling.
